# Genome-Wide Identification and Expression, Protein–Protein Interaction and Evolutionary Analysis of the Seed Plant-Specific *BIG GRAIN* and *BIG GRAIN LIKE* Gene Family

**DOI:** 10.3389/fpls.2017.01812

**Published:** 2017-10-25

**Authors:** Bhuwaneshwar S. Mishra, Muhammed Jamsheer K, Dhriti Singh, Manvi Sharma, Ashverya Laxmi

**Affiliations:** National Institute of Plant Genome Research, New Delhi, India

**Keywords:** BIG GRAIN, BIG GRAIN LIKE, Phylogenetic analysis, gene family, auxin, ARF7/19

## Abstract

*BIG GRAIN1* (*BG1*) is an auxin-regulated gene which functions in auxin pathway and positively regulates biomass, grain size and yield in rice. However, the evolutionary origin and divergence of these genes are still unknown. In this study, we found that *BG* genes are probably originated in seed plants. We also identified that seed plants evolved a class of *BIG GRAIN LIKE* (*BGL*) genes which share conserved middle and C-terminal motifs with *BG*. The *BG* genes were present in all monocot and eudicot species analyzed; however, the *BGL* genes were absent in few monocot lineages. Both *BG* and *BGL* were found to be serine-rich proteins; however, differences in expansion and rates of retention after whole genome duplication events were observed. Promoters of *BG* and *BGL* genes were found to be enriched with auxin-responsive elements and the *Arabidopsis thaliana BG* and *BGL* genes were found to be auxin-regulated. The auxin-induced expression of *AthBG2* was found to be dependent on the conserved ARF17/19 module. Protein-protein interaction analysis identified that AthBG2 interact with regulators of splicing, transcription and chromatin remodeling. Taken together, this study provides interesting insights about *BG* and *BGL* genes and incentivizes future work in this gene family which has the potential to be used for crop manipulation.

## Introduction

In seed crops like rice, the grain size is an important agronomic trait which determines yield. Studies identified many important determinants of grain size in rice which has the potential to be used for crop manipulation (Zuo and Li, 2014). The *BIG GRAIN 1* (*OsaBG1*) has been recently identified as a positive regulator of grain size in rice. Interestingly, the constitutive expression of *OsaBG1* in *Arabidopsis thaliana* also resulted in an increase in seed size (Liu et al., 2015).

Apart from increase in seed size, enhanced expression of *BG1* in rice and *A. thaliana* resulted in an increase in biomass suggesting that this gene is a general promoter of growth (Liu et al., 2015). *OsaBG1* is a primary auxin response gene and increased expression resulted in enhanced auxin sensitivity suggesting that this gene is involved in auxin signaling. OsaBG1 is predominantly localized in the plasma membrane and overexpression lines showed enhanced auxin levels and basipetal transport suggesting that this gene might be involved in the regulation of auxin transport. These results suggest that *OsaBG1* is involved in enhancing biomass and grain size through regulating auxin pathway in rice (Liu et al., 2015). Different studies have already implicated auxin signaling in regulating biomass and seed size in both monocots and eudicots. The loss of *AUXIN RESPONSIVE FACTOR 2* of *A. thaliana* results in an increase in seed size due to enhanced cell proliferation while ectopic expression of *AUXIN RESPONSIVE FACTOR 19* (*ARF19*) from *Jatropha curcas* promotes seed size and yield (Schruff et al., 2005; Sun et al., 2017). Decreased synthesis of auxin in *Pisum sativum* resulted in small and wrinkled seeds with reduced starch content (McAdam et al., 2017). In rice, the *NARROW LEAF1*, an inhibitor of polar auxin transport was found to be a suitable candidate gene for crop engineering for better yield (Qi et al., 2008; Fujita et al., 2013; Takai et al., 2013; Zhang et al., 2014).

In field trials, the *OsaBG1* overexpressing transgenic lines showed a significant increase in yield suggesting that *BG* genes are ideal candidates for crop improvement (Liu et al., 2015). The *BG* homologs were found to be present in eudicots. However, except the single study in rice, so far, no attempt has been made to study the *BG* genes in eudicots or any other monocot species. The enhanced biomass and bigger seed phenotype of *OsaBG1*-expressing *A. thaliana* lines suggest that the *BG*-dependent pathway of growth regulation might be conserved in all angiosperms (Liu et al., 2015). Many important seed crops such as legumes belong to eudicotyledonae and the potential of *BG* genes in crop improvement makes them ideal candidates to be studied in eudicots. Although *BG* genes were identified from selected monocots and eudicots, the origin and evolution of these genes in plants are yet to be identified.

In this study, we made use of the availability of a large number of sequenced genomes in the plant lineage to trace the evolutionary origin and evolution of *BG* genes. The comprehensive phylogenetic analysis suggest that *BG* genes are originated in seed plants. We also identified a group of *BIG GRAIN LIKE* (*BGL*) genes, which show sequence similarity with BG in some motifs and seems to be originated along with *BG* genes. Detailed phylogenetic analysis identified difference in the rate of retention and expansion among *BG* and *BGL* genes. Expression analysis of *A. thaliana BG* and *BGL* genes in different developmental stages was done and the spatiotemporal expression dynamics of these genes were identified. Further, the promoter analysis and auxin-dependent expression analysis of *A. thaliana BG* and *BGL* genes suggest the conservation of auxin-dependent transcriptional control of *BG* and *BGL* genes. Finally, we identified that AthBG2, a member of *A. thaliana* BG family; interacts with conserved regulators of splicing, transcription and chromatin remodeling. This comprehensive analysis of *BG* and *BGL* genes in plants can serve as a springboard for future work in this agronomically important class of genes.

## Materials and Methods

### Identification of BG and BGL Proteins

The BG and BGL proteins from different sequenced plant genomes were identified by BLASTP, iterated BLAST and profile-HMM based searches in various databases. The remaining BG proteins from *Oryza sativa* were identified from MSU Rice Genome Annotation Project Database using OsaBG1 as the query with default parameters (BLASTP, *e*-value: 1e-5) (Ouyang et al., 2007). The BG and BGL proteins from *Amborella trichopoda* were identified from Amborella Genome Database using OsaBG1 as the query (BLASTP, *e*-value: 1e-5) (Albert et al., 2013). The BG and BGL proteins from the remaining species were identified using AtrBG1 or AtrBGL1 as the query in BLASTP. The proteins from *Picea abies* were identified from ConGenIE (BLASTP, *e*-value: 1e-3) (Sundell et al., 2015). The BG and BGL proteins from *Nelumbo nucifera* and *Phoenix dactylifera* were identified from NCBI RefSeq database (BLASTP, *e*-value: 10) (Pruitt et al., 2007). The proteins from *Lotus japonicus* were identified from Lotus Base (BLASTP, *e*-value: 1e-5) (Mun et al., 2016). The proteins from the remaining species were identified from Phytozome v12 (BLASTP, *e*-value: -1) (Goodstein et al., 2012). The retrieved sequences were manually curated to remove repeats and partial sequences. These sequences were aligned and used for profile-HMM based search in hmmsearch available in HMMER web server v2.15.0 with default parameters in the respective reference proteomes (significance e-value: 0.01 for sequence and 0.03 for hits). Further, three rounds of iterated BLAST search was performed using the aligned sequences in different reference proteomes using jackhmmer available in HMMER web server v2.15.0 with default parameters (significance *e*-value: 0.01 for sequence and 0.03 for hits) (Finn et al., 2015). Finally, to identify the true BG/BGL hits, the hits obtained from profile-HMM and iterative searches were subjected to BLAST search in *A. thaliana* and/or *O. sativa* proteomes. The final set of verified protein sequences used for phylogenetic analysis is given in Supplementary Table S1. The corresponding CDS sequences were retrieved from the respective databases using the protein identifiers. The final set of CDS sequences used for phylogenetic analysis is given in Supplementary Table S2.

### Conserved Motif Analysis

Presence of characterized domains in BG and BGL proteins was analyzed by batch CD search tool against CDD v3.16 using the default parameters (*e*-value: 0.01) (Marchler-Bauer et al., 2015). The conserved motifs of BG and BGL proteins were identified from analyzing the aligned sequences of BG and BGL proteins from eudicots and monocots individually in Jalview (Waterhouse et al., 2009). The sequences of conserved motifs were exported from Jalview and used for HMM logo construction in Skylign (Wheeler et al., 2014). The fold recognition, topology and disorder prediction analysis was done using Phyre v2.0 with default parameters (Kelley et al., 2015).

### Phylogenetic Analysis

The phylogenetic trees were reconstructed based on Bayesian (MrBayes v3) and maximum likelihood (PhyML) estimation methods in TOPALI v2.5 (Milne et al., 2009). The protein and CDS sequences were aligned by ClustalX v2.1 (Larkin et al., 2007). The aligned file was used to identify the best-suited model of amino acid and DNA substitution in TOPALI v2.5 (Milne et al., 2009). JTT + I + G model was used for reconstruction of all protein-based phylogenetic trees (Jones et al., 1992). GTR + I + G model was used for the reconstruction of all DNA-based phylogenetic trees (Tavaré, 1986). For Bayesian phylogeny, two Metropolis-Coupled Markov Chain Monte Carlo runs with 16 chains were performed with the given settings [sample frequency: 10; burn in (%): 25] for 100,000 generations unless specified. The protein-based phylogenetic tree of eudicots was run for 500,000 generations to attain convergence. The protein-based phylogenetic tree of eudicots was run for 300,000 generations to attain convergence. The CDS-based phylogenetic tree of eudicots and monocots was run for 1,000,000 generations to attain convergence. To verify the convergence of the Bayesian phylogenetic reconstruction, potential scale reduction factor was confirmed to reach 1.0 as runs converged in MrBayes v3 (Ronquist et al., 2012). The maximum likelihood trees were constructed with 1000 bootstrap replicates. The species phylogenetic tree was obtained from NCBI Taxonomy Common Tree tool. The phylogenetic trees were edited and visualized with midpoint rooting in FigTree v1.4.3 (Rambout, 2016). The alignment files used for phylogenetic analysis and tree output obtained are deposited in Figshare^1^.

### Ka/Ks Ratio Calculation

The PAL2NAL program was used for Ka, Ks and Ka/Ks calculations (Suyama et al., 2006). The Ka/Ks ratios of paralogous and orthologous genes identified from the phylogenetic reconstruction were calculated.

### Expression and Promoter Analysis and Promoter: GUS Line Construction

The AtGenExpress expression data of *A. thaliana BG* and *BGL* genes in different developmental stages and auxin treatments were retrieved from Arabidopsis eFP browser (Schmid et al., 2005; Winter et al., 2007; Goda et al., 2008). The response of *BG* and *BGL* genes towards auxin treatments were calculated from this data. The values were plotted as heat maps using MultiExperiment Viewer (MeV, v4.8) (Saeed et al., 2006). For the qRT-PCR analysis of *AthBG2* in different developmental stages, the tissue and developmental stage-specific samples previously prepared in the lab were used (Jamsheer et al., 2015). The cDNA was prepared from 2 μg of total RNA using High-Capacity cDNA Reverse Transcription kit (Applied Biosystems, United States). The 1:20 diluted cDNA samples were used for qRT-PCR reaction in 7500 Fast Real-Time PCR System using SYBR-Green chemistry as per the manufacture’s protocol (Applied Biosystems, United States). The qRT-PCR primers of *AthBG2* and the endogenous reference *UBQ10* were designed from the transcript region using Primer Express v3.0 (Applied Biosystems, United States). The primers used for qRT-PCR are given in Supplementary Table S4. The relative quantification was performed using ΔΔCT method (Livak and Schmittgen, 2001).

For the promoter analysis, 1kb upstream sequences from the TSS of *A. trichopoda, A. thaliana* and *O. sativa BG* and *BGL* genes were retrieved from Phytozome v12 (Goodstein et al., 2012) (Supplementary Table S3). The presence of different auxin responsive elements reported in the literature (TGTCNN, NNGACA, ACTTTA, CACGCAAT, TGACG, and KGTCCCAT) was analyzed and plotted according to the scale. For the expression analysis of *AthBG2* toward auxin treatment in *Col-0*, 5 days old seedling grown under standard growth conditions (16:8 h photoperiod, 60 μmol m^-2^ s^-1^ light and 22°C ± 2°C temperature) were subjected to 1 μM IAA treatment in 0.5X liquid MS medium for different time points. For gene expression comparison between WT and *arf7arf19* mutant, 7 days old seedlings grown under standard growth conditions were subjected to 1 μM IAA treatment in 0.5X liquid MS medium for different time points. The tissue was harvested at the end of time points in liquid nitrogen and stored in -80°C. RNA isolation and cDNA preparation from this tissue were performed as described previously (Mishra et al., 2009). The qRT-PCR experiment and calculation were performed as described above. The seeds of *arf7arf19* were obtained from ABRC (CS24625) (Okushima et al., 2005).

For the promoter: GUS line construction of *AthBG2*, 2 kb upstream region from the TSS was cloned in pENTR/D-TOPO using the primer given in Supplementary Table S4 and mobilized to pMDC164 (Curtis and Grossniklaus, 2003) vector using Gateway technology (Invitrogen, Carlsbad, CA, United States). The *pAthBG2:: GUSA* construct was transformed in *Col-0* grown in standard growth conditions using floral dip method (Clough and Bent, 1998). The transformants were identified by screening the seeds in 0.5X MS medium with 15 μg/ml hygromycin A and homozygous lines were identified in the third generation. For GUS assay, the homozygous lines were grown under the standard growth conditions. The tissues were harvested and GUS assay was performed as described previously (Jefferson et al., 1987).

### Yeast Two-Hybrid Assay

The *AthBG2* CDS cloned in pGBKT7 (BD vector) and transformed into Y2H gold strain was used as the bait to screen the normalized Mate & Plate Universal Arabidopsis Y2H cDNA library as per the manufactures’ protocol (Clontech, Mountain View, CA, United States). The primers used for cloning are given in Supplementary Table S4. Before the Y2H screening, the construct was tested for auto activation and toxicity respectively on SD/–Leu/X-α-Gal/AbA (SDO/X/A) and SD/–Leu (SDO) plates. After identifying that the construct lacked auto activation and toxicity properties, it was used for Y2H library screening. The colonies obtained from SD/–Leu/–Trp/X-α-Gal/AbA (DDO/X/A) after Y2H library screening were further screened on SD/–Ade/–His/–Leu/–Trp/X-α-Gal/AbA (QDO/X/A) medium. From the colonies survived on QDO/X/A medium, prey plasmids (pGADT7; AD vector) were isolated and sequenced to identify the interacting protein. The interaction was further confirmed by co-transforming bait and recovered prey plasmids in Y2H gold strain and screening on QDO/X/A medium. A negative control experiment was conducted along with this experiment using bait vector and prey plasmids to identify the false interactors.

### Bimolecular Fluorescent Complementation and Subcellular Localization

For the BiFC experiments, *AthBG2* and *Y14* CDS were cloned into pENTR/D-TOPO vector and mobilized respectively to pSAT4A-DEST-n(1-174)EYFP-N1 and pSAT5-DEST-c(175-END)EYFP-C1 (Tzfira et al., 2005) vectors using Gateway technology (Invitrogen, Carlsbad, CA, United States). Primers used for cloning are given in Supplementary Table S4. The clones were cotransformed in onion peel using PDS-1000 Helios Gene Gun (Biorad). The transformed onion peels were incubated at 22°C in darkness for 24 h and fluorescence was analyzed in TCS SP2 (AOBS) laser confocal scanning microscope after the incubation (Leica Microsystems). Negative control experiments were conducted along with this experiment.

For the subcellular localization of AthBG2, the CDS cloned into pENTR/D-TOPO vector was mobilized to pEG104 (Earley et al., 2006) vector using Gateway technology (Invitrogen, Carlsbad, CA, United States). The vector and YFP-CDS construct were transformed individually in onion peels and fluorescence was analyzed as described above.

## Results

### Evolutionary Origin and Expansion of *BG* and *BGL* Genes

A combination of BLAST and HMM-based methods were employed to identify BG proteins from the selected sequenced viridiplantae members. Homology search in some of the sequenced chlorophyte genomes (*Chlamydomonas reinhardtii, Ostreococcus lucimarinus, Volvox carteri*, and *Micromonas pusilla*) could not identify any homologs of OsaBG1. We also searched for the BG proteins in *Klebsormidium flaccidum*, which belongs to Charophyta and shows prototypes of signaling pathways required for terrestrial life (Hori et al., 2014). However, we couldn’t retrieve any homologs of OsaBG1 from this genome either. Further, iterative searches also failed to identify any hits of BG proteins in the algal genomes we analyzed, indicating that BG proteins are absent in algae.

**FIGURE 1:**
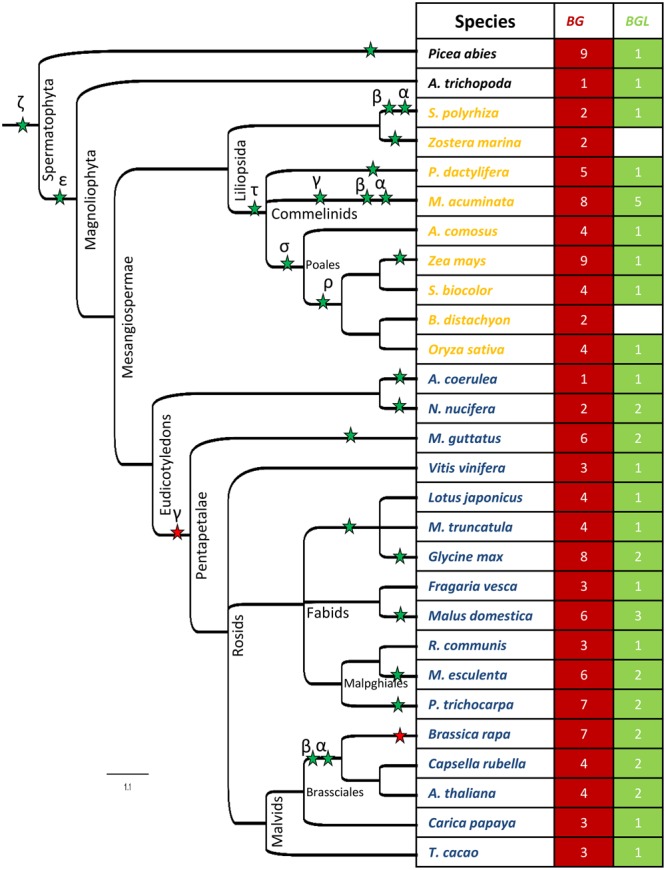
The distribution of *BG* and *BGL* genes in different plant genomes. The numbers represent the distribution of *BG* and *BGL* genes in each species. The asterisks indicates the WGD events in the plant lineages. The green and red asterisks indicate genome duplication and triplication respectively.

We next analyzed the presence of BG1 homologs in embryophytes. The searches in Bryophyta (*Marchantia polymorpha, Physcomitrella patens* and *Sphagnum fallax*) and Pteridophyta (*Selaginella moellendorffii*) could not identify any hits of BG1. Interestingly, 9 hits of BG proteins were identified from the gymnosperm, *Picea abies* (**Figure 1**). Further, a hit of BG1 was identified from *A. trichopoda*, which is the only living species belonging to the sister lineage of angiosperms (Albert et al., 2013). In both *P. abies* and *A. trichopoda*, BLAST search identified another protein which has similarity with the BG proteins in the middle and C-terminal regions (**Figure 1**, Supplementary Figure S1). We named these proteins as BIG GRAIN LIKE (BGL). Consistent with the sequence divergence observed among BG and BGL proteins, in the Bayesian phylogenetic interference of *A. trichopoda* and *P. abies* BG and BGL proteins, they recovered to distinct clades (Supplementary Figure S2). Relatively large number of BG proteins in *P. abies* indicates the possible contribution of whole genome duplications (WGD) in the expansion of *BG* genes in spermatophytes. In the plant lineage, there is evidence for an ancient WGD event (ζ) shared by seed plants and another WGD event (𝜀) common to angiosperms (Jiao et al., 2011). The absence of lineage-specific WGD in the *Amborella* lineage and the occurrence of Pinaceae-specific WGD event can be attributed to the increase in the number of *BG* genes in *P. abies* (Albert et al., 2013; Li Z. et al., 2015).

To dissect the evolutionary history of *BG* and *BGL* genes in angiosperms, we identified the *BG* and *BGL* genes from 17 eudicots and 9 monocots which represent important taxonomical positions (**Figure 1**). In eudicots, *BG* and *BGL* genes were found to be present in all species analyzed. Among the early diverging eudicots species analyzed, *Aquilegia coerulea* was found to have single *BG* and *BGL* genes each while their number was found to be increased to 2 in *Nelumbo nucifera*. Interestingly, both these genomes underwent lineage-specific WGD (Ming et al., 2013; Tiley et al., 2016). Further, the number of *BG* genes in majority of eudicot genomes without independent WGD (*Vitis vinifera, Fragaria vesca, Ricinus communis, Carica papaya* and *Theobroma caca*o) (Jaillon et al., 2007; Ming et al., 2008; Chan et al., 2010; Argout et al., 2011; Shulaev et al., 2011) events were found to be increased to 3 suggesting that the core eudicot genome triplication event contributed to this expansion (Jiao et al., 2012). However, in these species, the number of *BGL* genes remained 1 as in *A. trichopoda*. An expansion in the number of *BG* genes was observed in *Mimulus guttatus*, which can be attributed to the Lamiales-specific WGD event (Edger et al., 2016). Even in this species, only modest increase in the number of *BGL* genes was observed (**Figure 1**). These results indicate the difference in the rate of retention of *BG* and *BGL* genes after WGD events.

Legumes share an ancient papilionoid-specific WGD event that occurred ∼ 54 Mya (Pfeil et al., 2005; Bertioli et al., 2009). Apart from this ancestral WGD, *Glycine max* underwent an independent WGD event ∼ 13 Mya (Schmutz et al., 2010). Consistent with this, the legumes without independent WGD (*Lotus japonicus* and *Medicago truncatula*) (Sato et al., 2008; Young et al., 2011) possess 4 *BG* genes and 1 *BGL* gene each while *G. max* possesses 8 *BG* genes and 2 *BGL* genes (**Figure 1**). A similar increase in the number of *BG* and *BGL* genes was also observed in other species with independent WGD (*Malus domestica, Manihot esculenta, Populus trichocarpa* and *Brassica rapa*) (Tuskan et al., 2006; Velasco et al., 2010; Wang et al., 2011; Bredeson et al., 2016) events. The modest increase in the number of *BG* and *BGL* genes in *C. rubella* and *A. thaliana* could be due to the β and α duplications in crucifers (Bowers et al., 2003; Panchy et al., 2016).

In monocots, among the species analyzed, we could not identify BGL proteins from *Zostera marina* and *Brachypodium distachyon* even after repeated searches using various strategies (**Figure 1**). However, BGL proteins were found to be present in all other monocot species. The available evidence suggests that the monocot-specific τ duplication occurred after the divergence of Alismatales from other monocots (Jiao et al., 2014; Ming et al., 2015). The modest increase in the number of *BG* genes in species belonging to Alismatales (*Zostera marina* and *Spirodela polyrhiza*) can be attributed to the lineage-specific WGD event(s) (Wang et al., 2014; Olsen et al., 2016). The σ WGD shared by Poales and ρ WGD common to grasses might have contributed to the expansion of *BG* genes in *Ananas comosus, Zea mays, Sorghum bicolor, Brachypodium Distachyon*, and *Oryza sativa* (Paterson et al., 2004; Tang et al., 2010; Ming et al., 2015). As observed in eudicots, in the monocots with independent WGD (*P. dactylifera, Musa acuminata* and *Z. mays*) (Schnable et al., 2009; D’Hont et al., 2012; Al-Mssallem et al., 2013) event(s), the number of *BG* genes are further increased. However, except in *M. acuminata*, not much increase in the number of *BGL* genes was observed indicating the difference in the rate of retention in *BG* and *BGL* genes in monocots also.

### Identification of the Conserved Motifs in BG and BGL

The preliminary analysis of representative BG proteins from eudicots and monocots suggest that this is a novel class of protein with no known protein domains (Liu et al., 2015). To find out whether the BG and BGL proteins acquired any known domains, we analyzed all 124 BG and 40 BGL proteins identified from different species for the presence of known protein domains. Almost in all species we analyzed, the size of BGL proteins was found to be smaller than BG (Supplementary Figure S3). Our analysis revealed that except two BG proteins from *Z. mays* and one protein from *S. bicolor*, BG and BGL proteins do not possess any known functional domains (Supplementary Figure S4). The middle region of SbiBG2 and ZmaBG3 show partial similarity with the domain present in γ and τ subunits of DNA polymerase III (Jergic et al., 2007) (Supplementary Figures S4A,C,D). Similarly, the mid region of ZmaBG2 shows partial similarity with the domain present in the δ subunit of DNA polymerase III (Song et al., 2001) (Supplementary Figures S4B,D). Apart from these remote similarities, BG and BGL proteins did not show any significant similarities with any known protein domains suggesting that they belong to a novel class of proteins.

**FIGURE 2:**
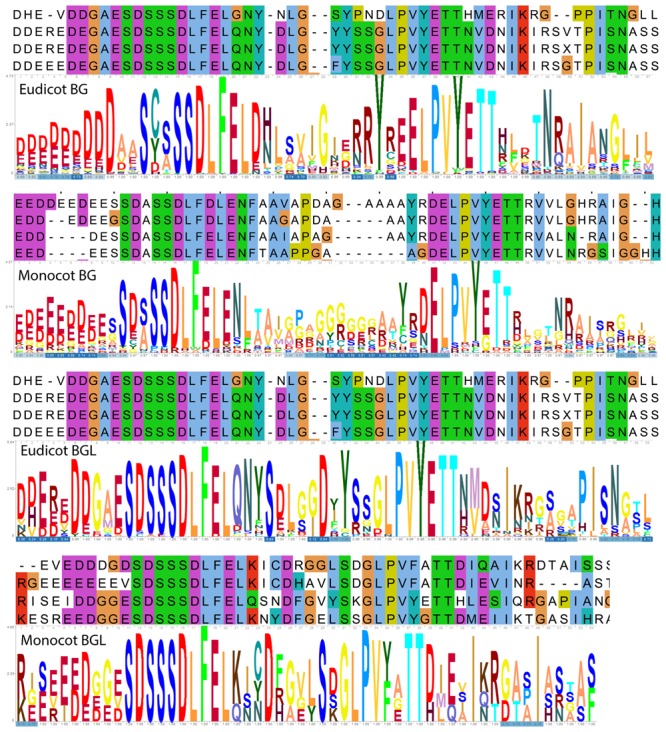
The conserved C-terminal motif in BG and BGL protein family in eudicots and monocots. The sample alignment is given on the top and HMM logo of the motif is given in the bottom for each group.

Since the domain search revealed that BG and BGL proteins do not possess any known domains, we analyzed the BG and BGL proteins from eudicots and monocots individually for the presence of conserved motifs shared across different species. As observed in the BLAST results, BG and BGL proteins in both eudicots and monocots show the presence of a conserved C-terminal region (**Figure 2**). This region was also found to be conserved among the *P. abies* BG and BGL proteins (Supplementary Figure S1). The N-terminal of this region was found to be enriched with glutamic acid and aspartic acid repeats and showed a propensity for disorder in BG and BGL proteins we analyzed (Supplementary Figure S5). Further, many other amino acids were also found to be conserved in this C-terminal motif in the BG and BGL proteins in both eudicots and monocots (**Figure 2**). The topology prediction of the C-terminal motif of BG proteins from *A. trichopoda, O. sativa* and *A. thaliana* identified that this region has high propensity to form helices (Supplementary Figure S5A). However, analysis of fold similarity with known structures identified very low similarity suggesting that this C-terminal region is a novel domain (Supplementary Table S5). Topology prediction of the C-terminal motif of BGL proteins from *A. trichopoda* and *A. thaliana* predicted different topologies. The C-terminal motif of AtrBGL1 and AthBGL1 showed an N-terminal repeat of three small beta sheets followed by a C-terminal short helix while the shorter C-terminal motif of AthBGL2 predicted to have only the repeats of beta sheets (Supplementary Figure S5B). Analysis of fold similarity of the C-terminal motif of BGL2 also showed weak similarities with known structures (Supplementary Table S5).

Further, we identified four other conserved motifs present in BG proteins in both eudicots and monocots (Supplementary Figure S6). Similarly, conservation analysis identified two other conserved motifs in BGL proteins in both eudicots and monocots (Supplementary Figure S7). The motif III of BG proteins was found to have similarity with the N-terminus of the long motif II of BGL indicating that this is another region where the BG and BGL protein show similarity. The motif analysis identified that both BG and BGL proteins show many repeats of serine, glutamic acid, aspartic acid, arginine and lysine. As observed in the motif analysis, amino acid composition analysis of BG and BGL protein found the highest enrichment of serine followed by lysine, glutamine, arginine etc (Supplementary Figure S8).

### Phylogenetic Analysis of BG and BGL Protein Family

**FIGURE 3:**
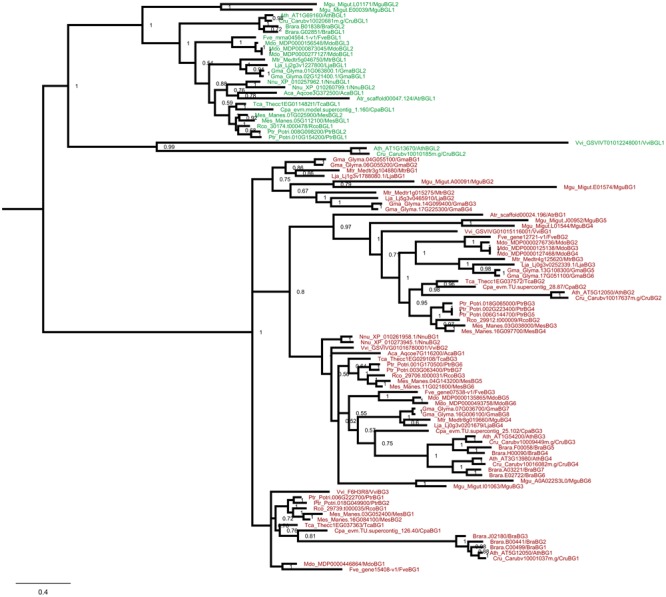
Phylogenetic analysis of BG and BGL proteins from eudicots. Bayesian phylogenetic reconstruction of BG and BGL proteins from eudicots and *A. trichopoda* based on JTT + I + G model. The posteriori probability values are given adjacent to the branches. The BG and BGL proteins are marked by red and green color respectively.

In order to get more insight into the protein evolution, Bayesian phylogenetic reconstruction was used to decipher the evolutionary relationship of BG and BGL proteins in eudicots and monocots. The eudicot and monocot phylogenetic tree of BG and BGL protein was constructed individually along with the BG and BGL proteins from *A. trichopoda*. The eudicot BG and BGL proteins were recovered to separate clades in the phylogram (**Figure 3**). In the BGL clade, one protein each from *V. vinifera, C. rubella* and *A. thaliana* formed a clade with strong probability (posterior probability support value: 1) from other BGL proteins. We annotated them as BGL proteins because sequence alignment shows that it shares similarity with other BGL proteins in many regions. However, they also showed more sequence divergence compared to other BGL proteins (Supplementary Figure S9A). Further, in the individual phylogenetic tree with *A. trichopoda* BG and BGL proteins, these proteins were closely positioned with AtrBGL1 (Supplementary Figures S9B–D). The formation of separate clade from other BGL proteins can be due to the increased sequence divergence observed in these proteins and the consequent long-branch attraction (Kolaczkowski et al., 2009). Interestingly, in the BG proteins, we recovered four major sub-clades with strong probability (posterior probability support value: 0.8–1) suggesting sequence variation among the eudicot BG proteins. The BG proteins from eudicots without independent WGD were found to be positioned in most of these sub-clades suggesting the early origin of this sequence divergence. In the sub-clade recovered in our analysis, the positioning of BG proteins from different species fairly reflect the eudicot phylogeny (Zeng et al., 2017), suggesting the involvement of concerted evolution in protein divergence. The same pattern was also observed in the BGL sub-clades. The recovery of clades of BG and BGL proteins and different sub-clades in both groups is also observed in a maximum likelihood based estimation of the phylogenetic tree supporting the inferences obtained from the Bayesian phylogenetic reconstruction (Supplementary Figure S10). Further, we reconstructed the Bayesian phylogram of *BG* and *BGL* genes of eudicots using CDS sequences. As observed in the protein phylograms, the *BG* and *BGL* genes recovered to separate clades in the CDS-based phylogram (Supplementary Figure S11).

**FIGURE 4:**
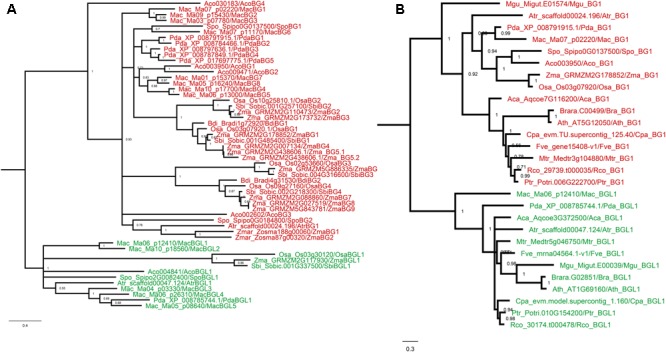
Phylogenetic analysis of BG and BGL proteins from monocots and eudicots. **(A)** Bayesian phylogenetic reconstruction of BG and BGL proteins from monocots and *A. trichopoda*. **(B)** Bayesian phylogenetic reconstruction of selected BG and BGL proteins from angiosperms. The phylograms were reconstructed on the basis of JTT + I + G model and the posteriori probability values are given adjacent to the branches. The BG and BGL proteins are marked by red and green color respectively.

The recovery of BG and BGL proteins into separate clades was also observed in the Bayesian phylogram of monocots (**Figure 4A**). Further, BG proteins from monocots were also recovered to sub-clades with strong probabilities (posterior probability support value: 0.71–1). The maximum likelihood based estimation of the phylogenetic tree also supported these inferences (Supplementary Figure S12). However, in the Bayesian phylogenetic reconstruction using CDS sequences, the *BGL* genes from Poaceae were recovered to a clade closer to *BG* genes while other *BGL* genes formed another clade. Further, the solitary *BG* gene from *A. trichopoda* and one *BG* gene each from *Z. marina* and *S. polyrhiza* positioned a branch in between the two clades of *BGL* genes (Supplementary Figure S13).

Taken together, the phylogenetic analysis of BG and BGL proteins from eudicots and monocots indicates the possible role of concerted evolution in the protein divergence. Strikingly, the recovery of BG and BGL proteins into distinct clades in the phylogenetic analysis supporting the occurrence of distinct motifs specific to BG and BGL proteins. To further validate this observation, a phylogram was constructed with eudicot and monocot BG and BGL proteins (**Figure 4B**). Irrespective of the ancient monocot-eudicot divergence, the BG and BGL proteins recovered to separate clades supporting the hypothesis that the origin of both types of proteins predates the divergence of angiosperms.

### Analysis of Selection Pressure on *BG* and *BGL* Genes

**FIGURE 5:**
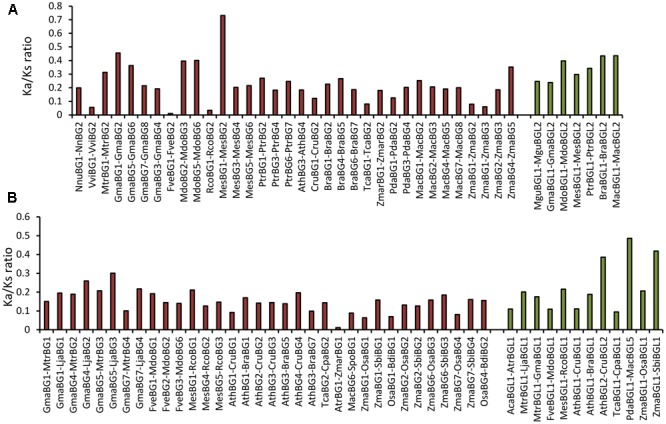
Analysis of selection pressure on *BG* and *BGL* genes. **(A)** The Ka/Ks ratio of paralogous *BG* and *BGL* genes from eudicots and monocots. **(B)** The Ka/Ks ratio of orthologous *BG* and *BGL* genes from eudicots and monocots.

To identify the nature of evolutionary pressure acting on *BG* genes over time, the Ka/Ks ratio of 23 eudicot and 11 monocot putative paralogous gene pairs identified from the phylogenetic reconstruction was calculated. The Ka/Ks ratio of paralogous *BG* genes from eudicot was found to be ranging from 0.0108 to 0.7304 (Avg. 0.246) (**Figure 5A**). The Ka/Ks ratio of monocot *BG* genes was found to be ranging from 0.0601 to 0.3519 (Avg. 0.185) (**Figure 5A**). These results indicate strong purifying selection acting on *BG* genes in both eudicots and monocots. Similarly, we calculated the Ka/Ks ratios of 6 eudicots and 1 monocot putative paralogous *BGL* gene pairs. The Ka/Ks ratios of *BGL* genes were found to be ranging from 0.238 to 0.434 (Avg. 0.326) in eudicots. The Ka/Ks ratio of *MacBGL1* and *MacBGL2* was estimated to be 0.435 (**Figure 5A**). These results suggest that both *BG* and *BGL* genes are under purifying selection in angiosperms.

To get better insight into the nature of selection in *BG* and *BGL* genes in closely related species, we calculated Ka/Ks ratio of putative orthologous genes inferred from the phylogenetic reconstruction (**Figure 5B**). The Ka/Ks ratio of 22 orthologous *BG* genes from eudicot was found to be ranging from 0.0917 to 0.3008 (Avg. 0.168). Similarly, the Ka/Ks ratio of 12 orthologous *BG* genes from monocots was found to be ranging from 0.0116 to 0.1851 (Avg. 0.116) confirming the purifying selection among *BG* genes. In 9 orthologous *BGL* eudicot genes analyzed, the Ka/Ks ratio varied from 0.0941 to 0.3856 (Avg. 0.177). Similarly, in the 3 orthologous *BGL* genes analyzed, the Ka/Ks ratio varied from 0.2065 to 0.4864 (Avg. 0.370) (**Figure 5B**). Taken together, these results confirm that both *BG* and *BGL* genes are under purifying selection in angiosperms.

### Tissue and Developmental Stage-Specific Expression of *A. thaliana BG* and *BGL* Genes

**FIGURE 6:**
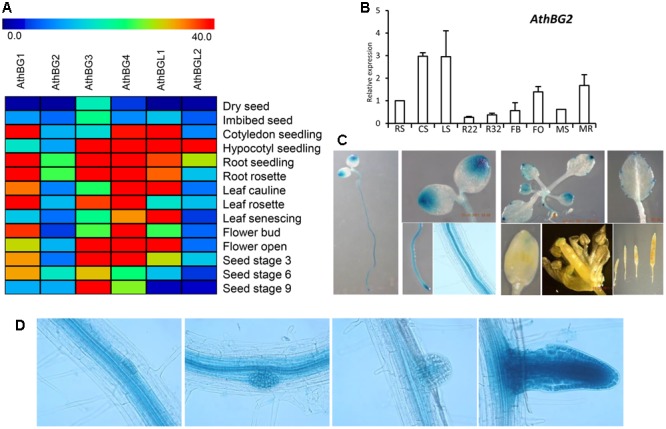
Tissue and developmental stage-specific expression analysis of *A. thaliana BG* and *BGL* genes. **(A)** Heat map showing the expression of *A. thaliana BG* and *BGL* genes in different tissues and developmental stages **(B)** The qRT-PCR expression analysis of *AthBG2* in different developmental stages and tissues. The expression in radicle stage was taken as control to compare the expression in other stages and tissues. *UBQ10* was used as the endogenous control. The graph was plotted from the values of two biological replicates with three technical replicates each. The abbreviation of samples: RS, seeding radicle stage; CS, seeding cotyledon stage; seedling two-leaf stage; R22, rosette 22 days old; R32, rosette 32 days old; FB, flower bud; FO, flower open; MS, mature silique; MR, mature root. **(C)** Promoter activity of *AthBG2* in different tissues and developmental stages of *A. thaliana*. **(D)** Promoter activity of *AthBG2* during lateral root development.

For further analysis of *BG* and *BGL* gene family, we chose the model system *A. thaliana* which possess 4 *BG* and 2 *BGL* genes (**Figure 1**). The expression analysis of *A. thaliana BG* and *BGL* genes based on microarray data identified that these genes are expressed more in the seedling stages. Further, the expression of these genes was found to be more in the root, leaf, and flower and seed stages (**Figure 6A**). To validate the microarray expression data, we checked the expression of *AthBG2* in different tissues and developmental stages using qRT-PCR (**Figure 6B**). In microarray data, the expression of *AthBG2* was found to be high in the roots of different stages, leaf, flower etc. In qRT-PCR analysis, expression of *AthBG2* was found to be high in seedling of cotyledon and 2 leaves stage. The expression was found to be relatively high during seed germination, flower and root of the mature rosette. However, the expression was found to be relatively less in the rosette before and after bolting (**Figure 6B**). We further analyzed the promoter activity of *AthBG2* using promoter: GUS assay (**Figure 6C**). In seedling stage, the *AthBG2* promoter was found to be active at cotyledon tip and stelar region of root and at the root tip. In mature leaves, the promoter activity was mainly restricted to serration tips. A small amount of promoter activity was observed in the stigma region. Further, promoter activity of *AthBG2* was observed during lateral root development in the young primordia and enhanced expression was observed in young lateral root (**Figure 6D**). Taken together, the expression analysis of *A. thaliana BG* and *BGL* genes identified that these genes are expressed profusely in the seedling stages. Interestingly, the promoter: GUS assay of *AthBG2* identified that the promoter activity of this gene is overlapping with the activity of *DR5: GUS* auxin-responsive reporter, which show maximum activity in the regions of auxin accumulation such as root tip and cotyledon tip (Ulmasov et al., 1997). Further, the promoter activity of *AthBG2* overlaps with the expression domain of genes such as *ARF7, ARF19, INDOLE-3-ACETIC ACID INDUCIBLE 2, LATERAL ORGAN BOUNDARIES-DOMAIN 16 (LBD16), LBD29*, and *LBD33* which are involved in the regulation of auxin-dependent development (Okushima et al., 2005, 2007; Hay et al., 2006).

### Auxin-Dependent Expression of *A. thaliana BG* and *BGL* Genes

**FIGURE 7:**
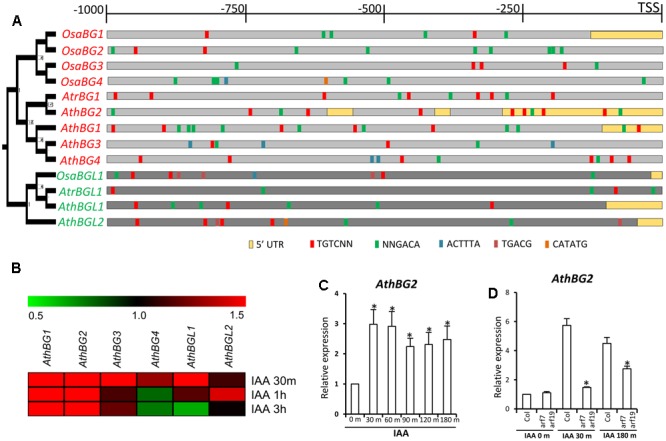
Promoter analysis and auxin-responsive expression study of *BG* and *BGL* genes. **(A)** Auxin-responsive elements in the *BG* and *BGL* promoters *A. trichopoda, A. thaliana* and *O. sativa* along with the Bayesian phylogram of proteins. **(B)** Heat map showing the auxin-dependent expression of *A. thaliana BG* and *BGL* genes. **(C)** The qRT-PCR expression analysis of *AthBG2* in response to IAA treatments. Asterisk indicates a significant difference in expression in treatment in comparison to untreated control (*P* < 0.05, Student’s *t*-test). **(D)** The qRT-PCR expression analysis of *AthBG2* in response to IAA treatments in *Col-0* and *arf7arf19* double mutant. Asterisk indicates a significant difference in expression in the mutant in response to 30 or 180 min IAA treatment in comparison to the response in the WT (*P* < 0.05, Student’s *t*-test). The graphs of qRT-PCR experiments were plotted from the values of two biological replicates with three technical replicates each. *UBQ10* was used as the endogenous control for both experiments.

The *OsaBG1* is a primary auxin-responsive gene (Liu et al., 2015). The promoter: GUS assay of *AthBG2* suggests that this gene might be involved in auxin signaling (**Figures 6C,D**). These results indicate that the auxin-dependent regulation of *BG* genes might be evolutionarily conserved. As the first step to test this hypothesis, we analyzed the presence of different auxin-responsive elements in the promoter and 5′UTR region of *BG* and *BGL* genes of *A. trichopoda, O. sativa* and *A. thaliana* (**Figure 7A**). The promoter and 5’UTR regions of both *AtrBG1* and *AtrBGL1* were found to be enriched with various auxin-responsive elements suggesting that the auxin-responsive expression of *BG* and *BGL1* genes predates the angiosperm divergence. In rice, similar to *OsaBG1*, the promoter of other *BG* genes were also found to be enriched with auxin-responsive elements. The same pattern is also observed in the promoters of *BG* and *BGL* genes from *A. thaliana* (**Figure 7A**). As evident in the promoter analysis, the auxin-dependent expression analysis of *A. thaliana BG* and *BGL* genes using the available microarray data identified that these genes are responsive to auxin in various degrees (**Figure 7B**). The expression of *AthBG1* and *AthBG2* was found to be induced in response to 30 min of auxin treatment and increased expression was retained in treatments of 1 and 3 h. The expression of *AthBG3* was induced in 30 min and in 1- and 3-h time points, expression was found to be almost similar to control levels. The expression of *AthBG4* was slightly induced in 30 min treatment and showed a small decrease in expression in the later time points. Among the *BGL* genes, *AthBGL1* expression was induced in response to 30 min treatment and repressed in response to 3 h treatment. In contrast, the expression of *AthBGL2* was induced in response to 1-h treatment (**Figure 7B**). These results indicate that the auxin-dependent regulation of expression of *BG* genes is conserved in monocots and eudicots. Further, the expression of *BGL* genes was also found to be responsive to auxin. In order to validate the microarray-based expression data, qRT-PCR expression analysis of *AthBG2* was done at various time points of auxin treatments (**Figure 7C**). As observed in the microarray data, the expression of *AthBG2* was found to be induced in 30 min and the increase in expression was found to be retained in all other time points. In rice, the increased expression of *OsaBG1* results in promotion of growth and increased grain size possibly through regulating auxin signaling pathway (Liu et al., 2015). In response to auxin, ARF7-ARF19 module redundantly activates cell cycle and positively regulates lateral and adventitious root formation and leaf cell expansion (Wilmoth et al., 2005; Okushima et al., 2007; Ito et al., 2016). Similar to *OsaBG1*, expression of *AthBG2* was found to be constitutively induced in response to auxin treatment at different time points (**Figure 7C**). Since *OsaBG1* is involved in the promotion of growth, we analyzed whether the auxin-dependent expression of *AthBG2* is dependent on ARF7-ARF19 module using the *arf7 arf19* mutant (Okushima et al., 2005) (**Figure 7D**). In the qRT-PCR analysis, the expression of *AthBG2* was found to be similar in the mutant and WT suggesting that ARF7 and ARF19 are not involved in regulating the transcription of *AthBG2* in normal conditions. As observed in previous experiment, the expression of *AthBG2* was found to be induced in WT in response to auxin; however, this induction was found to be significantly abolished in the *arf7 arf19* mutant in both time points studied suggesting the role of ARF7-ARF19 module in regulating the auxin-dependent expression of *AthBG2* (**Figure 7D**).

### Protein–Protein Interaction and Subcellular Localization Analysis of AthBG2

**FIGURE 8:**
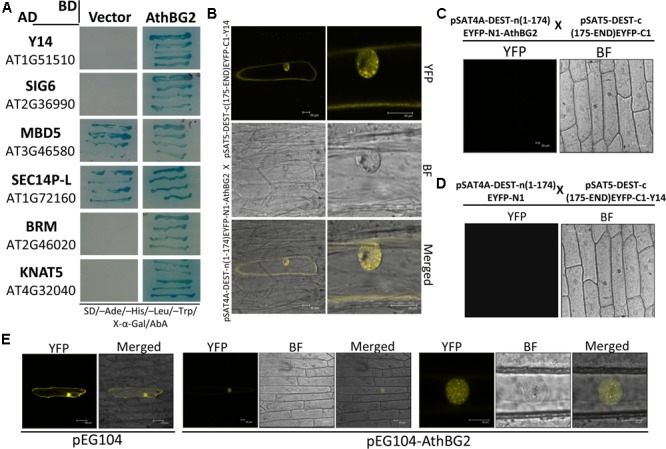
Protein-protein interaction and localization analysis of AthBG2. **(A)** The interacting proteins of AthBG2 identified from the Arabidopsis Y2H library screening. **(B–D)** BiFC confirmation of AthBG2-Y14 interaction with negative control experiments. **(E)** Subcellular localization of AthBG2. For both BiFC and localization experiments, YFP was excited at 514 nm and fluorescence emission was recorded at 530 nm.

In order to get clues about the molecular function of AthBG2, we screened Arabidopsis Y2H library using AthBG2 as bait. We obtained 68 colonies after screening on QDO/X/A medium. Sequencing of these colonies identified 6 proteins in repeats (**Figure 8A**). Further screening to identify positive interaction confirmed 4 genuine interacting proteins of AthBG2. The 4 interacting proteins of AthBG2 were found to have conserved molecular functions. The Y14 is a conserved eukaryotic protein which is a part of exon junction complex (EJC) and involved in RNA splicing (Park and Muench, 2007; Koroleva et al., 2009; Mufarrege et al., 2011; Cilano et al., 2016). SIGMA FACTOR 6 (SIG6) is a chloroplast sigma factor of RNA polymerase involved in chloroplast gene expression (Schweer et al., 2009; Chi et al., 2010). BRAHMA (BRM) is an SWI/SNF chromatin remodeling ATPase which is involved in the regulation of gene expression in response to many extrinsic and intrinsic cues (Han et al., 2015). KNOTTED1-LIKE HOMEOBOX GENE 5 (KNAT5) is a homeobox transcription factor expressed majorly in the distal end of the epidermal cells in the elongation zone of primary root (Truernit et al., 2006).

In order to validate the Y2H result, we analyzed the interaction of AthBG2 with Y14 using BiFC assay. The AthBG2-Y14 interaction was found in both cytoplasm and nucleus confirming the Y2H result (**Figure 8B**). The removal of AthBG2 or Y14 abolished fluorescent signal confirming that this interaction is specific (**Figures 8C,D**). In the nucleus, the AthBG2-Y14 interaction was found to form speckle-like bodies. Y14 is reported to be localized predominantly in the nucleus (Park and Muench, 2007). Further, in the nucleus, Y14 was found in nucleolus and speckles (Koroleva et al., 2009). The subcellular localization analysis found that AthBG2 is predominantly localized in the nucleus, both in diffused and speckle-like form (**Figure 8E**).

## Discussion

The *BG* genes are recently identified as positive regulators of growth and grain size in rice (Liu et al., 2015). In this study, through homology-based searches in 39 viridiplantae genome, we suggest that *BG* genes are probably originated in seed plants. *BG* genes are absent in *S. moellendorffii*, which belongs to Lycopodiophyta. Since there is no sequenced monilophyte genome available, at this point we cannot rule out whether the origin of *BG* genes predates the dawn of seed plants. Interestingly, a class of *BGL* genes were also found to be originated at the same time. The BG and BGL proteins share a conserved C-terminal motif and a middle motif. Apart from these shared motifs, BG and BGL proteins possess many unique conserved regions which resulted in their recovery to separate clades in the phylogenetic reconstruction. In the CDS-based phylogenetic reconstruction of *BG* and *BGL* genes from monocots, the recovery to specific BG and BGL clades was not that distinct, which could be due to the accumulation of synonymous mutations in the coding region. However, even in monocots, the phylogenetic reconstruction based on protein sequences using both Bayesian and maximum likelihood methods recovered distinct clades of BG and BGL proteins confirming the early divergence of BG and BGL proteins.

Unlike the animal and fungi genomes, plant genomes are characterized by many ancestral and recent WGD events. As a consequence of this, on an average, approximately 65% of genes in plant genomes have duplicated copies (Panchy et al., 2016). The genome sequencing of many plants identified specific gain or loss of many genes and gene families which ensure the survival of the species in their local environment (Tuskan et al., 2006; Jaillon et al., 2007; Chan et al., 2010; Young et al., 2011; Hori et al., 2014). The functional analysis in rice identified that *OsaBG1* is a general promoter of growth and seed size (Liu et al., 2015). Our study predicts that origin of these genes coincide with the origin of seed habit (Linkies et al., 2010). In our analysis, we found that ancient and recent WGD increased the number of *BG* genes in most of the species. Interestingly, although *BGL* genes are also originated along with *BG* genes in seed plants before the divergence of angiosperms into eudicots and monocots, in eudicot species without independent WGD, their number is not increased suggesting the selective loss of *BGL* genes after the ancient eudicot genome triplication. Characteristically, in species with recent WGD event(s), the numbers of *BGL* genes have been increased suggesting the contribution of recent WGD events in the expansion of *BGL* genes in such species. Consistent with this, analysis of retention rate of duplicated genes after WGD events in *A. thaliana* identified that the rate of retention of duplicated genes is high in recent WGD events (Panchy et al., 2016).

Increased gene dosage provides a selective advantage for the organisms and therefore polyploids generally show hybrid vigor and increased resilience (del Pozo and Ramirez-Parra, 2015; Panchy et al., 2016). Since *BG1* gene in rice is a general growth promoter, the increased retention of these genes in comparison to *BGL* genes could be because of the selective advantage it can provide for the organisms. Along the same line, the reduced retention of *BGL* genes in angiosperms might have occurred because these genes might be acting as a competitive inhibitor of *BG* functions in a manner similar to microProteins (Eguen et al., 2015). This preliminary hypothesis can be tested in model species like *A. thaliana* which will provide interesting insights about the molecular connection between *BG* and *BGL* genes.

In our intra- and inter-species analysis, both *BG* and *BGL* genes were found to be under purifying selection. Consistent with this, although both these protein families do not have any known domains, they possess many novel motifs which are conserved across the plant kingdom. The gene duplication relaxes the selection pressure which results in the concomitant neo-functionalization or sub-functionalization and hypo-functionalization or non-functionalization in one or both duplicated genes. Eventually, the survivors of this selection would undergo strong purifying selection. In *BG* genes, the paralogous gene pairs from species without recent WGD (*V. vinifera, T. cacao, R. communis*, and *F. vesca*) events were found to be under more strong purifying selection than the species with recent lineage-specific or independent WGD (*A. thaliana, C. rubella, B. rapa, M. esculenta, M. domestica, M. truncatula, G. max, Z. mays*, and *M. acuminata*). Further, difference in the Ka/Ks values observed among paralogous gene pairs from different species can be due to the difference in the timing of WGD event in particular lineage and slow nucleotide substitution rate observed in species such as *P. dactylifera* and *N. nucifera* (Wilson et al., 1990; Ming et al., 2013).

The diverse expression pattern of *A. thaliana BG* and *BGL* genes in different developmental stages indicates their general role in regulating plant growth. The *OsaBG1* is expressed abundantly in the vascular tissue of culm and young panicles (Liu et al., 2015). Our expression analysis identified that *A. thaliana BG* and *BGL* genes show high expression in seedling tissues and flowers suggesting common expression domains in eudicots and monocots. Further, the promoter: GUS assay identified that similar to *OsaBG1, AthBG2* is expressed profusely in the vascular tissues. Interestingly, the expression of *A. thaliana BG* and *BGL* genes was high in roots while the expression of *OsaBG1* was found relatively low in roots indicating differences in the expression domain in eudicots and monocots. The divergence in the promoter regions in monocots and eudicots might have contributed to this divergence in expression domain.

Among the phytohormones, auxin acts as the major regulator of plant development and OsaBG1 is found to enhance growth through promoting auxin signaling (Ljung, 2013; Liu et al., 2015). Our expression study identified that *A. thaliana BG* and *BGL* genes are responsive to auxin in varying degrees suggesting that the auxin-dependent transcriptional regulation is a conserved mechanism by which the transcription of *BG* and *BGL* genes are controlled. Promoter analysis identified significant enrichment of auxin-responsive elements in the promoters of *AtrBG1* and *AtrBGL1* suggesting that the auxin-dependent transcriptional regulation predates the angiosperm divergence. The promoter: GUS assay of *AthBG2* identified that the promoter activity of this gene is high wherever localized auxin maxima is present. Further, the auxin-dependent expression of *AthBG2* was found to be mediated through the ARF7-ARF19 module. These results indicate auxin as a major regulator of transcription of *BG* and *BGL* genes. The root-specific expression and the role of ARF7-ARF19 in the regulation of auxin-dependent expression of *AthBG2* gene suggest the possible involvement of this gene in primary and lateral root development. Further, ARF19 from *J. curcas* is already implicated in the regulation of seed size and yield suggesting the interconnection between ARF19 pathway and seed development (Sun et al., 2017).

The localization analysis identified the predominant localization of AthBG2 in the nucleus. OsaBG1 is predominantly localized in the plasma membrane (Liu et al., 2015). These results suggest that BG genes from monocots and eudicots may have undergone divergence in the localization pattern which might be contributing to differences in the molecular functions. Protein-protein interaction analysis identified that AthBG2 interact with nuclear proteins with conserved molecular functions. AthBG2 interacts with Y14, which is the component of EJC, in the cytoplasm and nuclear speckles suggesting the role of AthBG2 in mRNA splicing. Further, both BG and BGL proteins were found to be serine-rich with multiple conserved serine repeats. This is a characteristic feature of proteins involved in splicing (Richardson et al., 2011). The interaction with SIG6 and KNAT5 suggest the possible role of AthBG2 in regulating transcription. The other interacting protein we identified from the Y2H library screening is BRM; which is an SWI/SNF chromatin remodeling ATPase reported to be essential for the maintenance of root stem cell niche through positively regulating auxin level and the transcription of specific *PIN* and *PLETHORA* genes (Yang et al., 2015). Further, BRM is involved in all aspects of plant growth from the embryonic to reproductive development and stress mitigation (Tang et al., 2008; Han et al., 2012; Li C. et al., 2015; Peirats-Llobet et al., 2016; Xu et al., 2016). The interaction of AthBG2 with BRM suggests its possible involvement in auxin-regulated processes like embryonic, root, shoot and reproductive development (Ljung, 2013). This comprehensive analysis of BG and BGL proteins reveals insights into their origin and expansion in plants. Further, the expression, localization and protein-protein interaction analyses highlight the possible differences between eudicot and monocot BG proteins and encourage more functional analysis in this gene family.

## Author Contributions

AL, BM, and MJ conceived and designed the study. MJ performed phylogenetic and bioinformatic analysis. BM performed cloning, promoter: GUS analysis and BiFC. MJ and BM performed the Y2H analysis. BM, DS, and MS performed qRT-PCR analysis. MJ, DS, and MS performed the Ka/Ks estimation. AL, BM, and MJ analyzed the data. MJ wrote the manuscript. All authors reviewed the manuscript

## Conflict of Interest Statement

The authors declare that the research was conducted in the absence of any commercial or financial relationships that could be construed as a potential conflict of interest.

## Footnotes

1 https://figshare.com/s/54e4cf77da0709bbc1fb
