# Supplementary material for: Genome-Wide Identification and Expression, Protein–Protein Interaction and Evolutionary Analysis of the Seed Plant-Specific BIG GRAIN and BIG GRAIN LIKE Gene Family
Source: Front Plant Sci. 2017 Oct 25;8:1812. doi: 10.3389/fpls.2017.01812 (PMC5660992; doi:10.3389/fpls.2017.01812)
Supplement: Supplementary file 1 [file Presentation_1.PDF]

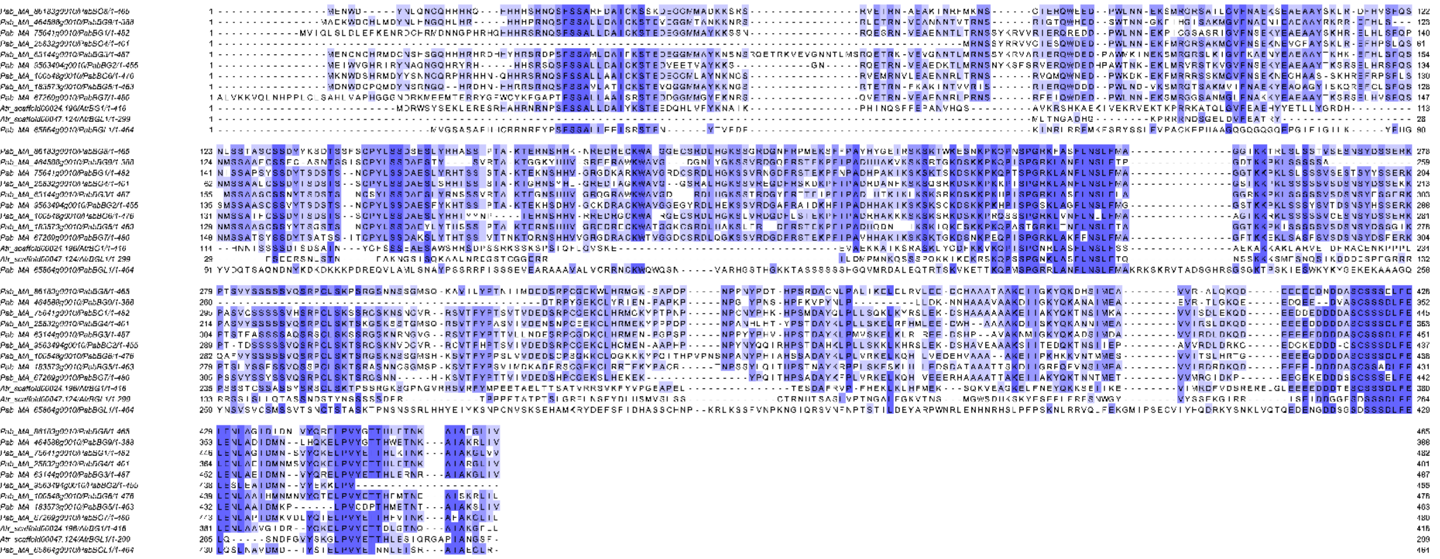

Figure S1. The alignment of BG and BGL proteins of *Picea abies* and *Amborella trichopoda*.

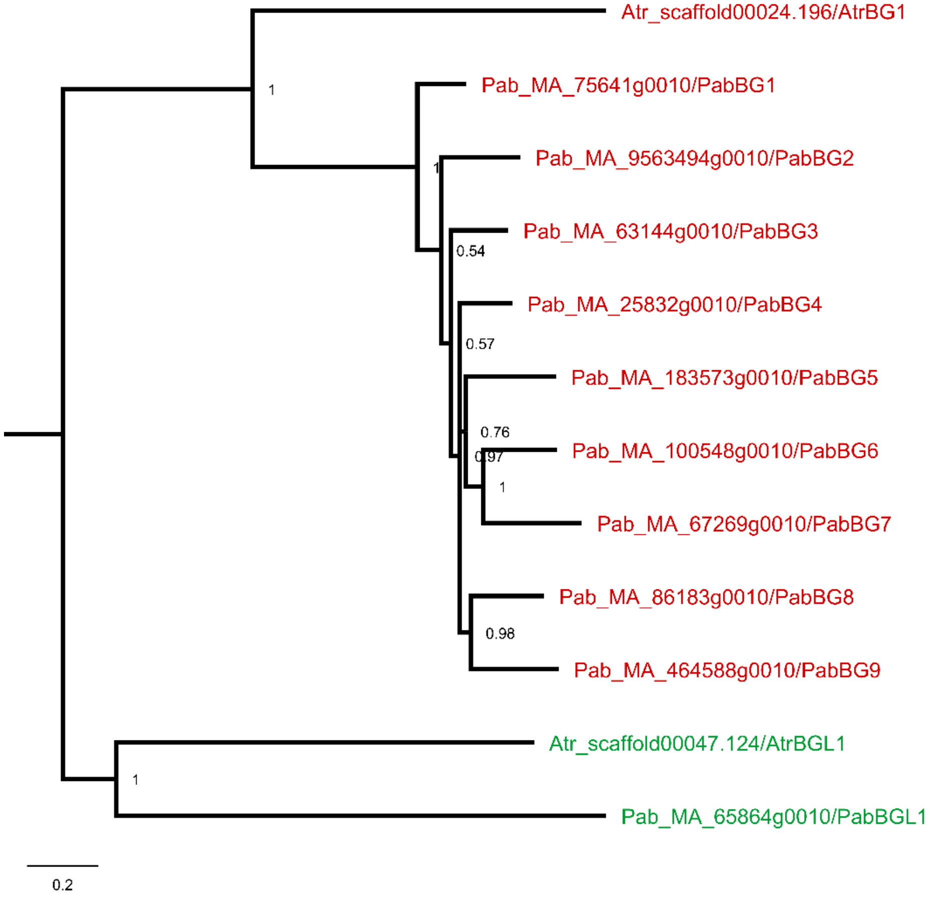

Figure S2. Phylogenetic analysis of BG and BGL proteins from *Picea abies* and *Amborella trichopoda*.

Bayesian phylogenetic reconstruction of BG and BGL proteins *Picea abies* and *Amborella trichopoda*. The phylogram was reconstructed on the basis of JTT+I+G model and the posteriori probability values are given adjacent to the branches.

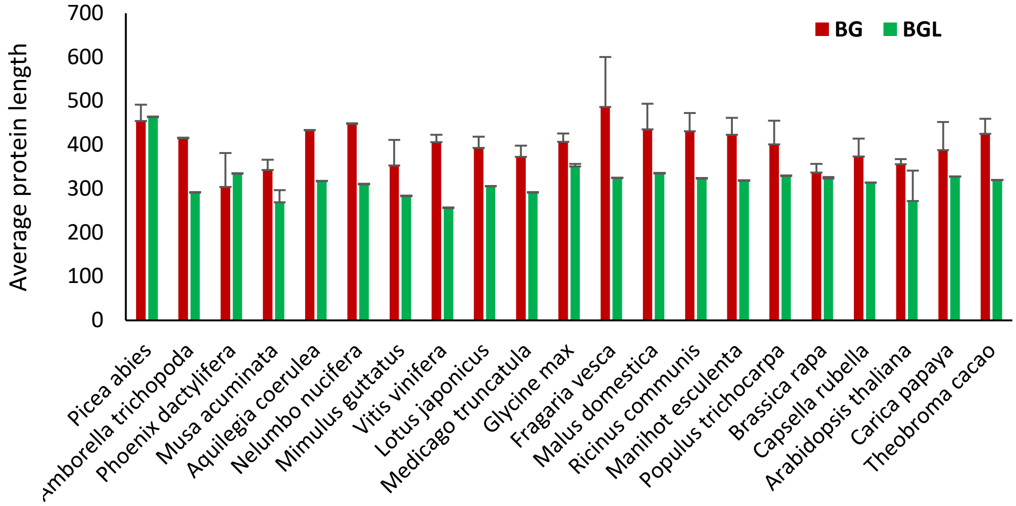

Figure S3. The protein length of BG and BGL in different species.
The bars represent protein length or average protein length (in species with more than one BG or BGL). The error bars represent + SD in species with more than one BG or BGL protein.

A

CD-length: 830 **E-value:** 3.61e-03 **Bitscore:** 39.06

```
                                    10        20        30        40        50
                            ....*....|....*....|....*....|....*....|....*....|
Sbi_Sobic.001G257100 127 APAEKQQAVPPGSATARRARKPSAAPASHGGCRRPASPGARLASLLNAif 176
Cdd:PRK07003         367 APGGGVPARVAGAVPAPGARAAAAVGASAVPAVTAVTGAAGAALAPKA-- 414

                                    60        70        80        90
                            ....*....|....*....|....*....|....*....|....*
Sbi_Sobic.001G257100 177 SGKRHSARQHPAPADDEPTACSTAPSSARPCLAKTPPSATARARA 221
Cdd:PRK07003         415 AAAAAATRAEAPPAAPAPPATADRGDDAADGDAPVPAKANARASA 459
```

B

CD-length: 620 **E-value:** 7.43e-03 **Bitscore:** 38.00

```
                                  10        20        30        40        50
                          ....*....|....*....|....*....|....*....|....*....|
Zma_GRMZM2G110473 106 EYSLRRLRPIRTSAGGVGPASVAPVEKQKPAPPgtakraRKPSAAPASGG 155
Cdd:PRK14954      364 ELALLRLIELVRNDGGVAPSPAGSPDVKKKAPE------PDLPQPDRHPG 407

                                  60        70        80        90       100
                          ....*....|....*....|....*....|....*....|....*....|
Zma_GRMZM2G110473 156 CRRPASPGARLASLLnaifSGKRNSARQHPAPADEEPACSTAPS-TARPC 204
Cdd:PRK14954      408 PAKPEAPGARPAELP----SPASAPTPEQQPPVARSAPLPPSPQaSAPRN 453

                          ....*..
Zma_GRMZM2G110473 205 LAKTPPS 211
Cdd:PRK14954      454 VASGKPG 460
```

C

CD-length: 618 **E-value:** 1.54e-04 **Bitscore:** 43.16

```
                                  10        20        30        40        50
                          ....*....|....*....|....*....|....*....|....*....|
Zma_GRMZM2G173732  83 CLSGRGYFSSSEVECS-----LRRLRPIRTSGGAgpASVAPEEKQQLAPP 127
Cdd:PRK14951      337 CLHGRAELGLAPDEYAaltmvLLRLLAFKPAAAA--EAAAPAEKKTPARP 384

                                  60        70        80        90       100
                          ....*....|....*....|....*....|....*....|....*....|
Zma_GRMZM2G173732 128 GAAMRARKPFATPASDSGNCRRPASPGARLASLLSAIFSGKRQHPAPADD 177
Cdd:PRK14951      385 EAAAPAAAPVAQAAAAPAPAAAPAAAASAPAAPPAAAPPAPVAAPAAAAP 434

                                 110       120
                          ....*....|....*....|...
Zma_GRMZM2G173732 178 EPACSTAPSSVRPCLAKTLPSAR 200
Cdd:PRK14951      435 AAAPAAAPAAVALAPAPPAQAAP 457
```

D

Query	Hit type	PSSM-ID	From	To	E-Value	Bitscore	Accession	Short name	Incomplete
Sbi_Sobic.001G257100/SbiBG2	superfamily	331207	127	221	0.003612	39.0624	cl26386	DNA_pol3_gamma3 superfamily	NC
Zma_GRMZM2G110473/ZmaBG2	superfamily	331068	106	211	0.007431	38.0003	cl26247	DNA_pol3_delta2 superfamily	NC
Zma_GRMZM2G173732/ZmaBG3	superfamily	331207	83	200	0.000154	43.1644	cl26386	DNA_pol3_gamma3 superfamily	NC

Figure S4. The CDD hits of BG and BGL proteins.
(A) The CDD hit of SbiBG2. (B) The CDD hit of ZmaBG2. (C) The CDD hit of ZmaBG3. (D) The hit details.

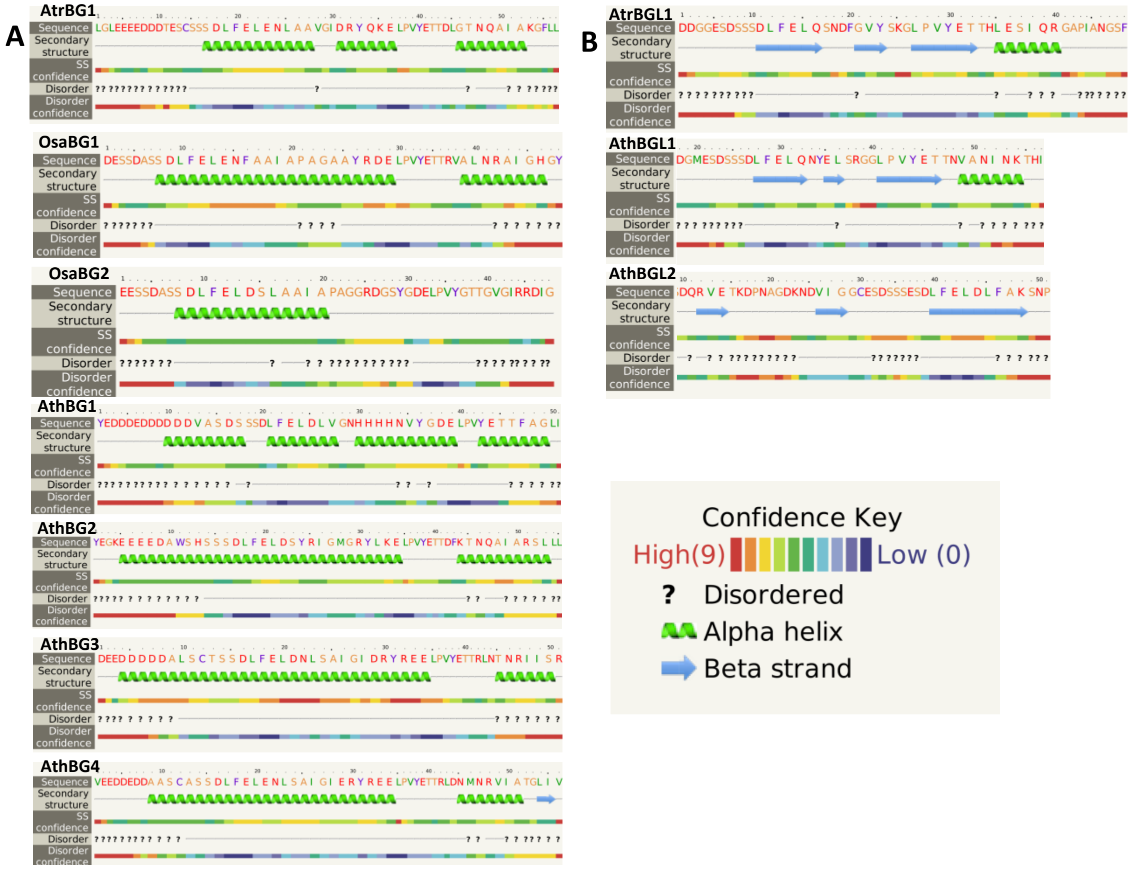

Figure S5. Topology and IDR prediction of C-terminal motif.

(A) and (B) Predicted topology and IDR propensity of C-terminal motifs of selected BG and BGL proteins respectively.

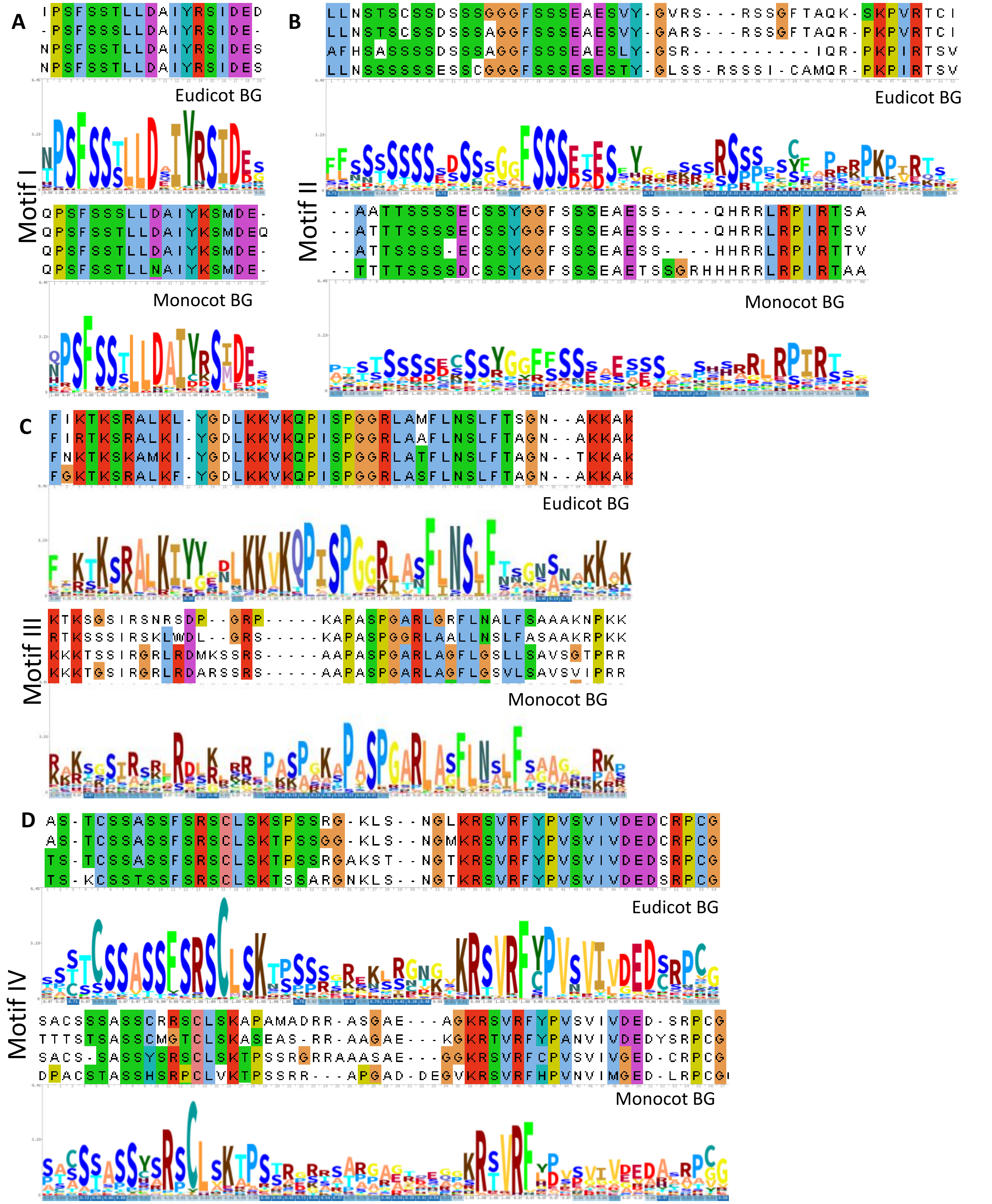

Figure S6. The conserved motifs identified in BG proteins.
(A) to **(D)** The four conserved motifs identified from the BG proteins in monocots and eudicots. Motifs were named according to the order of their distribution in proteins (N to C). The sample alignment for each motif is given on top and HMM logo is given in the bottom.

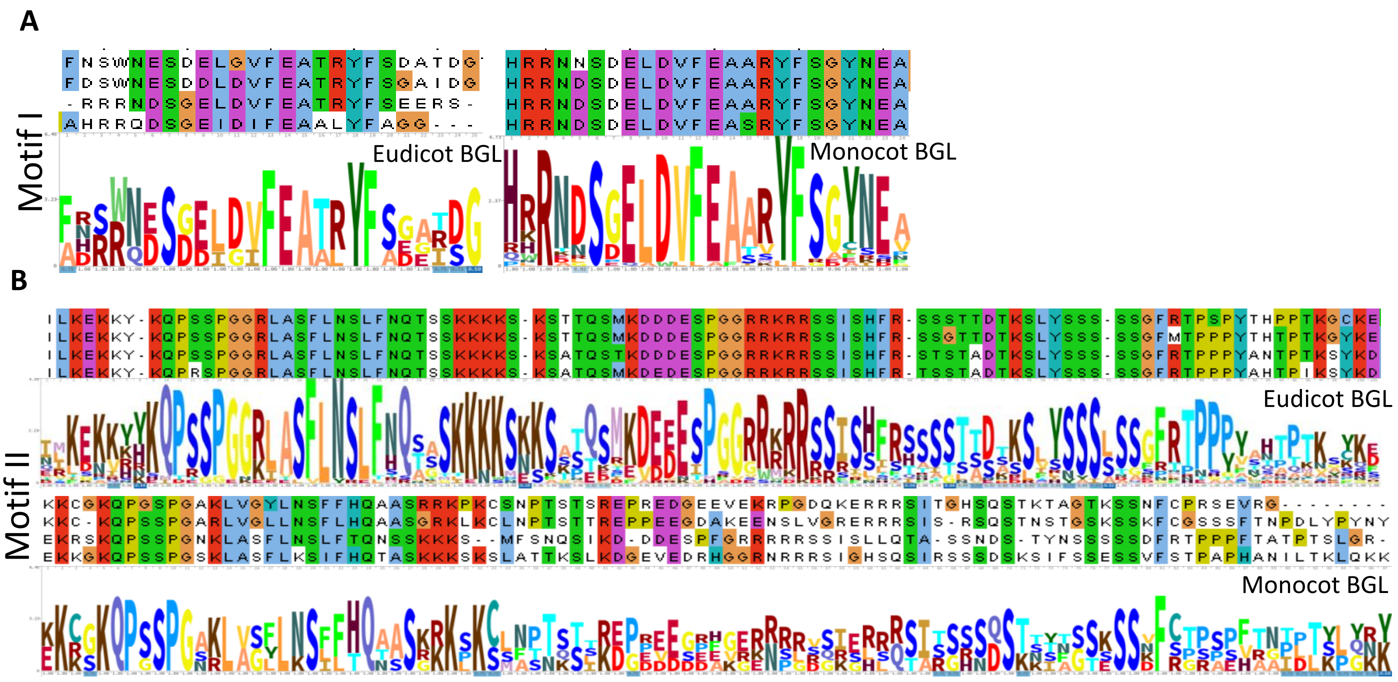

Figure S7. The conserved motifs identified in BGL proteins.
(A) and **(B)** The two conserved motifs identified from the BGL proteins in monocots and eudicots. Motifs were named according to the order of their distribution in proteins (N to C). The sample alignment for each motif is given on top and HMM logo is given in the bottom.

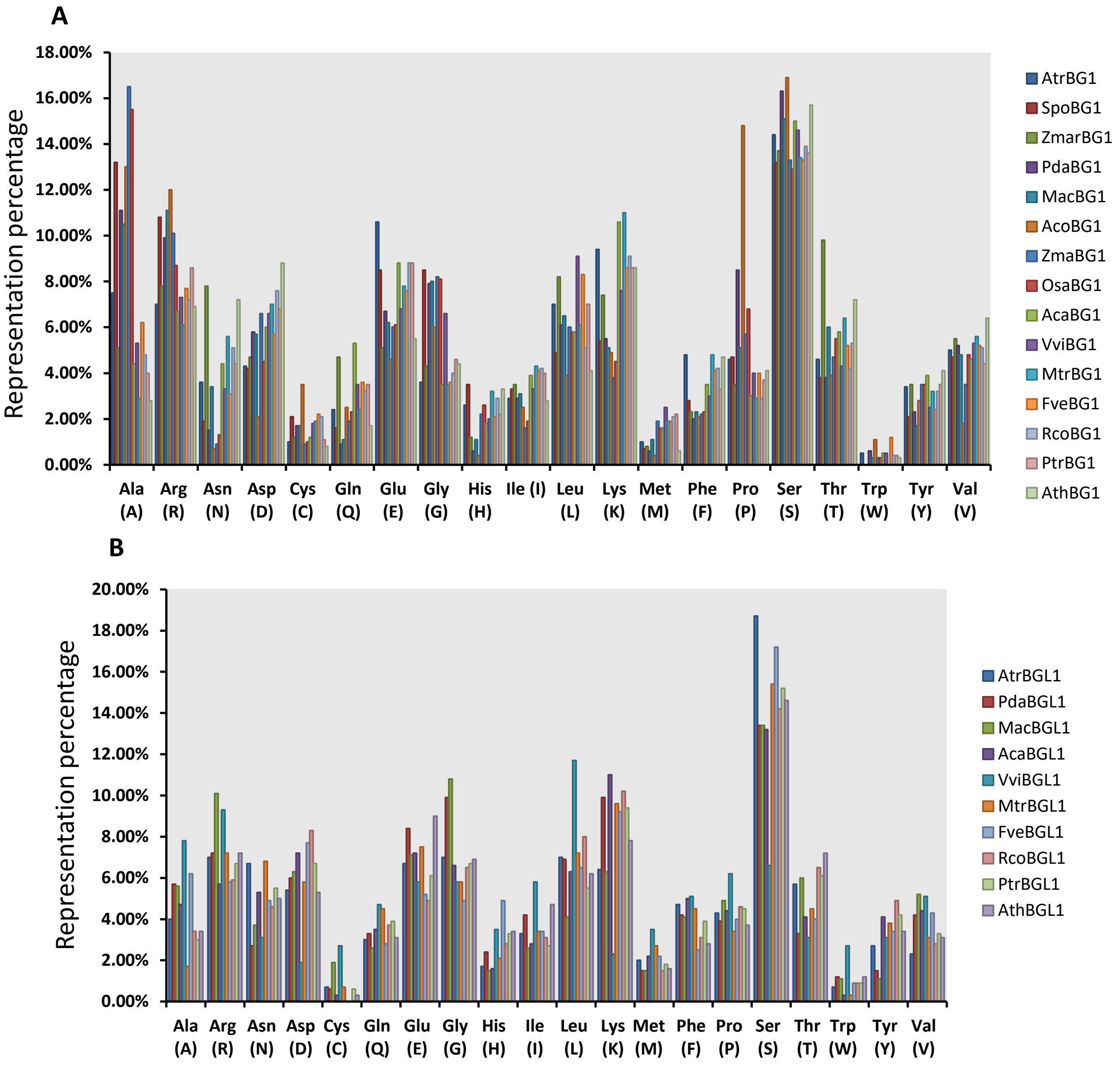

Figure S8. The amino acid composition of BG and BGL in different species.
The bars represent composition of each amino acid in percentage form.

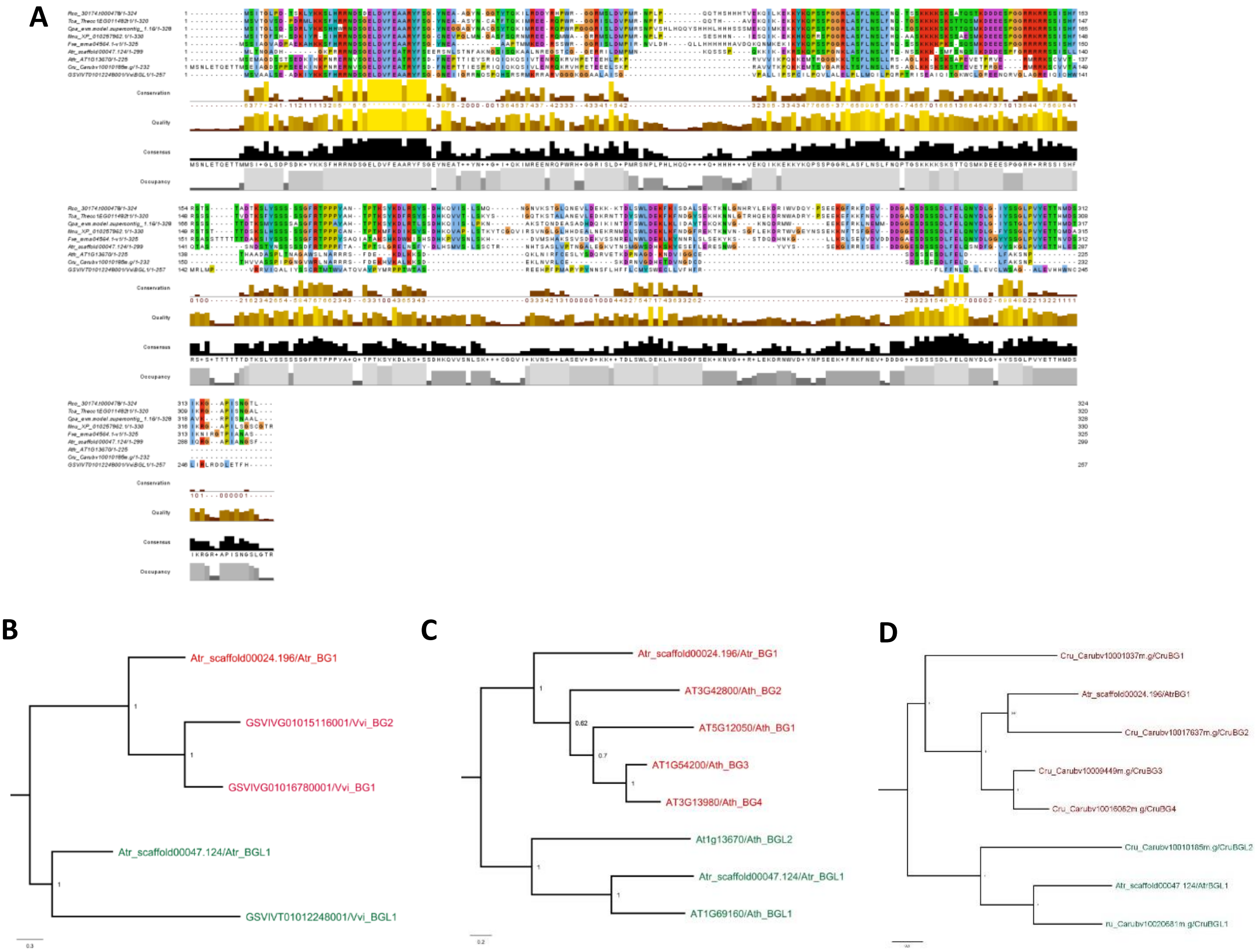

Figure S9. Phylogenetic analysis of BG and BGL proteins from *Vitis vinifera, Arabidopsis thaliana* and *Capsella rubella.*

(A) Sequence alignment of VviBGL1, AthBGL1 and CruBGL1 with other BGL1 proteins. **(B)** Bayesian phylogenetic reconstruction of BG and BGL proteins *V. vinifera* and the basal angiosperm *A. trichopoda*. **(C)** Bayesian phylogenetic reconstruction of BG and BGL proteins *A. thaliana* and the basal angiosperm *A. trichopoda*. **(D)** Bayesian phylogenetic reconstruction of BG and BGL proteins *C. rubella* and the basal angiosperm *A. trichopoda*. The phylograms were reconstructed on the basis of JTT+I+G model and the posteriori probability values are given adjacent to the branches.

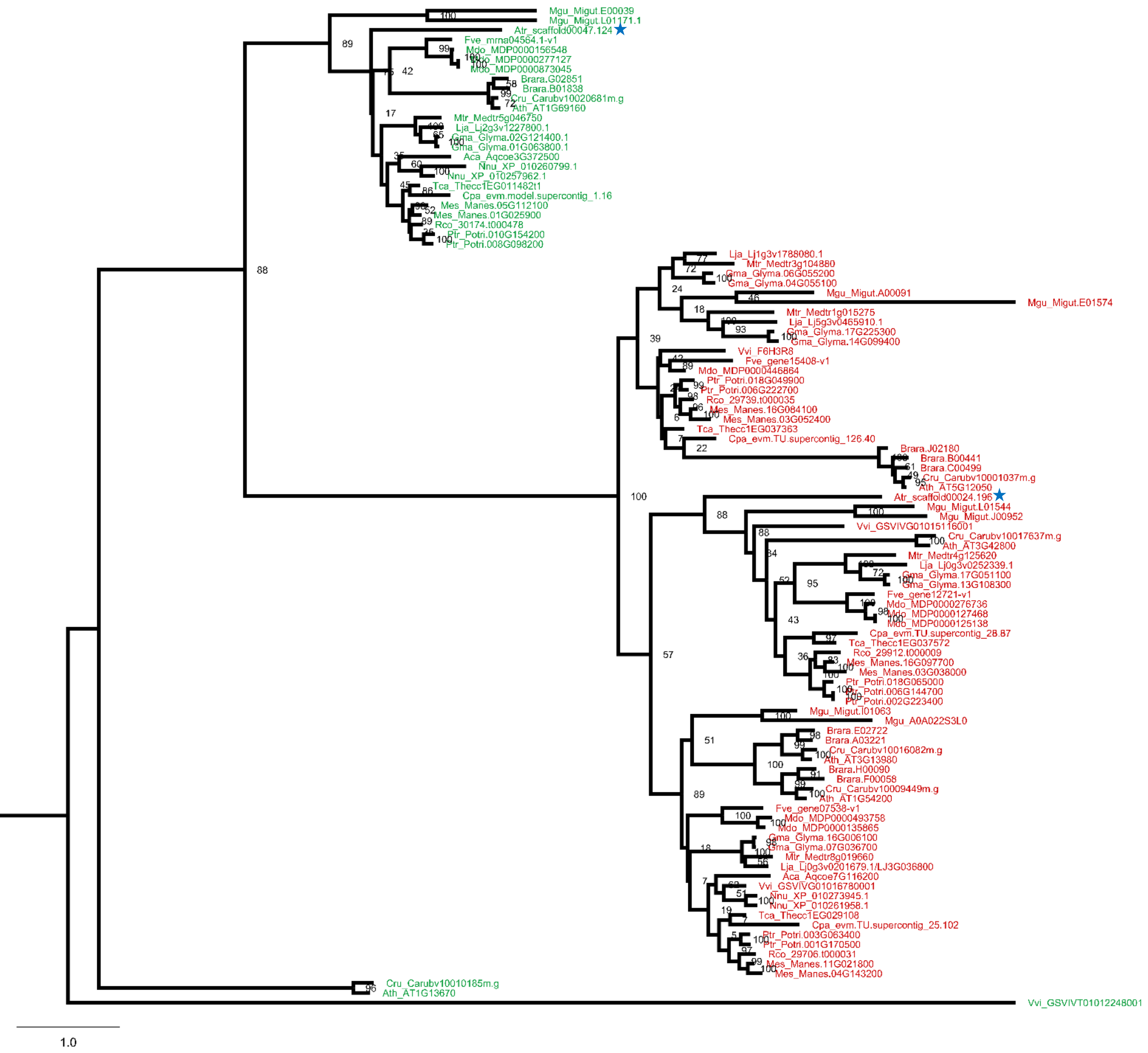

Figure S10. Maximum likelihood-based phylogenetic analysis of BG and BGL proteins from eudicots.

PhyML based phylogenetic reconstruction of BG and BGL proteins from eudicots and the basal angiosperm *A. trichopoda*. The phylogenetic tree was reconstructed based on JTT+I+G model. The bootstrap values based on 1000 replicates are given adjacent to the branches. AtrBG1 and AtrBGL1 are marked by blue asterisks. The BG and BGL proteins are marked by red and green colour respectively.

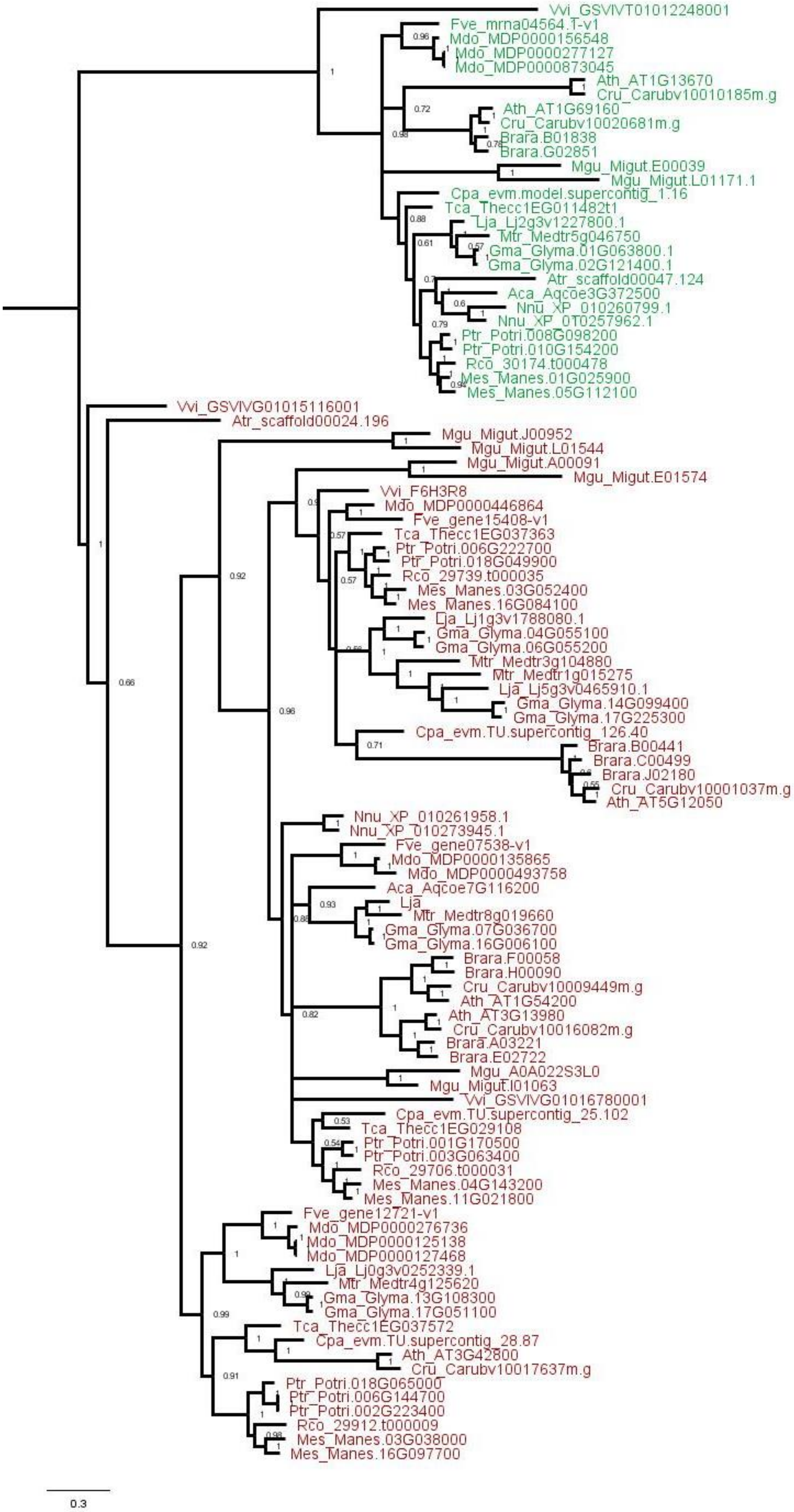

Figure S11. Phylogenetic tree of *BG* and *BGL* genes from eudicots inferred from CDS. Bayesian phylogenetic reconstruction of *BG* and *BGL* genes from eudicots and the basal angiosperm *A. trichopoda* based on GTR+I+G model. The CDS was used for the phylogram reconstruction. The posteriori probability values are given adjacent to the branches. The *BG* and *BGL* genes are marked by red and green colour respectively.

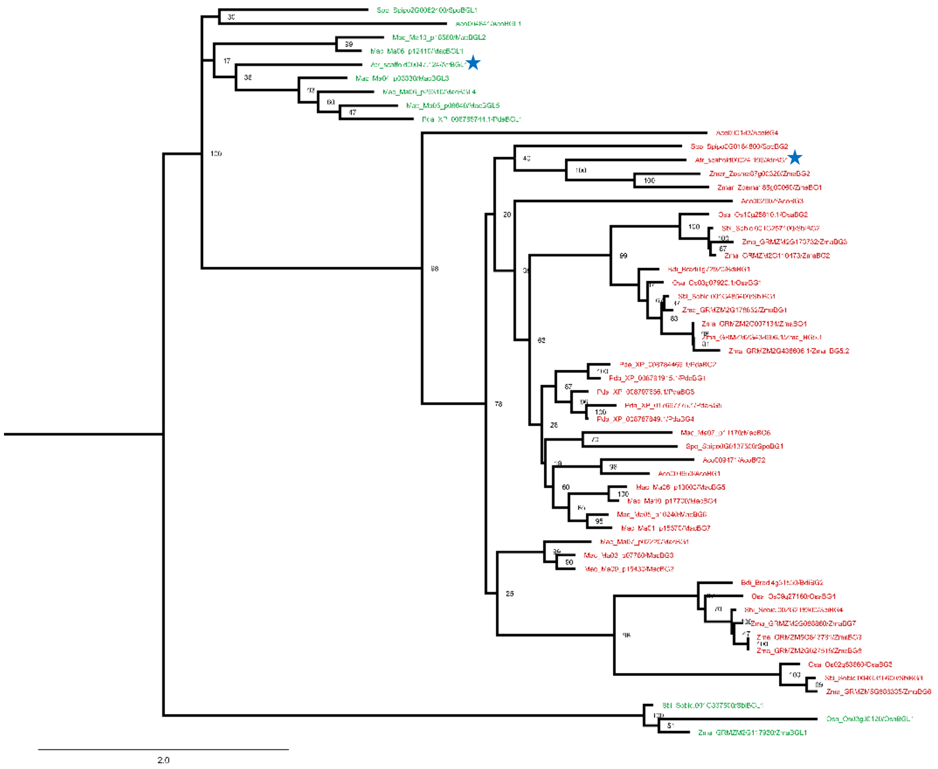

Figure S12. Maximum likelihood-based phylogenetic analysis of BG and BGL proteins from monocots.

PhyML based phylogenetic reconstruction of BG and BGL proteins from monocots and the basal angiosperm *A. trichopoda*. The phylogenetic tree was reconstructed based on JTT+I+G model. The bootstrap values based on 1000 replicates are given adjacent to the branches. AtrBG1 and AtrBGL1 are marked by blue asterisks. The BG and BGL proteins are marked by red and green colour respectively.

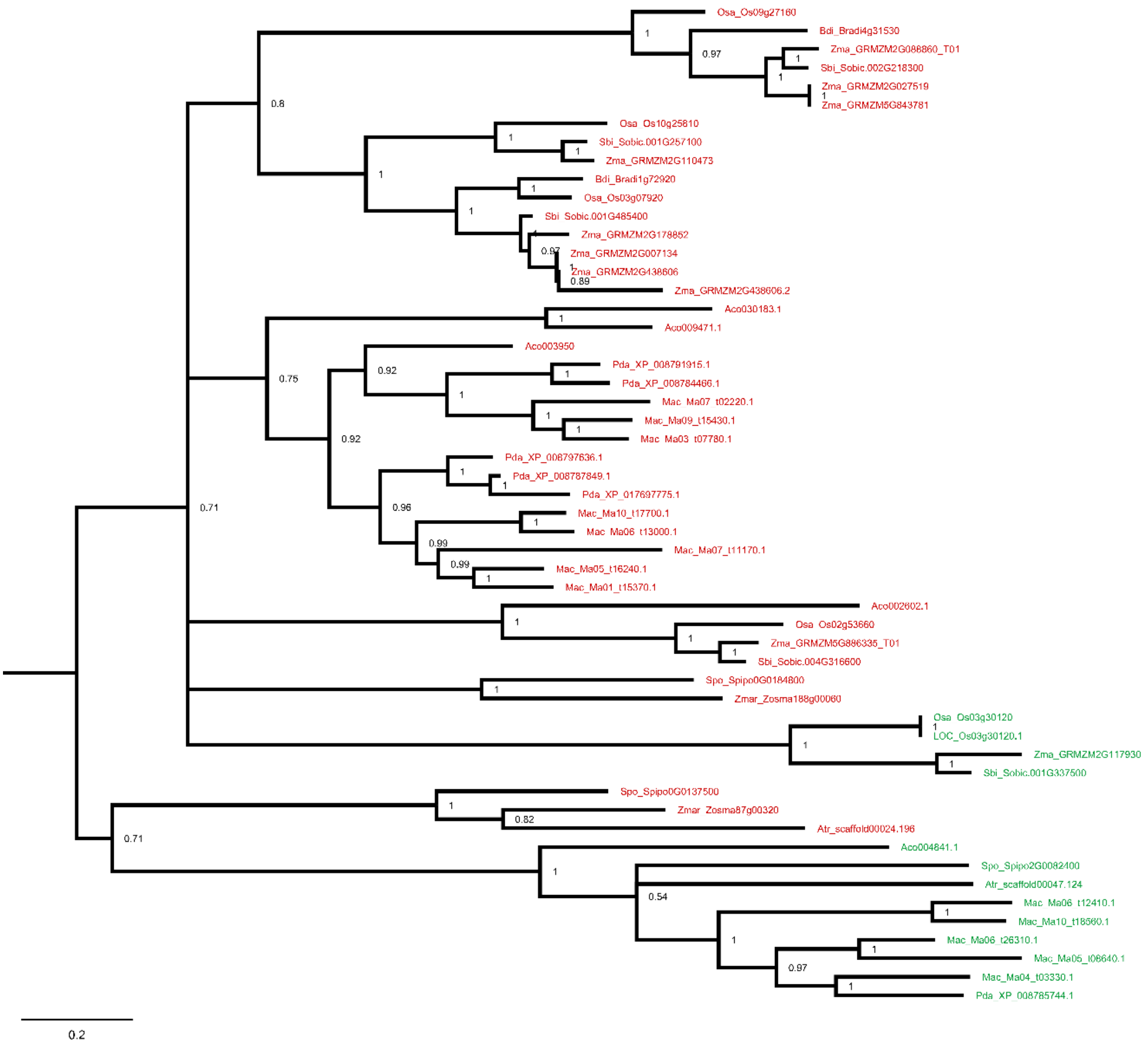

Figure S13. Phylogenetic tree of *BG* and *BGL* genes from monocots inferred from CDS. Bayesian phylogenetic reconstruction of *BG* and *BGL* genes from monocots and the basal angiosperm *A. trichopoda* based on GTR+I+G model. The CDS was used for the phylogram reconstruction. The posteriori probability values are given adjacent to the branches. AtrBG1 and AtrBGL1 are marked by blue asterisks. The BG and BGL proteins are marked by red and green colour respectively.

Table S1. The BG and BGL proteins identified in this study

>Pab_MA_75641g0010
MVIQLSLDLEFKENRDCHRMDNNGPHRHQHHHRSRNQSFSSALLDAICKSTEDEGGMMAYKKSSNRQE
SRNVEAENNTLT
RNSSYKRVVRIERQREDDPWLNNEKPICGSASRIGVFNSEKYEAEAAYSKHRELHLSFQPNISSAPSY
SSDYTSDSTSNC
PYLSSDAESLYRHTSSISTAKTEKNSHHVGRGDKARKWAVGRDCSRDLHGKSSVRNGDFRSTEKPFNP
ADHPAKIKSKSK
TSKDSKKPKQPTSPGRKLANFLNSLFMAGGTKKPKLSLSSSVSESTSYYSSERKPASVCSSSSSVHSR
PCLSKSSRGSKN
SNGVRRSVTFYPTSVTVDEDSRPCGEKCLHRMGKYPTPNPNPCNYPHKHPSMDAYQLPLLSQKLKYRS
LEKDSHAAAAAA
KEIIAKYQKTNSIMKAVVISDLEKQDEEDDEDDDDASCSSSDLFELENLAGIDMNSVYQKELPVYETT
HLKTNKAIAKGL
VV

>Pab_MA_63144g0010
MENCNCHRMDCNSHSGQHHHRHRDHHYHRSRDPSFSSAMLDAIFKSTEDEGGMMAYKNSNSRQETRKV
EVGNNTLMSRQE
TRKVEVGNNTLMRNSSYKRVARIERQWDEDPAWMKINEKSMRGRSLKIGVFKAENYEAEAAYTKHKEF
HLSFQSMSSAAS
CSSNYTSDSTSNCSYLSSDAESLYRNSSSVRTAKTEGNSHHVGRQGRAWKWAVGDRDLHGKSSTRDGY
FRTTEIPFIPAD
HTKIKLKSKSRKDSKKPKHPISPGRKLASFLNSLFLAGGRKKPKLSSSSSVSDSNSYFSSERKPTSTF
ASSSSAQSRSCL
SKSSRGSKNRNGVGRSVTFYPTTVLLNDESRPCGDKCLHRMENPGPSPNPPYYPHVHPSTDAYKLPMV
SKELKLRIREED
SHPAVAKNMIGKYQKADSIMDAVVIRDLDKQDEEEEEDDDDASCSSSDLFELENLAEIDMNVYQRELP
VYETTHLERNRA
IAKGLIV

>Pab_MA_25832g0010
MRNSSYRRVVGIESQWDEDPWLNNEKFMRGRSAKMGVFNSEKNEVGFAYSKLREFHPSLQSNMSSAAL
CSSDYTSDSTSS
CPYLSSDAESLSRHTSSISTAKTGRNSYHLGREDTAGKWAVGGSRALHGKSSVREGDFRSKDKPFIPA
DRDANFKSKSQS
RKDSKKPKKPTSPGRKLANFLNSLFMAGSTKKPKLSSSSSVSDSNSYDSSEKKPASVYSSSSSVQSRP
CLSKTSKGSKSS
TGMSQRSVTFYPASVIVDENSNPCEEKCLHRMEKYPPPDPNPANHLHTYPSTDAYKLPLLSKELRFHM
LEEDWHAAAAKD
IISKYQKANNIMEAVVIRSLDKQDEEEEDDDDDASCSSSDLFELENLAEIDMNMSVYQKELPVYETTH
LETNKAIARGLI
V

>Pab_MA_67269g0010
ALVKKVQLNHPPLCLSAHLVAPHGGGNDRKMEEMTERRYGFWCYKFGAPTFSSALLAAISRSTEDDGG
MMGYEKNRSQVE
TRNVEAENNRLPRNSRFEIQWDEDPWLNNEKSMRGGSANMGLFNAKKYEAEAAYTKSRSELHVSFQSN
MSSATSYSSDYT
SATSSITCPYLSSDAKSLYTHTSSIVTTNKTQRNSHHVVGRGDRACKWTVGGDCSRDLQGKSSVRDGD
FRSTEKPFIPAV

HHAKIKSKSKTGKDSNKPEQPISPGRKLAKFFNSLFMAGFTKKEKLSASFSVSDSNSYDSFERKPSSV
YSSYSSVQSRPC
LSKTSRGSNNHKSVTFYPTTVIVDEDSHPCGEKSLHEKEKYPQITHPSADAYKFPLVRKELKQHIVEE
ERHAAAATTAKE
IIAKYQKTNNTMETVVMRGIDKPEEGEKEDDDSSCSSSELFELENLAPIDMKVDLYQTELPVYETTHF
VTNKAFAKGLIL

>Pab_MA_100548g0010
MKNWDSHRMDYYSNNGQRPHRHHNQHHRSRNQSFSSALLAAICKSTEDEGGMLAYNKNGSRVEMRNVL
EAENNRLTRNSR
VQMQWNEDPWKDKEKFMRRRSSKMGVFNSEKNEGHAASSKHREFRPSFLSNMSSATFCSSDYTSDSTS
SCPYLSSDAESL
YRHTYYNPTERNSHHVRREDRGCKWAIRGECSRDLHGKSLVRDGDFLSTEKPFIPADRHAKKKSKSKS
RKDSKKPRQSSS
PGRKLVNFLNLLFMAAGTKKRKLSSSSSVCESNSYDSSERKQAFVYSSSSSVQSRPCLSKTSRGSKNS
SGMSHKSVTFYP
PSLVVDEDSCPSGKKCLQGKKKYPQITHPVPNSNPANYPHIAHSSADAYKLPLVRKELKQHILVEDEH
VAAAAKEIIPKH
KKVNTMMESVVITSLHRTGEEEEGDDDDASCSSSDLFELENLAAIHMNMNVYQTELPVYETTHFMTNE
AISKRLIL

>Pab_MA_86183g0010
MENWDYNLQNGQHRHRQHHHHSRNQSFSSARFDAICKSSKDEGGMMADKKSRSRVETRNAEAKINRFM
KNSGIERQWEED
PWLNNEKSMRGRSATLGVFNAEKSEAEAAYSKLRDFHVSFQSNLSSTASCSSDYYKSDTSSFSCPYLS
SDSESLYRHASS
IPTAKTERNSHHKNREDRECKWAIGGECSRDLHGKSSGRDGNFRPMEKSFFPAYHYGEIRSKSKTWKE
SNKPKQPNSPGR
KFASFLNSLFMAGGIKKTRLSLSSTVSESNSYDSSERKPTSVYSSSSSVQSRPCLSKPSRGSNNSSGM
SQKAVILYPTNI
IMDEDSRPCGEKWLHRMGKSAPDPNPPNYPDTHPSRDACNLPALIKELELRVLEEDCHAAATAAKEII
GKYQKDHSIMEA
VVRALQKQDEEEEEDNDDASCSSSELFELENLAGIDIDNVYQRELPVYGTTHLETNKAIAEGLIV

>Pab_MA_464588g0010
MAEKWDCHLMDYNLHNGQHLHRHHHPRNQSFSSALLDAICKSTEVEGGMTANKKNRSRLETRNVEANN
NTVTRNSRIGTQ
WHEDSWTNNGKFIHGISAKMGVFNAENIEAEAAYRKRREFHLSNMSSAAFCSSECASNTSSISCPYLS
SDAESTYSVRTA
KTGGKYHHVSREERAWKWAVGDGNLYGKSSVRDGQFRSTEKPFIPADHHAKVKSKSRTGKDSKKPKQP
ISPGRKLANFLN
SLFTPGDTKKPKLSSSSSADTRPYGEKCLYRIENPAPKPNPGIYPNSHPFKVPYNLPLLDKNNHAAAV
AAKDIIGKYQKA
NAIMEAEVRTLGKQEEDQEEDVASCSSSDLFELENLADIDMNLHQKELPVYGTTHWETNKAIAKRLIV

>Pab_MA_183573g0010
MDNWDCPQMDYNSRNGQHRDHNHHRRSRNQSFSSAVLATICKSTEVQGGMMAYKKNGSRTETRNFKAK
INTVVRISRIER
QWDDDPWLNDVKSMRGTSAKMVIFNAEKYEAQAGYISKQREFCLSFQSNMSSAASCSSYYTSGSTSNC
PYLSSDAEYLYT
HSSSIPTAKTERNSHHVGREDRECKWATGGKCSRDLHAKSLFRDFRSTEKPFIPADHQDNIKSKIQKE
SKKPKQPASTGR
KLTNFLNSLFTAGGTKKPKISSSSSVSDSNSYDSSGIKPTSLYSSFSSVQSSPCLSKTSRASNNCSGM
SPKSVTFYPSVI
MDKADFRSCGEKCLRRTEKYPACRTNPSSYLQITHPSTNAYKRPPLSKESKLHILEDSDATAAATTSK
DIIGRFQEVNSI

MEAVVIRDRDKQDEEEEDDDDASCSSADLFELENLAAIDMKPLPVCDPTHMETNTAIAKSLIV

>Pab_MA_9563494g0010
MEIWVGHRIRYNAQNGQHRYRHHHSRSQSFSSALLDAICKSTEDVEETVAYKKSGNRQETRNVEAEDK
TLMRNSNYKSFS
RVERQWDEDHPAWTNKEKLMRVRSTKVGVFNSEKYEAEAAYTKSRSELHRSFQSSMSSAASCSSVYTS
DSTSNCPYLSSD
AESFYKHTSSIPTAKTEKHSDHVGCKDRACKWAVGGEGYRDLHEKSSVRDGAFRAIDKHFIPADHHAK
IKSKSKSCKDSK
KPKHPISPGSKLAGFLNSLFMGGSTKKPKLSSSYSVTDSNSYHSSERKPTTDSSSSSVQSRPCLSKTS
RGSKNVDGVRRC
VTFHPTSVIVDEDSRPCGEKCLHGMENAAPHPNPYNYQQIRHPSTDAHKLPLLSKKLKHRSLEKDSHA
VEAAAKSIITED
QKTNSIIEPAVVRDLDRKGEDGKEDDDGSCSSSDLFELESLEAIDMNVYEKKLPV
>Tca_Thecc1EG029108
MDMWVKLPREDRCRNRRENPSFSSTLLDAIYRSIDESNGSKGEEELIFYRETTMRKKHSNNCSLKEEE
MTSLQRACMIEKWMEKKASCDNKVAIRRKSMADSERNSRNDFDPVLLNSSSSSSDSSCGGGFSSSESD
SFYNAKSRSSSSSSSHYTTHRPKPIRTSVSARPERYERPQHEVENSFHAAAQKPKHEGGFVRTKSKAL
KIYSDLKKVKQPISPGGRLASFLNSLFTAGNAKKAKISSSGYEERKLKSEQTSSTCSSASSFSRSCLS
KTPSSRGKLSSNGTKRSVRFCPVSVILDEDSRPCGHKSIHYENDQTSMIRKPSNKELEFRNLEENRRV
VEAAKDLLKSYQKKKEEYDMRDVRNGNGDSSEDDDEEDAASYASSDLFELDNLSAIGIERYREELPVY
ETTHLDTNRAIANGLIV

>Tca_Thecc1EG037363
MYRLEKTLREERYRHERDNPSFSSTLLDKIYRSIDDGEANHEDLKFYRETMQKKQSKGNMKSSRSRGG
GGGEEMSSFQRACLIEKWMEKKVSEKANAERKQVFSEFERKSHHEHDHDHDVLFFSSTSSSSDSSSGG
FSSSDTESMYGTRTIASCFVPPRPKPVRTSASARSDKPLKAEKTGRSERALFYEQRELHMFDDYHYNS
ASDHTPKLDESLFKSKSRAMKIYGNLKKVKQPISPGGRLASFINSLFTTGNTKKTKSSSSIVSCDDER
KLKSGQVSTCSSASSFSRSCLSKNSPSTRERLRNGVKRTVRFCPVSVIVDEDCRPCGQKCLYEEEDSS
LLSVSVPTAWKIGKTSSRKCEEELKLQIMEKTRRVEEMAREFLKEYHLNQKKNDYIPRDSRSNYVDEM
DEDEDDAASYSSSDLFELDHLVLIGNDRYREELPVYETTHVETNRAIANGLIV*

>Tca_Thecc1EG037572
MYRREGSVRETAVPQRRKTPSFSSSLLDAIYRSIDESANGDEATLCHYRETKTTLVKKQNNAPSEEER
RVSSLRRAIMIEDWVEKQSGYGSAVHFNSTSSSSDSSSGGIFSSSEAESSYKEKSRRSTPAKPEKSKQ
FEQRNFDNNNNNQQRAKREGGGFSKTKLKALKIYGELKKVKQPISPGGRITNFLNSIFNANAKKVKMC
SVGVSDDVSFDRKSKTTCSSASSFSRSCLSKTPSSRGNKYSNGKKRSVRFCPVSVIVDEDCRPCGHKC
IYEDDPSLMPTSTVQKNVKSSSRKEELKNFVKEKESGVSNKARDYLRSYQRRGTGKLDLRGFVDDYED
DDEEEEDDALSYSSSDLFELDHLIGIGRYREELPVYETTSLKTKQAIANGFIL*

>Tca_Thecc1EG011482t1
MSVTGVSDPDRMLKKSFHRRNDSGELDVFEAARYFSGYNEAASYNCATFTQKIMREERQPWRGGRISL
DVPMRNPFPQQTHAVEKQIKEKKYKQPSSPGGRLASFLNSLFNQTGSKKKKSKSTTQSMKDEEESPGG
RRKRRSSISHFRSSSTVDTKSFYSSSSSGFRTPPPYAHTPTKSYKDFRSYSDHKQVVTLSKYSIGQTK
STALANEVLEDKRNTTDYSWLDEKFKFNDGYSEKHKNNLGTRHQEKDRNWADRYPSEEKEFKKFNEVD
DGADSDSSSDLFELQNYDLGIYSSGLPVYETTHMDSIKRGAPISNGAL

>Cpa_evm.TU.supercontig_126.40
MYGWEKSLGENRYRQERRKPSFSSSLLDEIYRSIDDCGSDLQDLKFYPHTMQKKQSKVTTKSSRIPRK
QEEVSGKRRACLVEKWVEEKANEKSIVARQRGQLLKIFERENHYDHDHDHDALLFSSTSISSDSSSGG
FSSSDTDSIYGTRSRGGTKPIRNSVLPRSQKTECTLFYQRREFQLLDDHDPKQEDGFIKSKSRAMKIY
SNLKKVKQPISPGGRLASFINSLFSAGRSAKSSSPSTMMSCDVDRTIHSRQSKSGQISTCSSASSFSR
SCLSKNSPSTREKLRNGVKRSVRFYPVSIIVDEDCRPCGHKSLYKEENDAGVMSLSVPTSWKIGKSPS
RKKEEELKLMVMEKSRKVEEMARELLRDYHHHHHCHHHHRHHHHHHHHHSKKNSLTISSNVGDNYDDD
DAASDSSSDLFELDHLSVIGNNRYDEELPVYETTHMDTNRAIASGLIL*

>Cpa_evm.TU.supercontig_25.102
MADTKHFMNSVLFNSASTSSDSSTAGFSSSESDSFYTTTTTARSTSSSPTYTTPRRPNPIRTTSAAGY
ALPEKSRHVMMENTHKSKSDHKREDHGGFVRTKSKALKIYSDLKKGKQPISPGGRLATFLNSLFTAGN
GKKVSKISSSSATNEEQTPTCSSASSFSRSCLSKTPSSRTKSNSNGTKRSVRFCPVSVIVDEDCRPCG
HKSLYNQTQFMTESNERVDHDQWRVMEENRRVVQAAKDLLKSYRQNKVEFDMRDHVISHQKKVRCYDN
VEEGEEEEDDDDAASCSSSDLFELDNLSAIGIERYSEELPVYETTNLGTNRAIANGLIL*
>Cpa_evm.TU.supercontig_28.87
MHRRRTPSFSSSLLDSIYRSIDHSNTSQSHFGYYSPTKSSAMFNNHQDPDYDSRLHQDLRRAIMIEQW
VEKHNDYGVSAHLNCSSSSSDSSSGGIFSSSEAESCHKTKSRSRCSRQGRSDKCMQRITDNQKQQQKQ
PMRESGNGGFTKTKLKALKIYGELKKVKQPISPGGRITSFLNSIFNSNAKKVKLCSVGAMEDVGFERK
SKSTCSSATSFSRSCLSKSSSSSTRGKHGKSSNGETRRSVRFHPVSVIVDEDCRPCGHKSIYENDPSL
MPTPTVHKTLKEELKAYAMEKEAGVDRVARDYLRKYQKKQEFSLRSQVEHHENEEDEDGGEDALSYSS
SDLFELDHLIGIGRYREELPVYETTNLKTNQAIANGLIL*
>Cpa_evm.model.supercontig_1.160
MSITGLSDSDRLYKKSHHWRNDSGELDVFEAARYFSGYNEGGAGYNACGSYTQKIMREERQPWRPGGG
RISLDVPMRSNPVSHLHQQYSHHHLHHHSSSMEKQIMKEKKYKQPSSPGGRLASFLNSLFNQTGSKKK
KSKSTTQSMKDEEESPGGRRKRRSSISHFRSSSTTDTKSMYSSSSASGFRTPPAYAHTPTKSYKDLRT
LSSDHKQIISLPKNAKSTQNDEASADHDQIKINTDFSWLDEKLKLIDAYTEKQKNVGKNQDRMWEEKE
FRKLNEMMDDGGESDSSSDLFELQNYDLGIYSTGLPVYETTHMDTAVKRPISNAAL
>Ath_AT1G54200
MDPWDTYNIPLDHQHRLRNRRDHRHPSFSSTLLDQIYRSIDDSSTNSSSMRKTKHQNREDTRVSANRR
DDFNRSKNLKTIEPVFFKHSSSSSSDSSGFSSSESDYFYRRSKSSPAISHPKPIRTTVERFERSPQNH
RPNSSNKQEHGSFLKTKSKALKIYSDLKKVKQPISPGGRLATFLNSIFTGAGNTKKLNKINTTVTSTT
AAAAAASSTTTCSSASSFSRSCLSKTPSSSEKSKRSVRFCPVNVIFDEDSSKYNNKNNKVYGNNEREY
ESIRHTLENRVMEENRRVIEAAKELLRSYQKKNKEVIEVSVEDDEEDDDDDALSCTSSDLFELDNLSA
IGIDRYREELPVYETTRLNTNRIISR
>Ath_AT3G13980
MEITWEKPKSSSHHRNPSFSSTLLDQIYRSIDDSSPPPPLESIKKKKHHHQQRNASLHEDREISPIYH
RRSIAADFERSRRKTDFLRHSNSSSSDSSGFSSSESDSFHGRSKSSASPPSSSRQQPKPIRTSSVDHS
SAVQKPKELGGFLRTKSKALKIYSDLKKVKQPISPGGRLATFLNSLFTNAATNPKKHKKTTTVAVVEE
PHSSSTCSSASSFSRSCLSKTPSSSGKSKRSVRFCPVNVILDEDSSFTMPYAYNNERLYDNNEAKRVE
EHRRVIQAAKDLLRTYHNKNKVTTTNINNVEEDDEDDAASCASSDLFELENLSAIGIERYREELPVYE
TTRLDNMNRVIATGLIV
>Ath_AT3G42800
MAFPQRKRTPSFSSSVLDSVYRSIDESDGLQSDLKGSINENVSSSSSSPSPNKKDDKLTTLRRAIMDE
EHWLYARSSTTTTNSSDSSSFSSSEAESYRTKRRLRKLAEQGKRSGDERQRTKRTVMDNDSRLFSKSD
DDKKPKAVKIIEELKRSKQPVSPGARLTSFLNSIFQSNAKKVKLCSVGKTTDVKSSSSKSCFSRTRNK
TDNNNNNCKKLERSIRFYPVRVTIDGDCRDYAQKHITRVRKPIPEFTAKKSVKEEIKTNDHHTEFTCI
TRNIGLKDFVRSNKYEGKEEEEDAWSHSSSDLFELDSYRIGMGRYLKELPVYETTDFKTNQAIARSLL
L
>Ath_AT5G12050
MHKSKRNPSFSSTLLDEIYNSIDPKTQKTQPYVGSVNTTTKKQSIVTRSVPDRKIHRDRFFGSVSSSS
DSNSSIFSSSDTELTHGKKTTSSRPLCFGPSKTKPRKTEDKTLFHQNRATRVYDDYDYASDVPKFNRH
DENWENTRNRRSVKSSGNQKKPKTPASPGGRIVNFLNSLFSNNSKQSNAVKSYPRKTSYDDSAYVRKT
SNDYHSSTTTCSSASSFSRSCMNKGYEKSSGRIKRSVRFSPVNVIVPESFTSKEEDYFSNGNARKSVK
KNVEDGGRRSVEEIAREFLRDYHKNHENSLVKTNGFEDYEDDDEDDDDDDVASDSSSDLFELDLVGNH
HHHNVYGDELPVYETTFAGLIL
>Ath_AT1G69160
MSMKGISSAESDKLSRRISLTHKRNSEELDVFEAAVYFGYNEASSGDHGHTQKYGYNAAREENPRRWG
ILGGGRRISLDLPIRCSEQVYHLQQDHHEKHEVTTIKERLGNVRHKQPSSPGGKIASFLNSLFHQAGS
KKNKSKSKSKTKPTDPEVEEEIPGGGWMRRRRRSSISHFFSSSRSTSTTTTTTASSSSKSLISSSSSG
FRTPPPYLNTPTKNYKQFLNYTSATKQVGEEETKTNKEYSWLDEKLKVMESLSENQRIWSDDEDIDDD
RRIKREGEDDGMESDSSSDLFELQNYELSRGGLPVYETTNVANINKTHI
>Ath_AT1G13670
MSEMAGDSSTSEDKIHKPNRERNVSGELDVFEATRYFSDFNEPTTIEYSRIQIQKQSIVTENRQKRVH
PETEEHLPKPRVVVIKPQKKEMTRGGGKKLTSFLNSLLRSAGLKKKSKSAPEVETPRVERMRRKSCVV

TTHAADASPLTNAGAWSLNARRRSFDEKDLKKSDQKLNIRFCESLYSDQRVETKDPNAGDKNDVIGGC
ESDSSSESDLFELDLFAKSNP

>Cru_Carubv10010185m.g
MSNLETQETTMSEIAGDSPPSEEKINKPNRERNVSGELDVFEATRYFSDFNEPTTIESPRIQIQKQSI
VLENRQKRVHPETEEELSKPRAVVTKPQKKEMTSVGARKLTSFLNSLLRSAGLKKSKSKSTTEVETPR
GERMRRKSCVVTATHVVASSPIPGNGVWRLNARRRSFDEKHVKALKKSDEKLNVRLCESKDRNVGDHE
TDVNGDCDSDSSSESDLFELDLFAKSNP
>Cru_Carubv10001037m.g
MRKRTLEKTLLVLVSSNFIRRRLLLLLSAKKHTKQNRQLQSLFLSLLLSSSLILFLDQIIMHKSKRNP
SFSSTLLDEIYNSIDPKTQKTQSFVGSVNTTTKKQSIVSRSVPDRKIHRDRFFGSVSSSSDSNSSIFS
SSDTELTHGKKVTSSRPLCFGPSKSKPRKTEDKTLYHQNRASRVYDDYDYSSDVPKFTRHDENWENNR
FRRSAKSSGNPKKPKTPASPAGRIVNFINSLFSNNSSKQSNAVKSYPRKTSYDDSAFVRKTSNDYHSS
STTCSSASSFSRSCMNKSYEKSSERIKRSVKFSPVNVIVPEIFTNKEDDYFSNMRKSVKKNVEDGGRR
SVEEIAREFLRDYHKNHESSLVKNSGFEDYEDDEDDDDDDVASDSSSDLFELDLVGNNHHHHNNVYGD
ELPVYETTFAGLIS*
>Cru_Carubv10009449m.g
MDPWNFNEPNLDLHHHYRLRNHRHPSFSSSLLDQIYRSIDDTSSSTDLSSMRKTKHHQSRDDTSRVPS
SSNRRTTADDFIRSKNLKKPVFFKHSSTTTSSSSDSSGGFSSSESESFYRRSKSSPALSHPKPIRTTV
ERSPQAHRPNSTNKQEHGSFLKTKSKALKIYTDLKKVKQPISPGGRLATFLNSIFTGAGNTKKLNKIN
TSTVNNSTAAAPSTTTTCSSASSFSRSCLSKTPSSSEKSKRSVRFCPVNVIFDEDSSNAKYKNNNNSK
LYGNNEREYESSTRSHHHHHQNFDTLEIRVMEENRRIIEAAKELMRTYQKKHDQEDEEEEEDDALSCT
SSDLFELDNLSAIGIDSRYREELPVYETTRLNTTRIVSR*
>Cru_Carubv10016082m.g
METWEKPIPSSHHRRHPSFSSSLLDQIYRSIDDSSPASLDSTRNKKHHHHHHHRHEVSPADHRRSVAA
DFERSMRRSTTATDFLIHSNSSSSDSSGGAFSSSESDSFYGRSKSSASPPPPSSTRQQPKPIRTSTVD
HSSPLQKPKELGGFLRTKSKALKIYSDLKKVKQPISPGGRLATFLNSLFTNAAAANPKKQRKKTTSPV
RVGETVHSSSTTCSSASSFSRSCLSKTPSSSGKSKRSVRFCPVNVILDEDSSTIPMPYTYNNRLYGSN
EDKRDVMEEHRRVIQAAKDLLRTYHNKNKVTTTYDEDIKYIANVEEDEDDDAASCASSDLFELENLSA
IGIERYREELPVYETTRLDNMNRAIATGLTV*
>Cru_Carubv10017637m.g
MAFPQKKRTPSFSSSILDSIYRSIDESDGLQSDLRGGTINSNNDENVSSSSSTSSPSPTKKDDKLTTL
RRAIMDEEHWLYPRSSTTTTNTSDSSAFSSSEAESYRTKRRSSKFVEQVNKSGDDKKVKAVKMHEELK
RSKQPLSPGARLTSFLNSIFQSNAKKVKLCSVGKTTDVKSSSPRSCFSRTKHKTNSNNNCCKKLERSI
RFYPVRVTIDGDCRDYTQKPISERTAKKSIERVVTSLKEDIKTKVHQTELTCVTRKIGLKDYVTSNRD
EGNEDEDDVWSYSSSDLFELDSYRIGMGRYLKELPVYETTDFKTNQAIARGLLL*
>Cru_Carubv10020681m.g
MSMKGISSAEPDKLSRRISLTHKRNSGELDVFEAAVYFSGYNEASSGDHRNTQKYGYNAAREENRRKW
GILGGGRRISLDLPIRCSEQVHHLHQDHHEVTTIKDRLSNVRHKQPSSPGGKIASFLNSLFHQAGSKK
NKSKSKSKPTESEVEEEVPGGVSWMKRRRRSSISHFLSSSRSNSTTTTTSSSSKSLISSSSSGFRTPP
PYLNTPTKNYKQFLNYTSATKQVVEEDKKTNREFSWLDEKLKVMESLSENQRNWADDEDDDRRIKREG
YDDGMESDSSSDLFELQNYELSRGGLPVYETTNVANINNTHI*
>Brara.A03221
METWEKPSSRGHHRNPSFSSTLLDHIYRSIDDNSPPLEPTRKKKLLHEDLDTSPDKLVFHRRSIAADF
ERSRRTTATTTTAAADSVFLRYSNSTSSDSSGLSSSESDSFYGRSKSSASPPQPKPIRTSAVSSGERP
TTKQELGGFLRTKSKALKIYTDLKKAKQPISPGGRLATFLNSLFTNASTNPKKPKKNTTSISVLSETQ
SSSTTCSSASSFSRSCLSKTPSSSGKSKRSVRFCPVNVILDEDSSVYIPCGNDTKRYRQVMEEENRRV
IEAAKDLIRTYQKNKDLLAVTTCHDVEDDDDAASCASSDLFELENLSAIGIERYQEELPVYETTRLDN
TNRAVATSLTV*
>Brara.B00441
MHRSKTQSRNPSFSSTLLDEIYHSIDPKTQKTQPFLNSLNTAKKQSISVNRGRDRLFGSMSSSSDSNS
SIFSSSDTELTHPKKTTSSRPLCFGPSKTTKPRKTEDKALFHQNRAKTEDKALFRQNRATRVSDDYDY
ASDVPKITRYDEDWENNRTRRYTSKIKTPASPGVRIVNFINSLFSNNGSSKQPSSAVKSYPRKTSYDV
TKPTCSSASSFSRSCLNKTSEKPSDRIKRSVRFSPVNVIVTAAEEEEDYLRKSVKKNVEDRGRRSVEE
IAREFLRDYHKNHENGLVKKNDFEDYEDDDDDVASDSSSDLFELDLAGSHHHHNLYGDELPVYETTFA
GLIL*

>Brara.B01838
MSMKGVSSAEPDKISKKISFHKRNDAHSGELDVFEAAMYFSGYNEFSSCDHRHSHKHGYNAAREENRR
RWGILGGGRRISLDLPIRCSDQQVHHLHQDHLEKPEVKAIKERTSNVRYKQPSSPGGKIVSFLNSLFH
QVGSKKNKSKSKPTEREAEEEIHGGGWMRRRRRSSISHFLSSSRSNLTTTTTSSSSKSLLSSSSSGFR
TPPPYSNTPTKNYKQFLNFTSATNKVEVLEEKKINKELPWLDEKLNVMESLSEKKKVWGDHDDDADGD
RRIKREGDDDDGMESDSSSDLFELQNFELSRGGLPVYETTNVANINNTPL*
>Brara.C00499
MHRSKPQSRNPSFSSTLLDEIYNSIDPKTKKTQPFVGSVKKQSISVTRSVPDRKLHQDRFFGSVSSSS
DSNSSIFSSSDTELSHGTKKITSSRPLCFGPSKTKQSKTEDKALFHQNRATRVFHDYDYASSNQKKLK
TPSSPGVRIVNFINALFSKQPTAVKSYPRNTSYDDSAFSRKRTDYYYPSTTCSSASSFSRSCLNKRSE
KSSGRTKPRVRFSPVNVIVPEIEEEDYLSSGYVRKSVKKNVEDGGRRSVEEIAREFLRDYHKKHENSL
VKNNNDLEDYEDEDDDVASDSSSDLFELDLVGTHHHNLYEDELPVYETTFAGLIL*
>Brara.E02722
METWEKPRVSSSRDHYRQPSFSSTLLDQIYRSIDDSPPLESTRKKKHPHRNITSLHEEHASDKLVFHR
RSIAADFERSRRTTATSISSVFLTYSNSSSSDSSGLSSSELDSFYLRSKSSPPHTRQPKPIRTSVVEK
PSSKQEHGGFLRTKSKALKIYTDLKKVKQPLSPGGRLATFLNSLFTNASSNHKKLKKINTTVSSSAEQ
PPQSSSNTTCSSTSSFSRSCLSKTPSSSGKSKRSVRFCPVNVILDEDSSIHIPYGYSNKLHGNNVDRH
VMEEENRRVIEAAKDLIRTYQKNKDHLAVTTCDNVQEDDEDDIDDDAASYASSDLFELENLSAIGIER
YREELPVYETTRLDNTNRAIATSLIV*
>Brara.F00058
MDPWDNNTTDHQYRRRDHRHPSFSSSLLDQIYRSTDDSSDVSMRKKQNRAASLDENRVCLEKILLNRR
KTADDFAVNRRKTTEINTVEPVFFKHSSSSSSDSSGFSSSESDSFYKRTRSSRSPPVIHHHPKPIRTA
VERLERPNNKVKSKALKMYSDLKKVKQPISPGGRLATFLNSLFTGNTKKPNKTVSTATSSHTTCSSAS
SFSRSCLSKTTSSSEKTKRSVRFCPVNVIFDEDSKQRESIRHHQSRVMEENRRVIEAAKELIRTYREN
KDVEEEDDDDDDDAASCASSDLFELDNLSSIGIERYREELPVYETTRLNTNRIISR*
>Brara.G02851
MSMKGISSAEPDKVSKRMSSHKRTDAYSGELDVFEAAVYFSGYNDFSSGDHRHTQKYSYNAAREENRR
RWGILGGGRRRSLDLPMRYSEQVHHLHQDHPEKQDVTIIKERFGNVRHKQPSSPGGRIASFLNSLFHQ
AGSKKNKSKAKPTDREVEDEIPGGGWMRRRRRSSISHLLGSSKSNSTATTTSSSSKSLFSSSSSGFRT
PPPYLNTPTKNYKQFLNFTSTTNQVEVLEEKKINKELSWLDEKLRVMENLSEKDKFWADHDGDIDDDD
DDEARRIKSKGKDDDGMESDSSSDLFELQNYELSRGGLPVYETTNVANINNTHL*
>Brara.H00090
MDPWDKTNSLDHHHHRRQDHRHPSFSSTLLDQIYNSIDSSSAVNRRAVAGDSVRSRNLKTAEPVFFKH
WSSSSSSDSSGFSSSESDSFYRRSRSSRSPPEICHPKPIRTTVERLERPNNNNNNNNNKVKSKALKM
YSDLKRVKQPISPGGRLATFINSIFTGNTKKPNKTATSSSTTCSSASSFSKSCLSKTPSSSEKSKRSV
RFCESTRQRQNFDTLESRVMEENRRVIEAAKELIRTYQKNKDVVNIIGKEEEDDEEDDDDGASCASSD
LFELDHLSVIGIDSYREELPVYETTRFHTNRIISR*
>Brara.J02180
MHRSRNPSFSSTLLDEIYNSIDHKTHKTQSFTGFVNTTNNKQSISVTRPVPDRKIHKDRFFSSVSSSS
DSNSSFFSSSDTEPSHPKKKSSSRPLCFGPSKTTKPRKTEDKTLFHQNRAFRVSEDYDYEKNEHTRTR
RSSSNQKKPKTPASPGLRIVNFINSLFSSNNSSKGHPKKMSCDDSAFVRKPNDYYYPSTTCSSASSFS
RSCLNKSSEKSSDRVKRSVRFSPVNVIVPESRSYVEEEDYLSNNGYVRKSVKKNVEDRGRRSVEEIAR
EFLRDYHKKHDNGLVKINAFEDYEDDDDDVASDSSSDLFELDLVGTHHHNLYGDELPVYETTFAGLIL
*

>Ptr_Potri.001G170500
MDRWEKPLRDDRYRHQRQNPSFSSTLLDVIYRSIDESGNGKGEEEQLIFYRETMRKKHEINHGFKGEE
MTSLQRACMIENSSSSESSCGGVFSSSESESIYGVNSSRSSTTSYTMQRPKPVRTSISARPEKYQRRE
DLHQTDTFQHHERNYAPNQKAKPEGSFVKTKSKALKIYGDLKKVKQPISPGRRLASFLNSLFTTGNAK
KAKITTPGGSYEERKLKSEQASTCSSASSFSRSCLSKTPSSRGGKLSSNNGAKRSVRFYPVSVIVDED
CRPCGHKNLYGSDRQEMSKLKLHVMNENRRIEEVARDLLKNYQKKKEEHEEEEEESDDDDDIASCASS
DLFELDNLSVVGIERYREELPVYETTHLGTNRAIATGLFL
>Ptr_Potri.002G223400
MYKKERSSRESTFHPRRRTPSFSSTLLDSIYRSIDESNGEEQHVLGIKKQSCNSVSTTRRDTSSDSSS
AGGGGSGGGVFSSSENESSVRGNSSSCQQRTKPLSDKPHQKPKCEGGGFHKTKLRALKIYGELKKVKQ
PISPGGRIASFLNSIFNSASAAKKVKMCSIGAMDDVSFERKSKSACSSATSFSRSCLSKTPPPRGKPS

NGTKRSVRFYPVGVIVDEDSRPCGHKSIYEDDPGLMPTPRKVVKSSSVKELEVAKGAAADYLRSYHQR
KNVSEFDFRGFHNYVADDSDSDDESCTSSDLFELDHLIGIGRYREELPVYETTNFKTNQAIANGFFP
>Ptr_Potri.003G063400
MDRWEKPLRDDRYRHQRENPSFSSTLLDAIYRSIDENGSGKGEEEQLIYFRETMRKKHENNGIKDGEM
TSLQRACMIDKWMEKKVSHEKVAVRRKSMADFENKSRKDVDSVLLNSSSTSSESSCGGGFSSSESESI
YGVNSSRSSTTSYTMERPKPIRTSISARPEKYQRREDHHQIDMYHNHERNYAPNQKAKHEGSFVKTKS
KALKIYGDLKKVKQPISPGGRLASFLNSLFTAGNAKKAKISTSGGRYEERKLKSEQASTCSSASSFSR
SCLSKTPSSRGGKLSSNNGAKRSVRFYPVSVIVDEDCRPCGHKSLYGGDCQELSSTLVATTVTADARN
NVPTSGEELKFHVTNENRRIEEVARNLLKNYQRKKEEQFDHMSTDLCNDNNHEVMSSDEEEEEESDVA
SCASSDLFELDNLSAIGIERYREELPVYETTHLGTNRAIANGLIL
>Ptr_Potri.006G144700
MYKKERSSRESTFHPRRRTPSFSSTLLDSIYRSIDESNGEEQHVLGIKKQSCNSVSTTRRDTSSDSSS
AGGGGSGGGVFSSSENESSVRGNSSSCQQRTKPLSDKPHQKPKCEGGGFHKTKLRALKIYGELKKVKQ
PISPGGRIASFLNSIFNSASAAKKVKMCSIGAMDDVSFERKSKSACSSATSFSRSCLSKTPPPRGKPS
NGTKRSVRFYPVGVIVDEDSRPCGHKSIYEDDPGLMPTPRKVVKSSSVKELEVAKGAAADYLRSYHQR
KNVSEFDFRGFHNYVADDSDSDDESCTSSDLFELDHLIGIGRYREELPVYETTNFKTNQAIANGFFP
>Ptr_Potri.006G222700
MYRREKTMREERYKHGIKNPSFSSSLLDEIYRSIDDGEPKREELKFYRETMPKKQNKIGRNIGGEAGM
PTLQRACLIEKWMEQKVTQKVITQQRRQNSTELERKAQLDHDLDQDVLFFSSTSTSSDSSSGGFSSSD
TESMYGARSRASSFNPPRPKPVRTSLSARSGKTEKTERTLFHEQRELRMFDDYHYSSASEQTPRLEDN
IIKSKSRALKIYSNLKKVKQPISPGGKLANFLNSLFTTGNSKKSKNSSSIGNFDEERKLNSGQASTCS
SASSFSRSCLSKHSPSTREKLRNGVKRSVRFYPVSVIVDEDCRPVGHKSLYEEEESSLMSVSLPTAWK
IGKSPSRKTDDELKYQVMEKSRRVEEVAREFLKDHRQNQKKNDVTMIDVRGKYNDRYHDEDEDEDDDA
ASYSSSDLFELDHLAVIGNDRRYCEELPVYETTHLDTNRAIANGLIV
>Ptr_Potri.018G049900
MHRWEKTMEERYNKHDMKNPSFSSSLLDEIYRSFDDGEPKHEELKFYRETMPRKQNKGTSGIKIEREE
GMSALQRACLIEKWMEQKVSQKLITQQRRQKLIEFERKSQLDHDLDQDVLFFSSTSTSSDSSSGGFSS
SDTESMYGGFSRASSFNPPRPMPVRTSASARSGKTERTLFYEQREAHMFDDYHYNSASEQTPRFEESL
IKPESRALKTYRNLKKVKQPISPGSKLANFLNSLFTTGNTKKSKNSPTNGNFDEERELKSGQASTCSS
ASSFSRSCLSKHSPSTREKLRNGVKRSVRFYPVSVIVDEDCRPVGHKSLYDVEESSLKSVSLSTAWKI
GKSPSRKIDDELKYQVVEKSRRIEEVARVFLKDYHQNKKKNDVIKIGAGGKYNDRFEDEDEDEDEDDD
AASYSSSDLFELDHLAVIGKDRRYSEELPVYETTHLDTNRAIANGLMV
>Ptr_Potri.018G065000
MYKKERSSRETTFPQRRRTPSFSSTLLDSIYRSIDDSNGEEHVLGRYSRETTMIKKQGCNSVSTTRRD
ASFLEGEKQASPTLRRAVMIESWMEKRSSRGSKHYNATSSSSDSSSAGGGGGGGGGGDVFSSSENESS
VKENSSFAQQRTKPLSDTPQLKPRCEGGGFSKTKLRALKIYGELKKVKQPISPGGRIASFLNSIFNSA
SAAKKVKMCSIGAMEDVSFERKSKSACSSVTSFSRSCLSKTPPPRGKPSNGTKRSVRFYPVSVIVDED
SRPCGHKCIYEDDPGLMPVPQKAVKSTSVRELEVARGAAAGYWRRSCHQKKSVSEFDFRGFHSYVEGD
GDSNSDDDESCSSSDLFELDHLIGIGRYREELPVYETTNFKTNQSIANGFIL
>Ptr_Potri.008G098200
MSITGLSSDTGKPYKKSLHRRNDSDELDVFEAARYFSGYNEAGAGYNGAVYTQKVMREDHKHSWRGGR
VSLDVPMRNPLPHHLHQHSHTVEKQILKEKKYKQPSSPGGRLASFLNSLFNQTSSKKKKSKSTTQSMK
DDDESPGGRRKRRSSISHFRSSGTTDTKSLYSSSSSGFMTPPPYTHTPTKGYKELRSCSDHRQIVSLP
KQNGIVKSIAFRNEILDDKKNTDLSWLEEKYKFNDGFSDQKVPRNRGNQHLEKDRTWVDQYPSEEKEC
RKFDEVDDGTESDSSSDLFELQNYDLAGTYSNGLPVYETTRMDSIKRGAVPISNGTL*
>Ptr_Potri.010G154200
MSITGLSSDPSKPYKKSLHRRNNSDELDVFEAARYFSGYNEAGAGYNGATYTQRVMREDHKNSWRGGR
MSLDVPMRNPLPHHIHQQPHTVEKQILKEKKYKQPSSPGGRLASFLNSLFNQTSSKKKKSKSTTQSMK
DDDESPGGRRKRRSSISHFRSSSTTDTKSLYSSSSSGFRTPSPYTHPPTKGCKESRSYSDHKQIVSLS
KQNGTVKSTTFQNEISDDKKKSGFSWLEERYKFVNDGFSDQKAAKNRGNQYLEKDRTWVDEHYRSEEN
EFRKFNEVDDGAESDSSSDLFELQNCDLGIYSNGLPVYETTHMDSIKRGAVPISNGTL*
>Mes_Manes.01G025900
MSITGLSETSKLYKKSFHRRNDSDELDVFEASRYFSGYNEAVGYSGATYTQRVMREDHRYPWRGGRMS
LDVPMRNPLPQQSHTVEKQILKEKKYKQPRSPGGRLASFLNSLFNQTSSKKKKSKSATQSMKDEDESP
GGRRKRRSSISHFRTSSTADTKSLYSSSSSGFRTPPPYAHTPIKSYKDFRSYSDHKQVLSLSKHNGNV

KSTVLQNEVLDDKRNTDLSWLDEKFKCSDTFAEKPKSLGHRYLEKDRIWVDQYQPEDKIFRKFDEVDD
GAESDSSSDLFELQNYDLGIYSSGLPVYETTNMDSIKRGAPISNGTL*
>Mes_Manes.03G038000
MSLREMENTFSQRRRTPSFSSSLLDAICRSFDEPNCREEEIFSQCRKTTVIKKQSIVNSVSTTRRDGF
VEEDKEIFDLRRAIMIESWMEKQSTRGSVHCNSTSASSDSSSATGRNGGVFSPSEAESSVKDLTPQRN
KPISEKQQKPKCEGGFTKTKLQALRIYGELKKVKQPISPGGRIASFLNSIFRSGTAKKAKMCSIGDMG
DASYQRKSKSACSSVTSFSNSCFNKTPPSREKQSDGNKRSVRFYPVSVIVDEDSKPCGHKCIYEDDPG
LMPMPVPKISRGSSVKQDAAKGGSYIRNYQKKNISEFDFRGFHSYVEDSNDDDSSCSSSDLFELDHLI
GIGRYIEELPVYETTSLKTNQAIANGFSL*
>Mes_Manes.03G052400
MHRWERAIREDRYKLESKNPSFSSTLLDEIYRSTCEADTNHEDLKFYGETMMPMKHTRGSSVKVSRAV
EENKEMEALRRACLIEKWMDSKVTQKVSTQHSRKKLTEFERKLQLEHDLDQDAVFFSSTSISSDSSFG
GFSSSDTESCYGARSMASSSFFPTRPKPVRTSVSTRSGKTEKTERKVSTLFHEQTPRVEENIIKSKSR
ALKIYDNLKKVKQPTSPGGKLANFIHSLFTNGNTKKARGSSSVSNCDEAWKSKPRQAPSTCSSVSSFS
RSCLSKSSPSTREKLRNGVKRSVRFYPVSVIVDEDCRPCGHKSLYKEEQSSSFMSVSFPKSWKIGKSP
TRKVDDELKYQVIEKTRKVGEVAREFLKDYRQNQKKNDDLIMRNDFCHYNDQFGDDDDEDEDDEDDSS
CSSSDLFELDHLSVIGKNKYCEELPVYETTRVNTNRAIANSLIM*
>Mes_Manes.04G143200
MDNWEKPVREDRFRHKHRQNPSFSSTLLDAIYRSIDESNDKGEEELIFYKETMRKKHSNGFKGDERIA
TLQKACMIEKWMEEKVSYEKVAVRRKSVGDFDKNAPKDFDPKPPPMLLNSSSSSSESSCGGGFSSSES
ESTYGLSSSRSSSICAMQRPKPIRTSVSARPERYERCVDEISMYHHERKYSNYAPTQKPKHEGSFGKT
KSRALKFYGDLKKVKQPISPGGRLASFLNSLFTAGNAKKAKISSSLSGCEERKIKSEQTSKCSSTSSF
SRSCLSKTSSARGNKLSNGTKRSVRFYPVSVIVDEDSRPCGHKSLYANHEETSMAVTSTRNLANEELK
LHVMNESRRVEKVARDLLKNYQKKQEEFAARELFCNGNGESSAEEEEEDDDDEEVASCASSDLFELDN
LSAIGIERYREELPVYETTHLDKNRVIANGIIL*
>Mes_Manes.05G112100
MSITGLPEKSKLYKKSFHRRNDSDELDVFEASRYFSGYNEALSYNGANYAEKVMREDHRHLWKGGRMS
LDLPMRNPLPQQSYTVEKQISKEKKYKQPSSPGGRLASFLNSLFNQTSSKKKKSKSATQLMKDEDESP
SGRRKRRSSISNFRSSSTTDGKSFYSSSSSGFRTPPPYADTPTKGYKDFRSCSDHKQVSLSKHNGNTK
STAFQNEVLDEKRNTDLSWMDEKLKYDDGFSEKTKSLGHQYLEKDRIWIDQYPPEEKEFKKFNEVDDG
AESDSSSDLFELQNYDLGIYSSGLPVYETTHMDSIKRGAPISNSTL*
>Mes_Manes.11G021800
MDKWEKPVRAEDRFRHHHRENPSFSSTLLDAIYRSIDESNGKVEEELIFAMRKKHSNGLQKACMIEKW
MEEKVSYEKVAVGRKSMDDFDKNTRKDFSRKPPVLLNSSSSSSESSCGGGFSSSESESIYGLSSSRPS
STYAMQKPKPIRTSVSARPERYERAVDEIAVYQYNHHRDTNYAPTQKPKHEGSFVKTKSKALKIYGDL
KKVKQPISPGGRLASFLNSLFTAGNAKKAKISSSSVYEESKIKSEQTSTCSSASSFSRSCLSKTSSSR
GNKLSNGTKRSVRFYPVSVIVDEDSRPCGHKSLYGNHEETLMAVTATRNLTNEELKFHVMNESRRVEE
VARDLLKNYQKKKQEELAVRDLCTGNSELLEEDDDDDEDDAASCSSSDLFELDNLSAIGIERYREELP
VYETTHLDTNLAIANGLIL*
>Mes_Manes.16G084100
MHRWEKAMRDDRYKHEGKNPSFSSSLLDEIYRSTCEGDTNHEDLKFYGETIMPKKHSKVSSIKSGRAI
DEDKEVEALRRACLIEKWMDHKVSQKVSRQHSRKNLTEFERKSQLEHDIDQDVLFFSSTSSSSDSSSG
LFSSSDTESFYGARSRVSSSAAPRPKPVRTSVSALSEKIEKTEKKGRSLFYEQRELHMFGDYHYSSAT
EQTPRVEESIIKSKSRALKIYNNLKKVKQPISPGGKLANFINSLFTTGNTKKSKNSSSVSNCDEDWKS
KPAQTTSTCSSASSFSRSCLSKNSPSTREKLRNGVKRSVRFYPVSVIVDEDCRPCGHKSLYEEEESSS
LMSVSLPTAWKIGKSPSRKVDDELKYQAKEKSKRVEEVAREFLKDYHQNQKKNDDLIMRKAGCKYNDH
FEDDEEEDDDDDLSCSSSDLFELDHLSVIGKDRYCQELPVYETTHVDTNRAIANGLIM*
>Mes_Manes.16G097700
MYKKDRSSREMENTFPQRRRTPSFSSSLLDAIYRSIDEPNGGEEEFFSQYRETKMIKKQSTAKSVSTA
ARRDAFLEADLRRDIMIESWMEKQSTRGSLHSNSTSSSSDSSSGAGGSGGGVFSSSEAESSVKENSTR
VSILTPQRINPLSEKQQKPKCEGGFTKTKLRALKIYGELKKVKQPISPGGRIASFLNSIFNSGSGKKV
KLCSIGAVDDVSSERKSKSACSSVTSFSRSCLSKTPPSRGKQSNRSKRSVRFYPVTVIVDEDSRPCGH
KCIYEDDPGLMPMPIPQKIAKSSSLKGDVVKGANYIRKYQKKNISEFDFRGFHSYVENHEDSDDEEED
DDESCSSSDLFELDHLIGIGRYREELPVYETTSLKTNQAIANGLML*

>Rco_29706.t000031
MDHHQRENPSFSSTLLDAIYRSIDESNGKGEEELILYRETMRKKHSSNGFKDGAAVKEERKTSLKKAC
MIEKWMEEKVSYEKVAIRRKSMADFDKINTRKDFTHRPVLLNSSSTSSESSSGGGFSSSESESVYGLS
SRSSSTNYTMQRPKPIRTSASARPVDELGMYNHHHPTTQKPNNKHHEGSFVKTKSKALKIYGDLKKVK
QPISPGGRLASFLNSLFTAGNAKKAKISSPSGYEERTKFKSEQTSTCSSASSFSRSCLSKTPSSRGNG
TKRSVRFYPVSVIVGEDSRPCGHKTLYGTDQENPSLMTITTTRVPTNEELKFHVMNESRRVEEVARDL
LRNFQKKKQEEFDVSNGNVPRYQELEEDDDNEEEEDDDDAASYASSDLFELDNLSAIGIERYREELPV
YETTHLDTNRAIANGLLL
>Rco_29739.t000035
MHKCEKEMREDHRHKYEGKNPSFSSSLLDEIYRSICEGDTKHDDLKFYRETMPKKQNKDTRAIRGEKE
ADEVMASLRRACLIEKWMEQKVSQKVIGTQHKKQNSTVFERKSQHDHDIDQDVLFFSSTSSSSDSSFG
GFSSSDTESIYGARSGGYSFAPARPKPVRTSVSARSGKTERTLFYEQRELHMFDDYHCSSAFSEQNTP
RLEENIIKSKSRALKIYNNLKKVKQPISPGGKLANFINSLFTTGNTKKSKNSSASSIGNFVDERKFKS
AQASSTCSSASSFSRSCLSKNSPSTREKLRNGVKRNVTFYPVSVIVDEDCRPCGHKSLYEEEEEEEEE
SSAVMSVSLPRAWKIGKSPSRKVDDELKYQVIEKSRRAEDVAREFLKDYHQSQKKMNNDDVIMRARDV
RRNYNDHFEDEDEDNDNDNDDASCSSSDLFELDHLSVIGKDRYCQELPVYETTHVSTNRAIANGLIM
>Rco_29912.t000009
MYKKESSSSRENPLPQRKRTPSFSSSLLDAIYRSIDESNGGAGCGEEEVLSQQYQETTVIKKQSTRTQ
SVSTIRRDTCLEQEKDLSTLRRAILLESWMEKQTHSNSTSTSSESSSGVGGGRGVFSSSSETESSVEN
SRRTTTQQRSKQVSDKQQKPKCEGGFSRTKLKAMKIYGELKKVKQPISPGGRIASFLNSIFSPGSAKK
VKMCSIGAMDDVSTATDRKSKSACSSVTSFSRSCLSKTPPSRGKASNGNKSKRSVRFCPVSVIVDEDS
RPCGHKCIYEDDPGLMPTPVPPKLVKSSSFKEDAGKGAKYIRNYQKKNISEFDFRGFHSYIQDRDAVD
DEDSDEDEDNQSCSSSDLFELDHLMGIGRYREELPVYETTNFKRNQAIANGFIL
>Rco_30174.t000478
MSITGLPDTSKLYKKSLHRRNDSDELDVFEAARYFSGYNEAAGYNGGTYTQKILRDDYRHPWRGGRMS
LDVPMRNPLPQQTHSHHHTVEKQILKEKKYKQPSSPGGRLASFLNSLFNQTSSKKKKSKSATQSTKDD
DESPGGRRKRRSSISHFRSTSTADTKSLYSSSSSGFRTPPPYANTPTKSYKDLRSYSDHKQVISLSMQ
NGNVKSTGLQNEVLDEKKKTDLSWLDEKFKISDALSEKTKNLGNHRYLEKDRIWVDQYPSEEKGFRKF
DEVDDGADSDSSSDLFELQNYDLGIYSSGLPVYETTNMDSIKKGAPISNGTL

>Mdo_MDP0000125138
MYVRERLPKEETFLRRRRNPSFSSSLLDSIYRSIDESSGGDGDQGYVRESTAMVRKQSSSTKGDNEKV
NLRRAIMIENWVEKQSVHSSMFSNSASSSSESSSGAAFSSSETDSSYRSRTKPKAVEQRFVQFEEKEK
IESGGGSAFSRTKLRALKIYEELKKVKQPISPGGRIVSFINSIFNSGNVKKPKMCYVGAVEDVTISEN
VSNSKSACPSSSASASTFSRSCLSKPSSRAKKSSNGTKRSVRFYPVSVILGEDSQPPNHLKCVFEEDP
SLMPKPSFQKYARACPGNYDKLIQSGKXRTEDLLTFNRSTKSTSYRTTGAVSQNLVRSFCDNADDEES
DHDAESCSSSDLFELNHPVGVGRYMEELPVYETTNFRTNQAIAQGXL
>Mdo_MDP0000127468
MYVRERLPKEETFLRRRRNPSFSSSLLDSIYRSIDESSGGDGDQGYVRESTAMVRKQSSSTKGDNEKV
NLRRAIMIENWVEKQSVHSSMFSNSASSSSESSSGAAFSSSETDSSYRSRTKPKAVEQRFVQFEEKEK
IESGGGSAFSRTKLRALKIYEELKKVKQPISPGGRIVSFINSIFNSGNVKKPKMCYVGAVEDVTISEN
VSNSKSACPSSSASASTFSRSCLSKPSSRAKKSSNGTKRSVRFYPVSVILGEDSQPPNHLKCVFEEDP
SLMPKPSFQKYARACPGNYDKLIQSGKXRTEDLLTFNRSTKSTSYRTTGAVSQNLVRSFCDNADDEES
DHDAESCSSSDLFELNHPVGVGRYMEELPVYETTNFRTNQAIAQGXL

>Mdo_MDP0000135865
MERWDIKSLPKNREASSNRQSRNREASSHRQSRDNPSFSSSLLDSIYRSIDETTAGSEGVASRSEQHH
RLIFYKETMKKKQSTAAAVTTSGRHGHARSKEDQQHEIMNFRRACLVEKWMEKEAEKSVPVRRNSITD
FETKKSRYSHQNEFLTNSSSSSSDSSGGFSSSESDSMYGSKSRSSSSCYSMHRPKPIRTSISSEKVLF
DDHQRNHHHNSQKKKHENGFMKTKSKALKIYGDLKKVKQPISPGGRLASFLNSLFNAGHVKKSKIDDL
GAERKSSSKSGQMGYSNPSSTCSSASSFSRSCLRKAPLSRSELSGISSNVDAAKRSVRFCPVSVIVDE
DCRPCGQKTLLKEESDLMAVARAAAAIKPPTNEDVGVECCVMDVDEDDDHRRRIEEVARDYLMKNYQK
KHDEDDEDDDDAESYASSDLFELDTAGVEERYREELPVYETTFFDRNRAIANGLIL
>Mdo_MDP0000276736
MYVRERSPREETFSRRRRNPSFSSSLLDSIYRSIDESSGGDGDQGYVRDSSTMVKKQSCSAKGGKEKV
NLRRAIMIEKWVEKQNVHSSMFSNSASSSSESSSGAAFSSSETDSSCRSRTKPKAVEGRFVQFEEKEK

SESGGGGGFSKTKLRALKIYGELKKVKQPISPGGRIASFINSIFNSGNVKKAKMCHVGAVEDVSITEH
VSNSKSSCSSSSAPASTFSRSCLSKPSSRAKKSSNGTKRTVRFYPTSMVLGEDSQLSXHHKCVFEEDP
SLMXKPSFQKYARSCPGNYDTLIQSGXSRTQDLVTFNCTTKSTSYRKXCAVSPTSYRTTGAVGPTFVR
SFCDNADDEGSDHDAESCSSSDLFELNHPVGVGRYMEELPVYETTNFRTNQAIAQGFX
>Mdo_MDP0000446864
MSRWENTPREDRYRHEKKNPSFSSSLLDKIYRSIDEGSPRNREDSMFYNETMPKKQSKSGAKSGRAVE
EEEMASLRRACLIEKWMEKKVGQKVGGQRRQHLGELDRKLDRDLDAMFFSSTSSSSDSSSGGFSSSDA
DSMFGSKSRSSYFAPPRPKPVRTSVSTRSVKSEEKTVRKQRTLFYEQREVHMFDDYHYSCASDPTPKL
DEGMIKSKSRALKIYSNLKKVKQPLSPGGRVANFLNSIFTAGQTKKTKSTSSIGGYEEAVAERKLKSG
QDSTCSSASSFSRSCLSKNSPSTREKLRNGVKRSVHFCPVSVIVDEDCRPCGHKCLYEGEDQTLMPVT
VPTAWKIGRSPARKAEEELKLRVLEKSRRVEEAAREILRDCRRNQVKNELVSRDCRDGDEGDDDDAAS
CSSSDLFELDHLSVIGKERYLEELPVYETTHVRTNRAIANGLIM*
>Mdo_MDP0000493758
MERWDIKSLPKNREGRSNRHSRDNPSFSSTLLDSIYRSIDEPTAGRDEVVSMREEQRHLIFYKGTMKK
KQSTTAATTTSGRLGHGHYKEDQEREIMSLRRACLVEKWMEKKAAENSVPARRNSMADFETTKSRYSH
QNEFLTNSSSSSSDSSGGFSSSESDSMYGSKSRSSSSCYSMHRPKPIRTSLSSEKVPFDDHQRNYHHN
SQKKHENGFGKTKSKALKIYGDLKKVKQPISPGGRLASFLNSLFNAGHVKKSKIDDLGAERKSLPNLG
QMGYSNPSSTCSSASSFSRSCLRKAPLSRSELSGISSSNGDAAKRSVRFCPVSVIVDEDCRPCGQKTL
LEAQSGLIASAKAAAAIKPPANEDVGLECCVMDVDEDDDHGRRIEEVARDYLIKNYQKKHDEDDEDED
DDDDDAESYASSDLFELDTAGVEERYRCLVRWLRVAPRLGNLNVRSCYCLGYLSSVLTHNFSVTRSSF
SVASNFFSLPAQLLPFGVLAPSPTAAVWKTSQLEDESKMKKKTPLGPESDSLLGC*
>Mdo_MDP0000873045
MSMISTGVLSADPGADKIHKKLFHHRNDSGELDVFEAARYFSGYNEAPSSHKNNKTSAFSQRMMKEDR
SSWRGGRISLDMPIRHMLHHPQQNPNHHNXVAMEKQSNIKDKKYKQPSSPGGRLASFLNSLFNQSASK
KKKSKSSATQSMKDEEESPGGRRKRRSSISHFRSSSTTDAKSVYSSSSSGFRTPPPYSHAQMVASNSY
KDLRSYSDNHEQQYQQQQQQQTVSLSKYNTNFDEKRSSNKELTWLDHEKFKLSEKYKISSDQDHKGLL
RRLSEVVDDEREDEGAESDSSSDLFELQNYDLGYYSSGLPVYETTNVDNIKIRSXTPISNASS
>Mdo_MDP0000277127
MSMISTGVLSADPGADKIHKKLFHHRNDSGELDVFEAARYFSGYNEAPSSHKNNKTSAFSQRMMKEDR
SSWRGGRISLDMPIRHMLHHPQQNPNHHNQVAMEKQSNIKDKKYKQPSSPGGRLASFLNSLFNQSASK
KKKSKSSATQSMKDEEESPGGRRKRRSSISHFRSSSTTDAKSVYSSSSSGFRTPPPYSHAQMVASNSY
KDLRSYSDNHEQQYQQQQQQQTVSLSKYNTNFDEKRSSNKELTWLDHEKFKLSEKYKISSDQDHKGLL
RRLSEVVDDEREDEGAESDSSSDLFELQNYDLGYYSSGLPVYETTNVDNIKIRSVTPISNASS
>Mdo_MDP0000156548
MSIISTGVLSADPGADKIHKKSFHHRNDSGELDVFEAARYFSGYIEAPSSHNNNXSSNTSAFSQRMMK
EDRSSWRGGRISLDMPIRHMLHHPQQNPNHHNHVAVEKQNNIKDKKYKQPSSPGGRLASFLNSLFNQS
ASKKKKSKSSATQSMKDEEESPGGRRXRRSSISHFRSSSTTDAKSVYSSSSSGFRTPPPYSHAQTAAS
KSYKDLRSYSDNHKQQYQHQQQAVSLSKYNTNFDXKRSSNKELTWLDHEKFKLSEKYKISSELDHKGL
LKRLSEVVDDEEEDEGAESDSSSDLFELQNYDLGFYSSGLPVYETTNVDNIKIRSGTPISNASS
>Fve_gene07538-v1
MERWDHKSQREARHISKHRRENPSFSSSLLDSIYRSIDEGEENIGTRREGELIFYKETMTRKKQNSTT
IVSSHGVKAEEEIMSLRRACLIEKWIEKKAAAADKVVVRRNSMADFDRRFLNSSSSSSDSSCGGGFSS
SESDSMYGSRSRSSSSCYSMPRPKPIRTSVSSTRPENHHQKPKQENGFVKTKSKALKIYGDLKKVKQP
ISPGGKLASFLNSIFNGGNGKKHKINIDDLGSKSTNGSNCSSASSFSRSCLRKTSVSRGELSNGGGDS
AKRSVRFYPVSVIVDEDCRPCGHKTLEEIKRSFVMDEDDHRRQVEQVARSYLMKNYQKKTHDVEMEED
EDDDDDAASYASSDLFELDTVGVEERYREELPVYETTYFDRNRAIANGLIFVRIWLQVGLTLVKVFSL
ATARDGADHRAPAAVAHSHVWASSQIPINIRCRHRHVTTAPLRPSNDITTTRALTPAAALAGESTKLT
SEIVGRGRLSMGSFGRRGRQGSSFRKRKDDHGGNGGLCAPVCL*
>Fve_gene12721-v1
MYMRERSWLEDERFVSGGGRRRRTPSFSSSLLDSIYRSIDESNGGGGDDSNKKQSKQSCSVSKREEIK
GGGSGKTKTNLRRAMMIENWVERQSVHSSMFTNSASSSSESSSGAAFSSSDQTESSYKRRSKQSPVKA
ISVGSDNMEKTKSEGGSGSGGGFTKTKLRALKIYGELKKAKQPISPGGKIASFINSIFNSGNVKKAKM
CYVGAVEDVSTSEHVTKSTYSSSTTPASTFSRSCLSKPSSRSKKSSNGNKRSVRFYPISVILGEDDSQ
ISSHKCVYEQDPSLMPKPTFQKYARTSGQNFRRSFCEDGDDDESDAESYSSSDLFELNLPVGIGRYTE
ELPVYETTNFRTNQAIAKGLIL*
>Fve_gene15408-v1

MMQTLAYEDTAALLCWERSKVPRFVQSLSSIHLVLLLFLGIPNAHIFQCISAASFLNSKIKWVLSSTS
SMNFRSAGKLVPKTRPYDAIMLLSNIGQDQDQENKHGMLHCARQKVNLKPEAIWDFTHFTKRDTKAFE
PLTLVPCFILSLSSITMSKWDSPSFSSSLLDKIYHSIDDGERRGEDVKFYGETKTTALPKRNSKSGRN
IAVEEEMAAIRRACLIEKWIEKKAKEKAEAERRSRKLERDHDDSVFFSSNSCSSESSSGGFSSSDAES
MYGSRPQRASCFVPSLWPKPIRTRPVKTEEKKTEKKQSALSRQQRDFCLLDDYTQSRITDRTMKLEEA
TIKSKSRALKLYDNLKKVKQPISPGGRVASFLNSLFTTGQVKKSKGSSSVSGYEETERRIYSLQGPTC
SSASSFSRSCLSKNSPSTREKLRNGVKKSVQFYPVSVVVGEDRQPHSQKCVYEEQDAHLMPVTVPTAW
KIGRSRSRRTEEEIVKLRVLEKSRQAEEAAREFLKDHRRNQAKNESLFSRDYNNHDDDMDDDDASCSS
SDLFELDHLAVAGKERYLEELPVYETTRVKCTNRAIF*
>Fve_mrna04564.1-v1
MSSIAGVADPAEKAHKKSFHRRNDSGELDVFEAARYFSGYNEAAAPTMMKEDRSSWRGGRISLDMPIR
NVLDHQLLHHHHHHAVDQKQNMKEKIKYKQPSSPGGRLASFLNSLFNQSSSKKKKPKSSQSMKDEEES
PGGRRRRRSSISHFRSASSTTTTTTDAKSIYSSSSSGFRTPPPYSAQIATAKSHKDWKISHSDHKPVV
SNLSKHDVMSHAKSSVSDEKVSSNRELNWLDEKLKYNNRLSLSEKYKSSTDQDHNKGLLKRLSEVVDV
DDDDGAESDSSSDLFELQNYDLGGYYSSGLPVYETTNVDSIKNIRGTPIANAS

>Gma_Glyma.04G055100
MYTTKREQQRLRFQNHAKKPSFSSILLDQIYRSIDEGNDMKLYNETMAKQQNRVFVEEEEEEEEITAA
SIRRACLLDKWKESEKAGSQWKTQTEGKSRWHHHHNHEHDQEVMFFSSTSSSSDSSSGLLSSSDTESL
YGMRSKSRVSCFAPSRPKPVVTSASNEVGLIKSESRALKIYNNLKKVKQPISPGGKLSNFLNSLFATG
GSVKKTKTYDDDAKASTKSGQDSTCSSASSFSRSCLSKASTSSKDKLRDGVKRTVRFYPVSVIVGEDS
RACGHKCLYEEDTRVTEVSVPTAWKIGRKKNQEDEELRVVDRSRRVEEAAREFLKEYHRSQKKSDFTN
LDVEDDGDASSCSSSDLFELDHLAVMGNDRYGDELPVYETTYVSTNRAIANGLI*
>Gma_Glyma.06G055200
MYTTKREQQRFRFQNDSKTPSFSSTLLDKVYRSIDQSNDMKLYNETMGKKQSRVVVEEEEITAASIRR
ACLLEKWNRSEKVGTQWKTQTRWHHHRHHEHDQDVMFFSSTSSSSDSSSGLLSSSDTESLYGLRSKSR
FSCFAPPRPKPVMTSASNEEGLRKSKSRALKIYNNLKKVKQPISPGGKLSNFLNSLLATGGSVKKTKT
YDDTKAPKSGQDSTCSSASSFSRSCLSKATPSSSKDKLRDGVKRTVRFYPVSVILNEDSRPCGHKCLY
EEDTRVMAWKIGRKKNEDEELRVVDNSRRVEEAAREFLKEYRRSQKKSDFINLRDFTNLDNDDDDDA
ASCSSSDLFELDHLAVMGNNRYREDLPVYETTYVSTNRAIANGLI*
>Gma_Glyma.07G036700
MDKWDNKPSRKQHHRENPSFSSTLLDVIYRSIDEDPTDEKEEAQLIFYRETMRNQKQSNCFREEKPEA
EKHNSRRARKVENWMEKKANEKVVMGRNSLTEFERRTRSNSISNTLSMYSSSTSSESSSVGGFSSSES
ESFYGVQRPKPIKTSVSDKTKTKTTFDASLHSHNFRSHSSQSQKPKHENGSGKTKSKALKILYGELKK
AKQPISPGAKLASFLNSLFTSSGNAKKAKVSTTTTTTTTSTYRPVLIPVATDRTADTKSAAQQQQQPG
STCSSASSFSRSCLSKTPSSRSGAKRSVRFCPVSVIVDEDCRPCGHKNLHEGEESNGKNRSEELRLHV
MQESRRVEELARDLLKNYQKKSEVEFDDVMHYEDEEEEEDDDDVASCSSSDLFELDNLSAIGIERYRE
ELPVYETTHFNTNRAIANGFIL*
>Gma_Glyma.13G108300
MHERSMKEAAGTCPQRRRTPSFSSSLLDAIYRSIDESKSNLHDDQQLGLHHHDQTTHSFTSEKGGKKE
RMNLRRAVMLEDWMEKHGSHSLNAQLLNSSSSSSECSSAGGIFSSSETDTTTTLTLKKQRPARLTSEK
KKKKQDRIMSSEKQNKESGFTRTKLRALKIYGELNQRVKQPISPGSRIASFLSSIFNSQNVKKAKMCY
AGAVEDVSFEHKSNSPCFSSIPSSFSRRSCMSKTPSSAKKSNNNEVKRSVRFYPVSVILGEDSEPQSS
YHKCNIIYESEPNLGVRSSSIKELKKNTARGNENGAEEAAARGFVKGYRNSGQGEFDFRGFYDDDDED
DDDDVSCSSSDLFELDHLIGAARYQEELPVYETTNLETNKAIASGLCL*
>Gma_Glyma.14G099400
MYHLEKPQRDDKQFLTPSFSSTLLDQIYRSIDEGERKNGEIKFYRHTTMSTSKRQSRSNSKSMDAGDR
KIVRAKNGRKKLHCDEDIMFFSSTSISSTDSSSLGFSSSDTESISRASCFAPRVGRRGGGSASFRSEK
QGMRVLDGFCRNSSHRSKHAHSPLPVSSRHHVSVSEQQRHEHVTACDEEALMIKSKSRALRIYNNLKK
VKQPLSPGGRVTSFLNSLFANTKKTTSTTSRSCGEGNAPSSSSSCYYSSTCSSASSFSRSCLSKTMSS
ERDRLRNGVKRTVRFYPVSVIVDEDSRPCGDKRLCEEEEASGEFLREYNRHNSKIKSNDNLVLKDLPL
RKTNLVEDGDDNDDHDDDASSYASSDLFELDHLAVFGSGRYSEELPVYETTHVSTNRAIANGLIV*
>Gma_Glyma.16G006100
MDKWDNKPSRKQHHRENPSFSSTLLDVIYRSIDEDPTEEKEEAQLIFYRETMRKQKQGNCFREEKPEA
EKHNSRRARKVENWMEKRASEKVLMGRNSLTEFERRTRSNSISNTLSMYSSSTSSESSSVGGFSSSES
ESFYYGVQRPKPIKTSVSDKTKTKTNFDASLHSHNFRSHSSQSQKPKHENGFGKTKSKALKILYGELK

KAKQPISPGAKLASFLNSLFTSTGNAKKAKVSTTTTSTYRPVLIPIATDRVADTKSVSAAQQQPGSTC
SSASSFSRSCLSKTPSSRSGAKRSVRFCPVSVIVDEDCRPCGHKNLHEGEESLVDSRGKNRSEELRLH
VMQESRRVEELARDLLKNYQKKSEVEFDDVMHYEDEEEEEDDDDVASCASSDLFELDNLSAIGIERYR
EELPVYETTHFNTNRAIANGFIL*
>Gma_Glyma.17G051100
MHERSMKEAAGTCPQRRRTPSFSSSLLDAIYRSIDESKSNLHDDQQLGHHHHHDQTLATHSFTSEEKG
GKKERMNLRRAVMLEDWMEKYGSSRSLNAQLLNSSSSSSECSSAGGIFSSSETDTTTTTLKKQRPARP
TSEKKKKKKKKQDHIMSSEKKNKEGGFARTKLRALKIYGELNQRVKQPISPGSRIASFLSSIFNSQNV
KKAKMCYVGAVEDVSFEHKSKSPCFSSTPSSFSRRSCMSKTPSSAKKPNNNGVKRSVRFYPVSVILGE
DSEPQSNSHSHNKKCNNIYESESNLTVRKITRSSSIKELKKNTVRGKENATEEAARGFVKGYRNSGK
GEFDFIGFYGNGENENDENDDDDDVSCSSSDLFELDHLIGAARYQEELPVYETTNLETNKAIASGLCL
*
>Gma_Glyma.17G225300
MPIMYNLEKPQRDDKQFLTPSFSSTLLDQIYRSIDEGERKNGETKFYRHTTMSSSKRHNRSDSKSMDA
GDRKIVGGAKNNNRKHLHRDEDVMFFSSTSISSTDSSSLGFSSSDTESISRASCFAPQVGRGGGGSAS
FRSEKQGMRIFDSFCRTSSHRSSEHAHSQFCITSRRHLSVSEDHKQIRHGHRQVTACDEEEEALMIKS
KSRALKIYNNLKKVKQPISPGGRVTSFLNSLFANTKKTTTTTTSTSSRTCGEVNTPSSSSSCYYSSTC
SSASSFSRSCLSKTMSSERDRLRNGVKRTVRFYPVSVIVGEDSRPCGHKRLCEEKEASREFLREYNNR
HNPKIKSNDKLVLKDLSLRTTNVDVDDNDDDDDASSYASSDLFELDHLAVFGSDRYSEELPVYETTHV
STNRAIANGLIV*

>Gma_Glyma.01G063800.1
MSIAGLIDPEMNHNKSFHRRNDSGELDVFEAARYFSGYSEVVGYTSGSTTYTQKIMREERHHHGQRAA
RISLDMPMRSLLPQQFHGMEKQIIMKEKKNKQPSSPGGRLASFLNSLFSQSASKKKKSKSGSQSMKDE
DESPGGRRRRRSSISHFRSSSTADSKSLYSSLSSEFRTPPYVQTPTKSCKEFRTFSSEKKHALSFSAK
YNNNNGQQIRSSTTTTLQNELLWDEKKKREPTTTTTTTMLDDNSNHKHLSEKQRNNNNNKGNHELLLE
KDRILVDNKYSSEEKETTTQFKNLNEVDDGAESDSSSDLFELQNYDLGYYSSGLPVYETTNMDSIKRG
APISNGPL
>Gma_Glyma.02G121400.1
MSIAGLIDPEMNHNKSFHRRNNSGELDVFEAARYFSGYSEVLGSTTTTYTQKINMREERHHHHHHGH
RAARISLDMPMRSLLPQQFHGMEKQIIMKEKKHKQPSSPGGRLASFLNSLFSQSASKKKKSNKSSSQS
MKDEDESPGGRRRRRSSISHFRSSSTADSKSLYSSLSSGFRTPPYVQTPTKSCKEFRTFSSENKHALS
FSAKYNNNKNNNGQHVRSSTATTTLQNEFLWDEKKKREPTTTTTLLDDNSNHKHLSEKQKNNNNKGSH
ELLLEKDRMLVDNKYSSEEKETTTQFKNFNEVVVDDGAESDSSSDLFELQNYDLRYYSSGLPVYETTN
MDSIKRGAPISNGPL
>Mtr_Medtr1g015275
MYKFEKRSTIHDRFINPSFSSTLLDQIYRSIDEGETKLTTETTKFYREQKPTVINKPTTDRKYYNYEP
KTEKLVTVVKETNNNRKSNQHDHDQDALFFSSTSISSDSSSSGFSSSDTDSLYRTKSRSSSCFVPPRP
KPVRTSTSASFRFEKEKHGNHVFDDFCRSSETKQGTETRGEEVILIKNKSRAVKIYNNLKKVKQPISP
GGKLTSFLNSLFVNSNNEKKMKTEKPTKTKKQMNTWETHEGKVASTCSSASSFSRSCLSKTASFCGRN
DYKTVGFCGVEEGRAKVEEATRKFLNEYHSRNKKKKKDDLVLLKDLCINQNEEEDEDDDDVASCASSD
LFELDHLSVFGDSRYCEELPVYGTTRVS*
>Mtr_Medtr3g104880
MYKFENTHIEKRFHNNFENHSFSSTLLDQIYRSIDEGDRKVSDMKFYTETTFQKQSKTNAKFNRVFEE
QQPYLRGVCKEKEKITTQIDRKLHLDHEIHDQDVMFFSSTSSSSDSSGLLSSSSETESMYKAKSRGGS
CFAPSRPKPVKTTVPPERRIIANDEDTLIKSKSRALKIYNNLKKVKQPISPGGKLTSFLNSLFINTKK
TKTVSSYEDSNAERKGKPGQASTTCSSASSYSRSCLSKNSSKSRDKLHNGDKRTVRFYPVSVIVDEDN
RACGHKYLNKGVTKKNEEVVDKSKKEEEVAREFLREYHLNHKILRDFSMKKNEEVDDDVSSCSSSDLF
ELDHLDVMGNDRYCEDLPVFETTHVSTNRAIRIM*
>Mtr_Medtr4g125620
MHTRERSYPQRRRTPSFSSILLDTIDHSIDQSKTDFVDDVNNQPCLYNQPIKHVKFNEKCVNSKQRMN
LRQAVMIEDWMEKNSSSSSSECSSGGIFSSSDTDSSYNRQRSRTKYNPPQKHMNPIHHSNSEKQQKKK
QQVWEDGFTRTKLRALKIYGELNQKVKQPISPGSRIATFLSSIFNSHNVKKAKMCYVGAVEDVSFDHK
SKSPCFSSSVSSYSRRSCMSKTTSKSNNGVKRSVRFYPVSVILGEDSEQQHQPSTIRKITRNSSVNEL
KNINKVVMAKERFYDNGEDDDEDEDDDALSCSSSDLFELDHLVGGGRYQEELPVYETTNLEINKAIAN
GLCVNL*

>Mtr_Medtr8g019660
MEKWNKPSRDHRKQHQRENPSFSSSLLDVIYRSIDEGHHKTEEKEEKLMFCRETTTTMRKMKSVEAEK
PNFRKARKVENWNGRNSLTEFETRTTRSNSNTLSMHSSSSSSESSSAGGFSSSDSESFYGLQKPKPIR
TSVSEKPNIDSFQYGYSARNHNYNHNHNHSVQTQKPKNENGLGKTKSKALRILYGDLKKSKQPISPGA
KIASFLNSLFTSSGNTKKPKIPSSTATKTTTNSVLEAKSAQASTCSSVSSFSRSCLSKTPSSRSGAKR
SVRFCPVSVIVDEDCRPCGHKNLHEGEKGSSIYDGKNNSSEELRLHVLQESLRVEELARDLLKNYQKK
NEVDFNMQFEDEDEDDDDGASCSSSDLFELDNLSVIGIERYREELPVYETTHFNPNRVIGNGFIM*
>Mtr_Medtr5g046750
MSLAGLIIDQEMNHNKKSFHRRNDSGELDVFEASRYFSGYNEVIGYNNSSTFTQKIMREERNGYKGRI
SLDMPMRSLLPQQFHGGIDQKQMKEKKHNKQPSSPGGRLASFLNSLFNQSTSKKKSKSSSQSMKDEDE
SPGGRRRRRSSISHFRSSSTADSRSIYSLNSGFRTPPYVNTPTKGCREFKTLLSDQKNEVSLKKSSTT
LQNELCWDDKKKRDSNLKQFVEKKYYEEEKREVRKFNEVDDGAESDSSSDLFELQNYELSHYSSGLPV
YETTNMDNIKRGSTISNVPL

>Lja_Lj1g3v1788080.1
MIRRERRFLNESENPSFSSTLLDQIYRSIDQGDSDMKFYTGKTGEKQSRVCVTEEHLSAKSSVSRDWK
ANNVVGTQGRQRMMQKERNFHLGRQVHVHDQDALFFSSTSSSSDSSSGLLSSSSPDIETLYGVRSRGS
CFAPARPKPVRTTVPEKKHVERQRHIVSDDDDGLIKSKSRALKIYNNLKKVKQPISPGGKLTSFLNSL
FTTGNSNNTKKTKPCFEDANALRKTESGQASTCSSASSFARSCLSKNSPSTREKLRNNGVKRTVRFYP
VSVIVDEDSRPCGHKSLYETEDARLMAMSVPTAWKIGTKKNVEEVKDKKNRAMEEAAREILEEYHHIQ
RKSDLVLRRNLEDEIEDDDEDDAASCSSSDLFELDHLHEELPVYETTRVGTNRAIANRFIM
>Lja_Lj5g3v0465910.1
MYKLEKPRRDHNKLQSPSFSSTLLDQIYRSIDDGQSKSSESNFYRETTTTTTSKRHWTEKKDRTDNNN
NKVRSKNVAVVEETNNRKFHHDHDPDVLFFSSTSISSDTSSGGFSSSDTDSTWPRPVRTSASFRWENE
KHATRVQSFMHLTAQHKHGVTGVRDEEALIKSKSRALKIYNNLKKIKQPVSPGGKLTSFLNSLFATGE
AKKKKKKNDEIVERRVNSGLGSSSTCCSASSFSRSCLSKVSSCDRENMSNGVKRTVRFYPETVEEKKI
VVEKAAREFLREYRYNNQNKKTDLVLKDLSLRKNANYSEEHEEEDYDDAASYASSDLFELDHLAVLGN
GLYDEELPVFETTHFSSNPTIANGIRV
>Lja_Lj0g3v0252339.1
MEKMMPREEGFPQRRRTPSFSSTLLDAICRSIDESKSNVDEDPQLGLFNETTNPKQSISKQSNSTHCA
NIAEKSGNKERMNLRRAVMIEDWMEKQSSSQSSHFTTSSSSESTSGAGFSSSEAETTTHKSKPRHKSE
KKQQQQQPQKPKREGDGGGGGGFSRTKLKALKIYGELNQKVKQPISPASRIASFLSSIFNAGNMKKAK
MCYAGSTQDVDFNHKSKSPCFSSSASSFSRRSCMSKTPTSSSSSSAKGKRSVRFYPVSVILGEDSQND
PSLLPVSSSVRKMTRVSSIKEVKKNSAKVKENVLMKGYQNSCKNNQFDFRGSYDHGEESDDDDDDDALS
CSSSDLFELDHIVGAGRYQVELPVYETTNLERNKAIANGLCL
>Lja_Lj2g3v1227800.1
MSITGLIDPEMNHKKSFHLRNDSGELDVFEAARYFSGYNEVVGYTGSTLAQKIMREERHGHRARISLD
MPMRSLLPQQFHGMEKQMKEKKHKQPSSPGGRLASFLNSLFNQSASKKKKSKSSSQSMKDEDESPGGR
RRRRSSISHFRSSTLSSLSSGFRTPPYVLQTPTKSCKEFRTSSDHKHTVPLPTKLNGHAKQNTLQNEL
KKDSTFLDDKYKHSNNGLSEKQKNWGNELLEKDRTWLEKYSLEEKDIRKFNEVDDGAESDSSSDLFEL
QNYDLGYCSSGLPVYETTNMDNIKRGAPISSGLL
>Lja_Lj0g3v0201679.1
MEKWEKTSRDHRKQHHREKSPSFSSTLLDVIYRSIDEGQTEEKEESLIFYRETMRKQKQSNCFREEQS
VQIEAENPSFRRTRKVENWTEIRNSLTELERRTRSNSNTLSMYSSSSSSESSSAGGFSSSESESFYGV
QRPKPIRTSVSEKLPNPDDGHRSFSGRSKPSARPNTAQVGPNGHTASLNDTGRETVWTGSIGASRPKQ
HGFGKTKSKALRILYGELKKAKQPVSPGARLASFLNSLFNSGGGNAKKAKVSTTPPTTTTKVSNSAQP
SSTCSSASSFSRSCLSKTPSSRSGAKRSVRFCPVSVIVDEDCRPCGHKNLREADYGKSNDEELRLHVM
NESRRVEELARELLKNYQKKNAGDYDRMQCEDEDDDDAESCSSSDLFELDNLSAIGIERYREELPVYE
TTHFKTNRAIANGFV

>Vvi_GSVIVG01015116001
MTGPLLGLSTDSPVTSSLRTLPLSLSRLLFIYVHPKLYRESREQLERMERVQQRRKTPSFSSSLLDSV
LRSIDESSGERQQLLMSKKQGGAEEEEIASVRRAMMIEKWMRKQSGGSSVSSVVFNSAASAGSERSRK
VAKLVEQRNSAPKLGENHGGRGGGGGGGSFLKTKSKALKFYGDLKKVNQPISPGRRIANFLNSLFSSG
NAKKAKMCSFETVDDMSSERKVVKSVQESTCSSASSFSRSCLSKTPSSSRTKLKRSVRFYPVSVIVDE

DCRPCGHKCLYEDDPSLMPTLTAKNIPQTAWLKGELNEKNTSPANIADMRQIVRDYQKKISHEFKLAD
LCHNEDDDDDDDDDDDAESYSSSDLFELDHIAIGRYQEELPVYGTTYLGTNHSHRA*
>Vvi_GSVIVG01016780001
MGANLAGVEVGFVVHALEGDIRASAGERHRALFSLLSPQAWLLAFISLLRAIKHPSMAVSALFPFSFA
FTQGSSIIRNAAFQLSRVFSRFLAWALGFFYKEMQRWDKSLREDRYRNGRENPSFSSTLLDAIYRSID
EGGEGEEELVLYRETMRKKHTIMIEKWMEKKVSEKVVVRRKSMADFERRSRNDRDSFFLNSTSSSSDS
SSGGGFSSSEAESVRNYIASQKPKHEGGFVKTKSRALKIYGDLKKVKQPISPGGRLASFLNSLFTTGT
AKKAKISSSEDSTPERKSKSGHTSTCSSASSFSRSCLSKTPSSRSKLSNGTKRSVRFYPVSVIKNESY
LREAANHEDSDDDAASCASSDLFELDNLSAIGIDRYREELPVYETTRMDTNRAIASGLIL*
>Vvi_GSVIVT01012248001
MSVAALSEADKIYKKSFHRRNDSGELDVFEAARYFSGGNEIIGRRNQSPQHSRSRMKRRARVGGGKGG
AALAISGVPALLIPSPCILPQVLALELPLLMQILPQRPTRISEAIQITGKWCLGREENQRVGLAGREI
QIQHWMRLMPVRRVIQALIYSSCRTMTWVATQVAYPYMRPPTWTASREEHPFPMAPYPYNNSFLHFFL
CMYSWECLLVFHFRFLFFNLQLLLEVCLWSAGALEVHHWNCLIRLRDDLETFH
>Vvi_F6H3R8
MNKWEKSLREDKSSHERKNPSFSSSLLDEIYRSITDGDEDHKELHFYRETMAKKHSLRGSRGVGEEEM
ARVRGAGVTDKWMEKKGSEKVVSRHQRRSLPEIDKKWQYYQDPLFFSSSSSSSDSSYGGFSSSETESM
KSFFAPPPRPRPIRTSRLERSEQALSYGQSEFHMFDDYRHHSAITREPSKSEEEIIKSKSRALKMYGN
LKKVKQPISPGGRLATFLNSLFTAGNTKKSTKTTSSAGGCEDWSSERKLNSQPSTCSSASSFARSCLN
KNSPSVKEKSRNDAKRTVRFFPVSIIVDEDCRPCGRKCVYGEEGSTQMSASRKITKRNEEDLKFQDQT
RRVEAAARELHRNFHQKKNDNIFNKFHVNYDEEDEDDDAASCSSSDLFELDHLALIGNDRYQEELPVY
ETTHIDTNRAIANGLLV
>Mgu_A0A022S3L0
RRESNISHTQFTHSSSHFGGYSSSSSNSCPSFSSVLLDEIYRSIDRPEEEMHTITKKSDGHANVRPAY
IKSDELGIPANDYYQRACMVEKRMEANKTRKIRHGSSSASSWDSLGGSGGGGGGGGFFSSSEAESFRS
QKFGGGGEINTDPEVIISPRDRRGFEDRMGQKSKTKLRALRIYGDLKKMKQPISPGRKLAGFLNSLFT
AGNLGLKKKQTGTPTIPGGENSPTLKSTNYPSTSSSASSFSRSCLSGKMPPPPSGKSSGGGRSRKVSF
FPMSAISDDEDFPNNNNNVSIMKTAIDEEIMAHMMKKNRRVGEAARDFLVNNYQKKTETEEEEEEEDA
ASCGSSDLFELDIFGALDNGEIHTRV
>Mgu_Migut.L01171.1
MSAVITQWRNNNSGEIDVFEAARYFSGGADQNPINTAVPPPPPQRALACRISLEMPKNPYSNLSAAAA
AAMDHGGDQKKQEKKKYKQPSSPGGKLASFLNSLFNQTTSKNTTTKKLMKKKNTSVKSGSSNNDLEEE
RKRRSSISHFKITTTSDSSNKSTYANMMATPTKSSLRSLSHHQIELGVSAPSRIINAGVDQKRTISDN
NIISYRNNNAGFGFSDRSSTVMNRKFSDDDDDDEDGAESDSSSDLFDLPNHDLDFCSASSGNLPVYGT
THMDRIRISAPISAVATFL
>Mgu_Migut.A00091
MYTREENYHYKTRKNSMNTPSFSSTLLDEIYRSIDGGAAAAEKPEGFNEKPLRKQGGGGCASVRRVCL
VEKWMEKEANENVVAKKTAAAALLPELDNNDLLFFSSTSSSSDSSGALSSSSETEFFGSSSTKKPPKV
SCFSSTRPKPVRTGATTRVETKPQKDNRFLFDDYNNNSNKKNQDHQKSKTGDDLLIKSKSRALKIYAN
LKKVKQPISPGGKLTSFINSFFTNGNNVINTKIQDSSKSSPKASSTTCSSASSFSRSCLSKYSPKSRE
KIKNGVQRSVRFNPVSVVVDQDLRPCGQKPVYERPPLPPNSLAKLKFSKMEKNIEVEDAAKNVLRGYC
SKNGDNLSLFRKICDEQYDDEDDDDAMSDSSSDLFELDHLAFFGDKRFCEELPVYETTRLDTNRGVVS
RLVR*
>Mgu_Migut.E00039
MLSSNPNKNSLQWRKNSGELDVFEAAKYFSAANENEQNSDNVLSGTNLSQAIIVREDRQNILRRSRLS
LDLMPNDNPISHQKMIVKERHQPTSPGGKLASFLNSLFNQRHYSKTTKKKNNKSKPEKDFDDENTPGG
GRRKRRSSISHFIRITSSNNNNNHTTYPNTPAKQVIISLPKNTGNSNLNPASMDYECVKEKSRFSNGV
FSDKISRNGSFGFSSNKDDYEGAESDSSSDLFDLPNHELDFGSSDLPVYETTRVNIIRIAKPISRATT
TTTAV*
>Mgu_Migut.E01574
MYCRAKPSREENHKPKKCHRNTPSFSSTLLDEIYRSIDGGGNAEKAGAEINPGSHREKPATGLRSNNG
GRRSSTGNHHEQTAAARFSRGCLARNNDKDLSFDSTAKSEVSNQNEAADFTREEANEFINSLFTNLNS
NNKKTKKQDPKQQSSKVTSSSTPSSSTTSPSSRLSKHSSSIMRNNEIGINQPTVRFHPVGRPVSEDQD
SPCRPKSIVYDNDRKLYGRPPLPPNALARNRNINQRRSDFSMFEEIDDEDGVSDSSSDLFELDHLVLF
GNTNIFCQELPVYETTYFDNNRAVAAGLFCN
>Mgu_Migut.I01063

MVDGYSTNYRRQSDINTSLYNPSFSSTLLDTIYRSIDQGGEEEKLGMRTKQSIAYGSDGGAGVFRSEE
EMANFQRACMIEKWMEKKAGEKVAVRGNSAADLGMKKPRKDRENSTSSSSDSSCGGGDFFSSSEAESF
PVQRPKPIRTGLEKEKLEKNHRDSESEFHRRERRGFAGDVEQKPKHEGGFLKTKSRALKIYGDLKKVK
QPISPGGKLAGFLNSIFAGGNLKKPKTAANGDYSPSLKSANASTCSSASSFSRSCLSKTPSSSGGSKP
SNGAKRSVRFSPVSVIVDDKVNNNNNYTKKQTSKFTATAANNNNNKNNINSLRNVINEELMVHVMDKN
RRVEDAARDLLRNYHRKNQVGCEFDSSFDHLRKPQVIKNNQIINQNNFDEEDDDAASCASSDLFELDN
LSAIGMERYRQELPVYETTHL*
>Mgu_Migut.J00952
MAVGERQTQKHRRKTPSFSSSLLDAIYLSIDEPGCAAAAASQPHQQSEDFVHLRSNTRRNNAAHFEDE
IASLRRAITVEKWMENHTTTTTVVAAAAAAAVPRRRISSNSGTSSDSSILSSSSETDSSVLSRSSSSS
KNHLYTRTKSKAMKIYGELKKVKEPISPGGKIVNFFNSIFSPRNPKQKQTTVEEWSSSIRKSRSMRDP
PTTSSKSCLIKNPSSSICNKSKRSVKFDENEYSVNSSMPSVKSRLIKKSVELFENESDGDDMSCASSD
LFELENIGSYGGEELPVYGTTSIKMNRAIACGFAM*
>Mgu_Migut.L01544
MAVWEKQAKNKEPSFSSSLLDSIYRSIDENGAPNEQKMEDNFLLHRRNNAAAKVEEEIESLRKAIMIE
KWMENYKPINTTTTTTTMHFPSNSGSSTDSSIFSSSETESSVSRSYKIPQLENKPATKKSRAQKIYGD
LKKVREPVSPGGRIASFLNSIFSPRKTNELCSMRKSKSMKDTTTTTTTGTTTTASTKTCSLESRSCLS
KNRSSGDCKSKRSVRFCIVDGDCQPCGNKSGLFYDEKEDLVNTTVANIGSYFIKKNTKNARKNQEVRD
LDELSCGSSDLFELENIGRYEEELPVYETTNLELC*

>Nnu_XP_010261958.1
MDSWEKSLRDDRFLRQERKNPSFSSTLLDAIYRSIDEGDGEGEQELVLYRESMGRKQSCTSSGLKGRS
FLEMDSLRRGCMIEKWMDKKVSEKVVFRRKSTPEFDLKLQNDRNSTLLNSTSCSSDSSSGGGFSSSEA
ESVYGVRSRSSGFTAQKSKPVRTCIPVRSEKPLHYYDHELEIKLHAFEETHRRHHLEKDSVPQQKPSK
NEGGRFIKTKSRALKLYGDLKKVKQPISPGGRLAMFLNSLFTSGNAKKAKISSSVGGGEDTCSERNSK
SAQASTCSSASSFSRSCLSKSPSSRGKLSNGLKRSVRFYPVSVIVDEDCRPCGHKCLYEDDPGLIVRP
IAEPRLMNEELKFHLMEKNRRVEEAARDLLKSYQKKSDFEMRDANVDVRDDDDEEEDDDDDAASYSSS
DLFELENLAVIGIDRYREELPVYETTHLDTNRAIASGLIL
>Nnu_XP_010273945.1
MDRWHKSLREDRFCQERKIPSFSSTLLDAIYRSIDEDEGGEEELVFNREAMRKKQSIATDSNVRGSSF
LQEDRASLRRACMIEKWMEKKVTEKVVFRRKSTPDLDPKLPNDRKSVLLNSTSCSSDSSSAGGFSSSE
AESVYGARSRSSGFTAQRPKPVRTCIPAWSEKPLQYYGHQEQRKLHAFDEANLRHHQERDSLPQQKPS
KNEGGRFIRTKSRALKIYGDLKKVKQPISPGGRLAAFLNSLFTAGNAKKAKISSSACGGGMEDTCSER
ISKSAQASTCSSASSFSRSCLSKTPSSGGKLSNGMKRSVRFYPVSVIVDEDCRPCGHKCLYEDDPGLI
VRQMAEPRLMNEDLKFHLMEKNRRVEQAARDILSSYQQKKSGFDIRDAEIDVCDEEDDEGDDAASYSS
SDLFELDNLAVIGIDRYREELPVYETTHLGTNRAIASGLIL
>Nnu_XP_010257962.1
MSITGFSHSDKIYRSIHRRNDSGELDVFEAARYFSGCNEVPGLMNGASFSQRNMREERQMWAGRRMSL
DMPMRNPLPSESHHNIESQIKEKKHKQPRSPGGRLASFLNSLFHQAASKKKKSKSSAESMKDEEESPG
GRRKRRSSISHFRSTSTTTDSKSLHSSSSSGFRTPPPCANTPTKMFKDIKSYSDHKQVAPLSTKYTCG
QVIRSVNGLGLHHDEALNEKRNMDLSWLDEKLKFNDGFREKTKNVNSGFLEKDRTWVGEYNSSEKKNF
TSFGEVDDGGESDSSSDLFELQNYDLGFYSSGLPVYETTQMDAIKRGAPILSGSCGTR
>Nnu_XP_010260799.1
MSVTGFSYPAERVYRRVHRRNDSSELDIFEAKQYFSGCNEVLGLYGAALSHKAMREERQEWGMPMRHT
LQSQSRQIESQIINENKQKKPRSPCGRLVNFLSYLFHQAVSRKKKSKSSTQSEKDEEESSGGRRKRRS
RSMSSHFRSTSTTTDSKSMRSSSSSSGYRTPPYANSTTSTNMYKDLISYSDHKQVVPLPSENNEKVSS
ISLKREGLNKKRSMDLTWLDEKLLLKFNCGLEEKTNNLNHGFLEKDRTWVEDYSAENKSFTRFDDVDDG
GESDSSSDLFELQNFDIGFY

>Aca_Aqcoe3G372500
MSIKGFSQEKVHRKSTQRRDGSGELDVFEATSYFSGCNEAAGYNANLFSQKELREERQTLGGRRMSLD
MPSSASLPSQSHRVEKQTKEKKYKQPSSPGGKLASFLNSLFNQAASKKKKSKSMKDEEEIPGGGRRKR
RSSISLFQNIGNNDSRSLYASSTASGFRTPPAYIDTPTKAYKDPKGYSDHKYVVSLPKYTGKVNSVSS
QDEAVDEKKNMDLSWLDEKFRFIDEFSEKDKNMFKTDRIYVDKFDKFSSEKNNFVKKQAVVDHEVDDG
AESDSSSDLFELGNYNLGSYPNDLPVYETTHMERIKRGPPITNGLL*

>Aca_Aqcoe7G116200
MDQWTKSLREDKVRTRSRNPSFSSSLLDAIYRSIDEGEEDLVLYKETMKKKQTTSTTTTNSLKRDEKN
IDDFTRACLIEKWMEKKVSEKVVVKRQSVRDFDQKLQNNRSSSTAFHSASSSSDSSSAGGFSSSEAES
LYGSRIQRPKPIRTSVSAKPESFEKPLQYEQRKSTNIYESHYHHQHERISSVPQKVKQEGGFNKTKSK
AMKIYGDLKKVKQPISPGGRLATFLNSLFTAGNTKKAKLSNSVNGIEDLRSERKSKSTQTSTCSSASS
FSRSCLSKTPSSRGAKSTNGTKRSVRFYPVSVIVDEDSRPCGQKSIYEQQQQQQSKVVSMNEEFKYH
LMEKNRKIEEAAREFLKSYQDKNLEKKKCDYEMRDVHQDYEDDDDEDAESYASSDLFELDNFASIGNE
RYREELPVYETTHLEKNRPISNAYIL*

>Atr_scaffold00024.196
MDRWSYSEKLERESRHAHRNRNPSFSSALLDAIYKSTEEDQHLVFYKNAIKPHINQSFFEPANVHQSA
VKRSHKAEKIVEKRVEKTKPRRKATQLGVFEAEHYYETLLYGRDHHNNISSSSDTSDSATNYGFSSSE
AESAWSRRSDPSSRKSSKSPSTQFLGVSKEEVAEKKAIKSRASKLYGDFKKVKQPISPGNRLASFLNS
LFSSAEKAKKKAKLARVETDFRACENKPPPLPSSSTCSSASSYSRSCLSKTPSSRGKSGPNGVRRSVRF
YMPEETAELTTSATVRRSVKFYVPGEAPELTESDAFRVPFHEKLKLHFMEKSQKVEAGKELFNEYQKK
SEIIKEVIMRDFVDSRERELGLEEEEDDDTESCSSSDLFELENLAAVGIDRYQKELPVYETTDLGTNQ
AIAKGFLL
>Atr_scaffold00047.124
MLTNGADHGKPRRRNDSGELDVFEATRYFSEERSNLSTNFAKNGSISQKAALNREGSTCGGERRILDM
PMNKQSSSPQKKIKEKRSKQPSSPGNKLASFLNSLFTQNSSKKKSMFSNQSIKDDDESPFGRRRRRSS
ISLLQTASSNDSTYNSSSSSDFRTPPPFTATPTSLGRELNSFYDLHSMVSLSSCTRNHTSASLVPTNG
ALEGKVTNSMGWSDHKSKYESEFLERESNWGYVYSSEKGIRRISEIDDGGESDSSSDLFELQSNDFGV
YSKGLPVYETTHLESIQRGAPIANGSF
>Spo_Spipo0G0137500
MEKLQRTYHHRRNDFIATEAKSRQPHHHPSFSSCLLDAIDQSIDKGGGAGGGRCEIEGAGSSGAASAR
LRCSTRVSADEKKRVDQRSYSRHAAHAREPEDRRFAGRVSEKAAAAPRHRGATAEYEQRQPHRSRARE
RDRFCGNSTSSSSESSYGGFSSSEAESVVRSARHLPKPVRTGGTPERKAEQKMKGSIRSRFRASKVNG
EMKNGRTPVSPGARLAGFLNSLFSTAAHPKKAKVISAASNSAVRSVIRGGDESGCSSASSFSRSCLSK
PASNGGGSGGGAAAAAATKRSVRFIVEEECYPVCHESHYLPRLTAIRVREEPLLPPPLPSDLVAAAVA
EERRRVAEKARKLITAYEEKKRTSATALADEKEEDEDDDAASYCSSDLFELKDLSAIGGGGGDELPVY
ETTHFSANHAISRGIIV*
>Spo_Spipo0G0184800
MERWEKLQHRGGAAGTAAAGKYHRSFSSSLLDAIYRSIDDDDTVGGRGLPVAAAAADTRRRELHRAAA
PPPPYPAVWNHHPCRCRNHREERFFPRSAAAPPSSTLWNHHLHRCRKQREERSFPKSAVAPPSSASWN
HHHHRCRKQREERFSPKSAAAPAPSAAAPPPATSPESRSRRRESSIMEIFRSSKVYSELRKAKTPISP
GGRLTSFLHGLFSAAATPRKSKPTGAAAVAPPPPPPPACMVPAQRGEERKLAVERSAVAEARFHERKR
AAEEGEKDDDGDDDGGSCSSSDLFELENLTAISAAGGGEKRFMDELPVFETTRLEPRRRPPRCS*
>Spo_Spipo2G0082400
MKQADELDVFEAAGYYAETADAASVQGGWNMAEKGWRGGERRTEGPPPLQKVRDKKPKQPLSPGGRLA
SFLISFFHHGSSRQKKSALSISGGASVSFKHEDEGRSTRRRKRRASESSLFVYSSRSPCKANVDKKTH
PTSWRRGISKAASGPAREVFHGGRSGYGDPLREGGRRVSLNGILEKDSRFYCESLLQLEERTVWRRGE
EEEEQEEEGGEDGWESDSSSDLFDLPNCELVRI
>Zmar_Zosma188g00060
MERNNTHRRLTPSFSSILLHAIDRSIDEADGIGNTRVQTPCTCNRSRSNSTTPAAATYNQQQQYGGT
RNPKLRVKEKRFSSSEAESSVGGSLVDVSPTNKQKNKQKKKKQDSKSLGNRFRASKLYAEIKKAKPPV
SPGSRLASFVNQLFTSTGKTTTTNQNLIDRYRSYSTTSSSSYLNKALSSKSLNNSKLTVRVDEEIVMD
DDETRSYCSSSDLFELENLTGVGALDNYTNELPVYETTHLQTNRVIARRLIV*
>Zmar_Zosma87g00320
MDKSGTCYCCYCRRREAAANPSFSSSLLDAIYRSIDEGDDGNNDRPTRPRYCGIQKGNISLNITAVEN
KKKQVTTVVTEKANARQLTTVKTYDRRGCKPNQDKEVKERRFINSASSSSESSCGGFSSSEPETTRLK
PFARPVLSSFEQVKQKMEKHPNSLGNRFRSSKIYGELKKAKLPVSPGGRLSSFINQLFNSASHLKKVK
TSLPVGNTKDGLGDDSTCSSASSYSRSCLRKNPSTRSKTANSGKRSVRFYPVSVIDSDDLCPSINKCL

DKDCNCGYKVTDVSPRMHPLPPLRVKDPTVEDMKARDMIERAYANGKRSLFRFDDDDDDFDDAASYSS
SDLFELDNLVTIGDGCEEAPRDREMYSNELPVYETTLFRNNRAIPKQVVV*
>Pda_XP_008797636.1
MERWAKPPVRRGNDNPSFSSTLLDAIYRSIDEADGGAAREHFTGGAPDRRDASVPAAAKKQQSFFTDE
WPAEGRRTAVVSEKAVTRLRKPENRPRFPVNSTSSSSDCSSYGGFSSSEAESVPRPSGLRPIRTGGLL
YRSERARSNPAPPQPPAVGSPSAHHHQEKKKSGSIRSKFRDLRKAKAPASPGARLASFLNSLFTANGN
SRKSKSAAAAYGGSGVRGGEESACSTATSYSRSCLSKTPSSKGRPAVAAAAGQGVKRSVRFYPVSVIV
DEDCRPCGQKCLYEGDSAAVARRPPEAATIAKKRVEELLRGFEEEEEDEEASDSSSDLFELENLTAIG
GRGYRDELPVYETTHLGTNRAISHGFFL
>Pda_XP_008787849.1
MERGPKPALRCGNAHPSFSSTLLDVIYRSIDETDGGAVREHRSGGAPDRLYDRGPAAAKKQQSSVADR
WPAERRSTAAVTRLRKPDNRRGFLVNSTSSSSDCSSYGGFSSSDAESVSRPPGLRPIRTGSFLYRSEE
ARSRPPAPPPPVAASPPAHPHQDKKKSGSIRSKFRDLRKSKSPASPGARLASFLNSLFTAAGSPRKSK
SATAAAAAAAGPASGGGGGTGGEESACSTASSYSRSCLSKTPSSKGLPASVAGEAVKRSVRFYPVSVI
VDEDCRPCGQKCLYDGDPAAGATRRPPPPAAVAKKRVAELLRGFEDEEDDEEGSDSSSDLFELENLTA
IGGRGYRDELPVYETTHFGTNRALSHGLIL
>Pda_XP_008791915.1
MERWERPPSRRGRNKPSFSSSLLDAIYRSIDESDGGGGGGPTRSHGKSGAPDPSFSVSSSSAAAARKK
QKADELKWTAAAVSERAVIRRGTVDYRPGLVTPTSSSSDSSSYGGFSSSDAESYYSAGVRPVRSDRIR
SDPEIPPEKKKAGSIRSRLRDLRKSRATGPTSPGARLASFLNSLFAAAGNPKKSKISAPPPPPAAAVE
SACSSASSYSRSCLSKTPSSRGRRTPAAASAEGGKRSVRFCPVSVIVGEDCRPCGHKCLVDEDLAPPP
APSVVARKVEKLLRGLQKEEDEEEEDAMSDSSSDLFELENLTAIGRYRDELPVYETTDLGTNRAIAQG
LVA
>Pda_XP_008784466.1
MRSGPEVPPEKKKGGSIRSRLRDLTKGRAPTSPGARLASFLNTLFAAVGSPKKPKIAAPPPLESACSS
ASSYSRSCLSKTPSSRGRRKPPPPPPAAEGGKRLVRFCPVSVVVGEDCRPCGRKCLHDADLAAAAVAG
PLPPPAVARRVEELLRGLEREEEEGAISDSSSDLFELENLTGIGRYRNELPLYETTDMGTNHAIARGL
AV
>Pda_XP_017697775.1
MERWPKQCGNVKPSFASALLDFIDRSIDETDGGAVREHRTGGAPDRLYDRGPAAVKKQWSFVADRWPV
ERRSNVPVASGEEKQRTRLRKPDNRRGFLANSTFISSDCSSYGGFSSSNAESVPHPAGLRSIRIGSFL
YRSEEAISKPPAPPRPVAASPPAHPHQDKKKSGLIRSKFRDLRKSKSPASLGARLASFLNSPFTAAAA
GPASDPPLAGMSFRRTRPLTLAPIAPSPTVSSWN
>Pda_XP_008785744.1
MSTVGLPNTDKSLCSRPAHRRQDSGEIDIFEAALYFAGGVDGAGLPGRIGHQRVMREDRVGWRAERKS
LDAPMSIILPQECQRVENYDAKEKKGKQPSSPGSKLASFLKSIFHQTASKKKSKSLATTKSLKDGEVE
DRHGGRNRRRSIGHSQSIRSSSDSKSIFSSESSVFSTPAPHANILTKLQKKQSRSSKSNGQPKMATFY
PQGEVLDDKRVEGESLIAERAKSSDGLSKKSKVFEGGKPDVGWNEGWFLENRWVLNGDEGKLFKKHGE
LGEVFRRKEKESREEDGGESDSSSDLFELKNYDFGELSSGLPVYGTTDMEIIKTGASIHRAAF
>Mac_Ma06_p26310
MSSSSQSFPSVMSKGYSQADSDEIDVFEATWYFSGGIDGAGLGLQRSMREERLISWGGNRSLDTLARS
TRLPQQSKKVENQRNDKKSRQPSSAGRRLASFLNSFIKQAIYRKKSRALNPTESEEDASFEKMHAGRR
KGSINDSQRMKRNDSSILCSTERCCNSKSSEHQKYAPFCSQRELGCDKRVIDEDWLVERAKSMDGYPA
NKWLTSKAGNRLLDKEALWSEEFMKKQHKWFRRTEEEDRGGSESSSELFELKNYDLGNVAFR*
>Mac_Ma04_p03330
MSSCSGSFTSGMSRSRRWNDSGELDVFEAALYFSGEVTDVDLGLQRAWRTERRGSDTPTEATLFHQPR
KVESRFKDKKHKQPCSPGGKLANLVSSFFHQAASKKKSKAVSPSQSFKEEYGERLLRRRRNSINHPEI
MTGRDYSNSIFSSDRRSGTGVSSPYPIVPLSSERENWYDKRVAGAFLLAERSKLITDGFAGNTWVEKG
SYRMLHNREAHQWAEAFIEKEDKCRRREEEEEEEDDGGSDSSSDLFELKSYDLVF
>Mac_Ma05_p08640
MTKAHRQGDSDEIEVFEATRYFSGGIGTCEGLGLQGSTREERVSWGAGRRSLDTLRAILPGRSRKADD
QCKEKKKNKQRSSPGGKLASFLISLFKQTISRKKSQFKDRRFEEMHTGRRRSSVSCSQTVRSNELDYS
NSKPSGHQIKPAHFSSHREAWYERAKSMDGYPENKWVIDGVANGQDKEELSMPTEDDGGWSESSSDLF
ELNTFHLASDPFTDLPVYGSPNSDTLERAASIAGAAS
>Mac_Ma06_p12410

MHGVDACMSSLMSGKGFSSMNHRYRERFNSWNESDELGVFEATRYFSDATDGVGLVGGFGPRGAVAVE
QTVPRSSDGRLISGSPSLTRAKKCGKQPGSPGAKLVGYLNSFFHQAASRRKPKCSNPTSTSREPREDG
EEVEKRPGDQKERRRSITGHSQSTKTAGTKSSNFCPRSEVRGRRRSITEVSKERENWENKQVPNGAWP
RGRNKTFGRTGKEVEDDDGDSDSSSDLFELKICDRGGLSDGLPVFATTDIQAIKRDTAISSSAS*
>Mac_Ma09_p15430
MERCSRSKNPSFSSSLLDAIYRSMDDEHGDKSHRRAPGRSSNHKKQAEQPTVAPSTDHCHYRPCVTEY
HRRSVPPTSSSSSSSSASSTTPTAATTSSSSVGFSSSSDVESIRSDRIPRPDLLDPGKKKKKSKCGSI
RTGLRSLRKSREPASAAVAGSSSASPGARLASFLNALFASAGSPKKPKIPIPAAASVAAAAGGGGEDS
ACSSSTSSCRRSCLSKTPTAADRRRASGADAGKRSVRFYPVSVIVDEDSQPCGHKRLQDDAGNTAPPV
AARVEELLRAAGAEVEAEEEGDGDGSESSSDLFELENLTVMMRGGRFRNELPVYETTDPHTNRAIAQG
LIH*
>Mac_Ma07_p02220
MMEGWERPPHRRSRSKNASFSSSLLDAIYRSIEDDDVDNSKSGETRGGVPDRSFSHRKQVEPVTHSRN
GSRAPVVSELAVSRAGVTDYYRRSPCGVGTFAPTSSSSSSSSSITPRINATGLFSSSDGESNQSDRI
PRLDSPATKKKAKPKCSNLRSGLRGLRKPRTASDAAVATSPRARLVRFIIALFSAAGSSRKPKITVPS
AAAAKDGARTEESTTTSTSASSCMGTCLSKASEASRRQAAGAEKGKRTVRFYPANVIVDEDYSRPCGH
KRLQDGAANAAARVKELLRLGREEDDDAGGSESSSDLFELENLTEVMGEGRGGGRCRDELPLYETTNL
NKNRAIARGLIL*
>Mac_Ma01_p15370
MEMERWGKERPRRGHENPSFSSTLLDAIYRSMDESDGCGEPNPSVAALASPRLPAPSYRSAAAVSGKA
VAPPVRLPPISTSSSSDNSSYGGFSSSSEPESASSHRARFRPIRIGVMAPDRSPSVLRPPPPPPQPAV
HHSEKTKSGSIRSNRSDPGRPKAPASPGARLGRFLNALFSAAAKNPKKSKTSTLTVAAAVHVGDPACS
TASSHSRPCLVKTPSSRRAPGADDEGVKRSVRFHPVNVIMGEDLRPCGQKSVYACDRAAGAGTEIRRR
SFATEAAKGKSRDEARRRVEELLRRFEDEEDDMSDASSDLFELENLTVMGAGGGGGGGYRHELPVYET
THPGTNRAISRGIVV*
>Mac_Ma03_p07780
MERWERAPNRRSRSKNPSFSSSLLDAIYRSIDEEHSDNNGHLRAPDRSSNHKKREGQPMPAPCIDRCH
CRSRTMSPASERAANRSSVTDYRRHGSIARGSFTLTSCSSSSSSTTTTAAASRTSSSTGFSSSSDAES
IRSDCIPRPHHPTPEKKTKKAKCGSIRSGLRGLRKSRTPDAASMAAPSPASPGARLASFLNALFASAG
SPKKPKTPTLAVATTAAAGCGDSEDSACSSSASSCRRSCLSKAPAMADRRRASGAEAGKRSVRFYPVS
VIVDEDSRPCGHKRLQDDAEGEAAPVAARVEELLRAAGADAEAEEEGEDGGSESSSDLFELENLTVVM
RGGGYRDELPVYGTTDVSTNRAISQGLIH*
>Mac_Ma10_p18560
MNHLQEKSFDSWNESDDLDVFEATRYFSGAIDGTGLQVGGFGSHGAVTVEDRVPSSARKGNLDERQIS
SSSSLMKDKKCKQPSSPGARLVGLLNSFLHQAASGRKLKCLNPTSTTREPPEEGDAKEENSLVGRERR
RSISRSQSTNSTGSKSSKFCGSSSFTNPDLYPYNYRTTPSMSYKCNRGQKSVTFCPRIEAWRSTSITE
LLHNSGVQKEKDYGLHGIRTEERDAHDRENKKWVLNDPGDQYPGRDWFGDYDLPLPGVRKNSIRGEEE
EEEEVSDSSSDLFELKICDHAVLSDGLPVFATTDIEVINRASTAS
>Mac_Ma10_p17700
MEERWARGRNRSGHQNPSFSSTLLDAIYRSIDESDGGGATHAPIIVPKRPPPLLRPPVDRRTAGAADE
RAATRRRPFVPISTSSSSEKSSYGGFSSSSEPDSAATRLRPIRTAGPPIHSAPPPPAAASFDRHEEDE
KKKKKKTSSIRGRLRDMKSSRSAAPASPGARLAGFLGSLLSAVSGTPRRPTSTASGCDDSACSTASSH
SRSCLVKKPSTRERAPPAEGGKRSVRFYPVSVIVDEDLRPCGHKSVYEADRAAETSSRRPSSAAMEAR
RRVEELLRGMEEEEEEETSDSSSDLFELENLTVIGMAGGAVHSDELPVYETTHLDTNRSFSQSQRFLQ
I
>Mac_Ma07_p11170
MGNEAWRLRHGHRSFSSSLLDAIERSMDDPIAPKSEEMANRLAHGASDFLVPLASMERRPTATVTRPR
RPDFPPFSTSSSSCNSTSSGFFSSSSGRESPATLPLRLRPSRSPPPDQHRQQDKERSGSTRTKLRGLK
KYKAPGSPGALLAGFLNSLCTAAAGDRAKPKPSPSGADSACSAVSSRARPCLSKAPSTRDPAEGGNRS
VRFRADGEDPRRCGQTKKSACGGDHAVVEARGVKMRVEELLRTLAEEGEEEQDDLFELENLMVMEGGG
YRDELPVYGTTRPEKNRSRSN*
>Mac_Ma05_p16240
MERWGKERQRRGHECPSFSSTLLDAIYRSMDESDAGGAPEPSLAASRRPAMVVSERAGSRPRGLPPIS
TSSSSDNSSYGGFSSSSEPESASSHRARLRPIRSGEAPARCTAVSSPPTRPPPPPPLQQHQRISSPPV
VHHRERTKSSSIRSKLWDLGRSKAPASPGGRLAALLNSLFASAAKRPKKSKTPTATAAGAVGGYDDSC
STLSSHSRSCLVKAPSSRRAPPAEDEGAKRSVRFHPMSVIVGEDLRPCGQKNVYAGDRAAEGRRRTVA

TEVEGKGKTKTRMRVEELLRRFEDGEEEDGEISDSSSDLFELENLTVMTGGEGYRDELPMYETTHAAT
NRAISRGLVP*
>Mac_Ma06_p13000
MEERWARGKPRSRHQNPSFSSTLLDAIYRSIDESDGGATRDRHSLITVPKRPPPPLRPAAEWRTAEAA
TRCRPLAPISTSSSSDKSSYGGFSSSSEPDSGVNRLRPIMTVGAPIRSIPPPPAAAVFDRREEEKKKK
KTGSIRGRLRDARSSRSAAPASPGARLAGFLGSVLSAVSVIPRRPTPTAVTAAGGCDDSACSTASSLS
RSCLIKKPSTREQPPSGEGEKRSVRFYPVSVIVDEDLRPCGHKSVYGADTAPRRPSAVAMKARRRVEE
LLRGMEDEEEEMSDSSSDLFELQNLTVIGRERGRGRGVGGGYGDELPVYETAHLDMNRSTSQSQRFLK
IQERM*
>Aco004841
MSSFVSRKLSPRRNSSFELDVFDAKRYFSTAIDDDASTFVGYPGRARHQKAAAAEKKSFVTSSSAPVK
GKGSCDYNKCTTKQPTSPGLRLASFINSLIQQAASKRKPKPSAPNTPTTASQKSRSNEAVYSCVSSGF
TAPCLQFNTRSYVHKEKSRLRSDTNQYRKEPEGAAYRPHAKNGIWPWKEDKYVPRGDHGLYGNVYDGD
QSVCGGERRWVLSNIHKYRDDHHGYLMKRDWSWVGEPTLRQRKSFKRSTEEVEEYCGEDWESDSSSDL
FELQIDNSDLHRHGASGGLPVLGTTNMKALKRGTAIATVAS
>Aco002602
MAAAPISPHPVPSRAMRDAFFRTNLDVPAANSLAPSTIALAVAIHVHTLYCDLISAICVISESVSADV
WFFCLGFGDGIRDWRGRPCRRCPPAAHPAITPPSPPPSSTPSNATLDTPEDSQQNPTRPGATPDEAPC
PIWWVPDAAAAANTAHREPRPRPPSWAIRHSPAPLLHPPPPPPIETKSAAPKPRSIRSRLRELRKGRG
SSASSPLASLLKVLFTSAGHVNKAKIPAPEPEPEPEPACSSARSCLSARRGSAAVTPPKRSVRFHPVS
VIVDEDSRPCGHQRLRRAPEESSDSSSDLFELENLTSSAGLRDEPPVYGTTTRFAVSLPAAPPSTAIQ
TAQFQIS*
>Aco003950
MERWKKPPPPRARRGYSCDHPSFSSTLLDAIYRSIDEPEGAGAAAKPPPPPAAAWRSATVRERDAAPR
RPQQQPQPPQQQRRCGAWPTYSTSSSSECSSYGGFSSSEAESSAPGSARLKPILPEKKKSSSIRSRIR
EFRRSSKAPASPGARLASFLNSIFAAAAGNPRKPSKRPASAAAASCGESASACSTASSYSRSCLTKTP
STRGGLPPAAPPPPPPKRSVRFGPVSVIVDEELRPYEKKLPYDXSRRRPRRGAPPRAAPSYLRTTPCS
RSSCCGTSCGSG*
>Aco009471
MEGWGKPQLQRRNGHRYGNGNGNGSGSGYGCDNPSFSSTLLDAIYRSIDESDGRGEKHRGGGGGGGAA
THRPVPVAAQKKSGSYGSGAEASDGAGTRLKPGPSTSSSSECSSYGGFSSSEAESGPGSARLRPIRTG
VRPEQAPAPPIRSPRRSAKPPASPGARIASFVASLFAAAARSPRRPKAAAAAAAPPPPQPRSCLSRTP
STRDRPRPRAPPPRGEEGAGKRSVRFHAASAAAGEDRRTCGHRRVHDGDRAAAEARRVEGLLRGFEEE
EEDRRVAEEGDFMDWSFLEALSKGERRELGSLRVGNPNRHWRRRRRRRVWGRAPCVRDHDHEARYESR
LRPPRSHFIII*
>Aco030183
MDSRVEETLKQNLIRLHITDINTGHKVRHDLNTTTWDLLKTNLSPHLPPLSLSQVFLGRLHPSSPPVA
LDFYLSLSLLASAGEDRHTCGHRRVHNGDRAAVEARRVERLLQGFEEEKEDRRGSDASSDLFELETLI
AIGGVGGGGFGDELSVYETTTTRRSTNDAIARHGLIL*
>Zma_GRMZM2G117930
MQSRRPEHGSSGELDVFGATSYFAGLPAPGGDHCRRPSSVTELCFQANKQVELDTTRSVTRHGPPQQQ
HQHQRAGWHDDHASSERHATDKLLQLQVVVAANKRRPSSASSGKSKLAALLSFMVSPSPRASFRKENN
RQEALPSPSSARMLRQAEAARGGGDGDGEEPEPASNIDGSATAASPPPPPSSSRESSAQLQGLFGALD
DDDDDEFDLGVATGDRRLQGITVVRGGGGGGEERWVVRCCVPGGGGGSAWDDEERREKAVDAESSSEQ
SIKQDEQLVVVEAEQAEQNIKDVVVEVEVEQAGDVDDVDDDGSGNVDDPAHSHSDSWDSDSSSDLFDL
DLEHR
>Zma_GRMZM2G007134
MERWRDKDKGAAAPAPGRARRYGDQPSFSSTLLNAIYKSMDEPDDGVTSSSSTAAKKQNQDLRHSCYY
YKASLAAGSYRGSSRAAAPRGPQAATTSSSSDQCSSYGGFSSSEAESSQHRRLRPIRTSLATTAVAVA
APALAPEKKKAGVSIRARLRDLRRKPASPGPGARLAGFLNAIFSGRRAPPSASSCSRSCLSETPSTRG
QPKRTVRFLDSDGGGERRRTVPVGVAPELEQMLLHRMEVDSGDDDDDESSDASSDLFDLENFAAVDPD
GGAAYSDELPVYETTRLVLGHRAIGHGYARTGGVPTRVV*
>Zma_GRMZM2G110473
MERWAPAPPSAARERPRRRPGQPSFSSTLLDAICDSLDEQAGGHGATAERATAPTPRSAKEQHQAALH
YYYYKPFLAASHRAARAAPSPADDCSSGRGYFSSSEVEYSLRRLRPIRTSAGGVGPASVAPVEKQKPA
PPGTAKRARKPSAAPASGGCRRPASPGARLASLLNAIFSGKRNSARQHPAPADEEPACSTAPSTARPC

LAKTPPSARARARATRNRSRTVRFLDIEGEVAVAAAAAGCRRFPVVEVEDSDGGEESSDASSDLFELE
NLAALAPANGGPGCRRTCENELPVYGTTGAGLGNDSRLVRRRRPFGYVSHGRSCRGLFDFK*
>Zma_GRMZM2G173732
MERWAPPSVVREKPRRRPGQPSFSSTLLDAICDSLDEQAGGHGATAAPTRGSAKKQQQAPLHYYYYK
PSLAASNRAARADDCLSGRGYFSSSEVECSLRRLRPIRTSGGAGPASVAPEEKQQLAPPGAAMRARKP
FATPASDSGNCRRPASPGARLASLLSAIFSGKRQHPAPADDEPACSTAPSSVRPCLAKTLPSARARVT
RRQSRSRTVRFLDIEGDVAVAAAAAGCMRFPVVEVKDSGGSEESSDASSDLFELESLAALAPVNGISG
CRRTFENELPVYGDDWNWAWARHCSRPSPPFGYVSHGRS*
>Zma_GRMZM2G178852
MERWGDKDRGAAVPAPGRLRRYADQPSFSSSLLDAIYKSMDEPGDGATSAAAAGATKMQSHQDLHYSY
YYKTSLAGSYRGSRAAAAAHAATTTSSSSECSSYGGFSSSEAESSQHRRLRPIRTSVGAAASPAPAPE
KKKKAGANIRAKLRDLRKPASPGARLAGFLNTIFSGRRAPATPPSRGAESSACSTASSYSRSCLSKTP
STRGQPKRTVRFLDSDDGEAAAAAPGGERRRVQVGVAELERMLLHRMEMDSDEDDEDEEGSDASSDLF
DLENFAAGAPDAAAAYRDELPVYETTRVVLGHRAIGHGRSARVV*
>Zma_GRMZM2G438606.1
MERPWRDKDKGAAAPAAGRARRYGDQPSFSSTLLDAIYRSMDEPDDGVTSSSSTAAKKQNQDLRHSCY
YYKASLAAGSYRGSSRAAAPRGPQAATTSSSSDQCSSYGGFSSSEAESSQHRRLRPIRTSLATTAVAV
AAPALAPEKKKAGVSIRARLRDLRRKPASPGPGARLAGFLNAIFSGRRAPPSASSCSRSCLSETPSTR
GQPKRTVRFLDSDGGGERRRTVPVGVAPELEQMLLHRMEVDSGDDDDDESSDASSDLFDLENFAAVDP
DGGAAYSDELPVYETTRLVLGHRAIGHGYARTGGVPTRVV*
>Zma_GRMZM2G438606.2
MERPWRDKDKGAAAPAAGRARRYGDQPSFSSTLLDAIYRSMDEPDDGVTSSSSTAAKKQNQDLRHSCY
YYKASLAAGSYRGSSRAAAPRGPQAATTSSSSDQCSSYGGFSSSEDGAGRGGAGAGADAAPPDGGGQR
RRRRRREQRRQLRSVRPREFRGR*
>Zma_GRMZM2G027519_T01
MERRGHCHGGKPPHPHPPPRRVRTRQPSSGSFSASLLDAIYRSLDDGDGADVVVDAARGSVEEKAAAT
ATAQFWWLNKAAAPKPCRQSSSTADRDRRRREAGVARPRHSGSGYASSTASSSDSSAASYSSLSCSSA
STVGIESTCRRHGLPPPRVSLSEESVATDAEETTPPPPNSKPKKKARPCFPVARIRPRASVPPPSSGP
QPPSSPATFACALKALFSSARLQRKPKAPAPARTTPPPKISHPPRMSTTSAAKAADAPQPSEPTTVRL
HPEAEASVVRRRVEELVRGLEELEEDEERSDASSDLFELESLRGAGADELPVYGTTSLVANRAIAQGP
GGQLVNK
>Zma_GRMZM5G843781_T01
MERRGHCHGGKPPHPHPPPRRVRTRQPSSGSFSASLLDAIYRSLDDGDGADVVVDAARGSVEEKAAAT
ATAQFWWLNKAAAPKPCRQSSSTADRDRRRREAGVARPRHSGSGYASSTASSSDSSAASYSSLSCSSA
STVGIESTCRRHGLPPPRVSLSEESVATDAEETTPPPPNSKPKKKARPCFPVARIRPRASVPPPSSGP
QPPSSPATFACALKALFSSARLQRKPKAPAPARTTPPPKISHPPRMSTTSAAKAADAPQPSEPTTVRL
HPEAEASVVRRRVEELVRGLEELEEDEERSDASSDLFELESLRGAGADELPVYGTTSLVANRAIAQGP
GGQLVNK
>Zma_GRMZM2G088860_T01
MERRGNCHGGKHPPPRQARGPGERTRQPSSGGSFSASLLDAVYRSLDDGDGADVVVDAARGSGAEEKA
AATAQFWWANKEAATARPRQSSSSADGDRRRRETGAARPRHSGYASSTTSSSDSSASYSSFSCSSAST
TDTESTCRRHSPPPPRMSLSEESAATDAEEATATPPPPPKSKPKKKARPCFPVARIRPPKASVPSSSS
SGAQPPSPATFACALKALFSSVRLQRKPKAPAATPPPKISQPQPQPQPPSMSATSTAKAADAPAEPSV
QRTVRLRPEAEVSVVRRRVVEELVRSLEELEADEEGSDASSDLFELDSLRGAGADELPVYGTTSLVVA
NRAIAQGPAR
>Zma_GRMZM5G886335_T01
MPPHDGARAPPPRPSRGHQPSFSAALLDAIYHSLEADAEARTSTEARRTRTPASSPAQLPSRRRPTPE
LSPSPSPSPSRSSVRSPRLQRAPRPCRVRPDPQPNSSGSLLLPPPLPPHPHEHEPSSTGHRRVADAERKR
GRGRKSKRTAPFACLLNALLCNRRPARPVDRTPRATATPTPTPATAAPEPASARSILSSRASRSRRES
AAAGGVLAPARRAVRFSPVATVVGDGHGHGAGSVGTATTGLRAKESAAEAERRVEELLRALGVADERE
RAKESTESSSDLFELDSLPAFQDRGTDLPRSRTVADGDDGAGLLARPRPRVQ
>Sbi_Sobic.001G337500
MMMQSRQREHGSSGELDVFGATSYFAGLPPPAPDDADCRRPSSATTEPSADRLYFQATKVVVHPDTRT
KTMEDNSFRGPHQQQQQPGLHGHADRHDTNKQLQVVAAKRQQPPYSGKSKLAALLSFMVSPSPSPRAS
FRKEINKQEAPSTTTTTRLLRQAEAAPAAGGERETAAANNTYKAAANSPSSSSRERSSSMQLHGLFGA

HDDDDDDDEELDLGVATGDRRLQGITVVRGIGGGEERWVVRCCVPVPGGGAWDDEEHREKMLLDAAESA
SSEQNVKDELLVEVEQLQGDDDDDGNVVDPAPSSWDSDSSSDLFDLDLEYR
>Sbi_Sobic.001G257100
MERWPPVPAPERPRRRPGQPSFSSTLLDAICDSLDEQAGGQGAAAERAAEAPTPGSAKKQQQAALHYY
YYYKPSLAASHRAARAAPAPADDCSSGRGYFSSSEVEYSLRRLRPIRTSGGGVGPASVAPAEKQQAVP
PGSATARRARKPSAAPASHGGCRRPASPGARLASLLNAIFSGKRHSARQHPAPADDEPTACSTAPSSA
RPCLAKTPPSATARARATHSRRSRTVRFLDIEGEVAVAAAAAGCRRFPVVEVEGSDGGEESSDASSDL
FELENLAALAPANGGSGCRRTCEDELPVYGTTGAGLAQDIGLVRRRPFGYVSHGRSCRGLFDFK*
>Sbi_Sobic.001G485400
MERWGDKDKRAAGAAPGRARRYADQPSFSSTLLDAIYKSMDEPDDGVTSSGAATAAATKKQNHDLHYS
YYYKASLAGSYRGSSRAAAPGPHAATTSSSSECSSYGGFSSSEAESSQHRRLRPIRTSAAAGAAATAP
APALAPEQKKKAAKKAGANIRAKLRELRKPASPGASPGARLAGFLNAIFNGRRAPQTPPSASASRGAA
AESACSTASSYSRSCLSKTPSTRGQPKRTVRFLDSDDGGEAAAPAPGVERRRVQVGVAELEQMLLHRM
EMDSDEEDDEEDEESSDASSDLFDLENFAAVAPDAGAAAAAYRDELPVYETTRVVLGHRAIGHGYAHG
RSTRVV*
>Sbi_Sobic.004G316600
MPPHDDDGARVPPPRPSRGHQPSFSAALLDAIYHSLEADGEARLSTEARRTRTPASSSSPARRTPLPS
RRRPTPEQSPSRSSVRSPRLQKTPRPCRVRPDPQPILLPPPLPPPHPQESSTGDRRAADAEKKRGRRK
KSKGTAKAAPFACLLNALLCNRRPARARSVDHTPRATAAASEPASARSILSSRASRMESAAAGGILTP
ARRAVRFSPVATVVGDDHGHLTAPTGLRDTGAEMARANKESAAEAERKVEELLRALGVADERDRAKES
SESSSDLFELESLPAFEDRDTEMRRSRTAAGDGAGLLARPRPRVAV*
>Sbi_Sobic.002G218300
MERRGHCHGGKHPHPHPHPPSLPPPRRARGGERTRQPSSGSFSASLLDAIYRSLDDGGGTDAVVVDAA
RGSGAEENKAAATAQFWWANNKEMAANKPRQSSSSSADRDRDSRRRRAETGVARPRHSGYASSTTSSS
DSSAASYSSFSCSSASTTDTESSTCRRHSPPLPPRVSSSEESVATDAEEGATPPPPPPKSKPKKKSRP
CFPVARIRPKASVPASSSGPQPPSPATFACALKALFSSARLQRKSKTPAATPQPKTSHSQPQPQPPRM
SATTSAAKAADAAAEPSEPRTVRLRPEAEASVVVRRRVEELVRSLEELEEDEEGSDASSDLFELESLR
GAGADELPVYGTTSLVANRAIAQGPAR
>Bdi_Bradi1g72920
MERRGEKGAAAARARRPQGAAEQPSFSSTLLDAIYKSMDEPGHDAVSKKKQQEKEEALHYSYYYRPSL
AGSYRARAPGPAHATTTTSSSSDCSSYGGFSSSEAETSSGRHHHRRLRPIRTAAAAPAPPAPEKKASK
KQQVAPGASIRAKLRDLRKAPASPGARLAGFLNTIFAGGGGKRAPQTPPSASAAAEYACSTASSAASY
SRSCLSKTPSTRGGGGQQQAGRTVRFVDSAAEAPATVPGRRMPARAVEQMLLRRMEMESDEEDEESSD
ASSDLFELENFTAAPPGAAGDELPVYETTRVVLNRGSIGGHHGYGHGRSARVV
>Bdi_Bradi4g31530
MEKGRRSHGHGKRPPPLAPPRRERAAAASFSASLLDTIYRSLDEGSDADATAADVADTPRRSEENAPA
PAQFWWAKEAGGKPNMRRLETGPARRRHSGYASSTASSSDSASSSYSFSCSSASTTDTESTARRRRSP
PPPPRRQPEEVRADAAEAEPPSPPNNKAKTKKGRPCFPGARLRPRDASGPSSPAGGPLPPPSPGSFAC
VLRTLFTSGRLPRKQPKTPISRGPQTPQRTSPEPAETPRASATSSERRSVRFCSDAEASSVVRRRVEE
LVRSLGELEENDEGSDSSSDLFELESLGGANGDELPVYGTTSLVANRAIAHQAVF
>Osa_Os03g07920/BG1
MERWAAPKVTAGSARRYVADQPSFSSTLLDAIYKSMDEQPGHGGGATGVEAVAAAAKKQHEAALHYGN
YYKPSLAGSYRARAPGPHATTSSSSECSSYGGFSSSEAESSHHRRLRPIRTTVPGGAPGPAPEKKAKK
PGASIRAKLRDLRKPASPGARLAGFLNSIFAGKRAPATPPSATAGAESACSTASSYSRSCLSKTPSTR
GQAKRTVRFLDSDTESLASSTVVDRRRVPVEAVQQMLLQRMEMESDEDDDESSDASSDLFELENFAAI
APAGAAYRDELPVYETTRVALNRAIGHGYGHGRSARVV
>Osa_Os10g25810
MRDMEMRWAAPAPAARGRGRARRRAPDQPSFSSTLLDAICDSMDEGGEDGRTRNAASAAAKKRQEAAN
SYHYYYCYKPSLAASYRAAPALGSTADCPGRGYFSSSEVEYSLRRLRPIRTSAAGGAGDGAAVARKQR
HEQPDVEKTAKTKPGSASARACRRPASPGARLASLLNSIFSGKRPSAQRPACSPDYPEPACSTAPPSS
SSSYARRPCHAKTPRTPPTTTTARARPSRSRTVRFLDIDGKVAVAAAVAGCRRIPVMEVEADTDDGG
EESSDASSDLFELDSLAAIAPAGGRDGSYGDELPVYGTTGVGIRRDIGRRRPYGHAPCRSWSRAV
>Osa_Os03g30120
MHRRRQHGSSGELDVFGATRYFAGVATAARPIAVVVVREPEDMIIQVKTTTTTSSDKKTTEKEGHHHA
GQLDVVGVAKTTHRSKLAAFLGSLVSPESTSFRKKPPPAASSETTTYNYNDDDNLPKMQVPSSSSTSS

GRASIDVAAAAATVHGGGGGRHDDDDLGVDAMWEDRRLQGVRVVRCGRCDEERWVVRCGACCAWEEEE
EEHHHGHEKKAILAAAATSTRYGSHQVLAGDREVVGDGACSDWESDSSSDLFELDLEIT
>Osa_Os02g53660
MSPHHSGRLAPPRRNRENPSFSAALLDAIYHSLDADGSLPASPADAEGSPVPGRRRRPSQCNNLSPSA
SSVRSPRLQKTPRPCRVRPDPQPSLLLPPPQPPPPMPESTGDVAEKKRGRRKNKNGAKSAPFACLLNA
LLCNRRSARSAEPTTPRALAVAPAAVAVTAAEPASARSILSSRASRRQPAATGGILTPARRAVRFSPV
AVVVDDGEHGCRDAGVARLRGAEREVAAAQESAAEAERRVEELLRALGVAEESERAKESSESSSDLFE
LESLPAFDDAELPR
>Osa_Os09g27160
MGHVTVHSWSHSISLRVCLVQRRQCHGKNAPPTLAPPRRARGGAGGSFSASLLDAIYRSLDEGGGGDG
AGAVVDDARRSEAEEMKAAAAAAVPPQFWWAKSKQAAGAAGRSRRESVARPRHSGYASSTASSSDASS
SSYSSFTCSSASTTDTESTTHRRRHSQPPPQQPEDVDAAAAAAAAAPPNSKPKKKKKKSRPCFPGARL
RPRGTVPPPPPSSSGPSPATFACVVKALFSSSRLPRKPKAPTAVPLPPASPPVPQPPCMSAAATTTSN
TKASERRSVRFCPGAETSVVRRRVEELVRSLADVEEDEDGSDASSDLFELESLRGADGDELPVYGTTS
LATNRAIILRREQLASS

Table S2. The CDS of *BG* and *BGL* genes used for the phylogenetic analysis

>Rco_29706.t000031
ATGGATCACCACCAAAGAGAAAATCCGTCTTTCTCTTCAACTCTTCTTGATGCAATTTACCGTTCGAT
AGACGAATCAAACGGTAAAGGAGAAGAAGAGT
TGATTTTGTATAGAGAAACTATGAGGAAGAAGCACAGCAGTAATGGTTTCAAAGATGGAGCTGCAGTT
AAAGAAGAAAGAAAAACAAGTTTGAAAAAAGC
ATGCATGATTGAGAAATGGATGGAGGAGAAAGTAAGCTACGAAAAAGTTGCTATCCGGAGAAAATCCA
TGGCTGATTTCGACAAAATAAATACTCGAAAG
GATTTCACTCATCGTCCTGTGTTGCTTAACTCGAGTTCTACTTCTTCAGAGTCGAGCTCTGGTGGTGG
GTTTTCTTCTTCAGAATCTGAATCAGTTTATG
GTCTGAGTTCAAGATCATCGTCAACTAATTACACCATGCAAAGGCCAAAGCCAATTAGAACAAGTGCT
TCTGCTCGGCCTGTTGATGAACTTGGTATGTA
CAATCATCATCACCCCACAACACAAAAGCCTAATAATAAGCATCATGAAGGCAGTTTTGTCAAGACAA
AATCCAAAGCCTTGAAAATCTATGGTGATCTC
AAGAAAGTCAAGCAGCCTATTTCCCCTGGTGGCCGTCTTGCAAGCTTTCTCAACTCTCTTTTCACTGC
CGGAAATGCGAAGAAGGCGAAGATATCTTCGC
CGTCAGGGTATGAGGAAAGGACCAAGTTCAAGTCCGAGCAGACATCAACATGTTCTTCAGCTTCTTCA
TTCTCAAGGTCTTGTCTAAGCAAGACTCCTTC
CTCCAGAGGAAATGGAACTAAAAGATCGGTAAGGTTTTATCCGGTTAGTGTAATTGTTGGTGAGGATT
CCAGGCCTTGTGGGCACAAAACTTTATATGGC
ACTGATCAAGAGAATCCTAGTCTGATGACAATCACTACAACAAGAGTTCCTACCAATGAAGAGCTCAA
GTTTCATGTAATGAATGAAAGCCGCCGAGTTG
AGGAGGTTGCAAGAGATCTTTTAAGGAATTTTCAAAAGAAGAAGCAAGAAGAGTTCGATGTTAGCAAT
GGCAATGTTCCACGATACCAAGAATTAGAAGA
AGATGACGATAATGAAGAAGAAGAAGATGATGATGATGCAGCAAGTTATGCAAGTTCTGACTTGTTTG
AATTAGATAATCTTTCTGCTATTGGGATTGAA
AGGTATCGAGAAGAATTGCCTGTGTATGAAACAACTCATCTCGATACTAATCGAGCCATTGCCAATGG
CTTACTTCTGTAA
>Rco_29739.t000035
ATGCATAAGTGCGAGAAAGAAATGAGGGAAGATCATAGACACAAGTACGAGGGCAAGAACCCATCTTT
CTCTTCAAGTCTTCTTGATGAAATCTACCGTT
CTATTTGTGAGGGTGACACAAAACACGATGATTTGAAATTCTACAGAGAAACAATGCCAAAGAAGCAA
AACAAAGATACTAGAGCCATTCGCGGAGAGAA
AGAAGCAGATGAAGTGATGGCGTCTCTTCGTCGTGCTTGTTTGATCGAAAAATGGATGGAGCAGAAGG
TTAGCCAAAAGGTCATCGGCACGCAACATAAG
AAACAAAACTCGACAGTTTTCGAGAGAAAATCGCAACACGACCATGATATTGACCAAGATGTTCTCTT
CTTTAGCTCAACTTCTAGCTCCTCGGATTCTA

GCTTCGGAGGATTCTCGTCCTCCGATACAGAATCCATCTACGGTGCAAGATCAGGAGGCTATTCTTTC
GCTCCAGCAAGGCCTAAGCCTGTCAGGACAAG
CGTCTCAGCTCGATCAGGAAAAACAGAGAGGACTCTGTTTTATGAACAGAGAGAGTTGCACATGTTTG
ATGACTATCACTGCAGTTCTGCTTTTTCAGAG
CAGAACACTCCAAGGCTTGAAGAGAATATAATTAAGTCTAAATCAAGAGCTTTAAAGATTTACAACAA
TTTAAAGAAGGTGAAACAGCCAATTTCACCAG
GTGGTAAGCTTGCAAACTTTATCAATTCTCTTTTCACCACAGGAAACACAAAGAAATCAAAGAATTCG
TCTGCTTCTTCAATTGGGAATTTTGTTGATGA
GAGGAAATTTAAGTCAGCACAAGCATCGTCAACTTGTTCTTCAGCTTCATCATTTTCAAGATCATGTT
TAAGCAAGAATTCACCATCCACAAGGGAAAAA
TTAAGAAACGGGGTTAAAAGAAATGTCACATTTTACCCAGTGAGTGTAATTGTTGACGAAGATTGTAG
ACCATGCGGACACAAAAGCTTATATGAAGAAG
AAGAAGAAGAGGAAGAAGAATCATCTGCTGTAATGTCAGTTTCTCTGCCAAGAGCATGGAAAATCGGG
AAATCGCCATCAAGAAAAGTTGATGATGAGTT
AAAATATCAAGTAATTGAGAAGAGTAGAAGAGCGGAGGATGTGGCAAGAGAGTTTCTGAAAGATTATC
ATCAAAGTCAGAAGAAAATGAATAATGATGAT
GTAATAATGAGGGCGAGGGATGTTCGCCGCAATTACAATGATCATTTCGAGGATGAAGATGAAGATAA
CGATAACGATAACGATGATGCAAGTTGTTCGA
GTTCGGATTTATTCGAGCTCGATCATCTTTCAGTAATCGGTAAAGATAGGTATTGTCAGGAGCTTCCT
GTATATGAAACTACTCATGTCAGTACTAATCG
TGCCATTGCTAATGGCTTAATAATGTAG
>Rco_29912.t000009
ATGTATAAGAAAGAGAGCTCTTCTTCTAGGGAAAACCCGCTTCCTCAGAGAAAAAGAACTCCGTCTTT
CTCTTCATCTTTACTAGATGCCATTTATCGTT
CCATTGATGAATCTAACGGCGGCGCTGGCTGCGGCGAAGAGGAAGTTTTGAGTCAGCAGTACCAAGAA
ACTACCGTGATCAAGAAACAGAGCACCCGCAC
CCAATCTGTTTCTACAATTCGGCGCGATACTTGTCTTGAACAAGAAAAAGATTTATCTACTCTTCGTC
GAGCTATTCTGTTAGAAAGCTGGATGGAGAAG
CAAACTCACTCTAATTCGACTTCTACTTCTTCAGAGTCTAGCTCTGGAGTTGGAGGAGGCAGAGGCGT
ATTCTCATCATCCTCAGAAACTGAATCAAGCG
TTGAAAATTCAAGAAGAACAACAACACAGCAAAGAAGCAAGCAAGTTTCTGATAAGCAACAGAAGCCG
AAATGCGAAGGAGGGTTTTCCAGGACAAAGCT
AAAAGCAATGAAAATATATGGAGAACTAAAGAAAGTCAAGCAACCAATTTCTCCAGGCGGCAGAATTG
CTAGTTTCCTGAATTCTATTTTCAGCCCGGGA
AGTGCAAAGAAAGTAAAAATGTGTTCTATTGGAGCTATGGATGATGTCAGTACTGCCACAGACCGGAA
ATCAAAGTCTGCTTGTTCTTCGGTTACATCTT
TTTCACGTTCTTGTTTAAGCAAAACTCCGCCTTCAAGAGGGAAAGCGAGCAATGGCAATAAAAGCAAA
AGGTCAGTTAGATTCTGTCCAGTTAGTGTCAT
TGTTGATGAGGATTCAAGACCCTGTGGCCATAAATGTATCTATGAAGACGATCCAGGCTTGATGCCAA
CGCCTGTCCCTCCAAAACTCGTTAAGAGTTCT
TCTTTCAAAGAAGATGCTGGTAAAGGTGCAAAGTATATCAGAAATTATCAGAAGAAGAATATCAGTGA
ATTTGATTTCAGGGGATTTCATAGTTATATTC
AGGATCGTGATGCTGTTGATGATGAAGACAGTGATGAAGATGAGGACAATCAAAGTTGTTCAAGTTCT
GATCTTTTTGAGTTAGATCATCTTATGGGGAT
TGGAAGGTATAGAGAAGAATTACCAGTTTATGAAACTACAAATTTTAAAAGAAATCAAGCCATTGCGA
ATGGCTTCATTCTGTAG
>Rco_30174.t000478
ATGTCCATTACAGGACTCCCAGATACATCGAAACTTTACAAGAAATCGTTACATCGCAGAAATGATTC
TGATGAGCTTGATGTGTTTGAGGCAGCAAGGT
ATTTCTCAGGATATAATGAAGCTGCAGGATATAATGGTGGAACATATACACAGAAAATCCTGAGAGAT
GATTATAGACATCCTTGGAGAGGAGGAAGAAT
GAGCTTAGACGTCCCAATGAGAAATCCACTGCCCCAGCAGACTCATAGTCATCATCATACAGTAGAAA
AGCAAATATTGAAAGAAAAGAAATACAAGCAA
CCAAGCTCACCAGGAGGTAGACTAGCTAGTTTTTTGAATTCTCTTTTCAATCAAACAAGCTCCAAAAA
GAAGAAATCAAAATCCGCTACACAGTCAACGA

AAGATGATGATGAAAGTCCTGGTGGAAGAAGGAAAAGGAGGAGCAGTATCAGTCATTTCAGAAGCACA
AGTACTGCTGATACCAAATCTTTATATTCTTC
TTCAAGTTCTGGTTTTAGAACACCCCCTCCATATGCAAACACTCCTACAAAGAGCTACAAGGACCTCA
GAAGCTACTCGGATCACAAACAAGTAATTTCA
TTGTCGATGCAAAATGGGAATGTGAAGTCCACAGGCTTACAGAATGAGGTATTGGACGAGAAAAAGAA
GACAGACTTATCTTGGTTGGATGAGAAATTTA
AAATCAGTGACGCATTATCTGAGAAAACCAAGAACCTTGGCAATCATCGATATTTAGAGAAAGACAGG
ATTTGGGTTGATCAGTATCCATCGGAAGAGAA
AGGATTCAGAAAGTTCGATGAGGTAGATGATGGAGCTGACAGTGATTCAAGTTCTGATTTATTTGAGT
TGCAAAACTATGACTTAGGCATCTATTCAAGT
GGTCTGCCTGTGTATGAGACAACAAATATGGATAGCATCAAAAAGGGAGCACCAATTTCCAATGGCAC
TCTCTGA
>Mdo_MDP0000125138
ATGTATGTGAGGGAGAGGTTACCGAAAGAAGAAACGTTTTTGAGGCGAAGAAGAAACCCATCTTTCTC
TTCTTCGCTACTCGACTCCATCTACCGCTCCA
TTGATGAATCGAGCGGTGGAGATGGAGATCAGGGTTATGTTCGAGAGTCGACTGCAATGGTCAGGAAA
CAGAGCAGTTCTACTAAAGGAGACAATGAGAA
GGTTAATCTTCGAAGGGCTATTATGATTGAGAATTGGGTCGAGAAGCAGAGCGTTCACAGCTCCATGT
TCTCGAACTCCGCTTCGAGTTCCTCGGAGTCG
AGCTCGGGAGCTGCATTTTCGTCTTCCGAAACAGATTCGAGCTACAGATCAAGAACAAAACCGAAGGC
GGTCGAGCAGAGGTTCGTGCAGTTCGAGGAGA
AGGAAAAGATTGAGAGTGGCGGAGGAAGTGCGTTTTCAAGGACGAAGCTACGAGCTTTGAAAATCTAT
GAAGAATTGAAGAAAGTGAAGCAACCAATTTC
GCCCGGCGGGCGGATTGTGAGCTTCATCAACTCGATTTTCAATTCCGGCAATGTCAAGAAGCCAAAAA
TGTGTTATGTTGGAGCTGTGGAAGATGTGACC
ATTTCAGAGAATGTGTCGAATTCGAAATCGGCTTGTCCTTCTTCTTCTGCTTCTGCTTCTACTTTCTC
AAGGTCTTGCTTGAGCAAACCGTCTTCAAGAG
CGAAAAAAATCGAGCAATGGCACTAAAAGGTCCGTTAGATTTTACCCAGTGAGTGTGATTCTTGGTGAG
GATTCTCAGCCTCCAAACCACCTCAAATGTGT
GTTTGAAGAAGACCCAAGCTTGATGCCAAAACCCTCTTTTCAAAAATATGCAAGGGCTTGTCCTGGTA
ATTATGATAAGCTGATCCAATCGGGTAAGAGW
CGAACAGAAGACCTGCTCACATTTAATCGTAGCACAAAATCAACAAGTTACAGAACGACCGGGGCGGT
TAGTCAAAATTTGGTGAGAAGTTTTTGCGACA
ATGCAGATGATGAAGAAAGTGATCATGATGCTGAGAGCTGTTCAAGTTCTGATCTTTTTGAGCTGAAT
CATCCAGTTGGGGTTGGAAGGTACATGGAAGA
ACTTCCTGTGTATGAGACTACTAATTTCAGAACCAATCAAGCTATTGCCCAAGGGTTKTTGTAA
>Mtr_Medtr4g125620
ATGCATACTAGAGAGAGAGATCTTATCCACAAAGGAGAAGAACTCCTTCTTTCTCCTCCATTCTTCTTGA
CACCATTGATCATTCTATCGACCAATCCAAAA
CCGATTTTGTTGATGATGTAAACAACCAACCATGTCTCTACAATCAACCCATAAAACATGTCAAATTC
AATGAAAAATGTGTCAATTCCAAGCAGAGGAT
GAATCTTCGTCAAGCTGTGATGATAGAAGATTGGATGGAGAAAAACTCGAGTTCAAGCTCCTCAGAAT
GTAGCTCTGGTGGAATATTTTCATCCTCAGAC
ACAGATTCATCTTACAACAGACAAAGATCAAGAACCAAATACAATCCACCCCAGAAACATATGAATCC
AATTCATCATAGCAATTCAGAGAAACAACAGA
AGAAGAAGCAACAAGTTTGGGAAGATGGTTTTACAAGAACAAAGTTGAGAGCTTTGAAAATCTATGGT
GAATTGAATCAAAAAGTGAAACAACCCATTTC
ACCTGGTAGTAGAATAGCTACATTTCTTAGCTCCATTTTCAATTCTCACAATGTGAAGAAAGCTAAAA
TGTGTTACGTTGGTGCTGTTGAAGATGTTAGT
TTTGATCACAAATCAAAATCACCTTGTTTTTCTTCCTCAGTTTCTTCATATTCTAGAAGGTCTTGCAT
GAGTAAAACAACTTCAAAGTCCAACAATGGTG
TTAAAAGATCGGTTAGATTCTATCCTGTTAGCGTAATCCTAGGTGAGGATTCTGAACAACAACATCAA
CCTAGTACTATTCGAAAGATTACGAGAAATTC
TTCGGTTAATGAGCTGAAGAATATTAACAAGGTTGTTATGGCTAAAGAAAGATTTTATGATAACGGTG
AAGATGACGATGAAGATGAAGATGATGATGCT

TTGAGTTGTTCGAGTTCGGATTTGTTTGAGCTTGATCATCTTGTTGGAGGTGGAAGGTATCAAGAAGA
GCTTCCAGTTTATGAAACTACCAATTTAGAAA
TCAATAAGGCCATTGCTAATGGTTTGTGTGTTAATTTGTAG
>Ptr_Potri.001G170500
ATGGATAGATGGGAGAAACCACTAAGAGATGATAGGTATAGACACCAAAGGCAGAACCCATCTTTCTC
CTCTACTCTTCTTGATGTTATCTACCGTTCCA
TAGATGAGTCTGGCAATGGCAAAGGAGAAGAAGAGCAGCTGATTTTCTACAGAGAAACCATGAGAAAG
AAACATGAGATCAATCATGGCTTTAAAGGTGA
AGAAATGACAAGTTTGCAAAGAGCATGCATGATAGAGAACTCGAGTTCCTCAGAATCTAGCTGTGGTG
GTGTTTTTTCATCTTCAGAATCCGAATCAATT
TATGGTGTCAACTCATCAAGATCATCAACAACTAGTTACACCATGCAAAGGCCTAAGCCTGTCAGAAC
CAGCATTTCTGCTAGGCCAGAGAAATACCAAA
GACGTGAAGATCTTCATCAAACCGATACGTTTCAACATCATGAGAGGAATTACGCACCAAACCAGAAG
GCAAAACCTGAAGGCAGTTTTGTCAAGACAAA
ATCAAAGGCCTTAAAGATTTATGGTGATCTCAAGAAGGTCAAGCAGCCTATATCGCCTGGCCGCAGGC
TTGCGAGCTTTCTTAACTCTCTTTTCACTACA
GGCAATGCAAAGAAGGCCAAGATTACGACGCCGGGTGGAAGTTACGAAGAGCGGAAGTTAAAGTCTGA
GCAAGCATCTACATGCTCTTCAGCCTCTTCAT
TTTCAAGGTCTTGCTTGAGCAAAACCCCTTCTTCAAGAGGTGGGAAATTGAGTAGCAACAATGGAGCC
AAAAGGTCAGTGAGGTTCTATCCTGTTAGTGT
GATTGTTGATGAAGATTGTAGGCCTTGTGGGCATAAAAATCTATATGGAAGTGATCGTCAAGAGATGA
GTAAGCTTAAGTTGCATGTCATGAATGAAAAT
CGCCGAATCGAGGAAGTTGCAAGGGATCTCTTAAAGAACTATCAAAAGAAGAAGGAAGAGCATGAGGA
AGAGGAAGAAGAATCTGATGACGATGATGATA
TAGCAAGTTGTGCAAGTTCTGATTTGTTTGAATTAGATAATCTTTCTGTTGTTGGAATAGAGAGGTAT
AGAGAAGAATTGCCTGTGTATGAAACAACTCA
TCTGGGTACTAATCGAGCCATTGCTACTGGCTTATTCCTGTAA
>Ptr_Potri.003G063400
ATGGATAGATGGGAGAAACCTCTAAGAGATGATAGGTATAGACACCAAAGGGAGAACCCATCTTTCTC
CTCTACTCTTCTTGATGCTATCTACCGTTCCA
TAGATGAAAATGGAAGTGGTAAAGGAGAGGAGGAGCAGCTGATTTACTTCAGAGAAACCATGAGAAAG
AAGCATGAGAATAATGGTATTAAAGATGGAGA
AATGACAAGTTTGCAAAGAGCATGCATGATAGACAAGTGGATGGAGAAGAAGGTTAGCCATGAGAAGG
TTGCTGTAAGGCGAAAATCCATGGCGGATTTT
GAGAATAAATCTCGAAAAGATGTAGATTCTGTGCTGTTAAATTCAAGCTCGACTTCCTCAGAATCTAG
CTGTGGTGGTGGTTTTTCATCATCAGAATCTG
AATCAATTTATGGTGTCAACTCATCAAGATCATCAACAACAAGTTACACCATGGAAAGGCCTAAGCCT
ATCAGAACCAGCATTTCTGCTAGGCCAGAGAA
ATACCAAAGACGTGAAGATCATCATCAAATTGATATGTATCATAATCATGAGAGAAATTATGCACCAA
ACCAGAAGGCAAAACATGAAGGCAGTTTTGTC
AAGACAAAATCGAAGGCCTTAAAGATTTATGGTGATCTCAAGAAGGTCAAACAGCCTATATCACCCGG
CGGCCGGCTTGCAAGCTTTCTCAACTCTCTTT
TCACTGCAGGCAATGCAAAGAAGGCCAAGATTTCGACTTCAGGAGGCAGGTATGAGGAGAGGAAGTTA
AAATCTGAGCAAGCATCTACATGCTCTTCAGC
TTCTTCATTTTCAAGGTCTTGCTTGAGCAAAACACCTTCTTCAAGGGGAGGGAAATTGAGCAGCAACA
ACGGAGCCAAAAGGTCAGTGAGGTTCTATCCT
GTTAGTGTGATTGTTGACGAGGATTGTAGGCCTTGTGGGCATAAAAGTCTATATGGAGGTGATTGTCA
AGAATTGAGTAGTACTCTAGTGGCAACTACAG
TTACAGCAGACGCAAGAAATAATGTTCCCACAAGTGGTGAAGAGCTGAAGTTCCATGTAACGAATGAA
AATCGCCGAATCGAGGAAGTTGCAAGGAATCT
TTTAAAGAACTATCAAAGGAAGAAGGAAGAGCAATTCGATCATATGAGCACCGATCTTTGCAATGACA
ATAATCATGAAGTCATGTCTAGTGATGAGGAA
GAGGAAGAAGAATCTGATGTAGCAAGCTGTGCAAGTTCCGATTTGTTTGAATTAGATAATCTTTCTGC
TATTGGAATCGAAAGGTATAGAGAAGAACTGC
CCGTGTATGAAACAACTCATCTTGGTACTAATCGAGCCATTGCTAATGGTTTAATCCTGTAA
>Ptr_Potri.006G144700

ATGTACAAGAAGGAGAGATCTTCAAGGGAAAGCACATTCCATCCAAGAAGAAGAACCCCGTCTTTCTC
TTCAACTCTTCTTGACTCTATTTACCGTTCGA
TTGACGAATCCAACGGTGAAGAACAGCATGTGCTGGGGATCAAGAAACAGAGTTGCAACTCTGTTTCT
ACAACCAGGCGTGATACCTCTTCAGATTCTAG
CTCTGCAGGAGGAGGAGGAAGCGGAGGAGGTGTGTTCTCGTCCTCCGAAAACGAGTCAAGTGTCAGAG
GAAACTCAAGTTCTTGCCAACAGAGAACAAAA
CCACTTTCAGACAAACCACATCAGAAACCGAAATGTGAAGGAGGAGGGTTTCATAAGACGAAGCTAAG
AGCATTAAAAATCTACGGTGAATTAAAGAAGG
TAAAGCAGCCGATTTCACCAGGTGGTCGCATTGCAAGCTTTCTAAATTCTATTTTCAATTCAGCAAGT
GCTGCAAAAAAAGTGAAAATGTGCTCTATTGG
GGCCATGGATGATGTAAGTTTTGAGCGTAAATCAAAGTCTGCTTGCTCATCGGCTACTTCCTTTTCAA
GGTCCTGTTTGAGCAAAACACCTCCTCCAAGA
GGGAAACCAAGTAATGGCACAAAAAGGTCAGTTCGATTCTATCCTGTTGGTGTCATTGTTGATGAGGA
TTCAAGACCTTGTGGGCATAAAAGTATTTATG
AAGATGATCCAGGATTGATGCCAACGCCTCGAAAAGTTGTCAAAAGTAGTTCTGTCAAGGAATTAGAG
GTTGCTAAAGGAGCAGCAGCAGATTATTTAAG
AAGTTATCACCAAAGGAAGAATGTTAGTGAATTTGATTTCAGGGGTTTCCACAATTATGTTGCAGATG
ACAGTGACAGTGACGACGAGAGTTGTACAAGC
TCCGATCTTTTCGAGCTCGATCATCTTATTGGGATTGGAAGGTACAGAGAGGAGCTCCCAGTGTATGA
AACCACTAACTTCAAAACTAATCAGGCCATTG
CTAATGGCTTTTTCCCGTAA
>Ptr_Potri.008G098200
ATGTCCATCACCGGACTCTCATCAGACACCGGTAAACCTTACAAGAAGTCCTTGCATCGGAGAAATGA
TTCTGACGAGCTTGATGTATTTGAGGCAGCAA
GGTATTTCTCGGGATACAATGAAGCAGGTGCAGGTTATAATGGCGCAGTTTACACACAGAAAGTCATG
AGAGAAGATCATAAACATTCTTGGAGGGGAGG
AAGAGTGAGCCTAGATGTACCAATGAGGAATCCACTACCTCATCATCTCCATCAACATTCTCATACAG
TGGAGAAGCAAATACTGAAAGAGAAGAAATAC
AAACAACCAAGCTCTCCAGGTGGGAGACTAGCCAGCTTCTTGAATTCTCTCTTCAATCAAACAAGCTC
CAAAAAGAAGAAATCAAAGTCCACCACGCAAT
CAATGAAAGATGATGATGAGAGCCCCGGTGGAAGGAGGAAAAGGAGGAGCAGCATTAGTCATTTTCGA
AGCTCAGGCACTACTGATACAAAGTCTTTGTA
TTCTTCTTCAAGTTCTGGTTTTATGACACCCCCTCCTTATACACACACTCCTACAAAGGGCTACAAGG
AGCTCAGAAGCTGTTCAGATCACAGGCAAATA
GTTTCCCTGCCAAAGCAGAATGGGATTGTGAAGTCCATAGCCTTCCGAAATGAGATATTGGATGATAA
AAAGAACACAGATTTATCTTGGCTGGAAGAGA
AGTATAAATTCAATGATGGGTTCTCAGATCAGAAAGTACCCAGGAATCGTGGTAATCAACATTTAGAG
AAAGACAGGACTTGGGTGGACCAGTATCCATC
AGAGGAAAAGGAATGTAGGAAGTTCGATGAGGTGGATGATGGAACTGAGAGTGATTCAAGCTCTGATT
TGTTTGAATTGCAAAACTATGACTTGGCCGGT
ACCTACTCCAATGGTCTGCCTGTGTATGAAACAACACGTATGGATAGCATCAAAAGAGGAGCAGTACC
AATTTCCAATGGAACCCTATGA

>Nnu_XP_010260799.1
ATGTCTGTCACCGGGTTCTCGTACCCAGCAGAAAGGGTCTACAGGCGAGTTCACCGGAGAAATGATTC
CA
GTGAACTTGATATATTCGAAGCGAAGCAGTATTTCTCTGGCTGCAATGAGGTCCTCGGGCTCTATGGT
GC
AGCTCTTTCTCACAAGGCCATGAGAGAAGAAAGGCAGGAGTGGGGCATGCCCATGCGGCATACGCTTC
AG
TCTCAGTCTCGTCAGATCGAAAGCCAAATCATCAACGAAAATAAACAGAAGAAACCCAGGTCTCCATG
TG

GTAGACTTGTCAATTTTCTCAGCTATCTATTCCACCAAGCAGTTTCCAGGAAGAAGAAATCAAAGTCC
AG
TACACAGTCAGAGAAAGATGAAGAAGAGAGCTCAGGTGGAAGGAGAAAAAGGAGGAGCAGAAGCATGA
GT
AGTCATTTTCGAAGCACCAGCACCACCACCGATTCAAAGTCGATGCGGTCATCTTCAAGCTCATCTGG
TT
ATAGAACACCTCCTTATGCAAACAGTACTACTTCTACAAACATGTACAAGGATCTCATTAGTTACTCT
GA
TCACAAACAAGTGGTGCCTTTGCCGTCGGAAAACAATGAGAAGGTAAGCTCGATTAGCTTAAAACGAG
AG
GGTTTGAATAAGAAAAGAAGTATGGATTTGACTTGGTTGGATGAAAAACTACTCAAGTTCAACTGTGG
AC
TTGAAGAGAAAACCAACAACTTGAATCATGGGTTTTTAGAGAAAGATAGGACTTGGGTTGAGGATTAC
TC
AGCAGAGAACAAGAGCTTTACAAGGTTCGATGATGTAGATGATGGTGGAGAGAGTGATTCAAGCTCTG
AT
CTGTTTGAACTGCAGAATTTTGACATTGGTTTCTACTGA

>Nnu_XP_010261958.1
ATGGATAGTTGGGAGAAATCGCTAAGAGACGACAGATTTCTTCGTCAAGAGAGAAAGAACCCATCTTT
CT
CTTCCACCCTCCTCGACGCAATTTACCGTTCCATAGACGAAGGCGACGGAGAAGGAGAACAAGAACTT
GT
TCTCTATAGAGAAAGCATGGGGAGGAAACAGAGCTGTACCAGCAGCGGCCTCAAAGGAAGAAGTTTCT
TA
GAGATGGACAGTCTTCGGCGAGGGTGTATGATCGAGAAATGGATGGACAAAAAGGTAAGCGAGAAGGT
CG
TTTTCCGTCGTAAGTCGACGCCGGAGTTCGACCTCAAGTTACAGAACGATCGAAATTCCACACTCCTC
AA
TTCCACTTCATGCTCTTCAGATTCTAGCTCCGGAGGTGGATTTTCTTCTTCGGAAGCGGAGTCCGTTT
AC
GGAGTTAGATCGAGGTCGTCGGGTTTCACGGCGCAGAAGTCGAAACCGGTTCGAACTTGTATACCGGT
TC
GGTCGGAGAAACCTCTGCACTACTACGATCATGAACTAGAGATAAAGTTGCATGCTTTTGAAGAAACT
CA
TCGTCGTCATCATCTGGAGAAAGATTCAGTTCCACAGCAGAAGCCTTCGAAAAACGAAGGCGGTCGTT
TC
ATCAAGACTAAATCCAGAGCACTAAAGCTCTATGGCGATCTAAAGAAGGTGAAGCAACCGATCTCCCC
AG
GGGGACGACTTGCAATGTTTCTCAATTCTCTGTTTACTTCAGGTAATGCCAAGAAGGCAAAGATATCA
TC
TTCGGTTGGTGGTGGGGAGGATACGTGTTCTGAGAGGAACTCGAAATCGGCGCAGGCGTCGACATGTT
CG
TCGGCGTCGTCATTCTCGAGATCGTGTTTAAGCAAAAGCCCATCTTCCAGGGGAAAATTGAGTAATGG
CT
TGAAGAGGTCCGTCCGATTCTATCCAGTTAGCGTGATCGTGGACGAGGACTGCCGACCGTGTGGACAC
AA
ATGTCTATATGAAGATGATCCGGGCCTGATAGTGAGACCAATTGCAGAGCCCCGGTTGATGAATGAGG
AA
CTTAAATTCCACTTAATGGAGAAGAACAGGCGAGTGGAGGAAGCAGCACGAGATCTTCTGAAGAGCTA
TC
AGAAGAAGAGTGATTTCGAAATGAGAGACGCCAACGTCGACGTTCGCGACGATGACGACGAAGAGGAA
GA
CGACGACGATGATGCGGCGAGCTACTCAAGCTCCGACCTTTTTGAGCTCGAGAACCTAGCAGTAATTG
GT

ATCGACAGGTATCGTGAAGAGCTTCCGGTGTACGAAACAACCCATCTCGATACGAATCGCGCCATCGC
TA
GTGGCTTGATTCTGTAG

>Nnu_XP_010273945.1
ATGGATAGGTGGCATAAGTCGCTAAGAGAAGACAGATTTTGTCAAGAAAGGAAAATCCCATCTTTTTC
TT
CCACTCTCCTTGACGCGATTTACCGTTCCATAGACGAAGACGAAGGGGGAGAAGAAGAACTGGTTTTC
AA
TAGAGAAGCCATGAGGAAGAAACAGAGCATCGCAACAGACAGCAACGTTAGAGGAAGCAGTTTCTTAC
AA
GAAGATAGGGCGAGTCTTCGACGAGCCTGTATGATCGAGAAATGGATGGAGAAGAAGGTGACCGAGAA
GG
TCGTTTTCAGGCGGAAGTCTACCCCGGATTTGGACCCGAAGCTACCGAATGATCGAAAGTCTGTGCTC
CT
CAATTCGACTTCATGCTCTTCCGATTCTAGCTCCGCAGGTGGGTTTTCTTCCTCGGAAGCGGAGTCTG
TC
TACGGAGCAAGATCGAGGTCATCGGGTTTCACGGCGCAGAGGCCGAAACCGGTTCGAACTTGTATTCC
GG
CTTGGTCGGAGAAACCTCTGCAGTACTACGGTCATCAAGAGCAGAGAAAATTGCATGCTTTTGATGAA
GC
TAATCTTCGTCATCACCAAGAGAGAGATTCTCTTCCACAGCAAAAGCCTTCAAAGAACGAAGGTGGTC
GT
TTCATCAGGACCAAATCGAGAGCATTAAAGATCTACGGTGATCTAAAGAAGGTGAAGCAACCAATCTC
GC
CGGGGGGACGGCTTGCCGCGTTTCTCAATTCTCTGTTCACCGCAGGCAATGCGAAGAAGGCAAAAATC
TC
ATCTTCGGCTTGTGGTGGTGGTATGGAAGATACGTGTTCGGAGAGGATTTCGAAATCGGCACAGGCGT
CG
ACATGTTCGTCGGCGTCGTCATTCTCGAGATCGTGTTTAAGCAAAACCCCATCTTCGGGTGGGAAATT
GA
GCAATGGCATGAAGAGGTCGGTTCGATTCTACCCAGTTAGCGTGATCGTGGACGAGGACTGTCGGCCA
TG
TGGGCACAAATGTCTCTACGAAGACGATCCGGGCCTGATCGTGAGACAAATGGCGGAGCCCCGTTTGA
TG
AATGAGGATCTCAAATTCCACTTGATGGAGAAGAACAGGCGAGTCGAACAAGCAGCACGAGATATTCT
CA
GTAGCTACCAGCAGAAGAAGAGCGGTTTCGACATTAGAGACGCTGAAATCGATGTTTGTGACGAAGAA
GA
CGATGAGGGAGATGACGCGGCGAGCTATTCAAGTTCTGATCTTTTCGAGCTCGATAACTTAGCAGTAA
TT
GGTATAGATAGGTATAGAGAAGAGCTGCCAGTGTATGAAACAACCCATCTCGGTACCAATCGCGCCAT
CG
CTAGTGGCTTGATTCTGTAG

>Nnu_XP_010257962.1
ATGTCCATCACAGGATTCTCACACTCAGATAAGATTTACAGGTCAATTCACCGGAGAAATGATTCCGG
TG
AACTTGACGTGTTCGAAGCCGCACGGTACTTCTCCGGCTGCAACGAGGTCCCCGGGTTGATGAATGGT
GC
ATCTTTTTCTCAAAGGAACATGAGAGAAGAGAGGCAGATGTGGGCAGGCAGAAGGATGAGCTTAGATA
TG
CCGATGAGGAACCCACTTCCATCGGAGTCGCATCACAATATCGAAAGCCAAATCAAAGAGAAGAAGCA
CA
AGCAACCCAGGTCTCCAGGTGGTAGACTTGCTAGCTTTCTGAATTCTTTATTCCACCAAGCCGCTTCT
AA

GAAGAAGAAATCAAAGTCCAGCGCGGAGTCGATGAAAGATGAAGAGGAGAGCCCAGGTGGGAGGAGAA
AA
AGGAGGAGCAGCATTAGTCATTTTCGAAGCACCAGCACTACCACCGATTCAAAGTCGTTGCATTCATC
TT
CAAGCTCTGGTTTTAGAACACCTCCTCCTTGTGCAAACACTCCTACAAAGATGTTCAAGGATATCAAG
AG
CTATTCAGACCACAAGCAGGTGGCGCCTTTGTCGACCAAGTATACTTGTGGGCAGGTAATAAGGAGTG
TT
AATGGTTTGGGTTTACACCACGATGAGGCGTTGAACGAGAAGAGAAACATGGATTTGAGTTGGTTGGA
TG
AAAAACTGAAATTCAATGATGGGTTTCGGGAGAAAACTAAGAACGTAAATAGTGGGTTTTTGGAGAAA
GA
TAGAACTTGGGTGGGAGAGTATAACTCGTCTGAGAAGAAGAATTTTACAAGTTTTGGTGAAGTAGATG
AT
GGTGGGGAGAGTGATTCAAGCTCTGATCTGTTTGAGCTACAGAATTATGATTTGGGTTTCTACTCAAG
TG
GTCTACCTGTTTACGAGACCACTCAAATGGACGCCATTAAGAGAGGAGCACCAATTCTTAGCGGCAGT
TG
TGGCACCCGATGA

>Vvi_F6H3R8
ATGAACAAGTGGGAAAAAATCATTGAGAGAAGATAAATCCAGTCACGAGAGGAAGAATCCATCTTTCTC
TTCGAGTCTTCTGGATGAAATTTATCGTTCGATTACCGATGGTGACGAGGATCACAAAGAACTGCATT
TTTACAGAGAAACAATGGCGAAGAAGCATAGTTTGAGAGGCAGCAGAGGCGTTGGAGAGGAGGAAATG
GCGAGGGTTCGTGGAGCGGGCGTGACGGACAAATGGATGGAGAAGAAAGGTAGTGAGAAGGTTGTTTC
GCGACATCAACGGCGGTCGTTGCCGGAAATTGATAAAAAATGGCAGTACTATCAGGATCCTCTGTTCT
TCAGCTCGAGTTCTAGCTCTTCTGATTCCAGTTATGGAGGATTTTCGTCGTCGGAGACGGAATCGATG
AAGTCCTTCTTCGCTCCGCCACCAAGGCCTAGACCGATTCGAACCTCTCGTTTGGAGAGGTCGGAGCA
GGCTTTGTCCTATGGACAGAGCGAATTTCACATGTTTGATGACTATCGCCATCACTCTGCGATCACAA
GAGAACCGTCAAAGAGCGAAGAAGAGATTATCAAGTCGAAATCGAGAGCTTTGAAGATGTATGGCAAC
CTCAAGAAGGTGAAGCAGCCAATCTCACCGGGAGGCCGACTCGCGACGTTTCTTAATTCACTTTTCAC
TGCAGGAAACACTAAGAAGTCGACGAAGACCACATCATCAGCTGGAGGTTGCGAGGATTGGAGTTCCG
AGAGGAAGCTTAATTCGCAGCCATCAACTTGTTCTTCAGCCTCCTCATTTGCAAGATCATGTTTAAAC
AAGAATTCTCCTTCCGTAAAGGAGAAATCCCGCAATGACGCGAAGAGGACAGTCAGATTCTTCCCGGT
TAGCATTATTGTGGACGAAGACTGTCGGCCATGTGGCCGTAAATGCGTTTATGGAGAAGAAGGCTCAA
CTCAAATGTCGGCATCCAGAAAAATTACCAAAAGAAATGAAGAAGACCTCAAATTCCAGGACCAAACC
AGACGAGTCGAAGCAGCTGCCAGAGAATTACACAGAAACTTTCATCAGAAGAAAAACGATAACATTTT
CAATAAATTTCATGTCAATTATGACGAAGAAGACGAAGATGATGATGCTGCAAGTTGTTCAAGCTCTG
ATCTATTCGAGCTCGATCACCTCGCATTAATCGGTAATGACAGGTATCAAGAAGAGCTTCCAGTGTAT
GAAACCACTCATATAGATACTAATCGCGCCATAGCTAATGGCTTGCTTGTGTAG

>Mgu_A0A022S3L0
ATGGTGGATAGGAGAGAGAGTAATATCTCTCACACACAGTTTACACACAGTTCCTCACACTTCGGTGG
AT
ATTCATCGTCATCATCGAACAGCTGCCCATCCTTCTCCTCCGTGTTGCTCGACGAGATTTACCGGTCG
AT
CGATCGGCCGGAAGAAGAGATGCACACGATAACGAAGAAGAGTGACGGTCATGCTAACGTGCGCCCAG
CG
TACATTAAGAGTGATGAATTAGGAATTCCGGCGAATGATTATTACCAGCGTGCTTGCATGGTTGAGAA
AA
GGATGGAGGCGAATAAGACTCGAAAAATCCGGCACGGTTCGAGCTCCGCCAGCTCTTGGGATTCATTA
GG
CGGCAGCGGCGGCGGGGGCGGAGGCGGAGGTTTCTTCTCCTCCTCGGAAGCTGAATCGTTTCGGTCGC
AG
AAATTCGGCGGCGGTGGCGAGATCAATACGGATCCCGAAGTAATTATCAGTCCGCGTGATCGACGGGG
GT

TTGAGGATCGAATGGGGCAGAAATCGAAGACGAAACTGCGAGCGCTGAGAATATACGGCGATTTGAAG
AA
GATGAAGCAGCCGATTTCTCCCGGCCGGAAACTCGCCGGATTTCTGAACTCACTCTTCACCGCCGGAA
AC
CTAGGTCTGAAGAAGAAGCAGACCGGAACCCCAACCATCCCCGGCGGCGAAAATTCTCCAACGCTGAA
AT
CGACCAATTACCCATCCACGTCGTCGTCTGCTTCCTCATTCTCGAGGTCGTGCCTGAGCGGCAAAATG
CC
GCCGCCGCCGAGTGGGAAATCCTCCGGCGGCGGGAGATCAAGGAAGGTGAGCTTCTTTCCGATGAGCG
CG
ATCTCCGATGATGAGGACTTCCCCAACAACAACAACAACGTCAGTATTATGAAAACCGCCATTGATGA
AG
AGATCATGGCGCACATGATGAAGAAAAATCGACGAGTGGGAGAAGCTGCTAGAGATTTTCTCGTAAAT
AA
TTATCAAAAGAAAACCGAAACGGAGGAAGAAGAAGAAGAAGAAGATGCAGCAAGTTGTGGAAGCTCGG
AT
TTATTCGAGTTGGATATATTTGGAGCATTGGATAATGGAGAGATACACACAAGAGTTGCCGGTTTATG
A

>Lja_ Lj0g3v0201679.1
ATGGAAAAGTGGGAGAAAACCTCAAGAGATCACAGGAAACAACACCACAGAGAAAAAAGCCCTTCTTT
CTCTTCTACTCTTCTTGACGTTATCTACCGCT
CCATCGACGAAGGACAAACAGAAGAGAAAGAAGAATCACTCATATTCTACAGAGAAACCATGAGGAAG
CAGAAACAGAGCAACTGCTTCAGAGAAGAACA
GAGTGTTCAAATTGAAGCTGAAAACCCCAGCTTTCGCAGAACCAGGAAGGTAGAGAATTGGACTGAGA
TAAGGAATTCACTGACTGAGTTGGAACGAAGA
ACACGAAGCAATTCCAATACACTCTCCATGTATTCAAGCTCTTCCTCCTCAGAGTCAAGTTCTGCTGG
TGGATTCTCTTCATCAGAATCAGAGTCCTTCT
ATGGAGTGCAGAGGCCAAAGCCAATTCGGACCAGTGTTTCTGAAAAATTACCAAACCCTGATGATGGT
CACAGAAGCTTTTCCGGTCGGTCAAAACCGTC
CGCTCGCCCCAACACGGCTCAGGTCGGTCCAAACGGTCACACGGCCAGCCTGAATGACACTGGCCGAG
AGACTGTTTGGACCGGCAGCATCGGGGCGAGC
CGTCCGAAACAGCATGGTTTTGGGAAAACTAAGTCCAAAGCTTTGAGAATCTTGTACGGTGAATTGAA
GAAAGCAAAGCAACCCGTTTCACCAGGTGCTA
GGCTAGCTAGCTTTCTGAATTCTCTTTTCAATTCTGGTGGAGGAAATGCCAAAAAGGCAAAGGTTTCA
ACTACACCACCAACAACAACAACAAAGGTTTC
AAACTCAGCACAACCTTCTTCTACTTGTTCATCAGCTTCATCTTTTTCAAGGTCTTGTTTGAGCAAAA
CCCCATCTTCAAGATCAGGTGCAAAAAGATCT
GTGAGATTTTGCCCTGTGAGTGTGATAGTGGATGAAGATTGCAGACCATGTGGGCACAAGAATCTGCG
TGAAGCTGATTATGGAAAGAGTAACGATGAGG
AGCTAAGGTTGCATGTGATGAACGAGAGTCGAAGGGTGGAGGAGTTAGCAAGAGAATTGTTGAAGAAT
TATCAGAAAAAGAATGCAGGGGACTATGATCG
CATGCAGTGTGAAGATGAAGATGATGATGATGCAGAAAGTTGTTCAAGTTCTGATCTATTTGAATTGG
ATAATCTATCAGCAATTGGGATTGAGAGGTAT
AGGGAAGAATTACCTGTGTATGAAACTACCCATTTCAAAACCAATCGAGCTATTGCCAATGGCTTTGT
GA
>Lja_Lj2g3v1227800.1
ATGTCCATAACAGGGCTAATAGACCCAGAAATGAATCACAAGAAGTCCTTCCACCTCAGG
AATGATTCTGGTGAGCTTGATGTGTTTGAAGCTGCAAGATACTTCTCAGGATACAATGAA
GTTGTTGGCTACACTGGCTCTACTCTTGCCCAGAAGATCATGAGAGAAGAAAGGCATGGA
CACAGAGCCAGAATCAGCTTAGATATGCCAATGAGAAGCTTGCTTCCTCAGCAATTTCAT
GGCATGGAGAAGCAGATGAAGGAGAAGAAACACAAGCAGCCTAGCTCCCCAGGTGGTAGG
CTTGCAAGCTTCTTGAATTCTCTCTTCAACCAATCAGCATCTAAGAAGAAGAAATCAAAG
TCCAGCTCACAGTCCATGAAGGATGAAGATGAGAGCCCTGGTGGAAGGAGAAGGAGAAGG
AGCAGCATTAGCCATTTCAGAAGCTCNACACTCTCTTCTTTAAGCTCAGGATTCAGAACT
CCTCCTTATGTACTACAAACTCCAACAAAGAGCTGCAAGGAATTCAGAACCTCCTCAGAC

CACAAGCATACAGTTCCCTTGCCAACAAAGTTGAATGGACATGCAAAACAAAACACTTTG
CAAAATGAGTTGAAGAAAGACTCAACTTTTCTGGATGATAAGTATAAACATAGCAATAAT
GGGTTATCAGAGAAACAAAAGAACTGGGGGAATGAGTTGCTTGAGAAAGATAGGACATGG
CTGGAGAAATACTCATTAGAGGAGAAGGATATCAGAAAATTCAATGAGGTTGATGATGGT
GCAGAGAGTGATTCAAGCTCTGATCTGTTTGAATTGCAAAACTATGACTTGGGTTACTGC
TCAAGTGGTCTACCTGTCTATGAAACTACCAATATGGATAACATCAAGAGAGGAGCACCA
ATTTCCAGTGGTCTTCTGTGA
>Lja_Lj0g3v0252339.1
ATGGAGAAGATGATGCCTAGAGAAGAAGGGTTCCCACAAAGGAGAAGAACCCCATCCTTC
TCCTCCACACTCCTAGACGCCATTTGTCGCTCCATCGATGAATCAAAATCCAACGTGGAT
GAAGATCCACAACTGGGTCTCTTCAATGAAACTACCAACCCAAAACAGAGCATTTCAAAA
CAGAGCAATTCAACCCACTGTGCCAATATTGCAGAGAAAAGTGGCAACAAGGAGAGGATG
AATCTTCGCCGTGCCGTGATGATTGAAGATTGGATGGAGAAGCAGAGCTCATCACAAAGC
TCGCACTTCACAACTTCAAGCTCCTCTGAATCCACTTCAGGGGCAGGGTTTTCATCCTCA
GAAGCAGAGACAACCACCCACAAATCAAAACCAAGACACAAATCAGAGAAGAAACAACAA
CAGCAGCAACCGCAGAAGCCGAAGAGGGAGGGTGACGGTGGTGGAGGTGGTGGTTTTTCG
AGGACCAAGTTGAAGGCATTGAAAATCTATGGTGAGTTGAACCAGAAAGTGAAGCAACCA
ATTTCACCGGCGAGTCGCATAGCAAGTTTCCTCAGCTCAATTTTCAACGCCGGGAACATG
AAGAAAGCCAAAATGTGCTATGCTGGTTCCACACAAGATGTTGATTTCAACCACAAATCC
AAGTCCCCATGTTTCTCTTCCTCTGCATCTTCTTTTTCAAGAAGGTCTTGCATGAGCAAA
ACCCCAACTTCTTCATCTTCTTCCTCAGCAAAAGGGAAAAGATCAGTGAGATTCTACCCA
GTTAGTGTGATCCTTGGTGAGGATTCTCAAAACGATCCAAGTTTGTTGCCAGTTTCTAGT
GTTAGAAAGATGACAAGAGTGTCTTCAATTAAGGAGGTGAAGAAGAATTCTGCTAAGGTT
AAAGAAAATGTTCTTATGAAGGGTTATCAAAATTCTTGTAAGAATAATCAATTTGATTTT
AGAGGTTCCTATGATCATGGTGAGGAGAGTGATGATGATGATGATGATGATGCTCTAAGT
TGTTCAAGTTCTGATCTGTTTGAGCTAGATCACATAGTTGGAGCTGGAAGATACCAAGTG
GAGCTTCCTGTTTATGAAACTACAAATTTAGAAAGGAATAAGGCCATTGCTAATGGTCTT
TGTTTGTAG
>Lja_Lj1g3v1788080.1
ATGATAAGGAGAGAACGCAGATTCTTAAACGAGTCTGAAAACCCCTCATTCTCCTCCACC
CTGCTTGACCAGATTTACCGTTCCATTGATCAAGGAGATTCTGACATGAAGTTCTACACA
GGGAAAACAGGGGAAAAACAGAGCAGGGTGTGTGTAACAGAGGAGCATTTGTCGGCAAAA
TCGAGTGTTTCTCGTGATTGGAAAGCAAACAATGTAGTTGGAACTCAAGGGAGGCAGAGG
ATGATGCAGAAGGAAAGAAATTTCCATCTGGGTCGTCAAGTTCATGTTCATGACCAAGAT
GCTCTGTTTTTCAGCTCTACTTCGAGTTCTTCAGATTCAAGTTCGGGGTTGCTGTCGTCC
TCGTCCCCAGATATTGAAACCTTGTACGGAGTGAGATCAAGAGGTTCGTGTTTCGCTCCG
GCGAGGCCTAAGCCGGTTAGAACCACCGTGCCGGAGAAGAAGCACGTAGAGAGACAGAGA
CACATTGTGAGTGACGATGATGATGGTTTGATCAAGTCGAAATCGAGGGCGTTGAAGATT
TACAACAACCTGAAGAAGGTGAAGCAACCGATCTCACCAGGGGGGAAACTCACGAGTTTT
CTGAACTCTCTGTTTACTACAGGGAACAGCAACAACACGAAGAAAACCAAGCCCTGTTTT
GAAGATGCGAACGCTTTGAGAAAAACAGAGTCAGGACAAGCTTCTACTTGCTCCTCTGCT
TCCTCTTTTGCTCGCTCTTGTTTGAGTAAGAATTCACCTTCCACCAGAGAAAAAATTGCGG
AACAATGGGGTTAAGAGGACGGTGCGTTTCTACCCTGTGAGCGTGATTGTGGATGAAGAT
TCTCGACCCTGTGGGCACAAGTCCTTGTATGAAACAGAGGATGCGAGGCTAATGGCTATG
TCGGTGCCAACCGCGTGGAAAATTGGAACGAAGAAGAATGTTGAAGAGGTCAAGGACAAG
AAAAACAGGGCAATGGAAGAGGCTGCTAGAGAGATTTTGGAAGAGTACCATCATATCCAG
AGGAAGAGCGATTTGGTTTTGAGAAGAAACTTGGAGGATGAAATTGAAGATGATGATGAG
GATGATGCAGCGAGTTGTTCAAGTTCGGATCTGTTCGAGCTTGATCACCTGCATGAGGAG
CTGCCTGTGTATGAGACTACTCGTGTTGGTACTAATCGTGCCATTGCTAATCGTTTCATA
ATGTAA
>Lja_Lj5g3v0465910.1
ATGTACAAGTTGGAAAAGCCACGCAGAGATCACAACAAGTTGCAAAGCCCCTCTTTCTCT
TCCACCCTCCTGGACCAAATTTACCGTTCCATCGATGATGGCCAGAGTAAAAGCTCAGAA
TCAAATTTCTACAGAGAAACAACAACAACAACAACGAGTAAGAGACACTGGACAGAGAAA
AAAGACAGAACAGACAACAACAACAACAAGGTAAGAAGCAAAAACGTTGCTGTTGTGGAA
GAAACAAACAACAGAAAGTTCCATCATGATCACGACCCAGATGTTCTGTTCTTCAGCTCC

ACATCAATTTCTTCGGACACTAGCTCCGGAGGATTCTCCTCCTCTGACACTGACTCCACG
TGGCCGAGGCCAGTGAGGACAAGCGCGTCGTTCCGATGGGAGAACGAGAAACATGCGACG
CGTGTCCAGAGTTTTATGCATCTCACGGCCCAACACAAACACGGTGTTACCGGTGTTCGT
GATGAGGAAGCATTGATCAAGTCCAAATCGAGGGCGTTGAAGATTTACAACAACCTGAAG
AAAATAAAGCAACCTGTTTCACCGGGTGGAAAGCTCACGAGTTTCCTCAACTCTCTGTTC
GCAACAGGGGAAGCAAAGAAGAAGAAGAAGAAAAATGATGAGATTGTTGAGAGAAGAGTT
AACTCGGGACTAGGTTCTTCTTCTACTTGCTGCTCTGCTTCTTCGTTTTCAAGGTCTTGT
TTGAGCAAGGTTTCATCTTGTGATAGAGAAAATATGAGTAATGGGGTGAAGAGAACGGTG
CGTTTTTACCCGGAGACAGTGGAGGAGAAGAAAATAGTGGTGGAGAAAGCTGCTAGAGAG
TTCTTAAGAGAGTACCGTTACAACAACCAGAATAAGAAGACTGATTTGGTTTTGAAGGAT
TTGAGTTTAAGGAAAAATGCTAATTATAGTGAAGAACATGAAGAGGAGGATTATGATGAT
GCAGCAAGCTATGCAAGTTCAGATCTTTTTGAGCTTGATCATCTGGCTGTGTTGGGAAAT
GGTTTGTATGATGAGGAGCTTCCTGTTTTTGAAACTACACATTTTAGTTCAAATCCCACC
ATTGCTAATGGTATAAGAGTGTAG
>Ath_AT1G54200
ATGGATCCTTGGGATACTTATAACATACCACTAGACCACCAACACCGTCTCCGTAACCGCCGTGACCA
CCGTCATCCTTCCTTCTCCTCTACTCTCCTCG
ACCAAATTTACCGCTCCATCGACGATTCCTCTACTAACTCCTCATCCATGAGAAAAACCAAACATCAG
AACCGCGAGGACACGCGTGTTTCTGCTAACCG
CCGCGACGATTTCAACAGATCCAAGAATCTTAAAACCATAGAACCTGTTTTCTTCAAACATTCAAGCT
CAAGCTCCTCTGACTCAAGCGGATTCTCGTCT
TCCGAGTCTGATTATTTCTACAGACGATCTAAATCCTCGCCGGCGATTTCTCATCCTAAACCGATACG
AACCACCGTGGAACGATTCGAAAGATCTCCTC
AGAATCACCGACCTAACAGTAGTAATAAACAAGAACATGGAAGCTTTCTCAAAACGAAATCTAAAGCG
TTGAAGATTTATAGCGATTTGAAGAAAGTGAA
ACAACCGATTTCTCCCGGTGGTCGTCTCGCTACTTTCCTTAACTCCATATTCACCGGCGCCGGAAACA
CTAAAAAACTTAACAAAATCAACACAACCGTC
ACTTCCACCACCGCCGCCGCCGCCGCAGCGTCATCAACCACTACATGCTCCTCTGCTTCATCATTCTC
CAGATCTTGCTTGAGCAAAACGCCATCGTCTA
GCGAAAAATCGAAAAGATCCGTACGTTTTTGTCCCGTCAACGTCATCTTCGACGAAGACTCCTCCAAG
TACAACAACAAGAACAACAAAGTGTACGGAAA
CAACGAACGTGAATATGAATCCATTCGTCATACTCTAGAGAACCGAGTCATGGAAGAGAATCGTCGTG
TAATCGAAGCAGCTAAAGAACTTCTGAGAAGT
TACCAGAAGAAGAATAAAGAAGTCATTGAAGTCTCCGTCGAAGACGATGAAGAAGACGACGATGATGA
TGCGTTGAGTTGCACAAGCTCTGATCTGTTCG
AGTTGGATAATTTGTCGGCGATAGGAATCGATAGGTATAGAGAGGAGCTTCCGGTTTACGAAACAACT
CGCTTGAACACTAATCGTATCATTTCTAGATG
A

>Ath_AT3G13980
ATGGAGATCACTTGGGAGAAACCTAAATCTTCATCTCACCACCGTAACCCTTCTTTCTCCTCCACTCT
CCTCGACCAAATCTACCGCTCCATCGACGATT
CCTCCCCCCCTCCTCCCCTCGAATCCATCAAGAAGAAGAAGCATCACCACCAACAACGTAACGCTTCT
CTACACGAAGATCGTGAAATCTCTCCTATCTA
TCACCGCCGCTCAATCGCCGCTGATTTCGAGAGATCTAGGAGGAAGACAGACTTTCTCAGACATTCCA
ACTCTAGCTCTTCTGATTCAAGCGGATTCTCC
TCTTCCGAGTCAGATTCATTCCACGGACGCTCCAAATCTTCCGCTTCTCCTCCTTCTTCTTCTCGTCA
ACAACCGAAACCGATTCGAACATCATCCGTCG
ACCACTCCTCCGCCGTCCAGAAACCTAAAGAACTCGGTGGCTTCTTGAGAACGAAATCAAAAGCGTTA
AAGATCTACTCCGACTTGAAAAAAGTCAAACA
ACCTATCTCTCCGGGCGGCAGACTCGCTACTTTCCTTAACTCTCTTTTCACCAACGCCGCCACTAACC
CTAAGAAGCACAAGAAAACTACCACCGTCGCC
GTCGTGGAAGAACCACACTCCTCCTCCACATGCTCCTCCGCGTCGTCCTTCTCTAGGTCTTGCTTGAG
CAAAACTCCATCGTCTAGCGGCAAATCAAAAA
GATCCGTACGGTTCTGTCCAGTCAATGTGATCCTTGACGAAGATTCCTCCTTTACCATGCCTTACGCT
TATAACAACGAAAGACTCTACGATAACAACGA

AGCTAAACGCGTGGAAGAGCATCGCCGTGTCATCCAAGCAGCCAAAGATCTTCTTAGAACTTACCATA
ACAAGAACAAAGTTACAACTACGAACATCAAT
AACGTAGAAGAAGATGATGAAGACGATGCTGCGAGTTGTGCAAGCTCTGATCTATTCGAGTTGGAGAA
TCTGTCGGCTATTGGGATCGAGAGGTATAGAG
AAGAGCTTCCGGTTTACGAAACCACTCGTCTTGATAATATGAATCGAGTCATTGCTACTGGTTTAATT
GTATAA

>Cpa_evm.model.supercontig_1.160
ATGTCCATCACTGGACTCTCTGACTCCGATAGACTCTACAAGAAATCACACCACTGGAGGAATGACTC
CGGCGAGCTTGACGTTTTCGAGGCTGCAAGGT
ATTTCTCTGGGTATAATGAAGGTGGTGCTGGATATAACGCATGTGGATCCTACACGCAGAAGATCATG
AGAGAAGAGAGGCAGCCATGGAGACCAGGAGG
TGGAAGAATAAGCTTAGACGTGCCCATGAGATCAAATCCAGTCTCGCATCTTCACCAACAGTACTCTC
ATCATCATCTTCATCACCATTCATCATCTATG
GAGAAGCAGATTATGAAGGAGAAGAAGTACAAGCAACCGAGCTCTCCAGGTGGCAGACTGGCTAGTTT
CTTGAACTCTCTATTCAATCAAACAGGTTCCA
AGAAGAAGAAATCAAAGTCAACGACACAGTCAATGAAAGACGAAGAGGAAAGCCCAGGTGGAAGGAGG
AAAAGAAGGAGCAGCATTAGCCATTTTCGAAG
CTCCAGCACCACTGATACAAAGTCCATGTATTCTTCGTCTTCGGCGTCAGGGTTTAGAACACCTCCTG
CTTACGCACACACGCCGACAAAGAGCTATAAG
GATTTGAGAACCTTGTCGTCGGATCACAAGCAAATAATTTCCTTGCCAAAGAACGCAAAATCTACACA
AAATGATGAGGCCTCTGCAGATCATGACCAAA
TTAAAATCAACACAGATTTTTCTTGGTTGGATGAGAAATTAAAGTTAATTGACGCTTATACAGAGAAA
CAAAAGAATGTGGGTAAGAACCAAGATAGAAT
GTGGGAGGAGAAGGAGTTTAGAAAGTTGAACGAGATGATGGATGATGGAGGAGAAAGTGACTCGAGCT
CTGATCTGTTCGAGCTGCAGAACTACGACTTG
GGTATTTATTCAACTGGGTTGCCTGTTTACGAGACGACGCATATGGACACTGCCGTGAAAAGACCAAT
TTCCAATGCAGCCCTATGA

>Cpa_evm.TU.supercontig_126.40
ATGTACGGGTGGGAGAAATCACTCGGAGAAAATAGATACAGACAAGAGAGGAGGAAACCCTCTTTCTC
TTCGAGTCTTCTAGATGAAATCTATCGTTCAA
TCGATGATTGTGGAAGTGATTTACAGGACTTGAAGTTTTACCCACACACAATGCAAAAGAAACAGAGC
AAAGTTACGACCAAAAGCAGTAGAATTCCACG
AAAGCAAGAAGAGGTTTCTGGGAAACGTAGAGCTTGCTTGGTTGAGAAATGGGTGGAAGAGAAAGCGA
ATGAAAAAATCCATTGTTGCACGTCAGAGAGGC
CAACTGTTGAAAATTTTCGAGAGAGAAAATCATTACGATCACGATCACGATCACGATGCCTTGTTATT
TAGCTCCACTTCAATCTCATCAGATTCAAGCT
CTGGTGGGTTCTCATCCTCCGACACAGATTCCATCTACGGTACAAGATCTAGAGGTGGGACTAAGCCA
ATCCGAAACAGCGTCTTGCCTCGCTCACAGAA
AACAGAGTGTACTCTGTTTTATCAACGAAGAGAGTTTCAGTTGTTGGACGATCACGATCCAAAGCAAG
AAGATGGTTTCATCAAGTCAAAATCCAGGGCT
ATGAAGATTTACAGTAATCTCAAGAAGGTGAAGCAGCCGATTTCCCCCGGCGGCCGCCTCGCCAGCTT
CATTAATTCTCTTTTCAGTGCAGGAAGGTCCG
CAAAGAGCTCATCACCATCAACAATGATGAGCTGCGATGTAGATAGAACCATCCATAGCAGACAAAGC
AAATCAGGTCAAATCTCAACATGTTCCTCTGC
TTCTTCTTTTTCTCGGTCATGCCTGAGCAAAAACTCGCCTTCTACAAGGGAGAAGCTGCGTAATGGAG
TTAAAAGAAGCGTTCGGTTTTACCCTGTTAGC
ATCATTGTAGACGAGGATTGCAGACCCTGCGGCCATAAAAGTCTGTATAAAGAAGAAAACGATGCGGG
AGTAATGTCCTTATCAGTCCCAACTTCATGGA
AAATCGGCAAGTCGCCGTCGAGGAAGAAGGAGGAAGAGCTGAAGTTAATGGTGATGGAGAAGAGCAGG
AAAGTAGAGGAAATGGCGAGGGAGTTATTGAG
AGACTACCATCATCACCATCATTGTCATCATCACCATCGCCATCACCATCACCATCACCATCATCATT
CAAAGAAGAACAGTTTGACAATCTCCTCAAAC
GTTGGGGATAATTATGATGATGATGATGCAGCCAGTGATTCAAGCTCGGATTTGTTCGAGCTTGATCA
TCTTTCAGTAATTGGAAACAACAGGTATGATG

AAGAACTTCCTGTGTATGAAACTACCCACATGGATACTAATCGTGCCATTGCTAGTGGCTTGATCCTG
TAA

>Cpa_evm.TU.supercontig_25.102
ATGGCTGACACCAAGCATTTCATGAATTCCGTCTTGTTCAACTCAGCTTCCACTTCTTCTGATTCTTC
TACTGCTGGGTTTTCCTCCTCTGAGTCAGACT
CCTTTTATACTACCACTACTACAGCTAGGTCAACTTCATCGTCTCCAACTTACACCACCCCACGCAGA
CCTAACCCTATCAGAACAACGTCAGCCGCCGG
CTATGCTCTGCCCGAGAAGTCTCGTCATGTCATGATGGAAAATACCCATAAAAGTAAGTCCGATCATA
AGCGTGAAGATCATGGTGGTTTCGTCAGGACG
AAATCCAAGGCCTTAAAAATTTACAGCGATCTCAAGAAGGGTAAGCAGCCTATATCACCAGGCGGTCG
TCTTGCTACGTTTCTCAACTCTCTTTTCACAG
CCGGAAACGGCAAGAAGGTTTCCAAGATTTCATCTTCCTCGGCGACAAACGAAGAACAAACGCCGACT
TGCTCGTCTGCTTCCTCTTTTTCACGATCTTG
CTTGAGCAAGACCCCGTCTTCGCGTACAAAATCCAACAGTAACGGCACTAAAAGGTCTGTTAGGTTCT
GCCCTGTCAGTGTTATCGTCGACGAGGACTGC
AGGCCTTGTGGCCATAAATCTCTCTATAACCAAACCCAGTTCATGACAGAAAGTAACGAAAGAGTCGA
CCACGATCAGTGGCGAGTTATGGAGGAGAATC
GGCGTGTAGTTCAGGCTGCAAAGGATCTTCTGAAGAGTTACCGGCAGAACAAAGTGGAGTTCGACATG
AGAGATCATGTTATCAGCCATCAAAAGAAAGT
GAGATGTTATGATAATGTAGAGGAGGGTGAAGAAGAAGAAGATGATGATGATGCAGCAAGTTGTTCCA
GCTCCGATCTCTTTGAGTTAGATAATCTTTCG
GCTATTGGGATTGAGAGGTACAGTGAAGAGTTACCGGTATATGAAACAACTAATCTGGGTACTAATCG
AGCTATTGCTAATGGGCTGATCCTGTAA

>Cpa_evm.TU.supercontig_28.87
ATGCATAGAAGAAGAACCCCTTCTTTCTCTTCTTCTCTTCTTGATTCTATTTATCGTTCAATCGATCA
CTCCAACACTTCCCAATCACACTTTGGATACT
ACAGCCCAACTAAATCATCCGCCATGTTTAACAACCACCAAGATCCTGACTATGATTCTCGCCTCCAC
CAGGATCTTCGTCGAGCTATTATGATCGAGCA
ATGGGTCGAGAAGCACAACGACTATGGCGTCTCTGCTCACCTCAACTGTTCTTCTAGCTCCTCCGATT
CCAGCTCCGGAGGAATATTTTCGTCTTCCGAG
GCAGAGTCTTGTCATAAAACCAAGTCAAGATCAAGATGTTCCAGACAGGGAAGATCAGATAAGTGCAT
GCAGCGGATTACTGATAACCAGAAGCAGCAGC
AAAAGCAGCCAATGCGTGAATCTGGAAATGGTGGTTTTACCAAGACGAAGCTCAAAGCCCTGAAAATT
TACGGAGAGTTAAAGAAAGTGAAGCAACCAAT
TTCACCTGGTGGCCGCATAACCAGTTTCCTCAACTCAATTTTCAATTCTAACGCCAAGAAAGTCAAGC
TCTGTTCTGTTGGTGCCATGGAGGATGTGGGT
TTTGAGCGCAAATCAAAATCTACTTGCTCCTCGGCTACTTCGTTTTCTAGGTCTTGTTTAAGCAAATC
ATCTTCTTCGTCAACGAGAGGTAAGCACGGTA
AGAGTAGCAATGGCGAGACGAGGAGGTCTGTTAGATTTCATCCTGTTAGCGTCATTGTGGATGAAGAT
TGTAGACCCTGTGGCCATAAATCCATCTATGA
AAACGATCCAAGCTTAATGCCAACTCCAACGGTTCACAAGACATTGAAAGAGGAGCTCAAGGCTTATG
CCATGGAAAAAGAAGCAGGAGTTGACAGAGTA
GCTCGGGATTATCTGAGGAAGTATCAGAAAAAGCAAGAATTTTCTTTGAGGAGTCAAGTTGAACATCA
CGAGAATGAAGAAGATGAAGATGGAGGTGAGG
ATGCATTAAGTTATTCAAGCTCAGATCTATTTGAACTTGATCACCTTATTGGGATTGGAAGGTACAGA
GAGGAATTGCCAGTTTATGAAACTACCAATCT
AAAAACTAACCAAGCCATTGCTAATGGTTTGATTTTGTAG

>Tca_Thecc1EG011482t1
ATGTCGGTTACAGGAGTTTCAGACCCTGATAGAATGTTAAAGAAGTCATTCCATCGCAGGAATGACTC
TGGTGAGCTTGATGTGTTTGAGGCTGCACGGT
ATTTCTCAGGGTACAATGAAGCAGCCAGCTATAACTGTGCAACATTCACTCAGAAGATCATGCGGGAA
GAGAGGCAGCCCTGGAGAGGAGGCAGGATCAG

CTTGGACGTACCGATGAGAAACCCTTTTCCTCAACAAACTCATGCTGTGGAAAAGCAAATTAAGGAGA
AGAAATACAAGCAACCGAGCTCCCCAGGTGGT
AGACTTGCTAGCTTCTTGAACTCTCTTTTCAATCAAACAGGTTCCAAAAAGAAGAAATCAAAATCTAC
CACGCAGTCCATGAAAGATGAAGAAGAAAGCC
CTGGTGGGAGACGGAAAAGGAGGAGCAGCATTAGCCATTTCCGTAGCTCTAGTACCGTTGATACAAAG
TCCTTTTATTCTTCTTCAAGTTCTGGTTTCAG
GACCCCACCTCCTTACGCACACACGCCCACGAAAAGCTACAAGGATTTTAGAAGCTATTCAGATCACA
AGCAGGTGGTAACTCTGTCAAAGTACAGTATT
GGACAAACAAAATCTACAGCTTTAGCAAACGAAGTCTTGGAAGACAAAAGGAATACAACAGACTATTC
TTGGTTGGATGAGAAATTCAAGTTCAATGATG
GCTACTCAGAAAAACACAAGAATAATCTCGGTACCCGTCATCAAGAGAAGGATAGGAATTGGGCTGAT
CGATATCCATCAGAGGAGAAAGAATTCAAGAA
GTTCAATGAGGTTGACGACGGTGCAGATAGTGATTCAAGCTCTGATCTGTTTGAGTTGCAAAACTATG
ACTTAGGCATCTACTCAAGTGGCTTGCCTGTG
TATGAAACTACACATATGGACAGCATCAAGAGGGGAGCACCAATTTCCAATGGTGCACTATGA

>Tca_Thecc1EG029108
ATGGATATGTGGGTTAAACTGCCAAGAGAAGATCGGTGTCGAAACAGGAGAGAAAACCCATCTTTCTC
TTCTACTCTTCTTGATGCTATCTACCGTTCTA
TTGATGAATCCAACGGTAGTAAAGGAGAGGAAGAACTGATTTTCTACAGAGAAACTACCATGAGGAAG
AAGCACAGCAATAATTGTTCATTGAAAGAAGA
AGAAATGACGAGCCTTCAACGTGCTTGCATGATCGAGAAGTGGATGGAAAAGAAAGCCAGCTGTGATA
ACAAGGTTGCCATTAGACGAAAATCCATGGCG
GATTCTGAAAGAAACTCTCGAAATGACTTTGATCCCGTGTTGTTGAACTCGAGCTCCAGCTCCTCTGA
TTCCAGCTGCGGTGGTGGGTTTTCATCCTCGG
AATCAGATTCCTTTTATAATGCAAAATCAAGGTCATCATCTTCATCGTCAAGTCATTACACTACTCAT
AGGCCTAAGCCTATTCGGACGAGCGTCTCAGC
TCGGCCCGAGAGATACGAAAGGCCTCAACATGAAGTTGAGAACAGTTTCCATGCTGCTGCACAAAAGC
CAAAGCATGAAGGAGGTTTTGTGAGAACAAAA
TCCAAGGCCTTGAAGATTTACAGTGACCTCAAGAAGGTTAAGCAGCCGATATCTCCAGGTGGTCGGCT
TGCAAGTTTCCTTAACTCGCTTTTCACTGCAG
GGAATGCCAAGAAGGCAAAGATTTCATCGTCAGGGTACGAGGAAAGGAAGTTAAAGTCCGAGCAAACA
TCATCAACATGTTCTTCAGCTTCCTCGTTTTC
AAGGTCCTGTTTGAGCAAGACTCCATCTTCAAGAGGGAAGTTAAGCAGTAATGGGACAAAAAGGTCGG
TCAGGTTTTGTCCTGTTAGCGTTATTCTTGAT
GAGGATTCCAGGCCATGTGGGCATAAAAGCATTCACTATGAAAATGATCAAACGTCAATGATTAGAAA
ACCAAGCAACAAAGAGCTTGAGTTCCGCAACT
TGGAAGAAAACCGTCGAGTCGTGGAAGCTGCAAAGGATCTTTTAAAGAGTTACCAGAAAAAGAAGGAA
GAGTATGACATGAGAGATGTCCGCAATGGCAA
TGGAGATTCTAGTGAAGACGACGACGAAGAAGACGCAGCAAGTTATGCAAGTTCCGACCTCTTTGAAT
TGGATAATCTTTCAGCTATTGGTATTGAAAGG
TACCGAGAAGAATTGCCTGTGTACGAAACAACCCATCTCGATACTAATCGGGCCATTGCTAATGGTTT
GATTGTGTAA

>Tca_Thecc1EG037363
ATGTACAGATTGGAGAAAACACTGAGAGAAGAAAGATACAGGCACGAGAGAGATAACCCATCTTTCTC
TTCTACCCTACTTGACAAAATCTACCGGTCCA
TCGACGACGGTGAAGCGAATCATGAAGACCTGAAATTCTACAGGGAAACAATGCAAAAGAAACAAAGC
AAAGGGAACATGAAAAGCAGCAGAAGTAGAGG
AGGAGGAGGAGGAGAAGAGATGTCGAGTTTCCAACGAGCTTGTCTGATCGAGAAATGGATGGAAAAGA
AGGTTAGTGAAAAGGCAAATGCAGAAAGGAAA
CAGGTTTTCTCTGAGTTTGAAAGAAAATCACATCATGAACATGATCATGATCATGATGTTCTCTTCTT
TAGCTCCACTTCAAGCTCATCTGATTCCAGTT
CTGGTGGTTTCTCATCATCTGATACTGAATCTATGTATGGTACAAGGACGATAGCTTCATGTTTTGTG
CCACCAAGGCCTAAACCTGTTAGGACCAGCGC

GTCGGCTAGGTCGGACAAACCGCTGAAAGCAGAGAAAACAGGAAGATCAGAGAGGGCTCTGTTTTATG
AACAGAGGGAGTTGCATATGTTTGATGATTAT
CATTATAACTCTGCCTCTGACCACACACCAAAGCTTGATGAAAGTCTTTTCAAGTCAAAATCAAGAGC
CATGAAAATTTATGGCAATTTAAAGAAGGTGA
AACAACCCATTTCACCTGGTGGCCGTCTCGCGAGTTTCATCAATTCTCTGTTCACAACAGGTAATACA
AAGAAAACCAAGAGTTCATCTTCAATTGTAAG
CTGTGATGATGAAAGAAAATTGAAGTCAGGGCAGGTCTCAACGTGTTCTTCAGCTTCCTCATTTTCAA
GGTCATGTCTAAGCAAAAACTCGCCTTCTACG
CGAGAAAGGTTGCGTAATGGAGTTAAGAGAACAGTTAGATTCTGCCCGGTGAGTGTAATTGTTGATGA
AGACTGTCGACCATGTGGGCAAAAATGCTTAT
ATGAAGAAGAAGACTCGAGTTTACTGTCGGTTTCAGTTCCAACTGCATGGAAAATCGGGAAAACGTCA
TCAAGAAAGTGCGAAGAAGAGCTAAAGTTGCA
AATAATGGAGAAGACAAGGCGAGTGGAAGAAATGGCTAGAGAGTTTTTGAAGGAATATCATCTAAATC
AGAAGAAGAATGATTATATTCCAAGGGACAGT
CGCAGTAATTATGTGGACGAGATGGATGAAGACGAAGACGATGCAGCTAGCTATTCAAGTTCGGATTT
GTTTGAGCTGGATCACCTTGTTCTCATTGGCA
ATGATAGGTACCGAGAAGAGCTTCCAGTGTATGAAACTACTCATGTTGAAACAAATCGAGCCATTGCT
AATGGCTTGATAGTGTAG

>Tca_Thecc1EG037572
ATGTATAGAAGAGAGGGTTCCGTCAGAGAGACTGCGGTTCCTCAAAGAAGAAAAACCCCTTCTTTCTC
TTCTAGTCTTCTTGATGCTATTTATCGTTCCA
TTGATGAATCAGCTAATGGAGATGAAGCAACATTGTGTCATTACAGGGAAACAAAAACGACCCTTGTT
AAGAAGCAGAACAATGCACCTTCGGAAGAAGA
GAGAAGGGTTTCCAGTCTTCGACGTGCTATTATGATTGAGGATTGGGTTGAGAAGCAAAGTGGTTATG
GCTCAGCTGTGCATTTCAACTCAACTTCAAGT
TCCTCGGATTCTAGCAGTGGAGGAATCTTCTCGTCATCCGAAGCTGAGTCAAGTTACAAAGAGAAATC
AAGAAGATCAACACCAGCTAAACCCGAAAAGT
CTAAGCAGTTTGAACAAAGAAACTTTGACAACAACAACAACAATCAACAAAGGGCCAAGCGTGAAGGT
GGTGGCTTTTCTAAGACCAAACTGAAAGCCTT
GAAAATATACGGCGAATTGAAGAAAGTGAAGCAGCCGATTTCTCCTGGTGGTCGTATCACTAACTTCT
TAAACTCCATTTTCAATGCCAATGCGAAGAAA
GTGAAGATGTGCTCCGTTGGGGTTTCAGATGATGTAAGTTTTGATCGCAAATCAAAAACTACATGTTC
ATCAGCTTCTTCCTTTTCCAGGTCTTGTTTGA
GCAAAACACCTTCTTCAAGAGGCAATAAGTATAGCAATGGCAAGAAAAGGTCAGTTAGATTTTGTCCA
GTTAGTGTCATTGTTGATGAAGATTGTAGACC
CTGTGGCCATAAATGTATTTATGAAGATGATCCAAGTTTAATGCCAACTTCAACTGTTCAAAAGAATG
TTAAAAGTTCTTCAAGAAAGGAGGAGCTGAAG
AATTTTGTTAAGGAGAAAGAATCAGGAGTTAGCAATAAGGCAAGAGATTATCTAAGGAGTTATCAACG
GAGGGGTACTGGTAAATTGGATTTGAGAGGCT
TTGTTGACGACTATGAGGATGATGATGAAGAAGAAGAAGACGACGCTTTGAGTTATTCAAGCTCTGAT
CTTTTTGAGTTGGATCATCTCATTGGAATTGG
AAGGTATAGAGAGGAGCTGCCAGTGTATGAAACTACTAGTTTGAAAACTAAGCAAGCCATTGCCAATG
GCTTCATATTGTAG

>Ath_AT1G13670
ATGTCGGAGATGGCCGGAGATTCTTCAACATCAGAAGATAAAATCCATAAACCAAACCGCGAGAGGAA
TGTCTCCGGCGAACTTGACGTCTTCGAAGCCA
CTCGATATTTTTCCGACTTCAACGAACCAACGACGATCGAGTACTCAAGAATCCAAATACAGAAACAA
AGCATTGTTACAGAAAATCGACAGAAGAGAGT
ACACCCTGAGACAGAAGAACACCTCCCTAAACCTAGAGTCGTCGTCATAAAGCCGCAGAAGAAAGAGA
TGACACGTGGCGGCGGTAAGAAGCTAACCAGT
TTCTTGAACTCTCTTCTTCGCTCAGCTGGTCTAAAGAAGAAGTCAAAGTCAGCGCCGGAGGTGGAGAC
ACCACGAGTAGAAAGGATGAGGAGGAAGAGCT
GCGTAGTGACTACACACGCCGCAGATGCTTCGCCTCTCACCAATGCCGGCGCGTGGAGTTTAAACGCG
CGTAGAAGAAGCTTTGATGAGAAAGATTTGAA

GAAGAGTGATCAGAAGTTGAACATAAGATTTTGTGAGAGTCTGTATTCGGACCAAAGGGTTGAGACAA
AAGACCCAAATGCAGGAGACAAGAACGATGTA
ATTGGTGGATGCGAGAGTGATTCAAGTTCTGAATCTGATCTTTTTGAGTTGGATTTGTTTGCTAAATC
AAACCCCTAA

>Ath_AT1G69160
ATGTCCATGAAAGGGATCTCATCAGCAGAGTCAGATAAGCTCTCTAGGAGAATATCTTTGACCCACAA
GCGCAATTCCGAAGAGCTTGACGTGTTTGAAG
CTGCCGTGTACTTCGGCTACAATGAAGCCTCCTCCGGTGACCATGGACATACACAAAAGTATGGCTAT
AACGCAGCGAGAGAGGAGAATCCGAGGAGATG
GGGAATATTAGGAGGTGGAAGAAGAATCAGCTTGGATTTGCCTATTAGATGCTCAGAACAAGTGTACC
ATCTTCAACAAGATCATCATGAGAAGCACGAA
GTTACAACAATCAAGGAGAGACTTGGTAACGTGAGACACAAGCAACCGAGTTCACCAGGTGGGAAAAT
CGCCAGCTTCTTGAACTCCTTGTTCCATCAAG
CAGGTTCGAAGAAAAACAAATCAAAGTCAAAGTCAAAGACAAAGCCAACAGATCCAGAAGTGGAAGAG
GAGATCCCCGGAGGAGGATGGATGAGGAGGAG
GAGGAGAAGCAGTATCAGCCATTTCTTTAGCTCAAGCAGATCTACTTCAACAACCACCACTACCACGG
CGTCATCATCTTCAAAATCTCTGATTTCCTCA
TCAAGCTCGGGTTTCAGAACTCCTCCTCCTTATTTGAACACTCCCACCAAGAACTACAAGCAGTTCTT
GAACTACACTTCTGCCACAAAACAAGTGGGAG
AAGAGGAAACTAAGACCAACAAAGAGTACTCTTGGCTGGACGAGAAACTCAAGGTGATGGAGAGCCTC
TCGGAGAATCAGAGGATTTGGTCGGATGATGA
AGATATCGATGATGATAGGAGAATAAAGAGAGAAGGAGAGGATGATGGAATGGAGAGTGATTCAAGTT
CTGATCTCTTCGAGTTGCAAAATTACGAATTA
TCTCGTGGAGGCTTGCCAGTCTACGAGACTACCAATGTAGCTAACATCAACAAGACTCATATATAA

>Ath_AT3G42800
ATGGCTTTTCCTCAGAGAAAACGAACACCTTCTTTCTCTTCTTCTGTTCTTGACTCTGTTTACCGTTC
TATCGACGAATCTGACGGTCTCCAAAGTGACC
TAAAAGGATCCATCAACGAAAATGTATCTTCATCATCATCCTCACCATCACCAAACAAGAAAGACGAC
AAGCTTACAACTCTAAGGCGAGCAATCATGGA
CGAAGAACACTGGCTTTACGCCCGAAGCAGCACCACTACCACTAACTCCTCTGATTCCTCTTCTTTTT
CCTCTTCCGAGGCAGAGTCATATCGAACAAAG
AGAAGGTTGAGAAAGCTTGCGGAACAAGGTAAAAGATCAGGTGATGAGAGACAGAGAACAAAGAGAAC
GGTAATGGACAACGATAGTAGATTGTTTTCCA
AGTCCGATGATGATAAGAAGCCTAAAGCCGTGAAGATAATTGAGGAGCTAAAGAGATCGAAGCAGCCA
GTGTCTCCTGGAGCGCGTCTCACTAGCTTCCT
CAACTCGATCTTCCAATCTAACGCCAAGAAAGTGAAGCTTTGTTCTGTCGGTAAGACAACAGACGTTA
AGTCCTCATCCTCAAAGTCTTGCTTCAGTCGA
ACAAGAAATAAAACCGACAACAACAACAACAACTGCAAGAAGTTGGAAAGGTCTATAAGATTCTATCC
GGTTAGAGTAACCATTGATGGAGACTGCAGAG
ACTATGCTCAAAAACACATTACTCGCGTGAGGAAACCAATTCCAGAATTTACCGCTAAGAAGAGTGTC
AAAGAGGAGATTAAGACCAACGATCATCACAC
TGAGTTTACTTGCATCACAAGAAATATAGGTTTGAAGGATTTTGTGAGAAGTAACAAATATGAAGGTA
AAGAAGAGGAGGAGGATGCTTGGAGTCACTCA
AGTTCGGATCTGTTTGAGCTTGATAGTTACCGGATCGGTATGGGGCGGTACTTAAAAGAACTTCCAGT
CTATGAAACTACTGATTTCAAGACTAATCAAG
CCATTGCTAGAAGCTTGCTTTTGTAG

>Ath_AT5G12050
ATGCATAAATCCAAAAGAAACCCGTCATTCTCCTCCACTCTCCTCGATGAAATCTACAATTCCATCGA
TCCTAAAACCCAAAAAACACAACCTTATGTCG
GCTCTGTTAACACTACTACCAAAAAACAGAGCATTGTGACAAGATCGGTTCCTGACCGGAAAATCCAC
CGTGATCGTTTCTTTGGCTCTGTATCTTCTTC
CTCTGATTCCAACTCTAGTATTTTCTCATCTTCAGACACCGAACTAACTCACGGTAAGAAGACAACTT
CTTCTAGGCCGTTATGTTTCGGTCCTTCTAAG

ACAAAACCAAGAAAAACAGAGGATAAAACTCTGTTCCACCAAAACAGAGCTACCCGAGTGTACGACGA
CTACGATTACGCCTCCGACGTCCCAAAATTCA
ATAGACACGACGAGAATTGGGAGAATACGAGAAACAGAAGATCGGTGAAGAGCTCCGGAAACCAAAAG
AAACCAAAAACTCCAGCTTCACCTGGAGGAAG
AATCGTTAACTTCTTAAATTCGTTGTTCAGCAACAATTCAAAACAATCCAATGCGGTTAAGAGTTATC
CAAGGAAGACTAGTTACGATGATTCTGCTTAC
GTTAGGAAAACAAGTAATGATTATCATTCATCTACTACTACGTGTTCTTCAGCTTCTTCTTTCTCAAG
GTCGTGTATGAACAAAGGCTACGAGAAATCAT
CTGGGCGTATCAAAAGAAGCGTTAGATTTTCCCCGGTCAATGTTATCGTCCCCGAGAGCTTCACCAGC
AAAGAAGAAGATTATTTCAGTAACGGTAACGC
TAGAAAGTCGGTGAAGAAGAATGTGGAAGATGGAGGGAGGAGATCAGTGGAGGAGATAGCGAGAGAGT
TCTTAAGAGATTATCACAAGAATCATGAAAAC
AGTTTGGTTAAGACTAATGGTTTTGAGGATTATGAAGATGATGATGAAGACGATGATGATGATGATGT
TGCAAGTGATTCGAGTTCAGATCTGTTTGAGC
TTGATTTAGTTGGAAATCATCATCATCATAATGTGTACGGAGATGAACTTCCTGTGTATGAAACCACT
TTTGCTGGTTTGATCTTGTGA

>Brara.A03221
ATGGAGACTTGGGAGAAGCCATCTTCTAGAGGTCACCACCGTAACCCTTCCTTCTCCTCCACTCTCCT
CGACCATATCTACCGCTCCATCGACGACAACT
CCCCTCCACTAGAACCCACAAGGAAGAAGAAACTCCTCCACGAGGATCTCGACACTTCTCCAGACAAA
CTAGTCTTTCACCGCCGCTCAATAGCAGCTGA
CTTCGAAAGATCCAGGCGAACCACCGCCACCACCACCACGGCCGCAGCCGACTCTGTTTTCCTCAGAT
ATTCCAACTCCACCTCCTCTGATTCAAGTGGA
CTCTCCTCCTCCGAGTCAGACTCCTTCTACGGACGTTCCAAATCCTCTGCATCTCCTCCGCAACCTAA
ACCAATCCGTACCTCCGCCGTGAGCTCCGGCG
AGAGACCCACCACCAAACAAGAACTCGGTGGCTTCTTAAGGACCAAGTCAAAAGCGTTGAAGATCTAC
ACCGACTTGAAAAAAGCGAAACAACCAATCTC
TCCAGGCGGACGGCTCGCTACGTTCCTCAACTCCCTCTTCACCAACGCATCCACTAACCCTAAGAAGC
CCAAGAAAAACACCACTTCCATCTCTGTCTTG
TCGGAGACACAGTCATCCTCCACCACGTGCTCCTCAGCGTCGTCTTTCTCTAGGTCTTGCCTAAGCAA
AACTCCCTCGTCTAGTGGAAAATCTAAAAGGT
CCGTACGGTTCTGTCCGGTGAATGTGATCCTCGACGAAGATTCCTCCGTTTACATCCCTTGCGGTAAC
GACACCAAACGTTATCGCCAAGTTATGGAGGA
GGAGAATCGTCGCGTTATCGAAGCGGCCAAGGATCTTATCAGAACGTACCAAAAGAACAAGGATCTTT
TGGCCGTGACAACGTGCCATGACGTTGAAGAT
GATGATGATGCGGCGAGTTGTGCTAGCTCGGATCTGTTCGAGTTGGAGAATCTTTCGGCGATTGGGAT
CGAGCGGTATCAAGAAGAGTTACCAGTGTATG
AGACCACTCGTTTAGATAATACTAATCGAGCCGTTGCTACAAGTTTAACTGTATAA

>Brara.B00441
ATGCATAGATCCAAAACCCAATCTAGAAACCCGTCCTTCTCCTCCACTCTCCTCGACGAAATCTACCA
CTCCATCGACCCCAAAACCCAAAAAACCCAAC
CTTTTCTAAACTCTCTCAACACCGCCAAGAAACAGAGCATCAGCGTAAACAGAGGGAGAGATCGGCTA
TTCGGCTCTATGTCGTCTTCCTCCGACTCCAA
CTCCAGCATCTTCTCGTCTTCCGACACCGAACTAACTCACCCTAAGAAGACAACGTCTTCGAGACCGT
TATGTTTCGGTCCTTCCAAGACAACAAAACCG
AGAAAAACAGAGGATAAAGCTCTGTTTCACCAAAACAGAGCAAAAACAGAGGATAAAGCTCTGTTTCG
CCAAAACAGAGCAACACGTGTGTCCGACGACT
ACGATTACGCCTCCGACGTTCCCAAGATCACGAGATACGACGAGGATTGGGAGAATAATCGAACCAGA
AGGTACACGAGTAAGATCAAAACTCCAGCTTC
GCCTGGTGTACGGATCGTTAACTTCATCAACTCTCTGTTCAGCAACAACGGTTCTTCTAAACAACCCT
CCTCTGCGGTTAAGAGTTATCCGAGGAAGACG
AGCTATGACGTTACTAAACCGACGTGTTCTTCAGCTTCTTCCTTCTCAAGGTCGTGTCTGAACAAAAC
CTCAGAGAAACCATCTGACCGTATCAAACGGA

GCGTCAGGTTTTCTCCGGTGAATGTGATCGTCACCGCCGCGGAAGAAGAAGAAGATTATCTGAGAAAG
TCGGTGAAGAAGAATGTGGAAGATAGAGGGAG
GAGATCAGTGGAGGAGATTGCTAGAGAGTTTCTGAGAGATTATCACAAGAATCATGAGAACGGTTTGG
TCAAGAAGAATGATTTTGAGGATTATGAAGAT
GATGATGATGATGTTGCGAGTGATTCGAGTTCGGATCTGTTTGAGCTGGATTTAGCTGGGAGTCATCA
TCATCATAATCTGTATGGAGACGAGCTTCCTG
TGTATGAAACCACTTTTGCTGGTTTGATCTTGTGA

>Brara.B01838
ATGTCCATGAAAGGGGTCTCATCAGCAGAACCAGATAAAATCTCTAAGAAAATATCTTTCCACAAGCG
CAACGATGCTCACTCCGGCGAGCTTGACGTGT
TCGAGGCCGCCATGTACTTCTCCGGCTACAACGAGTTCTCCTCCTGCGACCATAGACACTCACATAAG
CATGGCTATAACGCCGCAAGAGAGGAGAATCG
AAGGAGATGGGGAATATTAGGAGGAGGCAGAAGAATAAGCTTGGATTTACCGATTAGATGTTCAGATC
AGCAGGTGCATCATCTTCACCAAGACCATCTT
GAGAAGCCAGAAGTCAAAGCAATCAAAGAGAGGACTAGTAACGTGAGATACAAGCAACCAAGCTCACC
GGGTGGGAAAATCGTCAGCTTCTTGAACTCTT
TGTTTCATCAAGTGGGTTCGAAGAAGAACAAGTCGAAGTCAAAGCCAACGGAACGAGAAGCCGAAGAG
GAGATCCATGGAGGAGGATGGATGAGGAGGAG
GAGGAGAAGTAGTATCAGCCATTTCTTGAGTTCAAGCAGATCTAATTTAACCACCACAACGACATCAT
CGTCATCAAAGTCTCTGTTGTCATCTTCAAGC
TCCGGTTTCAGAACTCCTCCTCCATATTCAAACACTCCCACCAAGAACTACAAGCAGTTCTTGAATTT
CACTTCCGCCACAAACAAAGTGGAAGTCCTAG
AGGAAAAGAAAATAAATAAAGAGTTACCTTGGCTTGATGAGAAGCTTAACGTGATGGAGAGCCTCTCA
GAGAAAAAGAAGGTTTGGGGTGACCATGATGA
TGATGCTGATGGTGATCGGAGAATAAAGAGAGAGGGAGATGATGACGATGGAATGGAGAGTGATTCAA
GCTCTGATCTCTTTGAGTTGCAGAACTTCGAG
TTGTCTCGTGGAGGCTTGCCAGTTTACGAGACTACCAATGTAGCTAACATCAACAACACTCCTCTATA
A

>Brara.C00499
ATGCATAGATCCAAACCTCAATCCAGAAACCCGTCATTCTCCTCCACTCTCCTCGATGAAATCTACAA
TTCCATCGATCCCAAAACCAAAAAGACTCAAC
CTTTTGTCGGCTCTGTTAAGAAACAGAGCATCAGCGTAACCAGATCAGTTCCTGACCGGAAACTCCAC
CAAGATCGGTTCTTTGGCTCTGTCTCCTCTTC
CTCTGATTCCAACTCCAGTATCTTCTCATCTTCCGACACCGAACTAAGTCACGGTACTAAGAAGATAA
CTTCTTCAAGGCCGTTATGTTTCGGCCCATCT
AAGACAAAACAGAGTAAAACAGAGGATAAAGCTCTGTTTCACCAAAACAGAGCAACCCGAGTGTTTCA
CGACTACGATTACGCCTCAAGTAATCAAAAGA
AGCTCAAAACTCCATCTTCACCTGGAGTAAGAATCGTTAACTTCATCAATGCCTTGTTCAGCAAGCAA
CCAACGGCGGTTAAGAGTTATCCGAGGAACAC
GAGCTACGACGACTCTGCATTCTCTAGAAAACGAACTGATTATTACTATCCATCTACGACGTGTTCTT
CAGCTTCTTCCTTCTCCAGGTCTTGTCTGAAC
AAACGCTCCGAGAAATCATCTGGCCGTACCAAGCCAAGGGTTAGATTCTCTCCGGTCAATGTGATCGT
CCCTGAGATTGAAGAAGAAGACTATCTCAGCA
GCGGCTATGTTAGAAAGTCGGTGAAGAAGAATGTGGAAGATGGAGGGAGGAGATCAGTGGAGGAGATT
GCGAGAGAGTTCTTGAGAGATTACCACAAGAA
ACATGAAAACAGTTTGGTCAAGAATAATAATGATCTCGAGGATTATGAAGATGAGGATGATGATGTTG
CAAGTGATTCGAGTTCGGATTTGTTTGAGCTG
GACTTAGTTGGAACTCATCATCATAATCTGTATGAAGACGAACTTCCTGTGTATGAAACCACTTTTGC
TGGTTTGATATTGTGA

>Brara.E02722
ATGGAGACTTGGGAGAAGCCAAGAGTTTCATCTTCTAGAGATCACTACCGTCAACCTTCCTTCTCCTC
CACTCTTCTCGACCAAATCTACCGCTCCATCG

ACGACTCCCCTCCTCTAGAATCCACTAGAAAGAAGAAGCACCCGCACCGTAATATTACATCTCTCCAC
GAGGAACATGCTTCAGACAAACTCGTCTTTCA
CCGCCGCTCCATCGCCGCTGACTTCGAGAGATCCCGGAGAACCACCGCCACCTCCATCTCCTCCGTTT
TCCTCACATATTCCAACTCCAGCTCCTCTGAC
TCAAGCGGGCTCTCCTCCTCCGAGCTAGACTCCTTCTACCTACGCTCCAAGTCCTCTCCTCCGCACAC
TCGTCAACCGAAGCCTATTCGAACCTCCGTGG
TAGAGAAACCTAGCAGCAAACAAGAGCACGGTGGTTTCTTGAGAACCAAGTCAAAGGCGTTAAAGATC
TACACCGACTTGAAAAAAGTGAAACAACCTCT
CTCTCCCGGAGGACGCCTCGCTACTTTCCTCAACTCTCTCTTCACAAACGCATCAAGCAACCATAAGA
AGCTTAAAAAAATCAACACCACCGTCTCTTCC
TCAGCGGAGCAGCCACCACAGTCCTCCTCCAACACCACATGCTCCTCCACGTCCTCTTTCTCCAGATC
TTGCTTAAGCAAAACTCCATCGTCTAGCGGGA
AATCCAAAAGATCCGTAAGGTTCTGTCCAGTGAACGTTATCCTCGACGAAGACTCCTCCATCCATATC
CCTTACGGTTATAGTAACAAGCTGCATGGAAA
CAACGTCGACCGCCACGTCATGGAGGAGGAGAACCGACGCGTTATCGAAGCGGCTAAGGATCTTATTA
GGACGTACCAAAAGAACAAGGATCATTTGGCC
GTGACAACGTGCGACAATGTTCAAGAAGATGACGAGGATGATATTGATGATGATGCGGCGAGTTACGC
GAGCTCTGACCTGTTTGAGTTGGAGAATCTTT
CGGCTATTGGGATCGAGAGGTATCGAGAAGAGTTACCAGTCTACGAGACCACTCGTTTGGATAATACG
AATCGAGCTATTGCTACAAGTTTAATTGTATA
A

>Brara.F00058
ATGGATCCTTGGGATAACAACACTACAGACCACCAGTACCGCCGCCGCGACCACCGCCATCCTTCCTT
CTCCTCCTCTCTCCTCGACCAAATCTACCGCT
CCACCGACGACTCCTCCGACGTCTCCATGAGGAAGAAACAGAACCGCGCCGCCTCTCTTGATGAGAAT
CGCGTCTGCCTCGAGAAAATCCTCCTTAACCG
CCGCAAAACCGCCGACGATTTCGCCGTTAACCGCCGCAAAACCACCGAAATAAACACCGTCGAGCCAG
TTTTCTTCAAGCACTCAAGCTCCAGCTCCTCC
GACTCGAGCGGATTCTCCTCCTCCGAGTCAGACTCCTTCTACAAACGAACTCGCTCGTCTCGCTCTCC
GCCGGTGATTCATCACCATCCTAAGCCGATTC
GAACCGCCGTCGAGAGACTCGAACGGCCTAACAATAAAGTGAAATCGAAGGCGTTGAAGATGTACAGC
GATTTGAAGAAAGTGAAGCAGCCGATCTCTCC
CGGCGGACGCCTCGCCACGTTCCTTAACTCTCTCTTCACCGGAAACACGAAGAAACCGAATAAAACCG
TTTCCACCGCCACGTCATCACACACCACTTGC
TCGTCAGCGTCTTCCTTCTCCAGATCTTGTTTGAGCAAAACGACGTCGTCCAGCGAAAAAACGAAACG
GTCTGTTCGTTTCTGCCCCGTCAACGTCATCT
TCGACGAAGACTCCAAGCAACGAGAGTCCATTCGCCACCATCAAAGCCGAGTTATGGAGGAGAATCGT
CGCGTGATTGAGGCCGCCAAGGAGCTTATCAG
AACGTACCGGGAGAATAAAGACGTCGAAGAAGAAGACGACGACGATGACGATGATGCTGCGAGTTGCG
CCAGCTCTGACCTGTTTGAATTGGATAATCTA
TCGTCGATAGGGATCGAAAGGTATCGCGAAGAGCTTCCCGTCTACGAAACAACTCGTTTGAACACGAA
TCGTATTATCTCCAGATGA

>Brara.G02851
ATGTCCATGAAAGGGATCTCATCAGCAGAACCAGATAAGGTCTCCAAGAGAATGTCTTCTCACAAGCG
CACTGATGCTTATTCTGGCGAGCTCGACGTGT
TCGAGGCCGCCGTATACTTCTCCGGCTACAACGACTTCTCCTCAGGTGACCATAGACACACACAAAAG
TATAGCTATAACGCTGCGAGAGAGGAGAATCG
AAGGAGATGGGGAATATTAGGAGGAGGGAGAAGAAGAAGCCTTGATTTACCGATGAGATATTCAGAGC
AGGTGCATCATCTTCACCAAGATCATCCTGAG
AAACAAGATGTTACAATAATCAAAGAGAGGTTTGGTAACGTGAGACACAAGCAACCAAGCTCGCCTGG
TGGGAGAATCGCCAGCTTCTTGAACTCTTTAT
TTCACCAAGCGGGCTCGAAGAAGAACAAGTCAAAGGCAAAGCCAACGGACCGAGAAGTCGAAGATGAG
ATCCCTGGAGGTGGATGGATGAGGAGGAGGAG

AAGAAGCAGTATTAGCCATCTCTTAGGCTCAAGTAAATCTAATTCAACAGCCACAACGACATCATCAT
CATCAAAATCTCTGTTCTCGTCGTCAAGCTCG
GGTTTCAGAACTCCTCCTCCTTATTTAAACACTCCCACCAAGAACTACAAGCAGTTCTTGAATTTCAC
TTCTACCACAAACCAAGTGGAAGTACTGGAAG
AAAAGAAAATAAACAAAGAGTTGTCTTGGCTTGATGAGAAACTTAGAGTGATGGAAAACCTCTCAGAG
AAAGATAAGTTTTGGGCTGACCATGATGGTGA
TATTGATGATGATGATGATGATGAGGCCCGGAGAATAAAGAGCAAGGGAAAGGATGATGATGGAATGG
AGAGTGATTCAAGTTCTGATCTCTTTGAGTTG
CAGAACTACGAGTTGTCTCGTGGAGGCTTGCCAGTTTACGAGACTACCAATGTAGCTAACATCAACAA
CACTCATCTATAA

>Brara.H00090
ATGGATCCTTGGGATAAAACTAACTCATTAGACCACCACCACCACCGTCGCCAAGATCATCGCCATCC
TTCCTTCTCCTCCACTCTCCTCGACCAAATCT
ACAACTCCATCGACTCCTCCTCCGCCGTTAACCGCCGCGCAGTCGCCGGAGATTCCGTTAGATCAAGG
AATCTCAAGACCGCTGAGCCAGTTTTCTTCAA
GCATTGGAGCTCCTCCAGCTCCTCAGACTCAAGCGGATTCTCCTCTTCCGAGTCCGACTCCTTCTACA
GGCGATCTCGTTCTTCGCGTTCTCCGCCGGAG
ATTTGTCATCCCAAGCCGATTCGAACCACCGTCGAGAGACTCGAACGACCTAATAATAATAATAATAA
TAATAATAATAATAAAGTGAAATCGAAGGCGT
TGAAGATGTATAGCGATTTGAAGAGAGTGAAACAGCCGATTTCTCCAGGCGGACGTCTCGCCACTTTC
ATAAACTCTATCTTCACCGGAAACACTAAGAA
ACCGAATAAAACCGCCACGTCATCATCCACCACTTGCTCATCCGCGTCTTCCTTCTCCAAATCTTGCT
TGAGTAAAACGCCGTCGTCTAGTGAAAAATCG
AAACGGTCTGTTCGCTTCTGTGAGTCCACGCGCCAACGTCAAAACTTTGATACTCTTGAGAGCCGAGT
GATGGAGGAGAATCGTCGCGTGATTGAAGCAG
CCAAGGAACTTATCAGAACTTACCAGAAGAATAAAGACGTCGTTAACATCATCGGCAAAGAAGAAGAA
GATGATGAAGAGGACGATGACGATGGTGCGAG
CTGCGCGAGCTCCGATCTGTTCGAATTGGATCATCTATCGGTGATTGGAATCGATAGTTATCGAGAAG
AGCTTCCCGTCTACGAGACGACTCGTTTTCAC
ACGAACCGTATAATCTCCAGATGA

>Brara.J02180
ATGCATAGATCCAGAAACCCGTCATTCTCCTCCACCCTCCTCGACGAAATCTACAATTCCATCGATCA
TAAAACCCACAAAACCCAATCTTTTACCGGTT
TTGTCAACACTACCAATAATAAACAGAGCATCAGCGTAACCAGACCGGTTCCTGACCGGAAAATACAC
AAAGATCGGTTCTTTAGCTCCGTGTCGTCTTC
CTCTGATTCCAACTCCAGTTTCTTCTCATCTTCCGACACAGAACCATCTCACCCTAAGAAGAAATCTT
CTTCAAGACCGTTATGTTTCGGCCCATCTAAA
ACAACAAAGCCAAGAAAAACAGAGGATAAAACTCTGTTTCACCAAAACAGAGCGTTTCGAGTTTCGGA
AGACTACGATTACGAGAAGAATGAACACACTA
GAACCAGAAGATCTAGTAGTAATCAGAAGAAGCCCAAAACTCCAGCTTCACCTGGTTTACGAATCGTT
AACTTCATCAACTCTTTATTCAGCAGCAACAA
CAGTTCCAAAGGTCATCCGAAGAAGATGAGCTGCGACGACTCTGCGTTCGTTAGGAAACCAAATGATT
ACTACTATCCATCTACGACGTGTTCTTCCGCT
TCTTCCTTCTCCAGGTCGTGTCTGAACAAAAGCTCCGAGAAATCATCTGACCGTGTCAAAAGAAGCGT
CAGGTTTTCTCCGGTTAACGTGATCGTCCCCG
AGAGCAGATCCTACGTCGAAGAAGAAGATTACCTCAGCAACAACGGTTATGTCAGAAAGTCAGTGAAG
AAGAACGTGGAAGATAGAGGGAGGAGGTCAGT
GGAGGAGATTGCGAGAGAGTTTCTGAGAGATTATCACAAGAAGCATGATAACGGTTTGGTCAAGATTA
ATGCTTTCGAGGATTACGAAGATGATGATGAT
GATGTAGCAAGTGATTCGAGTTCGGATTTGTTTGAGCTGGACTTAGTTGGAACTCATCATCACAATCT
GTACGGAGATGAACTTCCCGTGTATGAAACCA
CTTTTGCTGGTTTGATCTTGTGA

>Cru_Carubv10001037m.g

ATGAGGAAAAGGACATTGGAGAAAACACTATTAGTCCTCGTCTCTTCTAACTTCATTCGTCGTCGTCT
TCTTCTTCTTCTTTCTGCTAAAAAACACACAA
AACAAAACAGACAATTACAATCTCTCTTTTTGTCTCTTCTTCTTTCTTCTTCTTTAATCCTCTTTCTC
GATCAAATCATCATGCATAAATCCAAAAGAAA
CCCGTCATTCTCCTCCACTCTCCTCGATGAAATCTACAATTCCATCGATCCTAAAACCCAAAAAACTC
AATCTTTTGTCGGCTCTGTTAACACTACTACC
AAAAAACAGAGCATTGTGTCAAGATCGGTTCCTGACCGTAAGATCCACCGTGACCGGTTCTTTGGCTC
TGTGTCGTCTTCCTCTGATTCCAACTCCAGCA
TCTTCTCTTCTTCCGACACCGAACTTACTCACGGTAAGAAGGTAACTTCTTCCAGACCGTTGTGTTTC
GGCCCTTCCAAGTCAAAACCAAGGAAAACAGA
GGACAAAACTCTGTACCACCAAAACAGAGCATCACGAGTGTACGACGACTACGATTACTCCTCCGACG
TCCCCAAATTCACGAGACACGACGAGAACTGG
GAAAATAACAGATTCAGAAGATCTGCAAAGAGCTCCGGAAACCCCAAGAAACCCAAAACTCCAGCTTC
ACCAGCCGGAAGAATCGTCAACTTCATAAATT
CGCTGTTCAGCAACAACAGTTCAAAACAGTCCAATGCTGTTAAAAGCTATCCGAGGAAGACGAGCTAC
GACGACTCTGCTTTCGTTAGGAAAACATCGAA
TGATTATCACTCATCATCTACAACGTGTTCTTCAGCTTCGTCCTTCTCGAGGTCTTGTATGAACAAAA
GCTACGAGAAGTCATCTGAGCGTATCAAACGA
AGCGTTAAGTTTTCTCCGGTCAATGTGATCGTCCCCGAGATCTTTACAAACAAAGAAGATGATTATTT
CAGTAACATGAGAAAGTCTGTGAAGAAGAATG
TGGAAGATGGAGGGAGGAGATCAGTGGAAGAGATTGCGAGAGAGTTTTTGAGAGATTATCACAAGAAT
CATGAAAGCAGTTTGGTCAAGAACAGTGGATT
CGAGGATTACGAAGATGATGAAGATGACGACGACGATGATGTTGCAAGTGACTCAAGTTCAGATTTGT
TTGAGCTGGATTTAGTTGGGAATAATCATCAT
CATCATAATAATGTGTACGGAGATGAACTTCCTGTTTATGAAACCACTTTTGCTGGTTTGATCTCGTG
A

>Cru_Carubv10009449m.g
ATGGATCCTTGGAACTTTAACGAACCTAATTTAGACCTCCACCACCACTACCGTCTCCGCAACCATCG
TCATCCTTCTTTCTCCTCTTCTCTCCTCGACC
AAATCTACCGCTCCATCGACGACACTTCCTCTTCCACTGACCTCTCCTCCATGAGAAAGACCAAGCAC
CATCAGAGCCGCGATGACACGTCTCGCGTTCC
TTCTTCTTCCAACCGCCGCACTACCGCCGACGATTTCATCAGATCCAAGAATCTTAAAAAACCTGTTT
TCTTCAAGCACTCCTCTACTACTACGTCAAGC
TCCTCCGACTCTAGCGGCGGCTTCTCCTCTTCCGAGTCTGAGTCTTTCTACAGACGATCTAAGTCCTC
TCCCGCTCTTTCTCATCCCAAACCCATTCGAA
CCACCGTCGAACGATCTCCTCAGGCTCACCGACCTAACAGCACTAATAAACAAGAACATGGGAGCTTC
CTCAAAACGAAATCAAAAGCTTTGAAGATCTA
CACCGATTTGAAGAAAGTCAAACAACCCATTTCCCCCGGCGGTCGTCTCGCCACTTTTCTCAACTCCA
TTTTCACCGGCGCCGGAAACACCAAGAAGCTT
AATAAAATCAACACTTCCACCGTCAACAACTCCACAGCCGCGGCGCCATCTACAACCACCACGTGCTC
CTCCGCGTCCTCATTCTCCAGATCTTGCTTGA
GTAAAACTCCGTCTTCCAGCGAGAAATCTAAACGGTCCGTACGGTTTTGTCCCGTCAACGTCATCTTC
GACGAAGACTCCTCCAACGCCAAGTACAAGAA
CAACAACAACAGCAAATTGTACGGAAACAACGAACGTGAATATGAATCCAGCACTCGCTCCCATCATC
ATCATCATCAAAACTTTGATACTCTAGAGATC
CGAGTCATGGAAGAGAACCGTCGTATAATAGAAGCAGCTAAAGAACTCATGAGAACGTACCAGAAGAA
ACATGATCAAGAAGATGAAGAGGAGGAGGAGG
ATGATGCGTTGAGTTGCACGAGCTCTGATCTGTTCGAATTGGATAACTTATCGGCTATAGGAATCGAT
AGTAGGTATCGAGAAGAGCTTCCAGTTTACGA
AACCACTCGGTTAAACACGACTCGTATTGTTTCACGATGA

>Cru_Carubv10010185m.g
ATGTCAAATCTTGAAACTCAAGAAACAACAATGTCCGAGATCGCCGGAGATTCTCCACCATCAGAAGA
GAAAATCAATAAACCAAACCGCGAGAGGAATG

TCTCCGGCGAACTTGACGTCTTTGAAGCCACTCGATATTTCTCCGACTTCAACGAACCAACTACGATC
GAGTCCCCAAGAATCCAAATCCAGAAACAAAG
CATTGTTCTTGAAAACAGACAGAAGAGAGTACATCCTGAGACAGAGGAGGAGCTCTCGAAACCTAGAG
CTGTCGTCACAAAGCCGCAGAAGAAGGAGATG
ACAAGTGTCGGCGCTAGGAAGCTAACAAGTTTCTTGAACTCTCTTCTTCGCTCAGCGGGTCTAAAGAA
GTCAAAGTCGAAGTCAACGACGGAGGTAGAGA
CTCCACGAGGAGAAAGGATGAGGAGGAAGAGCTGCGTAGTGACGGCCACACACGTCGTAGCTTCTTCT
CCTATTCCAGGTAACGGCGTGTGGCGTTTAAA
CGCGCGTAGGAGAAGCTTTGATGAGAAACACGTGAAAGCTTTGAAGAAGAGTGATGAGAAGTTGAACG
TAAGGCTTTGTGAGAGTAAAGATCGAAATGTA
GGAGATCATGAGACTGATGTAAACGGTGATTGCGACAGTGATTCAAGTTCTGAATCTGATCTTTTTGA
GTTAGATTTGTTTGCTAAATCAAACCCTTAA

>Cru_Carubv10016082m.g
ATGGAGACTTGGGAGAAGCCTATCCCTTCATCTCACCACCGTCGTCACCCTTCCTTCTCCTCCTCTCT
CCTCGACCAAATCTACCGCTCCATCGACGATT
CCTCCCCTGCCTCCCTCGACTCCACTAGAAACAAGAAACATCATCATCATCACCATCACCGTCATGAG
GTCTCTCCAGCTGATCACCGCCGCTCTGTCGC
CGCTGATTTCGAGAGATCCATGAGGAGGAGTACTACCGCCACCGACTTTCTCATACATTCTAACTCCA
GCTCCTCTGATTCTAGCGGCGGTGCATTCTCA
TCTTCCGAGTCTGATTCTTTCTACGGTCGCTCCAAATCTTCTGCCTCCCCTCCTCCTCCGTCCAGTAC
TCGTCAACAACCCAAGCCTATCCGAACATCGA
CCGTCGACCACTCCTCCCCCCTCCAGAAACCTAAAGAACTCGGTGGCTTCTTGAGAACCAAGTCAAAA
GCGTTAAAGATCTACTCCGATTTGAAAAAAGT
GAAACAACCCATCTCCCCCGGCGGCAGACTCGCTACTTTCCTTAACTCTCTTTTCACCAACGCCGCCG
CAGCTAACCCTAAGAAGCAGCGCAAGAAAACC
ACCTCCCCCGTGCGCGTGGGAGAAACAGTACACTCCTCCTCCACCACATGCTCCTCCGCCTCGTCCTT
CTCCAGGTCTTGCTTGAGTAAAACCCCGTCGT
CTAGCGGCAAATCAAAAAGATCCGTACGGTTCTGTCCAGTCAATGTGATCCTCGACGAAGATTCCTCT
ACCATTCCCATGCCATACACTTACAACAACAG
GCTCTACGGTAGTAACGAAGATAAACGCGACGTTATGGAAGAGCATCGCCGTGTCATCCAAGCTGCCA
AGGATCTTCTCAGAACTTACCATAACAAAAAC
AAGGTTACTACAACGTACGACGAGGACATCAAGTACATCGCCAATGTAGAAGAAGACGAGGACGATGA
TGCCGCGAGTTGCGCGAGCTCTGATCTATTCG
AATTGGAGAATCTCTCGGCGATTGGGATCGAGAGGTATCGAGAAGAGCTCCCAGTTTACGAAACCACT
CGTCTTGATAATATGAATCGAGCCATTGCTAC
TGGCTTAACTGTATAA

>Cru_Carubv10017637m.g
ATGGCTTTTCCTCAGAAAAAACGAACACCTTCTTTCTCTTCCTCTATTCTTGATTCTATTTATCGTTC
TATCGATGAATCAGACGGTCTTCAAAGTGACC
TAAGAGGAGGAACCATCAATAGCAACAACGACGAAAATGTATCTTCATCATCATCAACATCATCACCA
TCACCAACCAAAAAAGATGATAAGCTCACAAC
TCTTCGAAGAGCAATCATGGATGAAGAACACTGGCTTTACCCCAGAAGTAGCACCACAACCACCAACA
CCTCTGATTCCTCTGCCTTTTCATCTTCTGAG
GCAGAATCATATCGAACAAAACGAAGATCGAGTAAGTTTGTGGAGCAAGTAAATAAATCAGGTGATGA
TAAAAAGGTGAAAGCCGTGAAGATGCATGAGG
AGCTAAAGAGATCAAAGCAGCCTTTGTCTCCTGGAGCTCGTCTTACTAGTTTCCTCAACTCGATCTTC
CAATCAAACGCCAAGAAAGTGAAGCTTTGTTC
TGTCGGTAAGACCACCGACGTGAAGTCTTCTTCTCCAAGATCTTGCTTCAGTCGAACAAAACATAAAA
CCAACAGCAACAACAACTGCTGCAAGAAGTTG
GAAAGGTCCATCAGATTCTATCCCGTTAGAGTAACCATTGATGGAGACTGCAGAGACTATACTCAAAA
ACCGATCTCAGAACGTACTGCTAAGAAGAGTA
TCGAAAGAGTAGTGACATCGCTGAAAGAAGATATTAAGACCAAAGTTCATCAAACTGAGTTGACTTGT
GTCACAAGAAAGATAGGTTTGAAGGATTATGT

GACAAGTAACAGAGACGAAGGTAACGAAGATGAGGATGATGTTTGGAGCTACTCAAGTTCGGATCTGT
TCGAGCTCGATAGTTACAGGATCGGTATGGGA
CGGTACTTAAAAGAGCTTCCGGTTTATGAAACTACTGATTTCAAGACTAATCAAGCTATTGCTAGAGG
CTTGCTTTTGTAA

>Cru_Carubv10020681m.g
ATGTCCATGAAAGGGATCTCTTCAGCAGAGCCAGATAAGCTCTCCAGGAGAATATCTTTGACCCACAA
ACGCAATTCTGGTGAGCTTGACGTGTTCGAGG
CCGCTGTGTACTTCTCCGGCTACAACGAAGCCTCCTCCGGCGACCATAGAAACACACAAAAGTATGGC
TATAACGCGGCCAGAGAGGAGAATCGTAGGAA
ATGGGGAATATTGGGAGGAGGAAGAAGAATCAGCTTGGATTTGCCAATTAGATGTTCAGAACAGGTGC
ACCATCTTCACCAAGATCATCATGAAGTTACA
ACAATCAAGGACAGGCTCAGTAACGTGAGACACAAGCAACCTAGCTCACCAGGTGGGAAAATCGCCAG
CTTCTTGAACTCTCTATTCCATCAAGCAGGTT
CAAAGAAGAACAAGTCCAAATCCAAGTCGAAGCCAACGGAATCCGAAGTGGAAGAGGAGGTCCCCGGA
GGAGTATCATGGATGAAGAGGAGGAGAAGAAG
CAGTATAAGCCATTTCTTGAGCTCAAGCAGATCTAATTCAACCACCACCACGACATCATCATCATCAA
AATCTCTAATCTCGTCATCGAGCTCCGGTTTC
AGAACTCCTCCTCCTTATCTCAACACTCCCACCAAGAACTACAAGCAGTTCTTGAATTACACTTCTGC
CACAAAACAAGTGGTTGAAGAAGACAAAAAGA
CCAACAGAGAGTTCTCTTGGCTTGACGAGAAGCTCAAGGTGATGGAGAGCCTCTCGGAGAATCAGAGG
AATTGGGCTGATGATGAAGATGATGATCGGAG
AATAAAGAGAGAAGGGTATGATGATGGAATGGAGAGTGATTCAAGTTCTGATCTCTTCGAGTTGCAAA
ACTACGAGTTATCTCGTGGAGGCTTGCCAGTC
TACGAGACTACCAATGTAGCTAACATCAACAACACTCATATATAA

>Mes_Manes.01G025900
ATGTCCATTACAGGACTCTCAGAAACATCTAAACTTTACAAGAAATCGTTTCATCGCAGAAACGATTC
TGATGAGCTTGATGTGTTCGAAGCATCAAGGT
ATTTCTCAGGATACAATGAAGCTGTGGGTTATAGTGGTGCAACTTATACACAAAGAGTCATGAGAGAA
GATCATAGATATCCTTGGAGAGGAGGAAGAAT
GAGCTTAGACGTACCGATGAGAAATCCACTACCTCAGCAGTCTCATACAGTGGAAAAACAAATATTGA
AAGAGAAGAAATACAAGCAACCAAGATCTCCA
GGTGGTAGATTAGCTAGCTTCTTGAATTCTCTCTTCAATCAAACAAGTTCTAAAAAGAAGAAATCAAA
GTCTGCCACACAATCAATGAAAGATGAGGATG
AAAGCCCCGGTGGAAGAAGGAAAAGGAGGAGCAGCATTAGTCATTTTCGAACCTCAAGCACTGCTGAC
ACAAAATCTTTGTATTCTTCTTCGAGTTCTGG
TTTTAGAACACCCCCTCCTTATGCACACACTCCAATAAAGAGCTACAAGGACTTTAGAAGCTATTCAG
ATCACAAACAAGTACTTTCCTTGTCAAAGCAC
AATGGAAATGTGAAATCCACAGTCCTCCAGAATGAGGTATTGGATGATAAAAGGAACACAGACTTATC
GTGGTTGGATGAGAAATTTAAATGCAGTGACA
CTTTTGCTGAGAAACCCAAGAGCCTTGGTCATCGATATTTAGAGAAAGATAGGATATGGGTTGATCAG
TATCAACCAGAGGATAAGATATTTAGAAAGTT
CGATGAGGTAGATGATGGAGCTGAGAGCGATTCAAGTTCTGATTTGTTTGAATTGCAAAACTATGACT
TGGGTATCTATTCAAGTGGTCTGCCTGTGTAT
GAGACAACAAACATGGATAGCATCAAAAGGGGAGCACCAATTTCCAATGGCACCCTCTAG

>Mes_Manes.03G038000
ATGTCTTTAAGGGAAATGGAGAATACATTTTCTCAAAGGAGAAGAACCCCATCTTTCTCTTCTTCCCT
TCTTGATGCAATTTGCCGTTCTTTTGATGAAC
CCAATTGCCGTGAAGAGGAAATTTTCAGTCAATGTAGAAAAACTACAGTGATCAAGAAACAAAGCATT
GTTAACTCTGTTTCTACCACCCGGCGTGATGG
GTTTGTTGAAGAAGATAAAGAGATTTTTGATCTTCGACGAGCTATTATGATTGAAAGCTGGATGGAGA
AACAAAGTACTCGTGGTTCTGTACACTGTAAT
TCTACTTCTGCTTCTTCGGATTCTAGCTCTGCAACAGGAAGAAATGGTGGAGTTTTCTCTCCCTCTGA
AGCTGAGTCTAGTGTCAAGGATTTAACCCCAC

AGAGAAACAAGCCAATTTCAGAAAAACAACAGAAGCCCAAGTGTGAAGGAGGATTCACTAAGACAAAG
CTACAAGCTTTGAGAATCTATGGCGAATTAAA
GAAAGTAAAGCAACCAATTTCACCAGGAGGTCGCATTGCTAGCTTTCTCAATTCTATTTTCCGTTCAG
GAACTGCCAAGAAAGCAAAAATGTGTTCGATT
GGGGACATGGGTGATGCGAGTTACCAGCGTAAATCAAAATCTGCATGTTCTTCAGTTACATCGTTTTC
AAATTCTTGTTTCAACAAAACGCCTCCTTCAA
GAGAGAAACAGAGCGATGGGAATAAAAGATCAGTAAGATTCTATCCTGTTAGTGTGATCGTTGATGAA
GATTCCAAACCTTGTGGCCATAAATGTATCTA
TGAAGATGATCCTGGGTTGATGCCAATGCCTGTGCCTAAAATTTCAAGGGGTTCTTCTGTAAAGCAAG
ATGCTGCTAAAGGAGGAAGTTATATCAGGAAT
TATCAGAAGAAGAATATCAGTGAATTTGATTTCAGGGGATTTCACAGTTACGTTGAAGACAGCAATGA
CGATGATAGCAGCTGCTCAAGCTCTGATCTTT
TCGAGCTGGATCATCTTATTGGGATTGGAAGGTACATAGAGGAGCTGCCAGTTTATGAAACTACAAGT
TTGAAAACGAATCAGGCCATTGCTAATGGCTT
CAGCCTGTAG

>Ptr_Potri.002G223400
ATGTACAAGAAGGAGAGATCTTCAAGGGAAAGCACATTCCATCCAAGAAGAAGAACCCCGTCTTTCTC
TTCAACTCTTCTTGACTCTATTTACCGTTCGA
TTGACGAATCCAACGGTGAAGAACAGCATGTGCTGGGGATCAAGAAACAGAGTTGCAACTCTGTTTCT
ACAACCAGGCGTGATACCTCTTCAGATTCTAG
CTCTGCAGGAGGAGGAGGAAGCGGAGGAGGTGTGTTCTCGTCCTCCGAAAACGAGTCAAGTGTCAGAG
GAAACTCAAGTTCTTGCCAACAGAGAACAAAA
CCACTTTCAGACAAACCACATCAGAAACCGAAATGTGAAGGAGGAGGGTTTCATAAGACGAAGCTAAG
AGCATTAAAAATCTACGGTGAATTAAAGAAGG
TAAAGCAGCCGATTTCACCAGGTGGTCGCATTGCAAGCTTTCTAAATTCTATTTTCAATTCAGCAAGT
GCTGCAAAAAAAGTGAAAATGTGCTCTATTGG
GGCCATGGATGATGTAAGTTTTGAGCGTAAATCAAAGTCTGCTTGCTCATCGGCTACTTCCTTTTCAA
GGTCCTGTTTGAGCAAAACACCTCCTCCAAGA
GGGAAACCAAGTAATGGCACAAAAAGGTCAGTTCGATTCTATCCTGTTGGTGTCATTGTTGATGAGGA
TTCAAGACCTTGTGGGCATAAAAGTATTTATG
AAGATGATCCAGGATTGATGCCAACGCCTCGAAAAGTTGTCAAAAGTAGTTCTGTCAAGGAATTAGAG
GTTGCTAAAGGAGCAGCAGCAGATTATTTAAG
AAGTTATCACCAAAGGAAGAATGTTAGTGAATTTGATTTCAGGGGTTTCCACAATTATGTTGCAGATG
ACAGTGACAGTGACGACGAGAGTTGTACAAGC
TCCGATCTTTTCGAGCTCGATCATCTTATTGGGATTGGAAGGTACAGAGAGGAGCTCCCAGTGTATGA
AACCACTAACTTCAAAACTAATCAGGCCATTG
CTAATGGCTTTTTCCCGTAA

>Ptr_Potri.006G222700
ATGTATAGGCGGGAGAAAACAATGAGAGAGGAAAGATACAAGCACGGCATCAAGAACCCATCTTTCTC
TTCATCACTTCTTGATGAAATCTATCGTTCTA
TTGATGATGGTGAACCAAAACGTGAAGAGTTGAAGTTCTACAGAGAAACAATGCCAAAGAAACAAAAC
AAAATTGGCAGGAACATTGGAGGAGAAGCAGG
GATGCCAACTCTTCAACGTGCTTGTTTGATCGAGAAATGGATGGAGCAGAAGGTCACCCAAAAGGTCA
TCACTCAACAGAGGCGGCAAAACTCAACAGAA
CTTGAGAGAAAAGCACAGCTTGATCATGATCTTGACCAAGATGTCCTGTTTTTTAGCTCAACCTCAAC
CTCTTCGGATTCTAGCTCTGGTGGGTTCTCAT
CTTCTGACACAGAATCTATGTACGGTGCAAGATCAAGGGCCTCTTCTTTCAATCCACCAAGACCTAAG
CCAGTCAGGACAAGCCTGTCCGCTCGGTCAGG
AAAAACAGAGAAAACAGAGAGGACTCTGTTCCATGAACAGAGGGAACTGCGTATGTTTGATGATTATC
ACTACAGCTCTGCTTCAGAACAGACACCAAGG
CTTGAAGACAACATAATCAAGTCCAAATCAAGAGCCTTAAAGATTTACAGCAATCTAAAGAAGGTGAA
ACAGCCAATTTCACCAGGTGGGAAGCTGGCGA
ATTTCCTCAATTCCCTCTTCACCACAGGGAACTCAAAGAAATCAAAGAATTCATCTTCCATAGGAAAC
TTTGATGAAGAAAGGAAACTTAACTCAGGACA

AGCATCGACCTGCTCTTCTGCTTCATCATTTTCAAGATCATGCTTGAGCAAGCATTCGCCATCGACCA
GGGAAAAATTACGCAATGGGGTTAAAAGGAGT
GTTAGATTTTACCCTGTGAGTGTAATTGTTGATGAAGATTGCAGGCCAGTCGGCCACAAATCATTATA
TGAAGAGGAAGAATCAAGTCTCATGTCTGTTT
CTCTGCCAACAGCATGGAAAATAGGGAAATCACCATCAAGAAAAACTGATGATGAGCTGAAGTATCAA
GTAATGGAGAAGAGTAGGAGAGTTGAGGAGGT
GGCTAGAGAGTTCTTGAAGGATCACCGTCAAAATCAGAAGAAAAATGATGTTACTATGATTGATGTTC
GCGGAAAATATAATGATCGCTATCACGATGAG
GATGAGGATGAGGATGACGATGCTGCAAGCTATTCGAGTTCGGATTTATTCGAGCTTGATCACCTTGC
AGTAATTGGAAACGATCGTAGGTATTGTGAAG
AACTTCCCGTGTACGAAACTACTCATCTTGATACTAATCGTGCCATTGCTAATGGCTTGATAGTGTAG

>Ptr_Potri.010G154200
ATGTCCATCACAGGACTCTCATCAGACCCTAGTAAGCCTTACAAGAAGTCCTTGCATCGGAGAAACAA
TTCTGATGAGCTTGATGTGTTTGAGGCAGCAC
GGTATTTCTCAGGGTACAATGAAGCAGGTGCGGGGTATAATGGTGCAACTTACACACAGAGAGTCATG
AGAGAAGATCATAAGAATTCTTGGAGGGGGAGG
AAGAATGAGCCTAGATGTACCAATGAGAAATCCGCTACCTCATCATATCCATCAACAGCCTCATACAG
TGGAGAAGCAAATACTGAAAGAGAAGAAATAC
AAGCAACCAAGCTCTCCAGGTGGTAGACTTGCCAGCTTCTTGAATTCTCTCTTTAATCAAACAAGCTC
TAAAAAGAAGAAATCAAAGTCCACCACACAAT
CAATGAAAGATGATGATGAGAGCCCCGGTGGAAGGAGGAAAAGGAGGAGCAGCATTAGTCATTTTCGA
AGCTCAAGCACTACTGATACAAAGTCTTTGTA
TTCTTCTTCAAGCTCTGGTTTTAGGACACCCTCTCCTTATACACACCCTCCAACAAAGGGCTGCAAGG
AGTCCAGAAGCTATTCAGATCACAAGCAAATA
GTTTCCTTGTCAAAGCAGAATGGGACTGTGAAGTCCACAACTTTCCAAAATGAGATATCGGATGATAA
AAAGAAGTCAGGTTTCTCTTGGCTGGAGGAGA
GATACAAATTCGTCAATGATGGGTTTTCAGATCAGAAAGCAGCCAAGAATCGCGGTAACCAATATTTG
GAGAAAGACAGGACTTGGGTTGATGAACATTA
TCGATCAGAGGAAAACGAATTTAGGAAGTTCAATGAGGTGGATGATGGAGCTGAGAGTGATTCAAGCT
CTGATTTGTTCGAACTGCAAAACTGTGACTTG
GGTATCTACTCAAATGGTCTGCCCGTGTATGAAACAACACATATGGATAGCATCAAAAGAGGAGCAGT
TCCAATTTCAAATGGCACCCTATGA

>Ptr_Potri.018G049900
ATGCATAGGTGGGAGAAAACAATGGAGGAAAGATACAACAAGCATGATATGAAGAACCCATCTTTCTC
TTCATCTCTTCTGGATGAAATCTACCGTTCTT
TTGATGATGGTGAACCAAAACATGAAGAGTTGAAATTCTATCGAGAAACAATGCCGAGGAAACAAAAC
AAAGGTACTAGTGGGATTAAGATTGAACGAGA
AGAAGGGATGTCAGCTCTTCAACGAGCTTGTTTGATCGAAAAATGGATGGAACAGAAGGTGAGTCAAA
AGCTCATCACTCAACAGAGGAGGCAAAAACTG
ATAGAATTCGAGAGAAAATCACAGCTTGATCATGATCTTGACCAAGATGTCCTGTTTTTTAGCTCCAC
TTCAACCTCTTCAGATTCTAGCTCTGGTGGGT
TCTCATCTTCTGATACAGAATCTATGTATGGTGGGTTCTCAAGGGCTTCTTCTTTCAATCCACCAAGG
CCTATGCCAGTCAGGACAAGCGCGTCGGCTCG
GTCAGGAAAAACAGAGAGGACTCTGTTTTATGAACAGAGGGAAGCGCATATGTTCGATGATTACCACT
ACAATTCTGCTTCAGAACAGACACCAAGGTTC
GAAGAGAGCTTAATCAAGCCCGAATCAAGAGCCTTAAAGACTTACCGCAATCTAAAGAAGGTGAAACA
GCCAATTTCACCGGGTAGCAAGCTCGCAAATT
TCCTTAATTCCCTCTTCACCACAGGGAACACAAAGAAATCAAAGAATTCACCTACCAATGGAAACTTT
GATGAAGAAAGGGAACTTAAATCAGGGCAAGC
ATCAACCTGCTCTTCTGCTTCATCGTTCTCAAGATCCTGTTTGAGCAAGCATTCGCCATCGACAAGGG
AGAAATTACGCAATGGGGTTAAAAGGAGTGTC

AGGTTTTACCCTGTGAGTGTAATTGTTGATGAAGATTGCAGGCCAGTCGGACACAAATCATTATATGA
TGTGGAAGAATCAAGTCTAAAGTCTGTTTCTC
TGTCAACAGCATGGAAAATTGGGAAATCACCATCAAGAAAAATAGATGATGAGCTTAAGTACCAAGTA
GTGGAGAAGAGTAGGAGAATTGAGGAAGTGGC
GAGAGTGTTCTTGAAAGATTATCATCAAAATAAGAAGAAAAATGATGTTATTAAGATTGGTGCTGGCG
GAAAGTACAATGATCGTTTCGAGGATGAAGAT
GAAGATGAGGACGAGGATGATGATGCTGCAAGTTATTCGAGTTCGGATTTATTCGAGCTTGATCATCT
TGCAGTAATCGGAAAAGATCGTAGGTATTCTG
AAGAGCTCCCTGTGTATGAAACTACTCATCTCGATACAAATCGTGCCATTGCTAATGGCTTGATGGTG
TAG

>Ptr_Potri.018G065000
ATGTACAAGAAGGAGAGATCTTCAAGGGAAACCACATTCCCTCAGAGAAGAAGGACCCCATCTTTCTC
TTCGACTCTTCTTGATTCTATTTACCGTTCGA
TTGATGATTCCAACGGCGAAGAACATGTGTTGGGGCGATACTCTAGAGAAACAACCATGATCAAGAAA
CAGGGTTGCAACTCTGTTTCAACAACCAGGCG
TGATGCATCATTTCTTGAAGGGGAGAAACAAGCATCTCCTACTCTTCGTCGAGCTGTCATGATTGAAA
GCTGGATGGAGAAGCGAAGCTCTCGTGGCTCT
AAGCATTATAACGCGACCTCAAGCTCTTCAGACTCTAGCTCTGCAGGAGGAGGGGGCGGAGGAGGGGG
AGGAGGAGATGTGTTCTCGTCCTCCGAAAATG
AGTCCAGTGTCAAAGAAAATTCGAGTTTTGCCCAACAAAGAACCAAACCACTTTCAGATACGCCACAA
CTGAAGCCAAGGTGTGAAGGCGGAGGCTTTAG
TAAGACAAAGTTAAGAGCATTAAAAATCTATGGTGAATTAAAGAAGGTAAAACAGCCAATTTCACCAG
GTGGTCGCATTGCAAGCTTCCTAAATTCTATT
TTCAATTCAGCAAGTGCTGCAAAGAAGGTGAAAATGTGCTCTATTGGGGCCATGGAAGATGTAAGTTT
CGAGCGTAAATCAAAGTCTGCTTGCTCATCGG
TTACTTCCTTTTCAAGGTCTTGTTTAAGCAAAACACCCCCTCCAAGAGGGAAACCAAGCAATGGCACA
AAAAGGTCAGTTCGATTCTATCCAGTCAGTGT
CATTGTTGACGAGGATTCTAGACCTTGTGGGCATAAATGTATTTATGAAGATGATCCAGGATTGATGC
CAGTGCCCCAAAAAGCTGTCAAGAGTACTTCT
GTCAGGGAATTAGAGGTTGCTAGAGGAGCAGCAGCAGGTTATTGGAGAAGGAGTTGTCATCAAAAGAA
GAGTGTTAGTGAATTTGATTTCAGGGGCTTTC
ACAGCTATGTTGAAGGTGATGGTGATAGCAACAGTGATGATGATGAGAGTTGTTCAAGCTCTGATCTC
TTTGAGCTGGATCATCTGATTGGGATTGGAAG
GTACAGAGAGGAGCTCCCAGTGTATGAAACCACTAATTTCAAAACTAATCAGTCCATTGCCAATGGCT
TCATTCTGTAA

>Aca_Aqcoe3G372500
ATGTCGATCAAAGGCTTTTCCCAAGAAAAAGTTCATAGAAAATCCACTCAAAGAAGGGATGGCTCAGG
CGAGCTGGATGTTTTTGAAGCAACTAGCTACT
TTTCAGGCTGTAATGAAGCTGCTGGCTATAATGCCAACTTATTTTCACAGAAGGAACTGAGAGAAGAA
AGACAAACATTGGGTGGGAGAAGGATGAGTTT
AGACATGCCATCATCAGCTTCTCTTCCTTCACAATCTCATCGAGTTGAAAAACAAACAAAAGAGAAGA
AATACAAGCAACCAAGCTCTCCTGGTGGTAAA
CTTGCTAGTTTTCTAAACTCTCTTTTTAATCAAGCAGCTTCTAAGAAGAAGAAATCAAAATCAATGAA
AGATGAAGAAGAGATTCCTGGCGGTGGCAGAA
GGAAGAGGAGGAGCAGTATTAGTCTTTTCCAAAACATCGGCAATAATGATTCCAGATCTTTATATGCA
TCGTCAACTGCTTCTGGTTTCAGAACACCACC
TGCGTATATAGATACCCCTACAAAAGCATATAAAGATCCAAAAGGCTACTCAGATCACAAATATGTGG
TCTCATTACCAAAGTATACTGGGAAGGTGAAC
TCTGTTAGTTCACAAGATGAAGCTGTGGATGAAAAGAAAAACATGGATTTATCTTGGTTGGATGAGAA
ATTCAGATTCATCGATGAGTTTTCAGAGAAAG
ATAAGAATATGTTTAAGACAGACAGGATATATGTTGATAAGTTTGATAAGTTTTCATCGGAGAAGAAC
AATTTTGTTAAAAAGCAGGCTGTTGTTGATCA
TGAGGTGGATGATGGTGCAGAAAGCGATTCAAGTTCTGATTTATTTGAATTGGGGAATTATAATCTTG
GTTCTTATCCTAATGATCTACCTGTGTATGAA

ACCACTCATATGGAGAGGATTAAGAGAGGACCACCCATTACTAATGGTCTCTTATAG

>Aca_Aqcoe7G116200
ATGGATCAGTGGACTAAATCGTTAAGAGAAGACAAAGTTCGAACAAGAAGTAGAAATCCTTCTTTCTC
TTCTTCACTTCTTGATGCAATATATCGTTCTA
TTGACGAAGGTGAAGAAGATTTAGTACTTTATAAAGAAACTATGAAGAAGAAACAAACTACTAGTACT
ACTACTACTAATAGTTTGAAAAGAGATGAAAA
AAATATTGATGATTTTACTAGAGCTTGTTTGATTGAGAAATGGATGGAAAAGAAAGTAAGTGAGAAAG
TTGTTGTTAAACGACAATCTGTTCGTGATTTT
GATCAGAAGTTGCAGAACAATCGTTCTTCATCAACTGCTTTTCATTCTGCTTCAAGTTCATCTGATTC
AAGTTCTGCTGGAGGTTTTTCTTCATCTGAAG
CAGAATCATTGTATGGTTCAAGAATTCAAAGACCAAAGCCGATTCGAACAAGTGTTTCTGCTAAACCA
GAGAGTTTTGAAAAGCCTTTGCAGTATGAACA
GAGGAAATCAACAAACATTTATGAATCTCATTATCATCATCAACATGAAAGAATAAGCTCTGTTCCTC
AGAAAGTGAAACAAGAAGGCGGTTTCAACAAG
ACGAAATCCAAAGCTATGAAGATCTATGGTGATCTTAAGAAGGTAAAACAACCAATTTCACCAGGTGG
AAGACTTGCTACATTTCTAAATTCATTGTTCA
CTGCTGGAAATACGAAGAAAGCGAAACTTTCTAATTCTGTTAATGGAATTGAAGATTTGAGGTCTGAA
AGAAAATCAAAGTCCACACAAACATCTACATG
TTCTTCAGCTTCTTCATTTTCAAGGTCTTGTTTAAGCAAAACTCCATCTTCAAGAGGAGCAAAATCCA
CTAATGGTACTAAAAGATCAGTTAGATTTTAT
CCTGTTAGTGTGATTGTTGATGAAGATAGTCGTCCTTGTGGACAAAAATCTATTTATGAACAACAACA
GCAGCAGCAGCAATCAAAAGTGGTTTCAATGA
ATGAAGAATTCAAGTATCATTTAATGGAGAAAAATCGGAAAATCGAAGAAGCAGCAAGAGAGTTTCTT
AAAAGTTATCAAGATAAGAATCTTGAAAAGAA
AAAGTGTGATTATGAAATGAGAGATGTTCATCAAGACTACGAAGACGACGATGATGAAGATGCAGAGA
GTTATGCAAGTTCTGATTTATTTGAATTAGAT
AATTTTGCTTCAATTGGGAATGAGAGGTATAGAGAAGAACTTCCAGTATATGAAACTACACATCTCGA
GAAAAATCGTCCCATATCTAATGCATATATCT
TGTAA

>Gma_Glyma.01G063800.1
ATGTCCATAGCAGGCCTTATAGACCCAGAAATGAATCACAACAAGTCCTTCCACCGGCGAAACGACTC
TGGCGAGCTCGATGTGTTTGAGGCAGCAAGGT
ACTTCTCAGGATACAGTGAAGTTGTTGGCTACACTAGTGGCTCCACCACCTACACTCAGAAGATCATG
AGAGAAGAAAGGCACCATCATGGACAAAGAGC
TGCCAGAATCAGCTTAGACATGCCAATGAGAAGCTTGCTCCCCCAACAATTCCATGGCATGGAGAAGC
AAATCATCATGAAGGAGAAGAAGAACAAGCAG
CCTAGCTCTCCAGGTGGAAGGCTTGCAAGCTTCTTGAACTCTCTCTTCAGCCAATCAGCATCAAAGAA
GAAGAAGTCAAAGTCAGGCTCACAATCCATGA
AAGATGAAGATGAGAGCCCTGGTGGAAGGAGAAGGAGAAGAAGCAGCATTAGCCATTTCAGAAGCTCA
AGCACCGCAGATTCAAAGTCCTTGTACTCCTC
CTTGAGTTCAGAGTTTCGAACTCCTCCTTATGTACAAACACCAACAAAGAGCTGCAAGGAATTCAGAA
CCTTCTCATCAGAAAAAAAGCATGCACTGTCC
TTTTCAGCAAAGTACAACAACAACAATGGACAACAAATAAGATCATCAACAACAACCACTTTGCAGAA
TGAGTTGTTGTGGGATGAGAAGAAAAAGAGGG
AACCAACAACAACAACAACAACAACCATGTTAGATGATAATAGCAACCACAAACACTTATCAGAGAAA
CAAAGGAACAACAACAACAACAAGGGAAATCA
TGAGTTATTACTTGAGAAAGATAGGATCTTAGTGGACAACAAGTACTCATCAGAAGAGAAGGAAACCA
CCACTCAATTTAAGAACTTAAATGAGGTTGAT
GATGGTGCAGAAAGTGATTCAAGTTCTGATCTGTTTGAATTGCAAAACTATGACTTGGGATACTATTC
AAGTGGTCTACCTGTCTATGAAACTACCAACA
TGGATAGCATCAAGAGAGGAGCACCAATTTCCAATGGCCCTCTGTGA

>Gma_Glyma.02G121400.1

ATGTCCATAGCAGGCCTTATAGACCCAGAAATGAATCACAACAAGTCCTTCCACCGGCGAAATAACTC
CGGCGAGCTCGATGTGTTTGAGGCAGCAAGGT
ACTTCTCAGGATACAGTGAAGTTCTTGGCTCCACCACCACCACCTACACTCAGAAGATCAATATGAGA
GAAGAAAGGCACCATCATCATCATCATCATGG
ACATAGAGCTGCCAGAATCAGCTTAGACATGCCAATGAGAAGCTTGCTCCCACAGCAATTCCATGGCA
TGGAGAAGCAAATCATCATGAAGGAGAAGAAG
CACAAGCAGCCTAGCTCTCCTGGTGGAAGGCTTGCAAGCTTCTTGAACTCTCTCTTCAGCCAATCAGC
ATCAAAGAAGAAGAAGTCAAATAAGTCAAGCT
CACAGTCCATGAAAGATGAAGATGAGAGCCCTGGTGGAAGGAGGAGAAGAAGGAGCAGCATTAGCCAT
TTCAGAAGCTCAAGCACTGCAGATTCAAAGTC
CTTGTACTCCTCCTTGAGTTCAGGGTTTAGAACTCCTCCTTATGTACAAACACCAACAAAGAGCTGCA
AGGAATTCAGAACCTTCTCTTCAGAAAACAAG
CATGCACTGTCCTTTTCAGCAAAGTACAACAATAACAAGAACAACAATGGACAACATGTAAGATCATC
AACAGCAACCACCACTTTGCAAAATGAGTTTT
TGTGGGATGAGAAGAAAAAGAGGGAACCAACAACAACAACAACCTTGTTGGATGATAATAGCAACCAC
AAACACTTATCAGAGAAACAAAAGAACAACAA
CAACAAGGGAAGTCATGAGTTATTACTTGAGAAAGATAGGATGTTAGTGGACAACAAGTACTCATCAG
AAGAGAAGGAAACCACCACTCAATTCAAGAAC
TTCAATGAGGTTGTTGTTGATGATGGTGCAGAAAGTGATTCAAGTTCTGATCTGTTTGAATTGCAAAA
CTATGACTTGAGATACTATTCAAGTGGCCTAC
CTGTCTATGAAACTACCAACATGGATAGCATCAAGAGAGGAGCACCAATTTCCAATGGCCCTCTGTGA

>Gma_Glyma.04G055100
ATGTACACAACAAAGAGAGAACAACAAAGGCTCAGGTTCCAAAACCACGCTAAAAAACCCTCCTTCTC
TTCCATTCTACTCGATCAAATCTACCGTTCCA
TCGACGAGGGCAACGACATGAAGCTCTACAACGAAACAATGGCGAAGCAACAGAACAGAGTCTTCGTC
GAAGAAGAAGAAGAAGAAGAAGAGATAACGGC
TGCCAGCATTCGCCGCGCTTGTTTGCTCGACAAATGGAAAGAAAGCGAAAAGGCTGGAAGTCAATGGA
AAACACAAACGGAGGGAAAATCGCGTTGGCAT
CATCATCATAATCACGAACATGACCAGGAGGTTATGTTCTTCAGTTCAACTTCGAGTTCCTCGGATTC
TAGTTCTGGTTTATTGTCCTCCTCTGACACGG
AATCCTTGTACGGAATGAGGTCGAAATCACGAGTTTCGTGCTTTGCGCCGTCGAGGCCGAAGCCAGTG
GTGACGTCAGCGAGCAACGAAGTGGGTTTAAT
AAAGTCAGAATCGAGGGCATTGAAGATTTACAACAACCTGAAGAAGGTGAAGCAGCCCATCTCACCGG
GGGGTAAGTTAAGTAATTTTCTTAACTCTCTG
TTCGCGACAGGGGGCAGCGTTAAGAAAACCAAAACCTACGACGACGACGCCAAAGCTTCCACCAAGTC
GGGACAGGACTCCACGTGTTCCTCCGCTTCCT
CGTTCTCGCGGTCTTGTTTGAGCAAGGCTTCGACTTCCTCTAAGGACAAATTGCGTGACGGGGTTAAG
CGAACGGTGCGTTTCTACCCCGTGAGTGTGAT
CGTGGGCGAGGATTCTCGAGCGTGTGGGCATAAGTGTTTGTATGAAGAGGACACTCGCGTAACGGAAG
TGTCCGTGCCAACCGCGTGGAAAATTGGGCGC
AAGAAGAACCAAGAAGATGAAGAATTAAGGGTCGTGGATAGGAGTAGGCGCGTGGAGGAGGCTGCTAG
AGAGTTCTTGAAAGAGTACCATCGAAGCCAGA
AGAAGAGCGATTTTACTAATTTGGATGTGGAAGATGATGGTGATGCATCGAGTTGTTCAAGTTCGGAT
CTGTTCGAGCTTGATCACCTAGCGGTGATGGG
AAACGATAGGTATGGTGATGAGCTTCCCGTTTACGAGACAACTTATGTTTCTACTAATCGCGCCATTG
CTAATGGCCTCATATAA

>Gma_Glyma.06G055200
ATGTACACAACAAAGAGAGAACAACAAAGGTTCAGGTTCCAAAACGACTCTAAAACCCCCTCCTTCTC
TTCAACTCTACTCGATAAAGTCTACCGTTCCA
TCGACCAGAGCAACGACATGAAGCTCTACAACGAAACGATGGGGAAGAAACAGAGCAGAGTCGTCGTC
GAAGAAGAAGAGATAACAGCTGCCAGCATTCG
CCGCGCTTGTTTGCTTGAAAAATGGAATCGAAGCGAAAAGGTTGGAACTCAATGGAAAACACAAACGC
GTTGGCATCATCATCGTCATCACGAACATGAC

CAGGACGTTATGTTCTTCAGTTCAACTTCGAGTTCCTCGGATTCTAGTTCTGGTTTGTTGTCCTCCTC
TGACACGGAATCATTGTACGGACTGAGGTCGA
AATCGCGATTTTCGTGTTTCGCGCCGCCGAGGCCGAAGCCAGTGATGACGTCAGCGAGCAATGAGGAG
GGTCTAAGAAAGTCAAAATCGAGGGCGTTAAA
GATTTACAACAATCTGAAGAAGGTGAAGCAACCCATCTCACCGGGGGGTAAGTTAAGTAATTTTCTTA
ACTCTCTGTTGGCGACAGGGGGGAGCGTAAAG
AAAACCAAAACCTACGACGACACGAAAGCTCCCAAGTCGGGACAAGACTCCACGTGTTCCTCCGCTTC
CTCCTTCTCGCGGTCTTGTTTGAGCAAGGCTA
CACCTTCCTCCTCTAAAGACAAGTTGCGAGATGGAGTTAAGCGAACGGTGCGTTTTTACCCCGTGAGT
GTGATCCTGAACGAGGATTCTCGACCGTGTGG
GCATAAGTGTTTGTATGAAGAGGACACGCGCGTAATGGCGTGGAAAATTGGGCGCAAGAAGAATGAAG
ATGAAGAATTAAGGGTCGTGGATAACAGTAGG
CGCGTGGAGGAGGCTGCTAGAGAGTTCTTGAAAGAGTATCGTCGAAGCCAGAAGAAGAGCGATTTCAT
TAATTTGAGGGATTTTACTAATTTGGATAATG
ACGATGATGATGATGATGCAGCGAGTTGTTCAAGTTCGGATCTGTTCGAGCTTGATCACCTAGCGGTG
ATGGGAAATAATAGGTATCGTGAGGATCTTCC
CGTTTACGAGACTACTTATGTTAGTACTAATCGCGCCATTGCTAATGGCCTCATATAA

>Gma_Glyma.07G036700
ATGGACAAGTGGGACAATAAACCCTCAAGAAAACAGCACCACAGAGAAAACCCTTCTTTCTCTTCCAC
TCTACTTGACGTTATCTACCGTTCCATCGACG
AAGATCCAACGGATGAGAAGGAAGAAGCCCAACTCATCTTCTACAGAGAAACCATGAGGAATCAGAAA
CAGAGCAATTGTTTCAGAGAAGAAAAAACCTGA
AGCTGAGAAACACAACTCTCGCAGGGCCAGGAAGGTGGAGAATTGGATGGAGAAGAAAGCCAACGAGA
AGGTTGTAATGGGGAGAAACTCATTGACGGAA
TTTGAACGAAGAACGCGAAGCAATTCAATTTCAAACACACTCTCCATGTATTCAAGCTCAACCTCTTC
TGAGTCAAGCTCTGTTGGAGGCTTCTCTTCTT
CAGAGTCAGAGTCCTTCTATGGAGTGCAAAGGCCAAAGCCAATTAAAACCAGTGTTTCTGATAAAACC
AAAACCAAAACAACCTTCGATGCTTCACTTCA
CAGTCACAACTTCAGGAGCCACTCTTCCCAAAGCCAAAAGCCAAAGCATGAGAACGGTTCTGGCAAAA
CCAAGTCCAAAGCCTTGAAAATCCTCTATGGT
GAGTTGAAGAAAGCAAAGCAACCAATTTCTCCAGGTGCAAAACTTGCTAGCTTCCTCAACTCTCTCTT
CACTTCAAGTGGAAATGCCAAGAAAGCAAAGG
TTTCAACAACAACAACAACAACAACTACGTCTACTTATCGTCCCGTCCTGATCCCGGTTGCGACAGAT
CGTACTGCTGATACTAAATCAGCAGCACAACA
ACAACAACAACCTGGTTCCACTTGTTCATCAGCATCTTCTTTTTCAAGGTCTTGTTTGAGCAAAACCC
CTTCTTCAAGATCAGGTGCAAAAAGGTCAGTG
AGGTTTTGCCCAGTGAGTGTCATAGTGGATGAAGATTGTAGGCCTTGTGGTCACAAAAATCTTCATGA
AGGTGAAGAGAGCAATGGAAAGAACAGAAGTG
AGGAACTTAGGCTGCATGTCATGCAGGAGAGTCGCAGGGTGGAGGAGTTAGCAAGAGACTTGTTGAAG
AATTATCAGAAAAAGAGTGAAGTGGAGTTTGA
TGATGTCATGCATTATGAAGATGAAGAAGAAGAAGAAGATGATGATGATGTGGCTAGTTGTTCAAGTT
CTGATCTTTTTGAGTTGGATAATCTATCAGCA
ATTGGGATTGAAAGGTATAGGGAAGAGTTGCCTGTGTATGAAACTACCCATTTCAATACCAATAGAGC
CATTGCCAATGGCTTCATTCTGTAA

>Gma_Glyma.13G108300
ATGCACGAGAGATCGATGAAAGAAGCTGCTGGCACGTGCCCACAAAGGAGAAGAACCCCTTCCTTCTC
CTCTTCTCTTCTCGACGCCATTTACCGTTCCA
TCGACGAATCAAAATCCAACCTTCACGACGATCAACAACTGGGTCTCCACCACCACGATCAAACCACA
CACAGTTTCACCTCTGAAAAAGGTGGCAAAAA
AGAAAGAATGAATCTTCGCCGAGCCGTGATGTTGGAAGATTGGATGGAGAAGCACGGTTCCCATTCCC
TCAATGCTCAGTTGCTAAACTCGAGTTCAAGC
TCCTCAGAATGCAGCTCTGCAGGGGGCATATTCTCCTCTTCAGAAACAGACACAACAACAACCCTAAC
GTTGAAGAAACAAAGACCAGCAAGGCTCACAT

CGGAGAAGAAGAAGAAGAAGCAGGACCGTATTATGAGTTCAGAAAAGCAGAACAAAGAAAGTGGTTTT
ACAAGGACCAAGTTGAGGGCGTTGAAAATTTA
CGGCGAATTGAACCAGAGAGTGAAGCAACCCATTTCACCGGGGAGTAGAATAGCTAGCTTCCTTAGCT
CCATCTTCAACTCACAGAACGTGAAGAAAGCC
AAAATGTGTTACGCTGGAGCCGTTGAAGATGTAAGCTTCGAGCACAAATCAAATTCCCCTTGTTTCTC
TTCCATCCCCTCTTCCTTTTCTAGAAGGTCTT
GCATGAGCAAAACACCCTCTTCGGCTAAAAAATCCAACAACAACGAAGTCAAAAGATCCGTGAGGTTT
TACCCCGTTAGCGTAATCCTAGGTGAGGATTC
TGAACCACAATCTAGTTATCATAAATGTAACATCATTTACGAGAGTGAACCTAATCTTGGTGTGAGAA
GTTCTTCCATAAAGGAGCTGAAGAAGAACACT
GCTCGGGGAAATGAAAATGGAGCTGAAGAAGCAGCAGCGCGTGGTTTTGTAAAAGGTTATCGAAATTC
TGGTCAGGGTGAATTTGATTTTAGAGGTTTTT
ATGATGATGATGATGAAGATGATGATGATGATGTGAGTTGTTCAAGTTCAGATTTGTTTGAGCTGGAT
CATCTTATTGGAGCTGCAAGGTACCAAGAAGA
GCTTCCTGTCTATGAAACTACCAATTTGGAAACAAATAAAGCCATTGCTAGTGGTCTATGTTTGTAG

>Gma_Glyma.14G099400
ATGTACCATTTAGAGAAGCCACAAAGAGATGATAAACAGTTTCTAACTCCCTCTTTTTCTTCCACACT
CCTCGATCAAATTTACCGTTCGATCGACGAGG
GTGAAAGAAAAAACGGCGAAATTAAGTTCTACAGACACACCACAATGAGCACTAGCAAGAGACAGAGC
AGGAGCAATTCTAAATCGATGGACGCTGGAGA
CAGAAAAATTGTCCGCGCAAAAAACGGCAGAAAAAAACTGCACTGCGACGAGGACATCATGTTCTTCA
GCTCCACTTCGATTTCGTCCACGGATTCCTCC
AGCCTCGGATTCTCCTCCTCCGACACTGAATCCATCTCACGGGCCTCGTGTTTTGCGCCAAGGGTGGG
GCGTAGAGGAGGAGGGAGCGCGTCGTTCCGGT
CGGAGAAACAGGGAATGCGCGTGTTAGACGGTTTCTGTCGGAACTCCTCCCACAGGTCCAAACACGCG
CATTCCCCGTTACCCGTCTCGAGCCGTCACCA
CGTGTCCGTGTCGGAACAGCAACGACATGAACACGTGACGGCTTGCGACGAGGAGGCGCTGATGATCA
AATCCAAGTCGCGAGCGTTGCGGATTTACAAC
AACCTTAAAAAGGTGAAACAGCCTCTTTCCCCCGGCGGCAGGGTCACGAGTTTCCTCAACTCGCTCTT
CGCCAACACAAAGAAAACTACTTCTACAACTT
CCCGCTCGTGCGGTGAAGGGAATGCTCCTTCTTCTTCATCTTCATGTTATTATTCTTCCACGTGTTCC
TCTGCTTCTTCGTTTTCACGGTCTTGCTTGAG
CAAGACCATGTCTTCAGAAAGGGACAGGTTGCGCAATGGGGTGAAGCGAACGGTGCGTTTTTACCCCG
TGAGTGTGATCGTGGATGAGGATAGCAGGCCC
TGTGGGGACAAACGCTTGTGTGAAGAGGAAGAAGCTTCTGGAGAGTTCTTAAGAGAGTACAACCGACA
CAACTCCAAGATTAAGAGCAATGATAATTTGG
TCTTGAAGGACTTGCCTTTAAGGAAAACTAACCTTGTGGAGGATGGTGATGACAATGATGATCATGAC
GACGATGCATCGAGTTATGCGAGCTCGGATCT
CTTCGAGCTTGATCATTTGGCTGTGTTTGGAAGTGGAAGGTATAGTGAGGAGCTTCCGGTGTATGAAA
CTACTCATGTTAGTACTAACCGCGCCATTGCT
AATGGCCTCATAGTGTAA

>Gma_Glyma.16G006100
ATGGACAAGTGGGACAATAAACCCTCAAGAAAACAGCACCACAGAGAAAACCCTTCATTCTCTTCCAC
TCTTCTTGACGTTATCTACCGTTCCATCGACG
AAGATCCAACGGAGGAGAAGGAAGAAGCCCAACTCATCTTCTACAGAGAAACCATGAGGAAGCAGAAA
CAGGGCAATTGTTTCAGAGAAGAAAAACCCGA
AGCTGAGAAGCACAACTCTCGCAGGGCCAGGAAGGTGGAGAATTGGATGGAGAAGAGAGCCAGCGAGA
AGGTTCTCATGGGGAGAAACTCACTGACGGAA
TTTGAAAGAAGAACGCGAAGCAATTCAATTTCAAACACACTCTCCATGTATTCAAGCTCAACCTCCTC
TGAGTCTAGCTCTGTTGGAGGGTTCTCTTCTT
CAGAGTCAGAGTCCTTCTATTATGGAGTGCAAAGGCCAAAGCCAATTAAAACCAGTGTTTCTGATAAA
ACCAAAACCAAAACCAACTTCGATGCTTCACT
CCACAGTCACAACTTCAGGAGCCACTCTTCCCAAAGCCAAAAGCCAAAGCATGAGAACGGTTTTGGCA
AAACCAAGTCCAAAGCCTTGAAAATCCTCTAT

GGTGAGTTGAAGAAAGCAAAGCAACCAATTTCACCTGGTGCAAAACTTGCTAGCTTCCTCAACTCTCT
CTTCACTTCAACTGGAAATGCCAAGAAAGCAA
AGGTTTCAACAACAACAACATCTACTTATCGTCCCGTCCTGATCCCGATTGCGACAGATCGTGTTGCT
GATACTAAATCAGTATCAGCAGCACAACAACA
ACCTGGTTCCACTTGTTCATCAGCATCTTCTTTTTCAAGGTCTTGTTTGAGCAAAACCCCTTCTTCAA
GATCAGGTGCAAAAAGGTCTGTGAGGTTTTGT
CCTGTGAGTGTGATAGTGGATGAAGATTGCAGGCCTTGTGGTCACAAGAATCTTCATGAAGGTGAAGA
GAGTTTGGTGGATTCCCGTGGAAAGAACAGAA
GTGAGGAACTTAGGCTGCATGTCATGCAGGAGAGTCGCAGGGTGGAGGAGTTAGCAAGAGACTTGTTG
AAGAATTATCAGAAAAAGAGTGAAGTGGAGTT
TGATGATGTCATGCATTATGAAGATGAAGAAGAAGAAGAAGATGATGATGATGTGGCTAGTTGTGCAA
GTTCTGATCTTTTTGAGTTGGATAATCTATCA
GCTATTGGGATTGAGAGGTATAGGGAAGAGTTGCCTGTGTACGAAACTACCCATTTCAATACCAATAG
AGCCATTGCCAATGGTTTCATTCTGTAA

>Gma_Glyma.17G051100
ATGCACGAGAGATCGATGAAAGAAGCTGCTGGCACGTGCCCACAAAGGAGAAGAACCCCTTCCTTCTC
CTCTTCTCTTCTCGACGCCATTTACCGCTCCA
TTGACGAATCAAAATCCAACCTTCACGACGATCAACAACTGGGTCACCACCACCACCACGATCAAACC
TTAGCCACACACAGTTTCACCTCTGAAGAAAA
AGGTGGCAAAAAGGAGAGAATGAATCTTCGCCGAGCAGTGATGTTGGAAGATTGGATGGAGAAGTATG
GTTCTTCCCGTTCCCTCAATGCTCAGTTGCTA
AACTCGAGTTCAAGCTCCTCAGAATGCAGCTCTGCAGGGGGAATATTCTCCTCTTCAGAAACAGACAC
GACAACAACAACCTTGAAGAAACAAAGACCAG
CAAGGCCCACATCGGAGAAGAAGAAGAAGAAGAAGAAGAAGCAGGACCATATAATGAGTTCAGAAAAA
AAGAACAAAGAAGGTGGTTTTGCAAGGACCAA
GTTGAGGGCGTTGAAAATTTACGGTGAATTGAACCAGAGAGTGAAGCAACCCATTTCACCAGGGAGTA
GAATAGCTAGCTTCCTTAGCTCCATCTTCAAC
TCACAGAACGTGAAGAAAGCCAAAATGTGTTACGTTGGAGCCGTTGAAGATGTTAGCTTCGAGCACAA
ATCAAAGTCCCCTTGTTTCTCTTCCACGCCTT
CTTCGTTTTCTAGAAGGTCTTGCATGAGCAAAACACCCTCTTCGGCTAAAAAACCCAACAACAATGGA
GTCAAAAGATCCGTGAGGTTTTACCCCGTTAG
CGTAATCCTAGGTGAGGATTCTGAACCGCAATCTAATAGTCATAGTCATAATAAGAAATGTAACAACA
TTTATGAGAGTGAATCTAATCTCACTGTGAGA
AAGATTACTAGAAGTTCTTCCATAAAGGAGCTGAAGAAGAACACTGTTCGGGGAAAAGAAAATGCAAC
CGAAGAAGCAGCAGCTAGGGGGTTTTGTAAAAG
GTTACCGAAATTCTGGTAAGGGTGAATTTGATTTTATAGGTTTTTATGGTAATGGTGAGAATGAGAAT
GATGAAAATGATGATGATGATGATGTGAGTTG
TTCAAGTTCAGATTTGTTTGAGCTGGATCATCTTATTGGAGCTGCAAGGTACCAAGAAGAGCTTCCTG
TCTATGAAACTACCAATTTGGAAACAAATAAA
GCCATTGCTAGTGGTCTATGTTTGTAG

>Gma_Glyma.17G225300
ATGCCAATCATGTACAATTTAGAGAAGCCACAGAGAGATGATAAACAGTTTCTAACTCCCTCTTTCTC
TTCCACTCTCCTCGACCAAATTTATCGTTCCA
TCGACGAGGGTGAAAGAAAAAACGGCGAAACAAAGTTCTACAGACACACTACTATGAGCAGTAGCAAG
AGACACAACAGAAGCGATTCGAAATCCATGGA
CGCTGGAGACAGAAAAATTGTCGGCGGCGCGAAAAACAACAACAGAAAACATCTGCACCGCGACGAGG
ATGTCATGTTCTTCAGCTCCACTTCGATTTCC
TCCACAGATTCCTCCAGCCTTGGATTCTCCTCCTCCGACACCGAATCCATCTCACGCGCCTCGTGTTT
CGCGCCACAGGTGGGGCGCGGAGGAGGAGGAA
GCGCTTCATTCCGTTCCGAGAAACAGGGAATGCGCATCTTCGACAGTTTCTGTCGAACCTCCTCCCAT
AGATCGTCGGAACACGCGCATTCCCAATTCTG
CATCACAAGCCGTCGTCATCTGTCCGTGTCAGAAGATCATAAACAAATCAGACACGGACACAGACAGG
TGACGGCTTGTGACGAAGAAGAAGAGGCCTTA

ATGATCAAATCCAAGTCGAGGGCCTTAAAGATTTACAACAACCTTAAAAAAGTGAAACAACCTATTTC
CCCTGGCGGCAGGGTCACGAGTTTCCTCAACT
CGCTCTTCGCCAACACGAAGAAAACCACCACCACTACTACTTCTACAAGTTCCCGCACGTGCGGCGAA
GTGAATACTCCTTCTTCTTCTTCTTCATGTTA
TTATTCTTCCACGTGTTCCTCTGCTTCTTCGTTTTCACGTTCTTGCTTGAGCAAGACCATGTCTTCCG
AAAGGGACAGGTTGCGCAATGGGGTGAAGAGA
ACGGTGCGTTTCTACCCAGTGAGTGTGATCGTGGGTGAAGATAGCAGACCCTGTGGGCACAAACGCTT
GTGCGAAGAGAAAGAAGCTTCTAGAGAGTTCT
TAAGAGAGTACAATAACCGACACAACCCCAAGATTAAGAGCAATGATAAATTGGTCTTGAAGGACTTG
TCTTTAAGGACTACTAACGTTGATGTTGATGA
TAACGATGACGACGATGATGCATCGAGTTATGCGAGCTCGGATCTCTTCGAGCTTGATCATTTGGCTG
TGTTTGGAAGTGATAGGTATAGTGAGGAGCTT
CCGGTGTATGAAACTACTCATGTTAGTACTAACCGCGCCATTGCCAATGGCCTCATAGTATAA

>Vvi_GSVIVT01012248001
ATGTCTGTCGCAGCTCTTTCAGAGGCAGATAAAATTTACAAGAAGTCCTTCCACCGGAGGAATGATTC
TGGCGAGCTTGATGTTTTTGAAGCTGCAAGGT
ATTTCTCCGGCGGCAATGAAATTATTGGAAGAAGAAATCAAAGTCCACAGCACAGTCGATCAAGGATG
AAGAGGAGAGCCCGGGTGGGAGGAGGAAAAGG
AGGAGCAGCATTAGCCATTTCCGGAGTTCCAGCACTGCTGATTCCAAGTCCGTGTATTCTTCCTCAAG
TTCTGGCTTTAGAACTCCCCCTCCTTATGCAA
ATACTCCCACAAAGACCTACAAGGATCTCAGAAGCTATTCAGATCACAGGCAAGTGGTGCCTTGGACG
AGAAGAGAATCAAAGAGTTGGTTTGGCTGGAC
GAGAAATTCAAATTCAGCATTGGATGAGATTGATGCCGGTGCGGAGAGTGATTCAAGCTCTGATCTAT
TCGAGCTGCAGAACTATGACTTGGGTTGCTAC
TCAAGTGGCTTACCCGTATATGAGACCACCCACATGGACAGCATCAAGAGAGGAGCACCCATTTCCAA
TGGCCCCCTACCCCTATAATAATTCTTTCCTC
CACTTCTTTCTCTGTATGTACAGTTGGGAATGTCTGCTTGTCTTCCATTTCCGTTTTTTGTTTTTTAA
CCTTCAATTGCTTTTAGAGGTATGCTTGTGGA
GTGCTGGAGCATTGGAAGTCCACCACTGGAATTGCTTGATTAGACTGCGGGATGATTTAGAGACCTTC
CACTAA

>Vvi_GSVIVG01015116001
ATGACTGGGCCACTCCTAGGCCTGTCCACTGACTCACCGGTCACGTCCTCTTTAAGAACCCTCCCTCT
CTCTCTTTCGCGTTTGCTCTTCATCTATGTCC
ACCCAAAGCTCTACAGAGAAAGCAGAGAACAGTTGGAAAGAATGGAGAGGGTTCAGCAGAGACGAAAA
ACTCCATCTTTCTCTTCGTCTCTTCTTGATTC
TGTTCTTCGTTCCATTGACGAATCATCTGGCGAGCGGCAGCAACTCCTCATGTCGAAGAAGCAAGGCG
GTGCTGAGGAGGAGGAGATAGCGAGTGTGAGA
CGAGCGATGATGATCGAGAAATGGATGAGGAAGCAGAGCGGTGGAAGTTCCGTGAGTTCCGTGGTTTT
TAATTCCGCGGCCTCCGCCGGATCGGAGAGGT
CTAGGAAGGTTGCGAAATTGGTGGAGCAGAGAAATTCGGCGCCGAAACTGGGGGAGAATCATGGCGGT
CGAGGAGGAGGAGGCGGAGGAGGAAGCTTCTT
GAAGACTAAATCAAAGGCGCTGAAATTCTACGGCGATTTGAAGAAGGTGAACCAGCCAATATCGCCCG
GACGCCGGATCGCGAACTTTCTCAACTCTCTG
TTCAGCTCCGGGAATGCCAAGAAAGCCAAAATGTGTTCGTTTGAAACTGTTGACGACATGAGCTCAGA
GCGGAAAGTGGTGAAGTCAGTGCAGGAATCAA
CCTGTTCATCTGCCTCCTCGTTTTCTCGATCTTGTTTGAGCAAAACGCCTTCTTCTTCAAGAACCAAA
CTGAAGAGATCCGTGAGATTCTACCCGGTGAG
CGTCATCGTAGACGAAGATTGCCGTCCGTGTGGCCACAAATGCCTCTACGAAGACGATCCGAGCCTAA
TGCCGACACTTACCGCCAAAAACATTCCACAA
ACAGCTTGGCTCAAAGGAGAGCTCAATGAGAAAAACACTTCTCCAGCTAATATCGCAGACATGCGCCA
AATTGTAAGAGACTATCAGAAGAAGATTTCTC
ACGAATTCAAATTGGCAGATCTTTGCCACAATGAAGACGATGATGACGACGACGACGACGACGACGAC
GCTGAGAGCTATTCGAGCTCTGATCTCTTCGA

GCTCGATCACATTGCGATCGGAAGGTACCAGGAGGAGCTTCCAGTGTACGGAACCACTTATTTGGGAA
CAAATCATTCCCATCGCGCTTGA

>Vvi_GSVIVG01016780001
ATGGGAGCCAACCTGGCAGGGGTTGAGGTAGGATTTGTGGTGCACGCGCTTGAAGGGGATATAAGAGC
GAGTGCAGGAGAGAGACACAGAGCTCTCTTCT
CTCTTCTCTCTCCACAGGCGTGGCTTTTGGCTTTCATTTCCCTTCTCAGAGCCATCAAGCACCCATCA
ATGGCTGTGTCTGCTTTGTTTCCTTTCTCGTT
TGCATTCACACAAGGGAGTAGCATCATTCGAAACGCTGCGTTTCAACTCTCTCGCGTTTTCTCTCGTT
TTCTAGCTTGGGCCTTGGGGTTCTTCTATAAA
GAGATGCAGAGGTGGGATAAATCGCTCCGTGAGGATCGGTACCGGAACGGTAGAGAAAATCCGTCCTT
CTCGTCGACTCTGCTCGACGCGATTTACAGGT
CGATCGACGAGGGTGGTGAAGGGGAGGAAGAGCTGGTTCTGTACAGAGAAACTATGAGGAAGAAGCAC
ACCATCATGATCGAAAAATGGATGGAGAAGAA
GGTGAGCGAGAAGGTGGTGGTTCGGCGAAAGTCCATGGCGGATTTCGAGAGAAGGTCGCGGAACGATC
GCGATTCTTTTTTCCTGAATTCGACTTCGAGC
TCCTCCGACTCCAGCTCGGGAGGAGGATTCTCGTCGTCGGAGGCGGAATCTGTAAGAAATTACATTGC
GTCGCAGAAGCCAAAGCACGAAGGCGGTTTCG
TGAAGACGAAGTCTCGAGCGCTGAAGATCTACGGCGATCTCAAGAAGGTGAAGCAACCAATCTCTCCC
GGCGGCCGCCTCGCGAGCTTCCTCAACTCTCT
CTTCACAACCGGAACCGCCAAGAAAGCGAAGATATCCTCGTCGGAAGACTCTACACCAGAACGCAAAT
CAAAGTCCGGCCACACGTCCACATGCTCATCA
GCCTCCTCCTTCTCCCGCTCCTGCCTGAGCAAAACGCCGTCATCCAGAAGCAAACTCAGCAACGGCAC
GAAGCGATCAGTGAGATTCTACCCCGTCAGCG
TCATAAAAAACGAATCGTACCTGAGAGAAGCCGCCAACCATGAAGATTCCGACGACGATGCAGCGAGC
TGCGCCAGCTCAGATCTCTTCGAGCTCGATAA
CCTCTCAGCCATCGGAATCGATAGGTACCGCGAAGAGCTTCCAGTGTATGAAACTACTCGCATGGATA
CGAATCGAGCCATTGCCAGCGGCTTGATTCTC
TAA

>Mes_Manes.03G052400
ATGCATAGGTGGGAGAGAGCGATTAGAGAAGATAGATACAAGCTTGAGAGCAAGAACCCATCTTTTTC
TTCCACTCTTCTCGATGAAATCTACCGTTCCA
CCTGTGAAGCCGACACCAACCATGAAGATTTGAAATTTTACGGTGAAACAATGATGCCCATGAAGCAC
ACCAGAGGTTCTAGCGTAAAAGTCAGTAGAGC
CGTCGAAGAGAATAAAGAGATGGAAGCTCTTCGCCGAGCTTGTTTGATCGAGAAATGGATGGACAGCA
AGGTGACCCAAAAGGTAAGCACGCAACACAGC
AGAAAAAAGTTGACGGAATTTGAGAGAAAATTACAGCTTGAACATGATCTGGACCAGGATGCTGTGTT
CTTTAGTTCCACTTCTATCTCCTCAGATTCTA
GCTTTGGTGGATTCTCATCCTCTGATACTGAATCCTGCTACGGTGCAAGATCAATGGCTTCTTCTTCG
TTCTTCCCAACAAGGCCTAAGCCTGTAAGAAC
CAGCGTCTCAACTCGATCAGGAAAAACAGAGAAAACAGAGAGAAAAGTGAGTACTCTGTTTCATGAAC
AGACGCCGAGAGTTGAAGAGAACATAATCAAG
TCGAAATCAAGAGCTTTAAAGATTTACGACAATCTAAAGAAGGTGAAACAGCCAACTTCACCTGGTGG
CAAGCTTGCAAATTTCATCCATTCTCTATTCA
CAAATGGAAATACAAAGAAAGCAAGGGGTTCTTCTTCTGTTAGCAATTGTGATGAAGCCTGGAAATCG
AAGCCAAGACAGGCACCGTCAACTTGCTCGTC
CGTTTCATCATTTTCAAGATCATGCTTAAGCAAGAGTTCACCATCAACAAGGGAAAAGCTTCGCAATG
GGGTTAAAAGATCTGTACGATTTTACCCAGTT
AGTGTTATCGTCGACGAAGATTGCAGACCATGTGGACACAAATCCTTGTACAAAGAAGAACAATCATC
CAGTTTCATGTCTGTTTCTTTTCCAAAATCAT
GGAAAATTGGGAAATCTCCAACAAGAAAAGTTGATGATGAGCTCAAATACCAAGTCATTGAAAAGACA
AGGAAGGTGGGGGAAGTTGCAAGAGAATTCTT
GAAAGATTATCGCCAAAACCAGAAGAAAAACGATGATCTAATTATGAGGAATGATTTCTGCCATTACA
ATGATCAGTTCGGAGATGACGACGATGAAGAT

GAAGATGATGAGGATGATTCAAGTTGTTCGAGTTCAGATTTGTTCGAACTCGATCATCTTTCAGTAAT
TGGAAAGAATAAGTACTGTGAAGAGCTTCCTG
TGTATGAAACTACTCGTGTTAATACTAATCGTGCCATAGCTAATAGCTTAATAATGTAA

>Mes_Manes.04G143200
ATGGATAACTGGGAGAAACCAGTCAGAGAAGATCGCTTTCGACACAAACATAGACAAAATCCTTCTTT
TTCTTCCACTCTTCTTGATGCAATTTACCGTT
CTATAGATGAATCCAACGATAAAGGAGAAGAAGAGCTGATTTTCTACAAAGAAACTATGAGGAAGAAG
CATAGCAACGGTTTCAAAGGAGATGAAAGGAT
TGCAACTCTGCAAAAAGCTTGCATGATAGAGAAATGGATGGAAGAAAAAGTAAGCTATGAAAAGGTAG
CAGTTCGGAGAAAATCCGTAGGAGATTTCGAT
AAAAATGCTCCAAAGGATTTCGATCCTAAACCACCTCCTATGTTACTGAACTCCAGCTCTAGTTCCTC
AGAATCTAGCTGTGGAGGTGGGTTTTCTTCCT
CAGAATCTGAGTCTACTTATGGTCTCAGCTCATCAAGATCATCATCAATTTGCGCCATGCAAAGGCCT
AAGCCTATTCGAACCAGCGTTTCTGCACGGCC
GGAGAGATACGAAAGATGTGTAGATGAAATTTCTATGTATCATCATGAGAGAAAATATTCAAATTATG
CACCAACACAAAAGCCAAAACACGAGGGCAGT
TTTGGGAAGACGAAATCCAGAGCCTTAAAGTTTTATGGCGATCTCAAGAAGGTAAAGCAGCCTATTTC
ACCTGGGGGTCGGCTTGCAAGCTTTCTCAACT
CTCTTTTCACTGCGGGAAATGCTAAGAAAGCCAAGATTTCATCTTCGTTGTCGGGGTGTGAGGAAAGA
AAAATTAAGTCTGAGCAGACATCGAAATGTTC
TTCAACTTCTTCATTTTCAAGGTCTTGCTTAAGCAAGACTTCTTCCGCTAGAGGGAATAAATTGAGCA
ATGGAACAAAAAGGTCTGTGAGGTTCTACCCA
GTTAGTGTGATTGTGGATGAGGATTCTAGACCTTGCGGACACAAAAGTCTCTATGCAAATCATGAAGA
GACTTCAATGGCAGTCACTTCTACAAGAAATC
TCGCAAATGAAGAGCTCAAGTTACATGTCATGAACGAAAGTCGCAGGGTTGAGAAAGTTGCAAGGGAT
CTCTTAAAGAACTATCAGAAGAAGCAAGAGGA
GTTTGCTGCAAGAGAACTTTTTTGCAACGGAAATGGAGAGTCTAGTGCAGAAGAAGAAGAAGAAGATG
ATGATGATGAAGAAGTAGCAAGTTGTGCAAGT
TCTGATTTGTTTGAATTGGATAATCTTTCTGCTATTGGGATTGAAAGGTATCGTGAAGAATTGCCTGT
GTATGAAACAACACATCTTGATAAGAATCGAG
TCATTGCTAATGGCATAATTCTGTAA

>Mes_Manes.05G112100
ATGTCTATTACAGGACTCCCGGAAAAATCTAAACTTTACAAGAAATCATTTCATCGCAGGAATGATTC
TGATGAGCTTGATGTGTTTGAGGCATCGAGGT
ATTTCTCAGGATACAATGAAGCTTTGAGTTATAATGGTGCAAATTATGCAGAAAAAGTCATGAGAGAA
GATCATAGACATCTTTGGAAAGGAGGAAGAAT
GAGCTTAGACTTGCCAATGAGAAATCCACTACCTCAGCAGTCATACACAGTGGAAAAGCAAATATCGA
AAGAGAAGAAATATAAGCAACCAAGCTCTCCC
GGGGGTAGACTAGCCAGCTTCTTGAATTCTCTCTTCAACCAAACCAGCTCTAAAAAGAAGAAATCAAA
GTCTGCCACACAGTTAATGAAAGATGAGGATG
AAAGCCCCAGCGGAAGAAGGAAAAGGAGGAGCAGCATTAGTAATTTTCGAAGCTCAAGCACCACAGAT
GGGAAATCTTTCTATTCTTCTTCAAGTTCTGG
TTTCAGAACACCTCCTCCTTATGCAGACACTCCTACAAAGGGCTACAAGGACTTCAGAAGCTGTTCAG
ATCATAAACAAGTTTCCCTGTCAAAACACAAT
GGAAATACGAAATCTACAGCCTTCCAGAATGAGGTATTGGATGAGAAAAGGAACACAGATTTATCGTG
GATGGATGAGAAACTTAAATACGATGATGGAT
TTTCTGAGAAAACCAAGAGCCTTGGTCATCAATATTTAGAGAAAGATAGGATTTGGATTGATCAGTAT
CCACCGGAAGAGAAGGAATTTAAAAAATTCAA
TGAGGTGGATGATGGAGCTGAGAGTGATTCAAGTTCTGATTTGTTTGAGTTGCAAAACTATGACTTGG
GCATCTATTCAAGTGGTCTGCCTGTGTATGAG
ACGACACATATGGATAGCATCAAAAGGGGAGCACCAATTTCCAACAGCACCCTTTGA

>Mes_Manes.11G021800

ATGGATAAGTGGGAGAAACCAGTCAGAGCTGAAGATCGCTTTCGACATCACCATAGAGAAAACCCGTC
TTTCTCTTCCACTCTTCTTGATGCTATTTACC
GTTCGATTGATGAATCCAACGGTAAAGTAGAGGAAGAGTTGATTTTCGCTATGAGGAAGAAACACAGC
AATGGTCTGCAAAAAGCATGCATGATAGAGAA
ATGGATGGAGGAAAAAGTAAGCTACGAGAAGGTAGCAGTTGGGAGAAAATCCATGGATGATTTCGACA
AAAATACTCGAAAAGATTTCAGTCGTAAACCT
CCTGTGTTGCTCAACTCCAGCTCAAGTTCCTCAGAATCTAGCTGTGGTGGTGGGTTTTCTTCCTCAGA
ATCTGAATCTATTTATGGTCTCAGCTCATCAA
GACCATCATCAACTTACGCCATGCAAAAGCCTAAGCCTATTCGAACCAGCGTTTCTGCTCGGCCGGAG
AGATACGAGAGAGCTGTTGATGAAATTGCTGT
GTATCAGTATAATCATCATCGCGACACAAATTATGCGCCAACGCAAAAGCCGAAACACGAAGGCAGTT
TTGTGAAGACAAAATCCAAAGCCTTAAAAATT
TATGGTGATCTCAAGAAGGTGAAGCAACCTATTTCTCCTGGGGGTCGGCTTGCAAGCTTTCTCAACTC
TCTTTTCACTGCGGGAAATGCAAAGAAAGCCA
AGATTTCATCGTCTTCTGTGTACGAGGAAAGCAAGATAAAGTCTGAGCAGACATCGACATGTTCTTCA
GCTTCGTCATTTTCAAGGTCTTGCTTGAGTAA
GACTTCTTCCTCTAGAGGGAATAAATTGAGCAATGGAACAAAAAGGTCGGTGAGGTTTTACCCAGTGA
GTGTGATTGTAGACGAGGATTCTAGACCTTGT
GGACACAAAAGTCTCTATGGAAATCATGAAGAGACTCTAATGGCAGTCACAGCTACAAGAAATCTCAC
AAATGAAGAGCTCAAGTTCCATGTCATGAATG
AAAGTCGCCGAGTTGAGGAAGTTGCAAGGGATCTCTTAAAGAATTATCAAAAGAAGAAGCAAGAGGAG
CTTGCTGTAAGAGATCTTTGCACTGGAAATAG
TGAGTTATTAGAAGAAGATGATGATGACGACGAAGACGACGCAGCAAGTTGTTCAAGTTCTGATTTGT
TTGAATTGGATAATCTTTCTGCCATTGGGATT
GAAAGGTATCGCGAAGAATTGCCAGTGTATGAAACAACCCATCTTGATACTAATCTAGCCATTGCTAA
TGGCTTAATCCTGTAA

>Mes_Manes.16G084100
ATGCATAGGTGGGAGAAAGCCATGAGAGATGATAGATACAAGCACGAGGGCAAGAACCCATCTTTCTC
TTCCAGTCTTCTTGATGAAATCTACCGTTCCA
CTTGTGAAGGTGACACAAATCATGAAGATTTGAAATTTTACGGTGAAACAATAATGCCCAAGAAGCAC
AGCAAAGTTTCTAGTATCAAAAGCGGTAGAGC
CATTGATGAGGATAAAGAGGTGGAAGCTCTTCGCAGAGCTTGTTTGATCGAGAAATGGATGGATCACA
AGGTGAGCCAAAAGGTCAGCAGGCAACATAGC
AGAAAAAACTTGACGGAATTCGAGAGAAAATCACAGCTTGAACATGATATAGACCAGGATGTTCTGTT
CTTCAGCTCCACTTCAAGCTCCTCAGATTCTA
GTTCCGGTTTATTCTCATCGTCCGACACCGAATCCTTCTACGGTGCAAGATCAAGGGTCTCTTCATCC
GCCGCACCGAGGCCTAAGCCGGTGAGGACTAG
CGTCTCTGCTCTATCAGAAAAAATAGAGAAAACAGAGAAAAAAGGAAGGTCTCTGTTTTATGAACAGA
GAGAATTGCATATGTTTGGTGATTATCACTAC
AGTTCCGCTACGGAGCAAACTCCAAGAGTTGAAGAGAGCATAATCAAGTCAAAATCAAGAGCCTTAAA
GATTTACAACAATCTCAAGAAGGTGAAACAGC
CAATTTCACCAGGTGGTAAGCTTGCAAACTTCATTAATTCTCTATTCACAACTGGGAACACAAAGAAA
TCAAAGAATTCATCTTCCGTTAGCAACTGTGA
CGAAGACTGGAAATCAAAGCCTGCACAGACAACATCAACTTGCTCTTCTGCTTCATCGTTTTCAAGAT
CATGCTTAAGCAAGAATTCACCATCCACAAGG
GAAAAACTCCGAAATGGGGTTAAAAGATCAGTTCGATTTTACCCTGTTAGTGTAATTGTTGACGAAGA
TTGTCGGCCGTGTGGACACAAATCCTTGTACG
AAGAAGAGGAATCATCCAGTCTCATGTCTGTTTCTCTGCCAACTGCATGGAAAATTGGAAAATCACCA
TCAAGAAAAGTGGATGATGAGCTCAAATACCA
AGCAAAGGAGAAGAGTAAGAGAGTGGAAGAAGTTGCAAGAGAATTCTTGAAAGATTATCATCAAAATC
AAAAGAAAAATGATGATCTCATTATGAGAAAG
GCTGGCTGCAAATACAATGATCATTTCGAGGATGATGAAGAAGAAGACGACGATGATGATCTGAGTTG
TTCGAGTTCAGATTTGTTTGAACTCGATCATC
TTTCAGTAATTGGAAAGGATAGGTATTGCCAGGAGCTTCCTGTGTATGAAACTACTCATGTTGATACT
AATCGAGCCATAGCTAATGGCTTAATAATGTA

G

>Mes_Manes.16G097700
ATGTATAAGAAGGACAGGTCTTCCAGGGAAATGGAGAATACATTTCCTCAAAGGAGACGAACTCCATC
TTTTTCTTCTTCCCTTCTTGATGCTATTTATC
GTTCGATTGATGAACCCAATGGCGGTGAAGAGGAATTTTTCAGTCAGTATAGAGAAACTAAGATGATC
AAGAAACAGAGCACTGCCAAATCTGTTTCTAC
TGCCGCCCGACGTGATGCGTTTCTTGAAGCAGATCTTCGAAGAGATATCATGATTGAAAGTTGGATGG
AGAAGCAAAGCACTCGTGGTTCTCTGCATTCT
AATTCTACTTCCTCTTCTTCAGATTCCAGCTCTGGTGCAGGAGGAAGTGGCGGTGGAGTGTTCTCGTC
ATCTGAAGCAGAGTCGAGTGTCAAGGAAAATT
CAACAAGGGTATCAATCTTAACTCCACAGAGAATCAACCCACTTTCAGAAAAACAACAGAAGCCAAAG
TGTGAAGGAGGATTTACAAAGACAAAGCTACG
AGCACTGAAAATCTATGGAGAATTAAAGAAAGTAAAGCAACCAATTTCACCAGGGGGTCGCATTGCCA
GCTTTCTGAATTCTATTTTCAACTCAGGGAGT
GGGAAGAAGGTGAAACTTTGTTCCATTGGAGCCGTAGATGATGTGAGTTCCGAGCGAAAATCAAAATC
TGCATGTTCTTCAGTTACATCATTTTCGAGAT
CTTGTTTAAGCAAAACGCCACCTTCAAGAGGAAAACAGAGCAACAGAAGCAAAAGGTCAGTGAGATTT
TATCCTGTTACTGTGATTGTTGATGAGGATTC
TAGGCCTTGTGGTCATAAATGTATCTATGAAGATGATCCTGGGTTGATGCCAATGCCGATTCCTCAAA
AAATTGCCAAGAGTTCTTCTCTGAAAGGAGAT
GTTGTTAAAGGAGCAAATTACATCAGGAAGTATCAGAAGAAGAATATCAGTGAATTTGATTTCAGGGG
TTTTCACAGTTATGTTGAAAATCATGAAGACA
GCGATGATGAAGAAGAAGATGATGATGAGAGTTGCTCAAGCTCTGATCTTTTCGAGCTAGATCATCTC
ATTGGAATTGGAAGGTACAGAGAAGAGCTACC
AGTTTATGAAACTACAAGTTTGAAAACAAATCAAGCAATTGCTAATGGCTTAATGCTGTAG

>Mdo_MDP0000127468
ATGTATGTGAGGGAGAGGTTACCGAAAGAAGAAACATTTTTGAGGCGAAGAAGAAACCCATCTTTCTC
TTCTTCGCTACTCGACTCCATCTACCGCTCCA
TTGATGAATCGAGCGGTGGAGATGGAGATCAGGGTTATGTTCGAGAGTCGACTGCAATGGTCAGGAAA
CAGAGCAGTTCTACTAAAGGAGACAATGAGAA
GGTTAATCTTCGAAGGGCTATTATGATTGAGAATTGGGTCGAGAAGCAGAGCGTTCACAGCTCCATGT
TCTCGAACTCCGCTTCGAGTTCCTCGGAGTCG
AGCTCGGGAGCTGCATTTTCGTCTTCCGAAACAGATTCGAGCTACAGATCAAGAACAAAACCGAAGGC
GGTCGAGCAGAGGTTCGTGCAGTTCGAGGAGA
AGGAAAAGATTGAGAGTGGCGGAGGAAGTGCGTTTTCAAGGACGAAGCTACGAGCTTTGAAAATCTAT
GAAGAATTGAAGAAAGTGAAGCAACCAATTTC
GCCCGGCGGGCGGATTGTGAGCTTCATCAACTCGATTTTCAATTCCGGCAATGTCAAGAAGCCAAAAA
TGTGTTATGTTGGAGCTGTGGAAGATGTGACC
ATTTCAGAGAATGTGTCGAATTCGAAATCGGCTTGTCCTTCTTCTTCTGCTTCTGCTTCTACTTTCTC
AAGGTCTTGCTTGAGCAAACCGTCTTCAAGAG
CGAAAAAATCGAGCAATGGCACTAAAAGGTCCGTTAGATTTTACCCAGTGAGTGTGATTCTTGGTGAG
GATTCTCAGCCTCCAAACCACCTCAAATGTGT
GTTTGAAGAAGACCCAAGCTTGATGCCAAAACCCTCTTTTCAAAAATATGCAAGGGCTTGTCCTGGTA
ATTATGATAAGCTGATCCAATCGGGTAAGAGW
CGAACAGAAGACCTGCTCACATTTAATCGTAGCACAAAATCAACAAGTTACAGAACGACCGGGGCGGT
TAGTCAAAATTTGGTGAGAAGTTTTTGCGACA
ATGCAGATGATGAAGAAAGTGATCATGATGCTGAGAGCTGTTCAAGTTCTGATCTTTTTGAGCTGAAT
CATCCAGTTGGGGTTGGAAGGTACATGGAAGA
ACTTCCTGTGTATGAGACTACTAATTTCAGAACCAATCAAGCTATTGCCCAAGGGTTMTTGTAA

>Mdo_MDP0000135865
ATGGAGAGGTGGGATATCAAATCACTCCCAAAAAACCGAGAAGCCAGCTCCAACCGACAGAGCAGAAA
CCGAGAAGCCAGCTCCCACCGACAGAGCAGAG

ACAACCCATCTTTCTCCTCCTCCCTCCTCGACTCTATTTACCGTTCCATCGACGAGACCACCGCCGGC
AGCGAAGGAGTAGCTTCGAGGAGCGAACAACA
CCACCGCCTCATCTTCTACAAAGAAACCATGAAGAAAAAACAGAGCACCGCCGCCGCCGTCACTACCA
GCGGCCGACATGGCCATGCACGCTCTAAAGAA
GACCAGCAGCACGAAATCATGAATTTCCGACGAGCCTGCTTGGTGGAGAAGTGGATGGAGAAGGAGGC
TGAGAAGTCCGTACCTGTTCGACGAAATTCCA
TTACGGATTTCGAGACGAAAAAGTCGAGATACAGTCACCAGAACGAGTTCCTGACGAATTCTAGTTCC
AGTTCTTCGGATTCGAGTGGTGGGTTTTCTTC
CTCGGAGTCAGACTCCATGTACGGATCAAAGTCGAGGTCCTCATCGTCATGCTACAGCATGCACAGGC
CGAAGCCAATTCGAACCAGCATTTCGTCTGAA
AAGGTTCTGTTTGATGATCACCAGAGAAATCACCATCACAATTCACAGAAGAAGAAGCACGAGAATGG
CTTCATGAAGACGAAATCGAAAGCGCTGAAGA
TTTACGGCGATTTGAAGAAGGTGAAGCAGCCGATTTCGCCAGGTGGTCGGCTGGCCAGTTTTCTCAAC
TCTCTGTTCAATGCAGGGCACGTGAAGAAGTC
CAAAATTGATGATTTGGGTGCGGAGAGAAAATCGTCATCAAAGTCTGGACAAATGGGGTACTCAAACC
CCAGTAGTACTTGTTCGTCAGCCTCTTCGTTT
TCGAGGTCTTGCTTGAGAAAAGCGCCTCTTTCGAGAAGCGAGTTGAGTGGCATCAGCAGCAATGTTGA
TGCCGCGAAGAGGTCAGTGAGGTTTTGTCCTG
TGAGTGTGATTGTGGATGAGGATTGCCGCCCGTGCGGACAAAAGACTCTGCTTAAAGAGGAGTCGGAT
TTAATGGCCGTGGCTAGAGCTGCTGCGGCCAT
TAAACCTCCAACAAATGAAGATGTTGGAGTTGAGTGTTGTGTGATGGATGTTGATGAGGATGATGATC
ATCGACGTCGAATTGAGGAAGTTGCGAGAGAT
TACTTGATGAAGAATTATCAGAAGAAGCATGACGAAGATGATGAGGATGATGATGATGCAGAGAGCTA
TGCAAGTTCTGATCTCTTTGAGTTGGATACTG
CTGGGGTTGAAGAAAGGTATCGTGAAGAACTGCCTGTGTATGAGACTACCTTTTTCGACAGAAATCGA
GCCATTGCTAACGGTTTGATTTTGTAA

>Mdo_MDP0000156548
ATGTCTATTATTAGTACAGGGGTGCTCTCGGCAGACCCTGGAGCTGATAAGATACACAAGAAATCGTT
CCACCACAGGAACGACTCCGGGGAGCTCGATG
TTTTCGAAGCTGCAAGGTACTTCTCAGGATACATTGAAGCTCCCAGCAGCCATAACAACAACAYCAGC
AGCAACACTTCGGCATTTTCGCAGAGGATGAT
GAAAGAAGACAGATCTTCYTGGAGAGGAGGCAGAATCAGCCTTGATATGCCTATAAGACACATGCTCC
ATCATCCTCAGCAAAACCCTAATCACCATAAC
CACGTGGCGGTGGAAAAGCAAAACAACATCAAAGACAAGAAATACAAGCAGCCGAGCTCTCCAGGCGG
GAGACTAGCCAGCTTCTTGAATTCTCTATTCA
ACCAATCAGCATCCAAGAAGAAGAAGTCAAAATCCAGCGCCACGCAGTCCATGAAAGACGAGGAAGAG
AGCCCAGGTGGGAGGCGGAYGAGGAGGAGCAG
CATTAGTCATTTTCGAAGCTCGAGCACCACGGATGCCAAGTCSGTGTATTCCTCATCGAGTTCAGGGT
TTCGAACACCTCCTCCTTATAGCCATGCACAA
ACGGCAGCCTCCAAGAGCTACAAAGATTTGAGAAGCTATTCGGATAATCACAAGCAGCAATATCAACA
CCAGCAGCAAGCGGTTTCTTTATCGAAGTACA
ATACGAACTTCGATGASAAAAGATCATCAAACAAAGAACTGACTTGGTTGGATCATGAGAAGTTCAAA
TTATCGGAGAAGTATAAGATTAGTTCCGAGCT
AGATCACAAGGGTTTGCTCAAAAGGCTAAGTGAGGTTGTTGACGACGAGGAGGAAGATGAAGGTGCAG
AGAGTGATTCGAGCTCTGATCTTTTCGAGCTG
CAGAATTATGACCTGGGTTTTTACTCGAGCGGCTTGCCTGTGTACGAAACTACAAATGTGGACAACAT
CAAGATCAGATCAGGAACACCAATTTCCAATG
CTTCGTCCTAA

>Mdo_MDP0000276736
ATGTATGTGAGGGAGAGGTCACCGAGAGAAGAAACGTTTTCGAGGCGAAGAAGAAACCCGTCTTTCTC
TTCTTCRCTRCTCGACTCCATCTACCGCTCGA
TTGATGAATCGAGCGGTGGAGATGGAGATCAGGGTTATGTTCGAGATTCGAGTACAATGGTAAAGAAA
CAGAGCTGCTCTGCTAAAGGAGGCAAGGAGAA

GGTTAATCTACGAAGGGCTATTATGATTGAGAAATGGGTCGAGAAGCAGAATGTTCACAGCTCCATGT
TCTCGAACTCCGCTTCGAGTTCCTCGGAGTCG
AGCTCGGGAGCTGCATTTTCGTCTTCCGAAACAGATTCGAGCTGCAGATCAAGAACAAAACCGAAGGC
GGTCGAGGGGAGGTTCGTGCAGTTCGAGGAGA
AGGAAAAGAGTGAGAGTGGCGGGGGAGGTGGGTTTTCAAAGACAAAGCTCCGAGCTTTGAAAATCTAT
GGAGAACTGAAGAAAGTGAAGCAACCCATTTC
SCCMGGCGGGCGTATTGCGAGCTTCATCAATTCAATTTTCAATTCCGGGAACGTCAAAAAGGCAAAAA
TGTGTCATGTTGGAGCTGTGGAAGATGTGAGC
ATCACAGAGCATGTGTCGAATTCGAAATCATCTTGTTCTTCTTCTTCTGCGCCGGCTTCTACTTTCTC
AAGGTCGTGCTTGAGCAAACCGTCTTCAAGAG
CGAAAAAATCGAGCAATGGCACGAAAAGGACGGTTAGATTTTACCCGACGAGTATGGTTCTTGGTGAG
GATTCTCAGCTTTCAASCCACCACAAATGTGT
GTTTGAAGAAGACCCAAGCTTGATGYCAAAACCCTCTTTTCAAAAATATGCAAGGTCTTGYCCTGGTA
ATTATGATACGCTGATMCAATCRGGCGMGAGT
CGAACGCAAGACCTCGTTACATTCAATTGTACCACAAAATCAACGAGTTACAGGAAGAYTTGCGCGGT
YAGTCCAACAAGTTATAGGACGACCGGCGCGG
TTGGTCCAACTTTTGTGAGAAGTTTTTGTGACAATGCAGATGATGAAGGAAGTGATCATGATGCTGAG
AGTTGTTCAAGTTCTGATCTTTTTGAGCTGAA
TCATCCAGTTGGGGTTGGAAGGTACATGGAAGAACTTCCAGTGTATGAGACTACTAATTTCAGAACCA
ATCAAGCTATTGCCCAAGGGTTCYTGTAA

>Mdo_MDP0000277127
ATGTCCATGATTAGTACAGGGGTGCTTTCAGCAGACCCCGGAGCTGATAAGATACACAAGAAATTGTT
CCACCACAGGAACGACTCCGGCGAGCTCGATG
TTTTTGAAGCTGCAAGGTACTTCTCAGGATACAATGAAGCTCCCAGCAGCCATAAAAACAACAAGACA
TCGGCATTTTCGCAGAGGATGATGAAAGAAGA
CAGATCGTCATGGAGAGGAGGCAGAATCAGCCTTGACATGCCTATCAGACACATGCTCCATCATCCTC
AGCAAAACCCTAATCACCATAACCAAGTGGCG
ATGGAAAAGCAAAGCAACATCAAAGACAAGAAGTACAAGCAACCAAGCTCTCCAGGCGGGAGGCTGGC
CAGCTTCCTGAATTCTCTATTCAACCAATCGG
CGTCCAAGAAGAAGAAGTCGAAATCCAGCGCCACACAGTCCATGAAAGATGAGGAGGAGAGCCCGGGT
GGGAGAAGGAAGAGGAGGAGCAGCATTAGTCA
TTTTCGAAGCTCTAGCACCACGGATGCCAAGTCCGTGTATTCCTCATCCAGTTCAGGATTTCGAACAC
CTCCACCTTATAGCCATGCTCAAATGGTAGCC
TCCAATAGCTACAAAGATTTGAGAAGCTATTCGGATAATCACGAGCAGCAATATCAACAGCAGCAGCA
GCAGCAAACGGTTTCCTTATCAAAGTACAATA
CGAACTTCGATGAGAAAAGATCATCAAACAAAGAACTGACTTGGTTGGATCATGAGAAGTTCAAATTA
TCGGAGAAGTATAAGATTAGTTCCGACCAGGA
CCACAAGGGTTTGCTCAGGAGGCTCAGTGAGGTTGTTGATGACGAGCGGGAAGATGAAGGTGCAGAGA
GTGATTCGAGCTCTGATCTTTTCGAGCTGCAG
AATTATGACTTGGGTTATTACTCGAGCGGCCTTCCTGTGTACGAAACTACAAATGTAGACAACATCAA
GATCAGATCAGTAACACCAATTTCCAATGCCT
CATCCTAA

>Mdo_MDP0000446864
ATGTCAAGGTGGGAAAATACGCCGAGGGAAGATAGATACAGGCACGAAAAGAAAAATCCTTCTTTCTC
ATCCTCTCTTCTCGACAAAATTTACCGTTCGA
TTGACGAGGGATCGCCGAGAAACCGCGAGGACTCGATGTTTTACAACGAAACAATGCCGAAGAAACAA
AGCAAAAGCGGTGCGAAAAGCGGCAGAGCAGT
GGAAGAAGAAGAGATGGCCAGCCTCCGCCGAGCTTGTTTGATCGAGAAATGGATGGAGAAGAAGGTTG
GCCAGAAGGTTGGTGGCCAAAGAAGACAGCAT
TTGGGTGAATTGGACCGAAAATTAGACCGTGATCTCGATGCTATGTTTTTCAGCTCCACCTCTAGCTC
CTCTGATTCGAGCTCCGGTGGGTTCTCCTCCT
CGGATGCGGATTCTATGTTCGGGTCTAAGTCGAGGTCCTCGTATTTCGCACCGCCACGGCCAAAACCG
GTTCGCACCAGCGTCTCGACCCGGTCGGTGAA

ATCAGAGGAAAAAACAGTGAGAAAACAGAGGACTCTGTTTTATGAACAGAGGGAAGTGCACATGTTCG
ATGACTACCACTACAGTTGTGCATCGGATCCG
ACTCCGAAGCTCGATGAAGGTATGATTAAGTCGAAATCGAGGGCATTGAAGATTTACAGCAACCTAAA
GAAAGTGAAACAGCCGCTTTCGCCAGGCGGTC
GCGTTGCGAATTTCCTCAATTCGATCTTCACTGCAGGGCAGACCAAGAAAACAAAGAGCACTTCATCA
ATTGGAGGGTATGAGGAAGCGGTTGCGGAGAG
GAAACTGAAATCAGGGCAAGACTCGACATGTTCTTCGGCCTCTTCGTTCTCGAGATCATGTTTGAGTA
AGAACTCACCATCTACAAGAGAAAAACTGAGA
AATGGGGTTAAAAGATCGGTTCATTTCTGCCCGGTGAGTGTGATCGTTGACGAAGATTGCAGGCCTTG
TGGACACAAATGCTTGTACGAAGGGGAGGATC
AAACCCTAATGCCGGTCACTGTTCCGACCGCATGGAAAATTGGTCGATCGCCAGCGAGAAAAGCGGAG
GAGGAGCTTAAGCTTCGAGTGTTGGAGAAAAG
CAGAAGAGTTGAGGAAGCGGCCAGAGAGATTTTGAGAGACTGCCGTCGGAACCAAGTAAAGAATGAAT
TAGTGAGCAGAGATTGTCGTGACGGTGATGAA
GGTGATGACGATGATGCTGCTAGTTGTTCGAGTTCGGATCTTTTCGAGCTTGATCACCTTTCGGTGAT
CGGGAAAGAAAGGTACCTTGAAGAGCTTCCGG
TGTACGAAACCACACACGTTCGTACAAATCGTGCCATTGCTAATGGCTTAATAATGTAG

>Mdo_MDP0000493758
ATGGAGAGGTGGGATATCAAATCACTCCCGAAAAACAGAGAAGGCCGCTCCAACCGGCACAGCAGAGA
CAACCCATCTTTCTCTTCCACCCTCCTCGACT
CCATTTACCGTTCCATCGACGAACCCACTGCTGGCCGTGATGAAGTAGTTTCGATGAGAGAAGAACAG
CGCCACCTCATTTTCTACAAAGGAACCATGAA
GAAAAAACAGAGCACCACCGCCGCCACCACTACTAGCGGCCGACTAGGCCATGGACACTATAAAGAAG
ACCAGGAGCGTGAAATCATGAGCCTCCGACGA
GCCTGCTTGGTCGAGAAATGGATGGAGAAGAAGGCGGCTGAGAACTCCGTACCTGCGCGACGAAATTC
CATGGCGGATTTCGAGACGACAAAGTCGAGAT
ACAGTCACCAGAACGAGTTCCTGACGAATTCTAGTTCCAGTTCTTCAGATTCCAGTGGTGGGTTTTCT
TCGTCGGAGTCTGACTCCATGTACGGATCAAA
ATCGAGGTCCTCATCGTCATGCTACAGCATGCACAGGCCGAAGCCAATTCGAACCAGCCTTTCGTCGG
AAAAGGTTCCATTTGACGATCACCAGAGAAAT
TACCATCACAATTCACAGAAGAAGCACGAGAATGGGTTCGGAAAGACGAAATCAAAAGCGCTGAAGAT
TTACGGCGATTTGAAGAAGGTGAAGCAGCCGA
TTTCGCCTGGTGGTCGGCTGGCCAGTTTTCTCAATTCTCTGTTCAATGCAGGGCACGTGAAGAAGTCC
AAAATTGATGATTTGGGTGCGGAGAGAAAATC
GTTACCAAATTTGGGACAAATGGGGTACTCAAACCCGAGTAGTACTTGTTCGTCAGCCTCTTCGTTTT
CAAGGTCTTGCTTGCGAAAAGCGCCTCTTTCG
AGAAGCGAGTTGAGTGGCATCAGCAGCAGCAATGGTGATGCCGCGAAGAGGTCGGTGAGGTTTTGTCC
TGTAAGTGTGATTGTGGATGAGGATTGCCGCC
CGTGCGGACAAAAGACTCTACTTGAAGCACAGTCGGGTTTAATAGCCTCGGCTAAAGCTGCTGCGGCC
ATTAAACCTCCAGCAAATGAAGATGTTGGACT
TGAGTGTTGTGTAATGGATGTTGATGAAGACGATGACCATGGCCGAAGAATCGAGGAAGTTGCGAGAG
ATTACTTGATCAAGAATTATCAGAAGAAGCAT
GATGAAGATGATGAGGATGAGGATGATGATGATGATGACGCAGAGAGCTATGCAAGTTCTGATCTCTT
TGAGTTGGATACTGCTGGGGTTGAAGAAAGGT
ACCGGTGCCTGGTGAGATGGCTGAGGGTTGCTCCGAGGCTCGGGAATCTGAACGTCAGAAGCTGCTAT
TGCTTGGGCTATCTTAGCAGTGTACTAACGCA
CAACTTCTCGGTCACGCGCAGCAGCTTCTCCGTCGCGAGCAACTTCTTCTCTTTGCCGGCACAACTTC
TTCCTTTTGGTGTGCTAGCTCCATCTCCAACT
GCTGCAGTCTGGAAGACGTCCCAATTGGAGGATGAGAGCAAAATGAAGAAAAAGACCCCGCTCGGTCC
CGAATCAGACTCGCTTCTAGGCTGTTGA

>Mdo_MDP0000873045
ATGTCCATGATTAGTACAGGGGTGCTTTCAGCAGACCCCGGAGCTGATAAGATACACAAGAAATTGTT
CCACCACAGGAACGACTCCGGCGAGCTCGATG

TTTTTGAAGCTGCAAGGTACTTCTCAGGATACAATGAAGCTCCCAGCAGCCATAAAAACAACAAGACA
TCGGCATTTTCGCAGAGGATGATGAAAGAAGA
CAGATCGTCATGGAGAGGAGGCAGAATCAGCCTTGACATGCCTATCAGACACATGCTCCATCATCCTC
AGCAAAACCCTAATCACCATAACCAMGTGGCG
ATGGAAAAGCAAAGCAACATCAAAGACAAGAAGTACAAGCAACCAAGCTCTCCAGGCGGGAGGCTGGC
CAGCTTCCTGAATTCTCTATTCAACCAATCRG
CGTCCAAGAAGAAGAAGTCGAAATCCAGCGCCACACAGTCCATGAAAGATGAGGAGGAGAGCCCGGGT
GGGAGAAGGAAGAGGAGGAGCAGCATTAGTCA
TTTTCGAAGCTCTAGCACCACGGATGCCAAGTCCGTGTATTCCTCATCCAGTTCAGGATTTCGAACAC
CTCCACCTTATAGCCATGCTCAAATGGTAGCC
TCCAATAGCTACAAAGATTTGAGAAGCTATTCGGATAATCACGAGCAGCAATATCAACAGCAGCAGCA
GCAGCAAACGGTTTCCTTATCAAAGTACAATA
CGAACTTCGATGAGAAAAGATCATCAAACAAAGAACTGACTTGGTTGGATCATGAGAAGTTCAAATTA
TCGGAGAAGTATAAGATTAGTTCCGACCAGGA
CCACAAGGGTTTGCTCAGGAGGCTCAGTGAGGTTGTTGATGACGAGCGGGAAGATGAAGGTGCAGAGA
GTGATTCGAGCTCTGATCTTTTCGAGCTGCAG
AATTATGACTTGGGTTATTACTCGAGCGGCCTTCCTGTGTACGAAACTACAAATGTAGACAACATCAA
GATCAGATCAGWAACACCAATTTCCAATGCCT
CATCCTAA

>Mtr_Medtr1g015275
ATGTACAAGTTTGAGAAGAGATCAACAATCCACGACAGGTTCATAAACCCTTCATTCTCTTCCACCCT
CTTAGACCAAATTTACCGTTCCATCGACGAAG
GAGAAACAAAATTAACCACCGAAACAACCAAATTTTACAGAGAACAAAAACCAACGGTTATAAACAAA
CCAACAACCGACAGAAAATATTATAACTACGA
ACCAAAAACTGAAAAACTTGTCACTGTCGTGAAAGAAACCAACAACAATCGAAAATCAAATCAACATG
ATCATGACCAAGATGCTCTGTTCTTCAGTTCA
ACCTCAATTTCTTCGGATTCTAGCTCCAGTGGATTCTCCTCCTCCGACACAGATTCATTATACAGAAC
AAAATCACGTTCCTCGTCGTGCTTTGTTCCAC
CGAGGCCAAAACCGGTGAGAACAAGCACGTCGGCATCATTTAGGTTTGAAAAAGAGAAACATGGGAAT
CATGTTTTCGATGATTTTTGTCGAAGTTCAGA
AACCAAACAAGGCACAGAAACTCGCGGCGAGGAAGTAATTTTAATAAAGAACAAATCAAGAGCCGTTA
AGATTTACAATAACTTGAAGAAAGTGAAGCAA
CCGATTTCGCCAGGTGGAAAACTCACGAGTTTCCTCAACTCTCTATTTGTTAATAGTAACAATGAGAA
GAAAATGAAGACTGAGAAACCAACAAAGACGA
AGAAGCAGATGAACACGTGGGAGACTCATGAGGGAAAAGTAGCTTCCACGTGTTCATCTGCTTCTTCG
TTTTCGCGGTCTTGTTTGAGCAAAACAGCGTC
ATTTTGTGGTAGAAATGATTACAAAACTGTTGGTTTTTGTGGAGTGGAAGAGGGTAGAGCTAAGGTGG
AGGAAGCTACTAGAAAGTTTTTGAATGAGTAC
CATAGCCGTAACAAGAAGAAGAAGAAGGACGATTTAGTTTTATTAAAGGATTTGTGTATTAATCAAAA
TGAAGAAGAGGATGAAGATGATGATGATGTGG
CAAGTTGTGCAAGTTCTGATCTTTTTGAGTTGGATCATTTGAGTGTGTTTGGAGATAGTAGGTATTGT
GAGGAGCTTCCTGTGTATGGAACTACCCGTGT
TAGTTAA

>Mtr_Medtr3g104880
ATGTATAAGTTTGAGAATACTCATATAGAAAAACGTTTTCACAACAACTTTGAAAATCATTCCTTCTC
TTCCACACTGCTCGACCAAATTTACCGTTCCA
TCGACGAAGGAGACAGAAAAGTTTCTGATATGAAGTTCTATACAGAAACAACATTTCAAAAACAGAGC
AAAACCAATGCTAAATTCAACAGAGTTTTTGA
AGAACAGCAACCATATCTTCGCGGTGTTTGTAAAGAAAAAGAAAAAATCACAACACAAATAGACAGAA
AGTTGCATCTTGATCATGAGATTCATGACCAA
GATGTTATGTTTTTCAGCTCAACTTCAAGTTCTTCAGATTCAAGTGGTTTATTATCATCCTCCTCCGA
AACAGAATCAATGTACAAAGCCAAATCACGAG
GTGGTTCGTGTTTCGCTCCTTCAAGGCCTAAGCCGGTGAAAACAACCGTTCCTCCAGAGAGAAGAATC
ATTGCAAACGATGAAGATACCTTAATCAAATC

AAAATCAAGAGCGTTGAAGATTTACAACAATCTCAAAAAAGTGAAGCAACCCATTTCACCAGGTGGAA
AACTCACAAGTTTTCTCAACTCTCTCTTCATA
AACACAAAGAAAACAAAAACTGTTTCGTCTTATGAAGACTCAAATGCAGAGAGGAAAGGTAAACCAGG
ACAAGCTTCAACAACTTGTTCTTCGGCTTCTT
CGTATTCACGTTCTTGTTTGAGTAAAAATTCATCTAAATCAAGAGATAAATTACACAATGGTGATAAA
AGAACGGTCCGTTTTTACCCGGTGAGTGTGAT
TGTTGATGAAGATAATCGAGCTTGTGGTCATAAATATTTGAATAAAGGAGTTACAAAGAAGAATGAAG
AAGTTGTGGACAAGAGTAAGAAAGAGGAGGAA
GTTGCTAGAGAATTTTTAAGAGAGTACCATCTTAACCATAAGATTTTGAGGGATTTTTCTATGAAGAA
AAATGAAGAAGTTGATGATGATGTATCAAGTT
GTTCAAGTTCAGATCTTTTTGAGCTTGATCACTTGGATGTGATGGGAAATGATAGGTATTGTGAAGAT
TTACCTGTGTTTGAGACTACTCATGTTAGTAC
TAATCGTGCTATTCGCATAATGTAA

>Mtr_Medtr5g046750
ATGTCCCTAGCAGGACTTATTATAGACCAAGAAATGAACCACAATAAGAAATCCTTCCACAGAAGAAA
TGATTCTGGTGAGCTTGATGTGTTCGAAGCAT
CAAGATACTTCTCAGGATACAATGAAGTTATTGGATACAATAATAGCTCAACTTTCACACAAAAGATT
ATGAGAGAAGAAAGGAATGGATACAAAGGTAG
AATAAGCTTAGACATGCCAATGAGAAGCTTACTTCCACAACAATTTCATGGTGGAATTGATCAGAAAC
AAATGAAAGAGAAGAAACACAACAAACAACCT
AGCTCACCAGGTGGTAGACTTGCAAGTTTCTTGAATTCTCTCTTCAATCAATCAACATCGAAGAAGAA
ATCAAAATCAAGTTCACAATCCATGAAAGATG
AAGATGAGAGTCCTGGTGGTAGGAGAAGGAGGAGAAGCAGCATTAGTCATTTCAGAAGCTCTAGCACT
GCAGATTCAAGGTCTATTTACTCTTTGAATTC
GGGTTTTCGAACTCCTCCTTATGTAAACACTCCAACAAAGGGATGCAGGGAATTCAAAACCTTATTAT
CAGATCAAAAGAATGAAGTGTCTTTGAAAAAA
TCATCAACCACTTTGCAAAATGAGTTGTGTTGGGATGATAAGAAAAAGAGAGACTCAAATTTGAAACA
GTTTGTGGAGAAAAAGTACTATGAAGAAGAGA
AAAGGGAAGTGAGAAAATTCAATGAGGTTGATGATGGTGCAGAAAGTGATTCAAGTTCTGATTTGTTT
GAATTGCAAAACTATGAATTGAGTCATTATTC
AAGTGGTTTACCTGTATATGAAACTACCAACATGGATAATATCAAGAGAGGATCAACAATTTCCAATG
TCCCTTTGTGA

>Mtr_Medtr8g019660
ATGGAGAAATGGAATAAACCTTCAAGAGATCATAGAAAACAACATCAAAGAGAAAACCCATCATTCTC
TTCATCTCTTCTTGATGTAATTTATCGATCGA
TCGACGAAGGACATCATAAAACAGAGGAAAAAGAAGAGAAACTCATGTTCTGTAGAGAAACAACAACA
ACCATGAGGAAGATGAAAAGTGTTGAAGCTGA
AAAACCAAACTTTAGAAAAGCAAGGAAGGTTGAGAATTGGAATGGAAGAAACTCCTTAACAGAATTTG
AAACAAGAACAACAAGAAGCAATTCAAACACA
CTCTCCATGCATTCAAGCTCATCTTCTTCAGAATCAAGTTCAGCTGGTGGTTTTTCATCATCTGATTC
AGAATCATTCTATGGTTTACAAAAACCAAAAC
CAATTAGAACAAGTGTTTCTGAAAAACCAAACATTGATTCATTTCAATATGGTTATAGTGCTAGAAAC
CATAACTATAACCATAACCATAACCATTCTGT
TCAAACTCAAAAACCAAAAAATGAGAATGGTTTAGGTAAAACAAAGTCCAAAGCTTTGAGAATTTTGT
ATGGTGATTTGAAAAAATCAAAGCAACCTATT
TCACCTGGTGCAAAAATTGCTAGCTTTTTGAATTCACTTTTTACTTCTAGTGGAAATACCAAAAAACC
AAAGATACCATCATCAACAGCTACAAAAACTA
CTACAAATTCTGTTTTGGAAGCAAAATCAGCACAAGCTTCAACATGTTCATCAGTGTCTTCATTTTCA
AGGTCTTGTTTGAGTAAAACTCCTTCATCAAG
ATCAGGTGCTAAAAGATCAGTGAGGTTTTGTCCTGTTAGTGTTATTGTTGATGAAGATTGTAGGCCAT
GTGGACACAAGAATTTGCATGAAGGTGAAAAG
GGTTCTTCAATCTATGATGGAAAGAATAATAGTAGTGAAGAGTTAAGGTTGCATGTTTTGCAAGAGAG
TTTAAGGGTTGAAGAGTTAGCAAGAGACTTGT

TGAAGAATTATCAGAAAAAAAATGAAGTTGATTTTAATATGCAATTTGAAGATGAAGATGAAGATGAT
GATGATGGAGCTAGTTGTTCTAGTTCTGATTT
GTTTGAATTGGATAATTTATCAGTAATTGGGATTGAAAGGTATAGAGAAGAATTACCTGTGTATGAAA
CTACTCATTTCAATCCCAATAGGGTTATTGGA
AATGGTTTCATTATGTAA

>Mgu_Migut.A00091
ATGTATACCAGAGAGGAGAATTATCATTACAAAACAAGAAAAAACAGCATGAACACACCTTCATTTTC
CTCCACCCTTCTCGACGAAATCTACCGCTCAA
TCGACGGCGGCGCCGCCGCGGCTGAAAAACCAGAGGGTTTCAACGAAAAGCCTCTCAGAAAACAGGGC
GGCGGTGGTTGTGCTTCTGTCAGAAGGGTTTG
TCTGGTCGAGAAATGGATGGAGAAGGAGGCTAACGAGAACGTCGTTGCTAAAAAAACGGCGGCGGCGG
CATTGTTGCCGGAGCTGGACAACAACGATCTG
CTGTTCTTCAGCTCGACTTCGAGTTCTTCCGACAGCTCCGGCGCACTTTCTTCATCGTCGGAAACAGA
GTTCTTCGGCTCCTCCTCCACGAAGAAGCCGC
CCAAGGTTTCTTGTTTCTCATCAACAAGACCTAAACCGGTTCGGACCGGCGCCACGACCCGAGTTGAA
ACAAAACCGCAAAAGGACAACCGGTTTTTGTT
CGACGATTACAACAACAACAGCAACAAGAAGAACCAGGATCATCAGAAAAGCAAAACGGGAGATGATT
TGCTGATCAAGTCAAAATCCAGGGCTTTAAAA
ATCTACGCCAATTTGAAGAAAGTGAAGCAGCCAATTTCTCCAGGTGGGAAGCTCACAAGTTTCATCAA
CTCCTTCTTCACCAATGGCAATAACGTAATTA
ATACAAAGATTCAGGATTCTTCGAAGTCTTCACCAAAGGCCTCGTCAACTACTTGTTCGTCGGCTTCT
TCGTTTTCCAGGTCATGTTTGAGCAAATACTC
GCCGAAATCAAGGGAGAAAATCAAGAACGGCGTTCAAAGAAGTGTGAGATTTAATCCAGTGAGCGTTG
TCGTTGATCAAGATTTAAGGCCGTGCGGGCAA
AAACCCGTTTACGAAAGGCCTCCGTTGCCACCAAATTCATTGGCAAAGTTGAAATTTAGCAAAATGGA
GAAAAATATCGAAGTTGAAGATGCTGCAAAGA
ATGTTCTAAGAGGGTATTGTAGTAAAAACGGCGACAATTTGTCCTTGTTTAGGAAAATTTGCGACGAA
CAATATGACGACGAAGACGATGATGATGCAAT
GAGTGATTCGAGTTCGGATTTATTCGAGCTCGACCACTTAGCATTCTTTGGAGACAAAAGATTCTGCG
AAGAATTACCCGTGTACGAAACTACTCGTTTA
GACACTAATCGCGGTGTTGTTAGCCGTTTGGTTCGTTAA

>Mgu_Migut.E00039
ATGCTTTCATCAAACCCTAACAAGAACTCACTCCAATGGAGGAAAAACTCCGGGGAGCTCGACGTTTT
CGAGGCGGCAAAGTACTTCTCCGCGGCCAACG
AAAACGAGCAAAATTCAGACAACGTCCTTAGCGGAACGAATCTTTCTCAGGCAATCATCGTCAGAGAA
GACAGACAGAATATTCTCCGACGATCCCGATT
GAGCCTGGATTTGATGCCGAACGACAATCCAATCTCTCATCAAAAGATGATCGTGAAAGAGAGACATC
AGCCAACTTCTCCAGGTGGCAAGCTGGCTAGT
TTCTTGAATTCCCTCTTCAACCAACGACACTATTCGAAGACGACGAAGAAAAAGAACAACAAGTCGAA
ACCCGAAAAAGATTTCGATGACGAAAATACCC
CTGGTGGAGGGAGAAGGAAAAGAAGAAGCAGCATTAGCCATTTCATAAGAATCACAAGTAGCAACAAC
AATAATAATCACACAACTTATCCGAATACTCC
GGCAAAACAGGTTATTATCTCCTTGCCTAAAAACACCGGGAACTCGAACCTGAATCCCGCGTCGATGG
ATTACGAGTGCGTCAAAGAGAAATCCAGGTTC
AGTAACGGCGTTTTCTCCGACAAAATATCGAGAAACGGGAGTTTCGGATTTTCGAGTAATAAAGATGA
TTATGAAGGTGCAGAGAGTGATTCCAGCTCGG
ATTTATTCGATTTACCGAATCACGAACTGGATTTCGGCTCAAGTGATCTGCCTGTTTACGAAACAACA
CGTGTGAACATAATCAGGATTGCTAAACCGAT
TTCCAGGGCTACTACTACTACTACTGCAGTTTAG

>Mgu_Migut.E01574
GCAAAACCATCCAGAGAGGAGAATCACAAACCTAAAAAATGCCACCGGAATACGCCGTCGTTTTCCTC
CACACTCCTCGACGAAATCTACCGCTCAATCG

ACGGCGGCGGAAATGCTGAAAAAGCCGGCGCAGAAATAAATCCTGGCTCCCACAGAGAAAAACCAGCC
ACTGGTTTAAGGTCCAACAATGGCGGCAGAAG
AAGCAGCACCGGAAATCACCACGAACAAACAGCAGCAGCGAGATTTTCAAGGGGTTGTTTGGCTCGAA
ACAACGACAAAGATCTCTCATTTGACTCCACA
GCAAAATCCGAGGTTTCCAATCGTCCGTACAGCACAAACCCTAATTCCGATTTCACCCGGGAAGAGGC
TAACGAATTCATCAACTCACTCTTCACCAATT
TGAATTCTAATAATAAAAAAACCAAAAAACAAGATCCGAAGCAGCAGTCTTCAAAGGTGACGTCATCG
TCTACGCCGTCGTCGTCGACTACTTCTCCTTC
GTCGCGGTTGAGCAAGCATTCATCATCAATTATGAGAAATAATGAAATTGGGATTAATCAACCGACGG
TCAGATTTCATCCCGTTGGTCGTCCCGTTTCG
GAGGATCAAGATTCGCCGTGTCGGCCCAAGAGTATCGTATACGATAATGATCGAAAATTATACGGAAG
GCCACCTTTGCCGCCAAATGCATTGGCTAGAA
ATCGGAATATTAATCAAAGGAGGAGTGATTTTTCCATGTTCGAAGAAATTGACGACGAAGATGGAGTT
AGTGATTCGAGTTCCGATTTATTCGAGCTGGA
TCATTTGGTTTTGTTTGGGAATACTAATATTTTTTGTCAAGAACTTCCGGTTTATGAAACTACTTATT
TTGACAATAATCGTGCTGTTGCTGCTGGTTTG
TTTTGTAATTAG

>Mgu_Migut.I01063
ATGGTGGATGGGTATAGTACTAATTACAGAAGGCAGAGTGATATTAATACCTCACTATACAATCCTTC
GTTTTCTTCCACTTTGTTGGATACGATTTACA
GATCTATTGATCAGGGCGGAGAAGAGGAGAAATTAGGTATGAGGACGAAGCAGAGTATTGCTTACGGT
TCCGACGGCGGCGCCGGTGTGTTTCGGAGTGA
GGAGGAAATGGCTAATTTCCAGCGCGCCTGTATGATTGAGAAATGGATGGAGAAGAAGGCCGGTGAGA
AGGTCGCCGTACGGGGAAATTCGGCGGCGGAT
TTGGGGATGAAGAAGCCGCGGAAGGATCGGGAGAATTCGACTTCTAGCTCCTCCGATTCCAGCTGCGG
CGGCGGCGATTTTTTCTCCTCGTCGGAGGCTG
AATCGTTTCCGGTGCAGAGGCCGAAGCCGATTCGGACGGGGTTGGAAAAGGAGAAACTCGAGAAAAAC
CACCGCGATTCCGAATCGGAGTTCCACCGCCG
CGAGAGGCGGGGTTTCGCCGGCGACGTGGAGCAGAAACCTAAGCACGAAGGCGGATTTCTCAAGACGA
AATCGCGAGCGCTGAAAATCTACGGCGACTTG
AAGAAAGTGAAGCAGCCGATTTCTCCCGGCGGCAAGCTCGCCGGATTTCTGAACTCTATCTTCGCCGG
CGGAAACCTGAAAAAACCTAAAACCGCCGCCA
ACGGCGACTACTCCCCGTCGCTAAAATCGGCAAATGCATCCACATGCTCGTCGGCTTCATCATTCTCC
AGATCCTGCCTCAGCAAAACGCCGTCGTCGTC
CGGAGGTAGTAAACCTTCCAACGGCGCGAAACGATCCGTACGATTTTCTCCTGTGAGCGTGATCGTAG
ACGACAAAGTAAACAACAACAACAACTACACC
AAAAAACAAACTTCAAAATTCACCGCCACCGCCGCCAACAACAACAACAACAAAAACAACATTAACAG
TCTCCGAAACGTCATCAACGAAGAGCTCATGG
TTCATGTAATGGACAAAAACCGGCGAGTGGAGGATGCCGCCAGAGATCTGCTACGGAATTACCATCGG
AAAAACCAAGTCGGATGTGAATTCGATTCGTC
ATTCGATCATTTAAGAAAACCGCAAGTAATTAAAAATAATCAAATCATTAATCAAAATAATTTCGACG
AGGAAGACGATGACGCGGCAAGCTGTGCGAGT
TCGGATTTGTTCGAATTGGATAATCTTTCAGCCATTGGAATGGAGAGGTATAGACAAGAATTACCAGT
TTATGAGACTACTCATTTGTAA

>Mgu_Migut.J00952
ATGGCTGTGGGAGAGAGACAAACACAGAAGCACAGGCGCAAAACGCCGTCGTTCTCCTCTTCCCTCCT
CGACGCCATTTACCTCTCCATCGACGAACCCG
GATGCGCCGCCGCCGCCGCCTCGCAACCCCACCAACAATCTGAAGACTTCGTCCACCTCCGCAGCAAC
ACCAGAAGAAACAACGCCGCCCATTTCGAAGA
CGAAATCGCAAGCCTCCGCCGAGCAATCACGGTCGAGAAATGGATGGAAAACCACACCACCACCACCA
CCGTCGTCGCCGCCGCCGCCGCCGCCGCCGTT
CCAAGGCGGCGCATCTCCTCAAACTCAGGCACATCATCAGATTCAAGCATCCTCTCCTCATCTTCAGA
AACAGATTCCTCTGTTCTCTCGAGATCTTCAT

CATCGTCAAAGAATCATCTCTACACAAGAACAAAATCCAAAGCAATGAAGATCTACGGAGAATTGAAG
AAAGTGAAAGAACCCATTTCACCCGGTGGCAA
AATCGTAAATTTCTTCAACTCCATCTTCAGCCCCAGAAACCCTAAGCAGAAACAGACCACAGTGGAGG
AATGGAGCTCCTCAATCAGAAAATCAAGATCA
ATGAGGGACCCACCAACTACATCCTCAAAATCTTGCTTAATCAAAAACCCGTCTTCTTCGATATGCAA
TAAATCGAAGAGGTCCGTTAAATTCGATGAAA
ACGAGTACTCGGTTAATTCATCAATGCCGAGTGTTAAAAGCCGGCTTATCAAGAAAAGTGTCGAGCTT
TTTGAGAATGAGAGTGACGGCGACGACATGAG
CTGCGCAAGCTCCGATCTTTTCGAGCTCGAGAATATCGGATCGTACGGCGGCGAAGAATTGCCCGTTT
ATGGAACGACGAGTATTAAGATGAATCGAGCC
ATTGCTTGTGGATTCGCAATGTAG

>Mgu_Migut.L01171.1
ATGTCTGCTGTGATCACCCAGTGGAGGAACAACAACTCCGGCGAGATCGATGTTTTTGAGGCGGCCCG
GTATTTCTCCGGCGGCGCCGATCAGAACCCAA
TTAACACAGCCGTTCCTCCTCCTCCTCCTCAAAGAGCACTTGCCTGCAGGATAAGCCTGGAAATGCCA
AAGAATCCGTACAGTAATCTGTCCGCCGCCGC
CGCCGCCGCCATGGACCACGGTGGAGATCAGAAAAAGCAAGAGAAGAAGAAATACAAACAGCCGAGCT
CTCCCGGTGGGAAACTCGCCAGTTTCTTGAAC
TCCCTCTTCAACCAAACAACCTCGAAAAACACAACCACCAAGAAGCTGATGAAGAAGAAGAACACGTC
GGTTAAATCTGGCAGTAGCAATAACGATCTGG
AAGAAGAGAGAAAACGAAGAAGCAGCATCAGCCATTTCAAAATCACAACTACTAGTGATTCTTCGAAT
AAATCCACGTATGCAAATATGATGGCAACTCC
GACGAAATCATCGCTCAGAAGTCTGTCTCACCACCAGATCGAGCTGGGGGTTTCGGCCCCCTCCAGAA
TAATCAACGCTGGCGTTGATCAGAAAAGAACT
ATTAGTGACAACAATATTATTAGTTACAGGAATAATAATGCGGGGTTTGGATTTTCAGACAGGAGTTC
GACCGTCATGAATAGGAAATTCAGCGATGACG
ACGACGACGACGAAGATGGCGCAGAGAGCGATTCGAGTTCGGATTTGTTCGATTTGCCGAACCATGAT
TTGGATTTTTGCTCGGCATCAAGTGGTAATTT
GCCTGTATACGGAACAACCCACATGGATAGAATCAGGATTTCTGCACCTATTTCCGCCGTTGCAACAT
TTCTATAA

>Mgu_Migut.L01544
ATGGCTGTGTGGGAAAAACAGGCTAAAAATAAAGAACCCTCGTTTTCTTCATCGCTCCTCGACTCCAT
TTACCGCTCCATAGACGAAAACGGAGCCCCGA
ACGAGCAAAAAATGGAAGACAATTTTCTTCTCCACAGAAGAAACAATGCTGCCGCAAAAGTCGAAGAA
GAAATCGAAAGTCTGCGAAAAGCGATCATGAT
CGAGAAGTGGATGGAAAACTACAAGCCCATTAATACTACTACTACTACTACTACGATGCACTTCCCTT
CAAACTCGGGCTCGTCGACGGATTCGAGCATT
TTTTCTTCTTCGGAAACAGAGTCCTCTGTTTCAAGATCGTACAAGATTCCGCAGCTCGAGAATAAACC
CGCGACGAAAAAAATCGAGGGCGCAGAAGATTT
ACGGAGACTTGAAGAAGGTGAGAGAGCCCGTTTCACCGGGTGGCAGGATCGCAAGTTTCTTGAACTCG
ATATTCAGTCCGAGAAAGACTAATGAATTGTG
CTCAATGAGAAAGTCGAAGTCAATGAAAGACACAACTACTACTACTACTACTGGAACAACGACGACTG
CGTCAACGAAGACATGCTCGTTGGAATCGAGA
TCTTGCTTGAGCAAAAACCGTTCGTCGGGAGATTGTAAGTCAAAGAGGTCCGTTAGATTCTGCATTGT
CGATGGAGATTGCCAGCCGTGTGGGAACAAGA
GCGGATTATTTTACGACGAAAAGGAAGATTTGGTTAATACAACGGTGGCTAATATCGGGAGCTACTTC
ATCAAGAAAAACACGAAGAATGCGAGGAAGAA
TCAAGAAGTTAGAGATTTGGATGAATTGAGCTGCGGAAGCTCGGATCTTTTCGAGCTTGAGAATATCG
GTAGGTATGAAGAAGAACTGCCTGTGTATGAA
ACCACAAATCTTGAATTATGCTAA

>Fve_mrna04564.1-v1
ATGTCATCTATTGCAGGAGTTGCAGACCCAGCTGAGAAAGCACACAAGAAATCCTTCCACCGGAGGAA
CGACTCAGGCGAGCTGGACGTGTTCGAAGCAG

CACGGTACTTCTCCGGCTACAACGAAGCTGCTGCTCCGACGATGATGAAAGAAGATAGATCGTCATGG
AGAGGAGGCAGAATTAGTCTTGATATGCCAAT
CCGAAACGTGTTGGATCATCAGCTTCTTCATCATCATCATCACCATGCGGTGGATCAGAAACAGAATA
TGAAAGAAAAGATCAAGTACAAGCAGCCGAGC
TCTCCAGGTGGGAGACTAGCTAGCTTCTTGAATTCGCTTTTCAACCAATCTAGCTCCAAGAAGAAGAA
ACCAAAATCCTCGCAGTCTATGAAAGATGAGG
AGGAGAGTCCAGGTGGAAGGAGAAGAAGAAGAAGCAGCATTAGCCATTTCCGAAGCGCGAGCAGTACT
ACTACTACTACTACTGATGCCAAGTCCATATA
CTCATCTTCAAGCTCTGGCTTCAGAACACCTCCTCCTTACAGTGCACAAATCGCAACCGCCAAGAGCC
ACAAAGATTGGAAAATAAGTCACTCAGATCAC
AAGCCAGTGGTGTCCAATTTGTCCAAACATGATGTTATGTCACATGCAAAATCTTCAGTCTCGGATGA
GAAAGTTTCATCAAACAGAGAGTTAAACTGGT
TGGACGAGAAGTTAAAATACAATAACCGGCTGTCGTTGTCAGAAAAATACAAGAGTTCAACAGATCAG
GATCACAACAAAGGATTGTTGAAGAGGCTGAG
TGAGGTGGTTGATGTTGATGATGACGATGGTGCAGAAAGTGATTCGAGCTCCGATCTTTTCGAGTTGC
AGAACTATGACTTGGGAGGATACTACTCGAGC
GGTTTGCCTGTGTATGAAACTACCAACGTGGATAGCATCAAGAATATCAGAGGAACCCCGATTGCAAA
TGCCTCATAG

>Fve_gene07538-v1
ATGGAGAGGTGGGATCACAAATCACAACGAGAAGCTCGACACATTAGTAAGCACCGTAGAGAAAACCC
TTCTTTCTCTTCGTCTCTGCTTGACTCTATTT
ACCGCTCCATCGACGAAGGTGAAGAGAATATTGGAACTCGGAGAGAAGGAGAGCTCATATTTTATAAG
GAAACCATGACGAGGAAGAAACAGAACAGTAC
TACTATTGTTTCTTCTCACGGCGTCAAAGCAGAGGAGGAGATCATGAGTCTCCGGCGAGCCTGCTTGA
TAGAGAAGTGGATAGAGAAGAAAGCTGCGGCG
GCTGACAAAGTCGTGGTTCGAAGAAATTCCATGGCGGATTTCGACAGAAGGTTCTTGAATTCGAGCTC
CAGCTCTTCGGATTCGAGTTGTGGAGGTGGGT
TTTCTTCATCGGAGTCAGACTCCATGTACGGGTCGAGATCGAGGTCGTCGTCTTCATGCTACAGCATG
CCGAGGCCCAAACCCATCCGGACCAGTGTCTC
ATCAACTCGACCGGAAAACCATCATCAGAAGCCGAAGCAGGAAAACGGATTCGTGAAGACGAAATCCA
AGGCGTTGAAGATTTACGGCGACTTGAAGAAG
GTGAAGCAGCCAATCTCACCGGGAGGAAAGCTCGCCAGTTTTCTCAATTCCATTTTCAACGGCGGGAA
TGGAAAGAAGCACAAGATTAACATCGACGACT
TGGGCTCCAAATCGACAAACGGGTCCAACTGTTCTTCGGCTTCTTCGTTCTCGAGGTCCTGTTTACGC
AAAACCTCTGTTTCAAGAGGCGAGCTCAGCAA
TGGCGGCGGCGACAGTGCGAAAAGGTCGGTGAGGTTTTATCCGGTGAGCGTGATCGTCGACGAGGATT
GCCGGCCGTGCGGGCACAAAACTCTTGAAGAG
ATAAAGAGGAGTTTTGTAATGGATGAAGATGATCATCGTCGCCAAGTTGAGCAAGTAGCTAGAAGCTA
TTTGATGAAGAATTACCAGAAGAAGACTCACG
ATGTGGAAATGGAGGAGGATGAGGATGATGATGATGATGCAGCGAGCTATGCGAGCTCTGATCTGTTC
GAGTTGGACACTGTTGGAGTTGAAGAAAGGTA
CCGTGAAGAATTACCGGTGTATGAAACTACGTATTTCGACAGAAATCGAGCCATTGCTAATGGTTTGA
TTTTTGTGAGGATTTGGCTGCAAGTCGGTCTC
ACTTTAGTTAAAGTATTTTCTCTGGCCACCGCACGTGACGGGGCCGACCACCGTGCCCCAGCAGCTGT
CGCGCACTCACACGTGTGGGCGTCGTCACAGA
TCCCAATAAATATCCGCTGTCGCCACCGCCATGTCACCACTGCCCCCCTACGGCCAAGCAACGACATC
ACCACCACTCGTGCTCTGACACCTGCGGCAGC
TTTGGCGGGTGAGAGTACGAAACTGACCTCGGAAATTGTAGGGAGGGGTCGTCTTTCGATGGGTAGTT
TTGGGAGGAGGGGGCGACAGGGCAGCAGCTTT
AGGAAAAGAAAAGATGATCATGGTGGTAATGGTGGTTTGTGTGCACCTGTGTGTCTGTGA

>Fve_gene12721-v1
ATGTACATGAGGGAGAGATCTTGGCTTGAAGATGAGAGGTTCGTTTCTGGAGGAGGAAGGAGGAGAAG
GACTCCCTCTTTCTCTTCTTCTCTTCTTGATT

CTATTTACCGTTCGATTGATGAATCCAACGGTGGAGGTGGGGATGATTCGAATAAGAAACAGAGCAAA
CAGAGCTGCTCTGTTTCAAAGCGTGAAGAGAT
TAAAGGAGGAGGCAGCGGGAAGACCAAGACCAATCTTCGCCGAGCCATGATGATCGAGAATTGGGTTG
AGAGGCAAAGCGTGCATAGCTCTATGTTCACG
AACTCGGCTTCGAGTTCTTCAGAGTCGAGCTCCGGAGCTGCATTTTCGTCTTCGGATCAAACAGAGTC
GAGCTATAAACGACGTTCGAAACAGAGTCCGG
TGAAGGCTATCTCTGTTGGCTCCGATAATATGGAGAAGACGAAAAGCGAGGGCGGAAGCGGAAGCGGA
GGTGGGTTTACGAAGACGAAGCTGAGAGCTTT
GAAAATCTACGGCGAGTTGAAGAAAGCCAAGCAACCCATTTCGCCGGGAGGGAAGATTGCGAGCTTCA
TAAACTCGATTTTCAATTCCGGGAACGTCAAG
AAAGCCAAGATGTGTTATGTTGGAGCTGTGGAAGACGTGAGCACTTCAGAGCATGTAACCAAGTCCAC
TTACTCTTCTTCTACTACTCCGGCTTCAACTT
TCTCAAGGTCATGTTTGAGCAAACCATCTTCAAGGTCCAAGAAATCCAGCAATGGCAACAAAAGGTCT
GTTAGATTTTACCCTATAAGTGTGATTCTTGG
TGAAGATGATTCTCAGATTTCAAGCCATAAATGTGTTTATGAACAAGATCCTAGCTTGATGCCAAAGC
CCACATTTCAAAAATATGCAAGAACTTCTGGT
CAAAATTTCCGGAGAAGCTTCTGTGAAGATGGTGATGATGATGAAAGTGATGCCGAGAGTTATTCAAG
TTCAGATCTTTTCGAGCTTAATCTACCTGTTG
GGATTGGTAGATATACAGAAGAGCTTCCAGTTTATGAAACCACTAACTTCAGAACCAATCAAGCCATT
GCCAAAGGCTTGATACTGTAA

>Fve_gene15408-v1
ATGATGCAGACGCTAGCCTACGAAGATACTGCAGCATTGCTTTGTTGGGAACGGAGCAAAGTTCCTCG
CTTTGTTCAGTCACTATCCAGTATCCACCTTG
TTCTTCTTCTCTTCCTCGGGATCCCCAACGCCCATATCTTTCAGTGCATTTCTGCAGCGAGCTTCCTA
AATTCAAAAATCAAGTGGGTTCTTTCAAGCAC
TAGTAGCATGAACTTTCGTAGCGCTGGCAAACTAGTTCCAAAGACGAGACCGTACGATGCAATCATGT
TATTGTCCAATATTGGGCAAGATCAAGATCAA
GAGAACAAGCATGGAATGCTCCACTGCGCCAGACAAAAGGTAAATCTAAAGCCCGAGGCAATCTGGGA
TTTCACTCATTTCACCAAAAGAGACACCAAAG
CTTTTGAACCTCTCACTCTTGTTCCTTGTTTCATTTTGTCACTCTCATCGATAACAATGTCAAAGTGG
GATAGCCCTTCTTTCTCTTCTAGTCTTTTGGA
TAAAATTTACCACTCAATTGACGACGGCGAGAGGCGAGGTGAGGACGTGAAGTTTTACGGCGAGACGA
AGACGACGGCATTGCCGAAGAGAAACAGCAAA
AGCGGGAGAAATATTGCTGTGGAGGAGGAGATGGCGGCGATTCGCAGAGCTTGTTTGATTGAGAAATG
GATTGAGAAGAAGGCAAAGGAGAAGGCCGAGG
CAGAACGGAGGAGCAGGAAATTGGAGAGGGATCATGATGACAGTGTGTTTTTCAGTTCAAATTCATGC
TCTTCCGAGTCGAGTTCCGGCGGATTCTCCTC
GTCGGACGCTGAGTCCATGTATGGCTCTCGCCCGCAGAGGGCGTCTTGTTTTGTTCCTTCTCTGTGGC
CGAAGCCGATTCGGACTCGACCGGTGAAAACA
GAGGAGAAGAAAACAGAGAAGAAACAGAGTGCTCTGTCTCGTCAACAGAGAGATTTTTGCTTGCTTGA
TGATTATACTCAGAGTCGGATCACAGATCGTA
CTATGAAGTTGGAAGAAGCTACGATCAAGTCCAAATCAAGGGCATTGAAGCTCTACGACAATCTAAAG
AAGGTGAAGCAGCCAATCTCTCCTGGCGGTCG
CGTTGCGAGTTTTCTCAATTCTCTCTTCACAACAGGGCAAGTCAAGAAGAGCAAGGGCTCATCATCAG
TTTCAGGGTATGAAGAAACAGAGAGGAGAATA
TATTCACTGCAGGGCCCAACGTGTTCTTCTGCTTCATCATTTTCAAGGTCATGTCTAAGCAAAAACTC
ACCCAGCACAAGAGAAAAGTTGCGCAATGGGG
TTAAGAAATCGGTCCAATTCTACCCGGTAAGTGTTGTAGTTGGCGAGGATCGTCAACCGCATAGCCAA
AAGTGCGTGTACGAAGAACAAGACGCACATCT
TATGCCTGTAACTGTGCCGACGGCATGGAAAATCGGTCGATCTCGGTCGAGAAGAACAGAGGAAGAGA
TCGTCAAGCTTCGAGTATTGGAGAAAAGCAGG
CAAGCAGAAGAAGCAGCCCGGGAGTTTCTGAAAGACCACCGTCGCAACCAAGCTAAGAATGAGTCTTT
ATTCAGTAGAGACTATAACAACCATGATGATG
ATATGGATGATGATGATGCTAGTTGTTCGAGTTCGGATCTTTTCGAGCTCGATCACCTTGCGGTGGCC
GGAAAAGAAAGGTATCTGGAAGAGCTTCCGGT

GTACGAAACCACTCGTGTCAAATGTACGAATCGTGCTATTTTCTAG
>Bdi_Bradi1g72920
ATGGAGAGGCGGGGTGAGAAGGGGGCAGCGGCGGCGAGGGCGAGGCGGCCGCAGGGCGCCGCCGAGCA
GCCGTCCTTCTCGTCGACGCTGCTGGATGCGATATACAAGTCCATGGACGAGCCGGGACATGACGCTG
TCAGCAAGAAGAAGCAGCAGGAGAAGGAGGAAGCCCTGCACTACAGCTACTACTACAGGCCGTCGCTG
GCGGGGAGCTACCGTGCGCGCGCGCCGGGGCCGGCGCACGCCACGACGACGACGTCGAGCTCGTCCGA
CTGCTCCAGCTACGGCGGCTTCTCCTCGTCCGAGGCCGAGACATCGTCCGGCCGCCACCACCACCGCC
GCCTGCGCCCCATCCGCACGGCCGCCGCCGCCCCCGCGCCACCGGCGCCGGAGAAGAAGGCGAGCAAG
AAGCAGCAGGTGGCGCCGGGCGCCTCGATCCGCGCCAAGCTGAGAGACCTCCGCAAGGCGCCGGCGTC
CCCCGGCGCGCGGCTCGCGGGCTTCCTCAACACCATCTTCGCCGGGGGCGGCGGCAAGCGCGCGCCGC
AGACACCGCCTTCCGCGTCCGCGGCCGCGGAGTACGCGTGCTCTACTGCATCCTCGGCGGCGTCGTAC
TCGCGCTCGTGCCTGAGCAAGACGCCCTCGACGCGCGGCGGCGGGGGGCAGCAGCAGGCGGGCCGGAC
GGTGCGGTTCGTGGACAGCGCCGCCGAGGCGCCGGCCACGGTGCCCGGCCGGAGGATGCCGGCGCGGG
CGGTGGAGCAGATGCTGCTCCGAAGGATGGAGATGGAGAGCGACGAGGAGGACGAGGAGAGCAGCGAC
GCCAGCTCTGACTTGTTCGAGCTCGAGAACTTCACTGCTGCTCCGCCGGGCGCGGCAGGGGACGAGCT
GCCGGTCTACGAGACGACCCGGGTCGTGCTCAACCGTGGAAGCATCGGCGGCCACCATGGCTACGGCC
ACGGCCGGAGCGCCAGAGTTGTCTGA
>Zma_GRMZM2G117930
ATGCAGAGCCGGCGGCCGGAGCACGGCAGCTCCGGCGAGCTCGACGTGTTCGGGGCGACGAGTTACTT
TGCCGGCTTGCCGGCGCCTGGTGGTGACCACTGTCGTCGTCCTAGTAGTGTAACAGAGCTGTGCTTCC
AAGCCAATAAGCAGGTGGAGCTTGACACGACGAGGAGCGTGACACGGCACGGCCCTCCACAGCAGCAG
CATCAGCATCAGCGCGCTGGGTGGCATGATGATCACGCATCGTCAGAGCGCCACGCCACCGACAAGCT
CCTGCAGCTGCAGGTAGTAGTGGCCGCCAACAAGCGGCGGCCTTCATCGGCTTCGTCAGGCAAGAGCA
AGCTCGCCGCCCTCTTGAGCTTCATGGTGTCGCCGTCGCCGAGAGCTAGCTTCCGCAAGGAGAATAAT
AGGCAGGAGGCCCTGCCGTCGCCGTCGTCGGCTAGGATGCTGCGGCAGGCGGAGGCCGCACGCGGCGG
CGGCGACGGCGACGGCGAAGAGCCAGAGCCTGCTAGTAATATTGATGGCAGCGCGACGGCGGCCTCCC
CGCCGCCGCCGCCGTCGTCCTCGCGCGAGAGCAGCGCGCAGCTGCAAGGCCTGTTCGGCGCGCTCGAC
GATGATGACGACGACGAGTTTGACCTGGGCGTGGCGACGGGGGACAGGAGGCTGCAGGGCATCACGGT
CGTGAGGGGCGGCGGCGGCGGCGGCGAGGAGAGGTGGGTGGTGAGGTGCTGCGTGCCCGGCGGCGGCG
GCGGCAGCGCCTGGGACGACGAGGAGCGCCGCGAGAAGGCGGTCGACGCAGAGTCATCAAGTGAGCAG
AGCATCAAGCAGGACGAGCAGCTGGTCGTCGTTGAGGCTGAGCAGGCTGAACAAAACATCAAGGACGT
GGTTGTTGAGGTTGAAGTTGAACAAGCAGGAGATGTTGATGATGTCGACGACGACGGTAGTGGTAATG
TTGACGACCCTGCTCACAGTCACAGTGACAGCTGGGACAGCGATTCTAGTTCTGATCTGTTCGATTTG
GATCTCGAGCACAGATGA

>Zma_GRMZM2G438606
ATGGAGAGGCCGTGGAGGGACAAGGACAAGGGAGCGGCGGCTCCGGCGGCGGGGAGGGCGCGGCGGTA
CGGCGACCAGCCGTCCTTCTCGTCCACGCTCCTCGACGCCATATACAGGTCCATGGACGAGCCTGACG
ACGGGGTAACATCGTCCTCCTCAACCGCCGCCAAGAAGCAGAACCAGGACCTGCGCCACAGCTGCTAC
TACTACAAGGCGTCGCTGGCGGCGGGGAGCTACCGCGGCAGCAGCAGGGCGGCGGCGCCCCGGGGGCC
ACAGGCCGCCACCACGTCGAGCTCCTCCGATCAGTGCTCCAGCTACGGCGGGTTCTCGTCGTCGGAGG
CGGAGTCGTCGCAGCACCGGCGCCTGCGGCCCATACGCACGAGCCTCGCCACCACCGCGGTGGCCGTG
GCCGCGCCCGCACTCGCGCCGGAGAAGAAGAAGGCCGGCGTCAGCATCCGCGCCAGGCTGAGGGACCT
CCGCCGCAAGCCGGCGTCCCCCGGCCCCGGCGCGCGGCTGGCGGGGTTCCTCAACGCCATCTTCAGCG
GCAGGCGCGCGCCGCCGTCGGCGTCCTCCTGCTCGCGCTCCTGCCTCAGCGAGACGCCGTCCACGCGG
GGCCAGCCGAAGCGGACCGTGCGGTTCCTGGACAGCGACGGCGGCGGCGAGCGCCGGAGGACGGTGCC
GGTCGGGGTGGCGCCGGAGCTGGAGCAGATGCTGCTCCACCGGATGGAGGTGGACAGCGGCGACGACG
ACGACGACGAGAGCAGCGACGCCAGCTCCGATCTGTTCGACCTCGAGAATTTCGCGGCCGTTGACCCG
GACGGCGGCGCCGCGTACAGTGACGAGCTGCCAGTGTACGAGACGACGAGGCTGGTGCTGGGCCACCG
CGCCATTGGCCACGGGTACGCGCGCACGGGAGGAGTACCCACCAGAGTGGTGTGA

>Osa_Os03g30120
ATGCACCGGCGCCGGCAACACGGTAGCTCCGGCGAGCTGGACGTGTTCGGAGCGACGCGCTACTTCGC
CGGCGTTGCTACGGCCGCCCGTCCCATCGCCGTCGTCGTCGTCAGGGAGCCCGAAGACATGATCATCC
AAGTGAAGACGACGACGACGACGAGCAGCGACAAGAAGACGACGGAGAAGGAAGGCCATCATCACGCC
GGCCAGCTGGATGTGGTCGGAGTCGCCAAGACGACCCACCGGAGCAAGCTCGCCGCCTTCCTCGGCTC

CTTGGTGTCGCCGGAATCGACGAGCTTCCGCAAGAAGCCGCCGCCGGCGGCGAGCAGCGAGACCACGA
CCTACAACTACAACGACGACGACAACCTGCCCAAGATGCAGGTACCCTCGTCGTCGTCCACCTCGAGC
GGCCGTGCTAGCATCGACGTCGCCGCGGCTGCAGCTACCGTGCACGGCGGCGGCGGTGGCCGCCATGA
CGACGACGATCTGGGCGTGGATGCGATGTGGGAGGATCGGAGGCTGCAGGGTGTCAGGGTTGTGAGGT
GCGGGCGCTGCGACGAGGAGCGGTGGGTGGTGAGGTGCGGCGCTTGTTGTGCTTGGGAGGAGGAGGAG
GAGGAGCACCACCATGGCCATGAAAAGAAGGCCATTCTTGCTGCTGCGGCGACATCAACAAGGTACGG
ATCTCATCAGGTGCTAGCAGGTGATCGAGAGGTTGTCGGCGATGGTGCATGTTCTGATTGGGAGAGCG
ACTCTAGCTCTGATCTGTTCGAGCTGGATCTTGAAATCACCTGA

>Sbi_Sobic.001G257100
ATGGAGAGGTGGCCGCCGGTTCCGGCGCCGGAGAGGCCGAGGCGGCGGCCTGGCCAGCCGTCCTTCTC
GTCGACGCTGCTGGACGCCATCTGCGACTCCTTGGACGAGCAGGCCGGCGGCCAGGGAGCTGCGGCGG
AGCGTGCCGCCGAGGCACCAACGCCTGGCAGCGCCAAGAAGCAGCAGCAGGCGGCCCTGCATTACTAC
TACTACTACAAGCCTTCCTTGGCCGCTAGCCACCGGGCGGCGCGTGCCGCGCCGGCGCCCGCCGACGA
CTGCTCCTCCGGCCGTGGCTACTTCTCGTCGTCCGAGGTCGAGTACTCCCTCCGCCGCCTCCGCCCCA
TCCGCACCTCTGGCGGCGGCGTCGGGCCGGCCTCGGTGGCTCCGGCGGAGAAGCAGCAGGCGGTCCCG
CCTGGCTCGGCGACGGCGAGGAGGGCGCGGAAGCCGTCCGCCGCCCCCGCCAGCCATGGAGGCTGCCG
CAGGCCGGCCTCTCCCGGCGCGCGGCTCGCCAGCCTGCTCAACGCAATCTTCTCCGGCAAGCGGCATT
CCGCGCGGCAGCACCCGGCTCCGGCGGACGACGAGCCGACGGCGTGCTCGACGGCGCCCTCCAGCGCG
CGCCCCTGCCTCGCCAAGACACCGCCATCGGCGACGGCGCGGGCGCGCGCGACCCACAGCCGCAGGAG
CAGGACCGTGCGGTTCTTGGACATCGAAGGCGAGGTGGCTGTGGCAGCGGCCGCCGCTGGCTGCAGGC
GATTCCCGGTGGTGGAAGTGGAGGGCAGCGACGGCGGCGAGGAGAGCAGCGACGCGAGCTCCGACCTG
TTCGAGCTGGAGAACCTCGCGGCGCTCGCTCCGGCGAACGGCGGCTCCGGTTGCCGTAGAACGTGCGA
GGACGAGCTCCCGGTGTACGGGACGACTGGAGCTGGGCTTGCGCAGGACATTGGACTTGTCCGTCGCC
GCCCGTTTGGGTACGTCAGCCATGGTCGGAGTTGCAGAGGCTTATTTGATTTCAAATAG

>Zma_GRMZM2G438606.2
ATGGAGAGGCCGTGGAGGGACAAGGACAAGGGAGCGGCGGCTCCGGCGGCGGGGAGGGCGCGGCGGTA
CGGCGACCAGCCGTCCTTCTCGTCCACGCTCCTCGACGCCATATACAGGTCCATGGACGAGCCTGACG
ACGGGGTAACATCGTCCTCCTCAACCGCCGCCAAGAAGCAGAACCAGGACCTGCGCCACAGCTGCTAC
TACTACAAGGCGTCGCTGGCGGCGGGGAGCTACCGCGGCAGCAGCAGGGCGGCGGCGCCCCGGGGGCC
ACAGGCCGCCACCACGTCGAGCTCCTCCGATCAGTGCTCCAGCTACGGCGGGTTCTCGTCGTCGGAGG
ACGGTGCCGGTCGGGGTGGCGCCGGAGCTGGAGCAGATGCTGCTCCACCGGATGGAGGTGGACAGCGG
CGACGACGACGACGACGAGAGCAGCGACGCCAGCTCCGATCTGTTCGACCTCGAGAATTTCGCGGCCG
TTGA
>Pda_XP_008791915.1
ATGGAACGGTGGGAGAGACCGCCGTCGCGGCGCGGCCGCAACAAACCTTCCTTCTCCTCCTCCCTCCT
CGACGCCATCTACCGCTCCATCGACGAATCCGACGGCGGCGGCGGCGGAGGACCAACGCGGAGTCACG
GCAAAAGCGGAGCGCCTGACCCATCCTTTTCCGTCTCCTCCTCCTCCGCCGCTGCCGCTAGAAAGAAG
CAGAAAGCGGACGAGCTCAAGTGGACGGCTGCGGCAGTGAGCGAGAGGGCGGTGATCCGACGCGGCAC
GGTGGACTACAGACCCGGTCTGGTCACCCCAACCTCCAGCTCCTCGGATTCCTCCAGCTACGGCGGCT
TCTCCTCCTCCGACGCCGAGTCCTACTACTCGGCCGGGGTCAGGCCGGTCCGATCGGACCGGATCCGG
TCCGATCCGGAGATCCCGCCGGAGAAGAAGAAGGCCGGGTCGATCCGGAGCCGGCTCCGGGATCTGAG
GAAGAGCCGGGCCACCGGCCCGACGTCTCCGGGGGCGCGGCTGGCGAGCTTCCTCAACTCGCTCTTCG
CCGCCGCCGGAAACCCCAAGAAATCCAAGATCTCCGCGCCGCCGCCGCCGCCTGCAGCGGCGGTGGAG
TCCGCGTGCTCGTCGGCGTCGTCGTACTCGCGGTCGTGCCTGAGCAAGACGCCGTCATCGAGAGGGCG
GCGGACGCCGGCGGCGGCGTCCGCGGAGGGGGGCAAGAGGTCGGTGCGGTTCTGCCCCGTGAGCGTGA
TCGTCGGCGAGGACTGCCGGCCGTGCGGGCACAAGTGTCTCGTCGACGAGGATCTGGCGCCGCCGCCG
GCGCCGTCTGTGGTGGCGAGAAAGGTGGAGAAATTGCTCCGGGGGTTGCAGAAGGAGGAGGACGAGGA
GGAAGAGGATGCGATGAGCGATTCGAGCTCGGATCTGTTCGAGTTGGAGAATTTGACGGCGATCGGAC
GGTACAGGGACGAGCTCCCGGTGTACGAGACCACCGATCTGGGGACCAATCGCGCGATCGCCCAGGGT
TTAGTTGCGTAA

>Mac_Ma06_t26310.1
ATGTCCTCTTCCTCCCAGTCCTTCCCCTCAGTGATGTCGAAGGGTTATAGCCAAGCTGACTCCGACGA
GATCGACGTCTTCGAGGCAACATGGTACTTCTCCGGCGGAATCGATGGCGCAGGCCTCGGCCTTCAGA

GATCCATGAGAGAAGAGAGACTGATCAGCTGGGGAGGAAATAGGAGTTTGGACACACTTGCAAGGTCC
ACCAGACTTCCACAGCAGTCCAAAAAGGTTGAGAACCAACGCAATGACAAGAAGAGCAGACAGCCCAG
CTCGGCCGGTCGTAGGTTAGCTAGCTTCCTCAACTCTTTCATCAAGCAAGCCATCTACAGGAAGAAAT
CAAGGGCTCTCAACCCCACTGAGTCAGAGGAGGACGCAAGTTTTGAGAAGATGCATGCAGGGAGGAGA
AAAGGTAGCATCAACGACTCCCAGCGCATGAAGAGAAATGACTCCAGCATCCTGTGTTCCACAGAGCG
TTGCTGCAATAGCAAGTCTAGTGAGCACCAGAAATATGCTCCTTTCTGCTCACAGAGAGAACTTGGGT
GCGATAAGAGGGTAATAGATGAGGATTGGCTGGTTGAGAGAGCTAAATCCATGGATGGTTATCCTGCG
AACAAATGGCTAACTAGTAAAGCTGGTAATCGGCTTCTTGACAAAGAAGCATTGTGGAGTGAAGAGTT
CATGAAGAAGCAACACAAGTGGTTCAGGAGGACAGAAGAGGAGGATCGGGGAGGGAGTGAATCCAGCT
CTGAACTGTTTGAGCTGAAGAACTATGATCTGGGGAATGTTGCCTTTCGTTGA

>Mac_Ma05_t08640.1
ATGACAAAGGCTCACCGCCAAGGTGACTCCGACGAGATTGAGGTTTTCGAGGCGACACGATACTTCTC
CGGTGGAATCGGTACCTGCGAAGGCCTTGGCCTTCAAGGAAGCACGAGGGAAGAGAGAGTGAGTTGGG
GAGCAGGTAGGAGGAGTTTGGATACACTGAGAGCCATACTCCCAGGCCGGTCCCGAAAGGCCGACGAC
CAGTGCAAGGAGAAGAAGAAGAACAAGCAGCGAAGCTCCCCTGGTGGTAAGCTAGCTAGCTTCCTCAT
CTCTCTCTTCAAGCAAACAATCTCTAGGAAGAAATCACAGTTCAAAGATCGACGTTTCGAGGAGATGC
ACACAGGGCGGAGGAGAAGCAGCGTCAGCTGTTCTCAGACTGTAAGAAGTAACGAGCTGGATTACTCC
AACAGCAAGCCCAGTGGGCACCAGATCAAACCTGCTCATTTCAGCTCACACAGAGAAGCTTGGTACGA
GAGAGCGAAGTCCATGGATGGTTATCCTGAGAACAAATGGGTGATCGATGGAGTTGCTAATGGGCAAG
ACAAGGAAGAGTTGTCCATGCCGACGGAAGATGATGGAGGATGGAGTGAATCCAGCTCTGATCTGTTT
GAACTGAACACCTTTCATCTGGCGAGTGATCCGTTCACAGATCTGCCCGTTTATGGGAGCCCAAACTC
GGATACACTCGAGAGAGCTGCTTCCATTGCTGGTGCTGCTTCGTAA

>Mac_Ma04_t03330.1
ATGTCTTCTTGTTCTGGTTCCTTCACCTCAGGGATGTCGAGGTCCCGCCGTTGGAACGACTCCGGCGA
GCTTGATGTCTTCGAGGCAGCACTCTACTTTTCTGGCGAGGTTACTGATGTAGATCTTGGACTTCAAA
GAGCCTGGAGAACTGAGAGGAGGGGTTCGGATACACCCACGGAAGCCACACTCTTCCATCAGCCCCGA
AAGGTTGAGAGTCGATTCAAAGACAAGAAGCACAAGCAACCCTGCTCCCCTGGTGGCAAGTTGGCCAA
CCTTGTGAGCTCCTTCTTCCACCAAGCAGCTTCAAAGAAGAAATCAAAGGCCGTCTCCCCTTCGCAGT
CATTTAAGGAGGAGTACGGGGAGAGGCTCTTAAGGAGGAGGAGAAACAGCATCAACCATCCAGAGATC
ATGACAGGTAGAGACTACTCCAACTCTATCTTTTCTTCTGACAGAAGAAGTGGGACTGGAGTCTCTTC
CCCTTACCCTATTGTCCCTTTAAGCTCGGAGCGAGAGAATTGGTACGATAAGAGGGTGGCGGGTGCGT
TTCTGTTGGCCGAGAGATCGAAGCTCATTACTGATGGGTTTGCCGGGAACACATGGGTTGAGAAGGGA
AGCTATCGCATGCTACATAACAGGGAGGCTCATCAGTGGGCTGAGGCGTTCATAGAAAAGGAAGACAA
GTGTAGGAGAAGAGAGGAGGAGGAGGAGGAAGAGGACGATGGGGGGAGTGATTCCAGCTCAGACCTGT
TTGAGCTGAAGAGCTATGACTTGGTGTTTTGA

>Mac_Ma06_t12410.1
ATGCATGGAGTCGACGCCTGCATGTCTTCCCTGATGTCCGGCAAGGGATTCTCCAGCATGAATCACCG
CTACAGGGAGCGATTCAACAGTTGGAATGAGTCCGACGAGCTCGGCGTCTTCGAGGCGACGCGGTACT
TCTCTGATGCGACGACGGCGTAGGTCTCGTAGGTGGATTTGGCCCTCGTGGAGCTGTTGCAGTCGAGC
AGACGGTGCCCAGGAGCTCAGATGGGAGACTAATTAGCGGCAGTCCCTCGCTGACGAGAGCCAAGAAG
TGCGGCAAGCAACCTGGCTCCCCTGGAGCTAAATTAGTCGGCTATCTCAACTCTTTCTTCCATCAAGC
GGCTTCTCGGAGGAAGCCGAAGTGTTCGAATCCCACCTCGACGTCCAGAGAGCCACGAGAGGATGGGG
AAGAGGTGGAGAAGAGACCTGGAGATCAGAAGGAGAGAAGAAGAAGCATCACCGGCCATTCTCAAAGC
ACCAAGACCGCTGGCACCAAATCCAGCAATTTCTGCCCACGAAGTGAGGTAAGGGGAAGGAGAAGAAG
TATCACGGAGGTGTCAAAGGAGAGAGAAAACTGGGAGAACAAACAGGTGCCGAACGGTGCATGGCCAA
GGGGAAGAAATAAGACCTTCGGTAGAACAGGAAAAGAGGTGGAAGATGATGACGGGGACTCCGACTCC
AGTTCCGATCTATTCGAGCTGAAGATTTGCGATCGCGGCGGCCTCTCCGACGGCTTGCCCGTCTTTGC
AACCACAGATATCCAAGCTATCAAGAGAGACACTGCGATATCAAGTTCTGCATCTTAG

>Mac_Ma09_t15430.1
ATGGAGCGATGTAGCCGATCGAAGAACCCGTCGTTCTCGTCGTCTCTGCTCGACGCCATCTACCGCTC
CATGGACGATGAGCACGGCGATAAGAGCCACCGGAGAGCACCCGGCCGTTCCTCCAACCACAAGAAGC
AAGCGGAGCAACCCACGGTGGCACCCTCCACCGATCACTGCCATTACCGACCCTGCGTCACCGAATAC

CACCGCCGCAGCGTCCCTCCCACTTCCAGCTCCTCCTCCTCCTCTTCGGCTTCCTCCACCACACCCAC
CGCCGCCACCACAAGCAGCAGCTCCGTCGGCTTCTCCTCGTCGTCCGACGTCGAGTCCATCCGATCGG
ACCGCATCCCTCGGCCCGATCTCCTGGACCCGGGGAAAAAGAAGAAGAAATCCAAGTGCGGATCGATC
CGTACCGGGCTCCGGAGTCTGAGGAAGAGCCGGGAGCCCGCCTCGGCTGCGGTGGCAGGATCGTCGTC
GGCGTCTCCAGGAGCGCGGCTTGCGAGCTTCCTCAACGCCCTGTTCGCCTCCGCGGGGAGCCCCAAGA
AACCCAAGATACCTATTCCCGCCGCTGCGTCGGTGGCGGCCGCGGCAGGTGGAGGTGGGGAGGACTCG
GCGTGCTCGTCTTCCACGTCCAGTTGCAGGCGCTCGTGTCTGAGCAAAACGCCAACGGCGGCGGACCG
GCGGCGGGCGTCGGGGGCCGATGCGGGGAAGCGGTCGGTGCGGTTCTACCCGGTAAGCGTGATCGTCG
ACGAGGACTCCCAGCCCTGCGGCCACAAGCGCCTCCAGGACGACGCGGGCAATACGGCGCCGCCGGTG
GCGGCGAGGGTGGAGGAGCTGCTGAGGGCGGCGGGGGCGGAGGTGGAGGCAGAGGAGGAGGGCGACGG
CGACGGAAGCGAGTCGAGCTCGGATCTGTTTGAATTGGAGAATCTGACGGTTATGATGCGCGGTGGAC
GGTTCCGCAATGAGCTACCCGTGTATGAGACCACCGACCCTCACACTAATCGCGCCATCGCTCAAGGT
TTGATCCATTAG

>Mac_Ma07_t02220.1
ATGATGGAGGGGTGGGAGAGACCTCCGCACCGTCGCAGCCGATCAAAGAACGCGTCGTTCTCCTCTTC
TCTCCTTGACGCCATCTACCGCTCCATCGAAGACGACGACGTGGATAACAGCAAAAGCGGAGAGACGA
GGGGCGGAGTACCCGACCGCTCCTTTTCCCACAGGAAGCAAGTGGAACCGGTGACGCACTCCCGAAAC
GGGAGCAGAGCACCCGTAGTGAGCGAGCTGGCGGTGAGCCGTGCCGGCGTCACCGACTACTACCGCCG
CAGCCCTTGTGGCGTCGGCACATTCGCTCCGACTTCCAGCTCCTCCTCCTCCTCCTCCTCGTCCATCA
CCCCTAGAATCAACGCCACAGGATTGTTCTCATCGTCAGACGGTGAGTCCAACCAGTCGGACCGCATT
CCTCGGCTCGATTCCCCTGCCACGAAGAAGAAAGCGAAGCCCAAGTGCAGCAACCTCCGTAGCGGGCT
CCGGGGCCTGAGAAAGCCCCGGACGGCGTCGGACGCAGCTGTAGCGACGTCGCCGCGGGCGCGGCTGG
TGAGATTTATAATCGCGCTCTTTTCCGCCGCCGGGAGCTCCAGGAAGCCTAAGATCACCGTCCCCTCC
GCCGCGGCGGCCAAAGATGGGGCCCGAACGGAGGAGTCGACGACGACATCCACCTCGGCTTCGAGCTG
CATGGGTACGTGCCTGAGCAAGGCGTCGGAAGCGTCACGGCGGCAGGCTGCGGGAGCGGAGAAGGGGA
AGAGGACGGTGCGGTTCTATCCGGCGAACGTCATCGTCGACGAGGACTACTCGCGGCCCTGCGGCCAC
AAGCGCCTCCAGGACGGTGCCGCCAATGCAGCGGCGAGGGTGAAGGAGCTGCTAAGGCTGGGACGGGA
GGAGGACGACGACGCCGGGGGGAGCGAGTCAAGCTCGGATCTGTTCGAGCTGGAGAATCTAACGGAGG
TGATGGGGGAAGGGAGAGGGGGAGGAAGGTGCAGGGATGAGCTCCCCTTGTACGAGACCACCAACCTT
AACAAAAATCGGGCCATAGCACGAGGTTTG
ATCCTTTAG

>Mac_Ma01_t15370.1
ATGGAAATGGAGAGATGGGGGAAGGAGCGGCCGCGGCGTGGCCACGAGAATCCGTCCTTCTCCTCCAC
GCTGCTGGACGCCATCTACCGCTCCATGGATGAGTCGGACGGCTGCGGTGAGCCTAACCCGTCAGTCG
CCGCCCTCGCCTCCCCCAGGCTGCCCGCCCCGTCCTACCGCTCTGCCGCGGCGGTGAGCGGGAAGGCG
GTCGCCCCACCCGTCCGCCTCCCGCCCATCTCCACCTCCAGCTCCTCCGACAACTCCAGCTACGGCGG
CTTCTCCTCCTCCTCCGAGCCCGAGTCTGCATCCAGCCACCGCGCCCGGTTCAGGCCTATCCGGATCG
GCGTGATGGCGCCGGATCGGTCCCCCTCCGTCCTCCGTCCTCCTCCTCCTCCCCCGCAGCCCGCCGTC
CACCACAGCGAGAAGACGAAGTCGGGCTCGATCCGCAGCAATCGCAGCGATCCGGGGCGACCCAAGGC
GCCGGCGTCGCCGGGGGCCCGCCTCGGCCGCTTCCTCAACGCCCTCTTCTCTGCCGCAGCGAAGAACC
CCAAGAAGTCGAAAACCTCAACCTTAACCGTCGCCGCCGCCGTCCACGTCGGCGACCCGGCCTGCTCC
ACGGCGTCGTCCCACTCCCGGCCGTGCCTCGTCAAGACGCCGTCGTCGCGACGGGCGCCGGGGGCGGA
CGACGAGGGCGTGAAGAGGTCGGTGAGGTTCCACCCGGTGAACGTGATCATGGGCGAGGACCTGCGGC
CGTGCGGCCAGAAGAGCGTCTACGCGTGCGATCGTGCCGCGGGGGCCGGGACCGAGATCAGGCGGCGG
TCGTTCGCGACGGAGGCGGCGAAGGGGAAGTCGAGGGATGAGGCGAGGAGAAGGGTGGAGGAGCTCCT
AAGAAGGTTCGAGGACGAGGAAGACGACATGAGCGACGCGAGCTCGGATCTGTTCGAGCTGGAGAATC
TGACGGTTATGGGTGCGGGAGGCGGAGGCGGAGGTGGTTACAGGCACGAGCTTCCCGTTTACGAGACG
ACGCATCCCGGTACCAATCGCGCCATTTCTCGTGGTATTGTCGTGTAA

>Mac_Ma03_t07780.1
ATGGAGCGATGGGAGAGAGCGCCGAACCGTCGTAGCCGATCGAAGAACCCGTCATTCTCCTCCTCTCT
CCTCGACGCCATCTACCGATCCATCGACGAAGAACACTCCGATAACAACGGCCACCTTCGAGCGCCCG
ACCGCTCCTCCAACCACAAGAAGCGAGAGGGGCAACCCATGCCGGCACCCTGCATTGACCGGTGCCAC
TGCCGGTCCAGAACGATGTCCCCCGCGAGCGAGCGGGCGGCGAACCGATCCAGCGTCACCGACTACCG

CCGCCATGGCTCCATCGCCAGAGGCAGCTTCACTCTCACTTCCTGCTCTTCTTCGTCCTCGTCAACCA
CGACCACCGCCGCTGCCAGCAGAACTAGCAGTTCCACCGGCTTCTCCTCATCGTCCGATGCCGAGTCC
ATTCGGTCGGACTGCATCCCTCGGCCCCATCACCCTACACCGGAGAAGAAAACGAAGAAGGCCAAGTG
CGGCTCGATCCGTAGCGGGCTCAGGGGTCTGAGGAAGAGCCGGACGCCTGACGCGGCATCGATGGCGG
CGCCATCGCCAGCGTCTCCGGGTGCCCGGCTGGCTAGCTTCCTCAACGCGCTCTTCGCCTCCGCCGGG
AGCCCTAAGAAGCCCAAGACCCCCACTCTCGCCGTCGCGACGACGGCGGCGGCAGGCTGCGGAGACAG
CGAGGACTCGGCGTGCTCGTCGTCAGCGTCGAGCTGCAGGCGGTCCTGCCTGAGCAAGGCACCAGCGA
TGGCAGACCGACGGCGAGCGTCGGGAGCCGAGGCGGGGAAGCGGTCGGTGCGGTTCTACCCAGTGAGC
GTGATCGTCGACGAGGACTCCCGCCCCTGCGGCCACAAGCGACTCCAGGACGACGCAGAGGGTGAGGC
GGCGCCGGTGGCGGCGAGGGTGGAGGAGCTACTGAGGGCGGCGGGGGCGGATGCGGAGGCAGAGGAGG
AAGGAGAGGACGGGGGCAGCGAGTCGAGTTCGGATCTGTTCGAGCTGGAGAATCTGACGGTGGTGATG
CGAGGAGGAGGGTACCGTGATGAGCTACCCGTGTACGGGACCACCGACGTCAGCACTAATCGGGCGAT
CTCTCAAGGTTTGATCCACTAG

>Mac_Ma10_t18560.1
ATGAATCACCTCCAGGAGAAGTCATTCGACAGCTGGAATGAATCCGACGACCTCGACGTGTTCGAGGC
GACGCGGTACTTCTCCGGTGCAATCGATGGCACGGGCCTGCAGGTAGGTGGATTTGGCTCCCATGGAG
CTGTGACGGTCGAAGATAGGGTGCCAAGCAGCGCGCGGAAGGGAAACTTAGATGAGAGACAAATAAGC
AGCAGTTCCTCGCTGATGAAAGACAAGAAGTGCAAGCAGCCCAGCTCTCCCGGTGCTAGGTTAGTCGG
CCTACTCAACTCTTTCCTCCATCAAGCGGCTTCCGGGAGGAAGCTCAAGTGTTTGAACCCCACCTCGA
CGACCAGAGAGCCACCGGAGGAGGGGGATGCGAAGGAGGAGAATAGCCTTGTGGGGAGAGAGAGAAGA
AGAAGCATCAGCCGTTCCCAAAGCACCAACAGCACTGGTTCCAAATCCAGCAAGTTCTGTGGCAGCAG
CAGCTTTACGAATCCAGACTTGTATCCATACAATTACCGTACTACTCCATCGATGAGTTACAAGTGCA
ACCGGGGCCAAAAATCTGTCACCTTCTGCCCACGAATCGAGGCATGGAGAAGCACAAGCATCACCGAG
CTGCTGCACAACAGTGGAGTACAAAAGGAAAAGGATTACGGACTCCACGGAATTAGAACCGAGGAGAG
AGATGCGCATGACAGGGAGAACAAAAAATGGGTACTGAACGACCCCGGCGATCAGTATCCGGGAAGAG
ATTGGTTCGGGGATTATGATCTTCCATTGCCAGGGGTAAGAAAGAACTCGATCAGAGGGGAAGAAGAA
GAGGAGGAGGAGGTGTCTGACTCCAGCTCCGATCTGTTCGAGCTGAAGATCTGTGATCATGCTGTGCT
CTCGGACGGCTTGCCCGTCTTCGCAACTACAGACATCGAAGTCATCAACAGAGCAAGTACTGCATCTT
AG

>Mac_Ma10_t17700.1
ATGGAAGAGAGGTGGGCGAGGGGCAGAAATAGGAGCGGTCACCAGAATCCCTCCTTCTCCTCCACCCT
CCTCGACGCTATCTACCGCTCCATCGATGAGTCCGACGGCGGTGGCGCCACCCACGCTCCTATCATCG
TCCCGAAGCGCCCTCCGCCGCTCCTGCGGCCCCCGGTGGACCGGCGAACCGCAGGGGCGGCAGACGAG
AGGGCGGCAACCCGTCGCCGCCCGTTTGTGCCCATCTCCACCTCTAGCTCCTCCGAAAAGTCCAGCTA
CGGCGGCTTCTCCTCCTCCTCTGAGCCCGACTCGGCGGCGACCCGGCTCAGGCCAATCCGGACTGCCG
GGCCGCCGATCCATTCGGCCCCTCCTCCGCCCGCAGCGGCGTCGTTCGACCGCCACGAGGAGGATGAA
AAGAAAAAGAAGAAGAAGACGAGCTCGATCCGCGGCAGGCTTCGGGACATGAAAAGTTCCAGGTCGGC
GGCGCCGGCTTCGCCTGGGGCTCGCCTTGCTGGCTTCCTCGGCTCTCTATTGTCGGCGGTGTCGGGGA
CCCCGAGAAGGCCGACGTCCACCGCAAGCGGATGCGACGACTCCGCCTGCTCCACGGCGTCGTCCCAC
TCGCGATCGTGCCTCGTCAAGAAGCCGTCGACGAGGGAGCGAGCTCCTCCAGCGGAGGGGGGGAAGCG
ATCGGTGAGGTTCTATCCGGTGAGCGTGATCGTCGACGAGGACCTGCGGCCGTGCGGGCATAAGAGCG
TTTACGAGGCGGATCGGGCAGCGGAGACATCGTCGCGGCGGCCATCGTCGGCGGCAATGGAAGCGAGG
AGGAGGGTGGAGGAGCTCCTGAGAGGGATGGAGGAGGAGGAAGAAGAGGAGACGAGCGATTCGAGCTC
CGATCTGTTCGAGTTGGAGAATCTGACGGTTATCGGAATGGCAGGGGGAGCAGTGCACAGTGATGAGC
TTCCCGTGTACGAAACCACCCACCTCGACACGAATCGCTCCTTTTCGCAATCTCAACGCTTCCTTCAA
ATATAA

>Mac_Ma07_t11170.1
ATGGGGAACGAGGCATGGCGGCTCCGTCACGGGCACCGCTCCTTCTCGTCGTCCCTCCTCGACGCCAT
CGAGCGCTCCATGGACGACCCCATCGCCCCCAAGAGCGAGGAAATGGCGAATCGCCTCGCTCACGGAG
CATCCGACTTCCTGGTTCCCCTCGCCTCCATGGAGCGGAGGCCCACGGCGACCGTGACCCGACCCCGG
CGTCCAGACTTCCCCCCGTTCTCCACTTCCAGCTCATCCTGCAACTCCACTTCGAGCGGCTTCTTCTC
CTCGTCCTCCGGGCGTGAGTCCCCCGCCACCCTCCCACTCAGACTCCGACCGAGCCGGTCGCCGCCAC

CCGACCAGCACCGCCAGCAGGACAAGGAGAGATCGGGCTCGACTCGGACAAAGCTACGAGGTCTGAAG
AAATATAAGGCGCCAGGGTCGCCAGGGGCCCTCCTCGCGGGG
TTCCTCAACTCACTCTGCACCGCGGCGGCGGGGGACCGGGCGAAACCGAAGCCATCACCCTCCGGCGC
GGACTCCGCCTGCTCGGCCGTCTCGTCGCGGGCGCGACCTTGCCTTAGCAAGGCGCCATCGACGAGGG
ATCCAGCGGAGGGCGGGAACAGGTCAGTGAGGTTCCGCGCCGATGGGGAGGATCCGCGGCGATGCGGG
CAGACGAAGAAAAGCGCGTGCGGCGGGGATCACGCGGTGGTGGAGGCGAGAGGGGTGAAGATGAGGGT
GGAAGAGCTCTTGCGAACGTTGGCGGAGGAGGGAGAAGAGGAACAGGACGATCTGTTTGAGTTGGAAA
ACTTGATGGTGATGGAAGGGGGTGGTTACAGGGACGAGCTTCCGGTGTACGGCACGACCCGTCCCGAG
AAGAATCGCTCGAGGTCTAACTGA

>Mac_Ma05_t16240.1
ATGGAGAGATGGGGGAAGGAGCGGCAGCGGCGTGGTCACGAGTGCCCATCCTTCTCCTCCACGCTGCT
CGACGCAATCTACCGTTCCATGGACGAGTCCGACGCCGGAGGAGCGCCCGAGCCTTCCCTCGCCGCCT
CGAGGAGGCCCGCCATGGTGGTCAGCGAGAGGGCGGGGAGCCGACCCCGTGGCCTCCCGCCCATCTCT
ACGTCGAGCTCCTCAGATAATTCCAGCTACGGGGGCTTCTCCTCCTCCTCCGAGCCTGAGTCGGCGTC
GAGCCACCGAGCCCGACTCAGGCCTATCCGTAGCGGGGAGGCGCCGGCTCGCTGCACCGCCGTCTCCA
GCCCTCCTACTCGTCCTCCCCCGCCGCCACCGCTGCAGCAGCATCAACGGATCTCCTCGCCGCCGGTC
GTACACCACCGCGAGAGGACGAAGTCGAGCTCGATCCGGAGCAAGCTCTGGGATCTGGGGCGATCCAA
GGCGCCAGCGTCACCAGGGGGCCGCCTCGCCGCCCTCCTGAACTCTCTTTTCGCCTCCGCAGCCAAAA
GACCGAAGAAGTCCAAAACCCCGACCGCCACCGCCGCGGGCGCCGTCGGCGGCTACGACGACTCGGCC
TGCTCCACTCTGTCATCTCACTCCCGATCGTGCCTCGTCAAGGCGCCGTCGTCGAGGCGGGCACCACC
GGCGGAGGACGAGGGAGCGAAGAGGTCGGTGCGGTTCCACCCGATGAGCGTGATCGTGGGCGAGGACC
TGCGGCCGTGCGGGCAGAAGAACGTGTACGCTGGCGATCGGGCGGCGGAGGGGAGGCGGCGAACGGTG
GCGACGGAGGTGGAAGGGAAGGGGAAGACAAAGACGAGGATGAGGGTGGAAGAACTCCTGAGAAGGTT
CGAAGACGGGGAAGAAGAAGACGGAGAGATCAGCGACTCGAGCTCGGATCTGTTCGAGCTGGAGAATC
TGACGGTGATGACAGGGGGAGAGGGATATAGGGATGAGCTTCCAATGTACGAGACTACGCATGCCGCT
ACTAATCGCGCCATTTCTCGTGGACTCGTCCCCTAA

>Mac_Ma06_t13000.1
ATGGAAGAGAGGTGGGCGAGGGGAAAGCCCAGGAGCCGTCACCAGAACCCCTCCTTCTCCTCCACCCT
CCTCGACGCCATCTACCGCTCCATCGACGAGTCCGACGGCGGAGCCACGCGAGACCGCCACTCTCTCA
TCACCGTGCCGAAGCGCCCTCCGCCGCCCCTCCGGCCCGCGGCAGAGTGGCGAACCGCAGAGGCGGCG
ACCCGTTGCCGGCCGCTTGCGCCCATCTCCACCTCCAGTTCCTCAGACAAGTCAAGCTACGGCGGCTT
CTCCTCCTCTTCCGAGCCCGACTCGGGGGTAAACCGGCTCAGGCCGATCATGACCGTCGGAGCTCCGA
TCCGCTCCATCCCTCCTCCGCCGGCTGCTGCGGTGTTCGACCGCCGTGAGGAGGAGAAGAAGAAAAAG
AAGACGGGCTCGATCCGCGGCAGGCTTCGAGACGCGAGAAGTTCCAGGTCGGCCGCGCCGGCGTCCCC
GGGCGCCCGCCTCGCCGGCTTCCTCGGCTCTGTATTGTCGGCGGTGTCGGTGATCCCGAGAAGGCCAA
CGCCCACCGCTGTCACCGCCGCGGGCGGATGCGACGACTCCGCCTGCTCCACGGCGTCGTCCCTCTCG
CGATCCTGCCTCATCAAGAAACCATCGACGAGGGAGCAGCCTCCGTCGGGGGAGGGGGAGAAGCGATC
GGTGAGGTTCTACCCGGTGAGCGTGATCGTGGACGAGGATCTGCGGCCGTGCGGGCACAAGAGCGTCT
ATGGAGCGGACACCGCGCCCCGAAGGCCGTCGGCGGTGGCAATGAAGGCGAGGAGGAGGGTGGAAGAG
CTCCTAAGAGGGATGGAGGATGAGGAAGAGGAAATGAGCGATTCAAGCTCCGATCTGTTCGAGCTGCA
GAACTTGACGGTGATCGGAAGGGAAAGAGGAAGGGGAAGGGGAGTGGGAGGAGGTTACGGTGATGAGC
TTCCGGTGTACGAGACGGCTCACCTCGACATGAATCGCTCCACTTCCCAGTCTCAACGTTTCCTAAAA
ATACAAGAAAGAATGTAA

>Zma_GRMZM2G088860_T01
ATGGAGCGCCGCGGAAATTGCCACGGCGGCAAGCACCCGCCGCCGCGCCAGGCGCGAGGGCCCGGGGA
GCGCACCCGGCAGCCGTCCAGCGGCGGCTCCTTCTCGGCATCGCTCTTGGACGCCGTCTACCGCTCCC
TCGACGACGGCGATGGTGCGGACGTCGTCGTTGACGCCGCGCGTGGGAGTGGTGCGGAGGAGAAGGCG
GCCGCGACGGCGCAGTTCTGGTGGGCGAATAAGGAGGCGGCCACGGCCAGACCCAGGCAGTCCTCGTC
GAGCGCGGACGGGGACAGGCGACGCCGGGAGACGGGAGCTGCGCGCCCGCGCCACTCGGGGTACGCGT
CGTCGACCACGTCGTCGTCCGACTCGTCGGCTAGCTACAGCAGCTTCTCCTGCTCGTCGGCGTCGACC
ACAGACACTGAGTCCACGTGCCGCCGCCACAGCCCCCCGCCGCCGCGGATGTCATTGTCAGAGGAATC
CGCCGCCACAGACGCCGAGGAGGCAACCGCAACCCCTCCACCGCCACCCAAGAGCAAACCGAAGAAGA
AGGCCAGGCCGTGTTTCCCCGTAGCAAGAATCCGACCACCAAAGGCCTCGGTGCCATCATCATCGTCG

TCTGGAGCGCAGCCGCCATCGCCGGCGACGTTCGCGTGCGCCCTTAAGGCTCTGTTCTCCTCGGTGCG
CCTCCAGAGGAAGCCCAAGGCTCCGGCTGCAACCCCTCCGCCCAAAATCTCGCAGCCGCAGCCGCAGC
CACAGCCTCCGAGCATGTCGGCGACGAGCACTGCGAAGGCGGCTGACGCGCCGGCGGAACCGTCGGTG
CAGAGGACGGTGAGGCTCCGCCCGGAAGCCGAGGTGTCGGTGGTGCGGAGAAGGGTGGTGGAGGAGCT
GGTGCGGAGCCTCGAGGAGCTGGAGGCGGACGAGGAGGGGAGCGACGCCAGCTCCGACCTCTTCGAGC
TGGACAGCCTTCGTGGCGCCGGCGCGGACGAGCTGCCCGTGTACGGCACGACCAGCCTCGTGGTGGCC
AACCGCGCGATCGCCCAGGGACCAGCTCGTTGA

>Zma_GRMZM5G886335_T01
ATGCCTCCGCACGACGGCGCCCGAGCCCCGCCGCCCCGGCCCAGCCGGGGCCACCAGCCGTCCTTCTC
CGCCGCGCTCCTCGACGCCATCTACCACTCCCTCGAGGCCGACGCCGAGGCGCGCACGTCCACAGAGG
CGCGCCGGACGCGCACGCCGGCGTCGTCCCCGGCGCAGCTTCCGAGCCGACGTCGCCCGACGCCCGAG
CTGTCGCCGTCGCCGTCCCCGTCCCGCTCCTCGGTCCGGTCACCGCGGCTGCAGAGGGCGCCGCGGCC
GTGCCGCGTCCGGCCGGACCCGCAGCCCAACTCCAGCGGCTCCCTCCTCCTGCCTCCGCCCCTTCCGC
CGCACCCACACGAACACGAGCCGTCGTCGACCGGGCACCGCCGCGTCGCGGACGCGGAGAGGAAGAGG
GGCCGCGGGAGGAAGAGCAAGAGGACGGCGCCGTTCGCGTGCCTCCTCAACGCCCTGCTCTGCAACAG
GAGGCCGGCGAGGCCGGTCGACCGCACGCCGCGTGCAACGGCAACGCCAACGCCAACGCCCGCCACCG
CGGCGCCGGAGCCGGCGTCGGCGAGGTCGATCCTCTCGTCGCGCGCCTCGCGGAGCCGGAGGGAGTCC
GCGGCGGCGGGAGGGGTTCTGGCCCCGGCGAGGCGGGCGGTGCGGTTCTCGCCCGTGGCGACGGTAGT
GGGCGACGGGCACGGGCACGGCGCTGGATCGGTCGGGACGGCGACGACGGGGCTGCGGGCCAAGGAGT
CCGCGGCGGAGGCGGAGAGGAGGGTGGAGGAGCTGCTGCGCGCGCTCGGCGTCGCGGATGAGCGGGAG
AGGGCTAAGGAGAGCACCGAGTCCAGCTCCGACCTGTTCGAGCTCGACAGCCTGCCCGCATTCCAAGA
CAGAGGCACCGACCTGCCGCGTTCCAGAACCGTCGCCGACGGCGACGATGGCGCCGGGCTGCTGGCAC
GGCCGCGGCCCCGCGTGCAGTGA

>Zma_GRMZM2G110473
ATGGAGAGGTGGGCGCCAGCGCCACCGTCGGCGGCGCGGGAGAGGCCGAGGCGGCGACCTGGCCAACC
ATCCTTCTCGTCCACGCTGCTGGACGCCATCTGCGATTCCTTGGACGAGCAGGCCGGCGGCCACGGAG
CAACGGCGGAGCGTGCCACGGCACCGACGCCTCGGAGCGCCAAGGAGCAACACCAGGCGGCCCTACAT
TACTACTACTACAAGCCCTTCTTGGCCGCCAGCCACCGGGCGGCGCGCGCGGCCCCTTCGCCTGCGGA
CGACTGCTCCTCCGGCCGTGGCTACTTCTCGTCGTCCGAGGTCGAGTACTCCCTCCGCCGCCTCCGCC
CCATCCGAACCTCAGCTGGCGGCGTTGGGCCGGCCTCGGTGGCTCCCGTGGAGAAGCAGAAGCCGGCC
CCGCCAGGCACGGCGAAGAGGGCGCGGAAGCCGTCCGCCGCCCCCGCCAGCGGCGGCTGCCGCAGGCC
GGCCTCCCCCGGCGCGCGGCTCGCCAGCCTTCTCAACGCCATATTCTCCGGCAAGCGGAATTCTGCGC
GGCAGCACCCGGCTCCGGCGGACGAGGAGCCGGCGTGCTCGACGGCGCCATCCACCGCGCGCCCCTGC
CTCGCCAAGACACCGCCGTCCGCGAGGGCCCGGGCCAGGGCGACCCGGAACCGTAGCAGGACCGTGCG
GTTCTTGGACATCGAAGGCGAGGTGGCTGTGGCTGCGGCCGCCGCTGGTTGCAGGCGATTCCCGGTGG
TGGAAGTGGAGGACAGCGACGGCGGCGAGGAGAGCAGCGACGCGAGCTCCGACTTGTTTGAGCTAGAG
AACCTCGCAGCCCTTGCTCCCGCGAACGGCGGGCCCGGTTGCCGTAGGACGTGCGAGAACGAGCTTCC
GGTGTACGGGACGACTGGAGCTGGTCTTGGGAACGACAGTAGACTTGTCCGTCGTCGACGCCCGTTTG
GGTACGTCAGCCATGGTCGGAGTTGCAGAGGGTTGTTTGATTTCAAATAG

>Zma_GRMZM2G178852
ATGGAGAGGTGGGGGGACAAGGACAGGGGGGCGGCGGTTCCGGCGCCGGGGAGGTTGAGGCGGTACGC
CGACCAGCCGTCCTTCTCGTCCTCGCTCCTCGACGCCATATACAAGTCCATGGACGAGCCCGGCGACG
GGGCAACATCCGCCGCCGCCGCGGGAGCGACCAAGATGCAGAGCCACCAGGACCTGCACTACAGCTAC
TACTACAAGACGTCGCTGGCGGGGAGCTACCGCGGCAGCAGGGCTGCGGCGGCGGCGCACGCCGCCAC
CACCACGTCGAGCTCCTCCGAATGCTCGAGCTACGGTGGGTTCTCGTCGTCGGAGGCGGAGTCGTCGC
AGCACCGGCGGCTGCGGCCCATACGCACGAGCGTGGGCGCGGCCGCGTCGCCCGCGCCCGCGCCGGAG
AAGAAGAAGAAGGCCGGCGCCAACATCCGCGCCAAGCTGAGGGACCTCCGCAAGCCGGCGTCCCCCGG
CGCGCGGCTGGCGGGGTTCCTGAACACCATCTTCAGCGGCAGGCGCGCGCCGGCGACGCCGCCGTCGC
GGGGCGCGGAGTCCTCCGCGTGCTCCACGGCGTCCTCGTACTCGCGCTCCTGCCTCAGCAAGACGCCG
TCCACGCGGGGCCAGCCGAAGCGGACCGTGCGGTTCCTGGACAGCGACGACGGCGAGGCGGCGGCGGC
GGCGCCCGGCGGCGAGCGGCGGAGGGTGCAGGTCGGGGTGGCGGAGCTGGAGCGGATGCTGCTCCACC
GGATGGAGATGGACAGCGACGAGGACGACGAGGACGAGGAGGGCAGCGACGCCAGCTCCGACCTGTTC

GACCTCGAGAACTTCGCGGCCGGTGCCCCCGACGCCGCGGCGGCGTACAGGGACGAGCTGCCGGTGTA
CGAGACGACGAGGGTGGTGCTGGGACACCGCGCCATTGGCCACGGGAGGAGTGCCAGGGTGGTGTGA

>Zma_GRMZM2G007134
ATGGAGAGGTGGAGGGACAAGGACAAGGGAGCGGCGGCTCCGGCGCCAGGGAGGGCGCGGCGGTACGG
CGACCAGCCGTCCTTCTCGTCCACGCTCCTCAACGCCATATACAAGTCCATGGACGAGCCTGACGACG
GGGTAACATCGTCCTCCTCAACCGCCGCCAAGAAGCAGAACCAGGACCTGCGCCACAGCTGCTACTAC
TACAAGGCGTCGCTGGCGGCGGGGAGCTACCGCGGCAGCAGCAGGGCGGCGGCGCCCCGGGGGCCACA
GGCCGCCACCACGTCGAGCTCCTCCGATCAGTGCTCCAGCTACGGCGGGTTCTCGTCGTCGGAGGCGG
AGTCGTCGCAGCACCGGCGCCTGCGGCCCATACGCACGAGCCTCGCCACCACCGCGGTGGCCGTGGCC
GCGCCCGCACTCGCGCCGGAGAAGAAGAAGGCCGGCGTCAGCATCCGCGCCAGGCTGAGGGACCTCCG
CCGCAAGCCGGCGTCCCCCGGCCCCGGCGCGCGGCTGGCGGGGTTCCTCAACGCCATCTTCAGCGGCA
GGCGCGCGCCGCCGTCGGCGTCCTCCTGCTCGCGCTCCTGCCTCAGCGAGACGCCGTCCACGCGGGGC
CAGCCGAAGCGGACCGTGCGGTTCCTGGACAGCGACGGCGGCGGCGAGCGCCGGAGGACGGTGCCGGT
CGGGGTGGCGCCGGAGCTGGAGCAGATGCTGCTCCACCGGATGGAGGTGGACAGCGGCGACGACGACG
ACGACGAGAGCAGCGACGCCAGCTCCGATCTGTTCGACCTCGAGAATTTCGCGGCCGTTGACCCGGAC
GGCGGCGCCGCGTACAGTGACGAGCTGCCAGTGTACGAGACGACGAGGCTGGTGCTGGGCCACCGCGC
CATTGGCCACGGGTACGCGCGCACGGGAGGAGTACCCACCAGAGTGGTGTGA

>Spo_Spipo0G0184800
ATGGAAAGGTGGGAGAAGCTTCAACACCGAGGCGGCGCCGCAGGAACCGCCGCCGCCGGCAAGTATCA
CCGCTCCTTCTCCTCCAGTCTCCTCGACGCCATCTACCGCTCCATCGACGACGACGACACTGTCGGAG
GAAGAGGACTACCAGTCGCCGCCGCCGCCGCCGATACAAGGCGGCGAGAGCTCCACCGGGCGGCGGCG
CCGCCGCCGCCCTACCCCGCCGTTTGGAACCACCACCCCTGCCGCTGCAGGAACCATAGGGAGGAGCG
GTTCTTCCCCAGGTCGGCGGCGGCGCCGCCCTCCTCCACCTTGTGGAACCACCACCTCCACCGCTGCA
GGAAACAGAGGGAGGAGCGGTCCTTCCCCAAGTCGGCGGTGGCGCCGCCCTCCTCCGCCTCGTGGAAC
CACCACCACCACCGCTGCAGGAAACAGAGGGAGGAGCGGTTCTCCCCCAAGTCGGCGGCCGCGCCCGC
TCCATCCGCCGCTGCTCCTCCTCCGGCAACATCGCCGGAGAGCAGGAGTAGGAGGAGGGAGAGCTCGA
TCATGGAGATATTCCGGTCGTCGAAGGTGTACAGCGAGCTGAGGAAGGCGAAGACCCCGATTTCCCCC
GGCGGCCGGCTCACCAGCTTCCTCCACGGTCTCTTCTCCGCCGCCGCGACCCCCAGGAAGTCGAAGCC
CACCGGAGCGGCGGCGGTGGCGCCGCCGCCACCGCCGCCGCCTGCCTGCATGGTTCCCGCTCAGCGGG
GGGAAGAAAGGAAGCTCGCCGTGGAGAGGTCGGCGGTGGCCGAGGCGCGATTTCACGAGAGGAAGAGA
GCAGCGGAGGAGGGGGAGAAGGACGACGATGGAGATGACGACGGCGGCAGCTGCTCTAGCTCCGACCT
CTTCGAGCTGGAGAATCTGACGGCCATCTCCGCCGCCGGCGGCGGCGAGAAAAGGTTCATGGACGAGC
TCCCGGTGTTCGAGACGACCCGCTTGGAACCCCGTCGCCGTCCGCCACGCTGTTCCTAA

>Spo_Spipo0G0137500
ATGGAAAAGCTGCAGAGAACTTACCACCACCGCCGCAATGACTTCATCGCCACCGAAGCCAAGAGTCG
CCAACCCCACCACCACCCCTCCTTCTCCTCCTGTCTCCTCGACGCCATAGACCAGTCTATCGACAAGG
GCGGCGGCGCCGGAGGAGGAAGATGCGAGATCGAAGGCGCGGGTTCCAGCGGAGCAGCGTCGGCGCGG
CTCCGCTGCTCCACTCGTGTCTCCGCTGACGAGAAGAAGCGCGTCGACCAAAGAAGCTACTCCCGCCA
TGCCGCCCACGCCCGCGAACCGGAAGACCGGCGCTTCGCGGGGCGGGTCTCGGAGAAGGCGGCGGCGG
CGCCGCGCCACCGGGGAGCCACCGCGGAGTACGAGCAACGGCAGCCACACCGCAGCCGGGCGCGGGAG
AGAGATCGCTTCTGCGGTAATTCGACGTCTAGCTCGTCTGAGTCGAGCTATGGCGGTTTCTCCTCCTC
AGAGGCCGAGTCCGTCGTCCGCTCTGCACGTCACCTGCCGAAGCCCGTTCGAACCGGCGGCACACCGG
AGAGAAAGGCGGAGCAGAAGATGAAGGGCTCCATCCGGAGCAGGTTCCGTGCGTCCAAGGTCAACGGG
GAGATGAAGAATGGGAGGACCCCCGTGTCCCCCGGCGCCCGCCTCGCTGGCTTCCTCAACTCCCTCTT
CTCTACGGCCGCCCACCCCAAGAAGGCGAAGGTCATCTCCGCGGCTTCGAACTCCGCCGTCCGCAGCG
TCATCAGAGGAGGAGACGAGTCTGGTTGCTCCTCTGCTTCGTCCTTTTCCCGGTCCTGCCTCAGCAAA
CCGGCGTCGAACGGCGGCGGCAGCGGGGGCGGCGCCGCCGCCGCCGCCGCTACCAAGAGATCCGTCAG
GTTCATCGTCGAAGAGGAGTGCTACCCCGTATGCCACGAGTCGCACTATCTACCGCGTCTAACGGCCA
TCAGGGTGAGGGAGGAACCCCTGCTACCGCCGCCGCTGCCTTCTGATCTGGTGGCCGCCGCCGTTGCG
GAGGAAAGGAGGCGCGTGGCAGAGAAGGCGAGGAAACTCATCACCGCCTACGAGGAGAAGAAGAGGAC
CAGCGCGACCGCCTTAGCCGACGAGAAGGAGGAAGACGAAGACGACGACGCCGCGAGCTACTGCAGCT

CCGACCTATTCGAGCTGAAGGATTTGTCGGCGATCGGCGGCGGCGGCGGCGACGAACTGCCGGTGTAC
GAGACAACGCACTTCTCCGCCAATCACGCCATCTCCCGGGGCATCATCGTGTAA

>Spo_Spipo2G0082400
ATGAAGCAAGCCGACGAGCTCGACGTCTTCGAAGCGGCGGGTTACTATGCCGAGACAGCCGACGCCGC
GAGCGTCCAGGGAGGGTGGAACATGGCCGAGAAGGGCTGGAGAGGAGGGGAGAGGAGGACCGAAGGGC
CACCTCCTCTACAGAAGGTCAGAGACAAGAAGCCGAAGCAGCCCCTCTCGCCTGGTGGTAGGTTAGCC
AGCTTCTTGATCTCCTTCTTCCACCATGGCTCTTCCAGACAGAAGAAATCAGCGCTTTCCATTAGTGG
GGGAGCCAGTGTCTCCTTTAAACATGAAGACGAAGGGAGGTCAACAAGGAGGAGGAAGAGGAGAGCCA
GCGAGTCGTCCTTGTTCGTCTACTCTTCGCGCTCTCCATGCAAGGCCAACGTGGACAAGAAGACCCAT
CCTACAAGCTGGCGCCGAGGCATTTCGAAAGCTGCTTCAGGTCCAGCGAGAGAGGTCTTCCATGGAGG
AAGATCCGGCTATGGCGATCCTCTACGGGAGGGTGGCAGGAGGGTGTCCCTTAATGGGATCTTAGAGA
AGGACAGCAGATTCTACTGCGAGAGCTTGCTGCAGTTAGAGGAAAGGACTGTCTGGAGGAGGGGTGAG
GAGGAGGAGGAGGAGCAGGAGGAGGAGGGGGGAGAGGACGGGTGGGAGAGCGACTCGAGCTCCGACTT
GTTCGATCTGCCCAACTGCG
AACTGGTTCGCATCTAG

>Atr_scaffold00024.196
ATGGACAGGTGGTCTTACTCTGAAAAGCTAGAGAGAGAAAGTCGACATGCCCACCGGAACAGGAACCC
CTCCTTCTCCTCCGCCCTCTTGGACGCCATCTACAAATCCACAGAAGAAGACCAGCACCTCGTCTTCT
ACAAGAACGCCATTAAACCCCACATAAACCAGTCCTTCTTTGAGCCTGCGAATGTCCACCAATCTGCG
GTGAAGCGGAGCCACAAAGCCGAGAAAATCGTCGAAAAAAGAGTGGAGAAGACGAAGCCCCGCCGGAA
AGCCACCCAACTCGGAGTATTCGAGGCGGAGCACTACTACGAAACCCTCCTCTACGGAAGAGACCACC
ATAACAACATCTCCTCTTCTTCGGATACGAGTGATAGCGCGACAAACTATGGGTTCTCCTCCTCCGAG
GCCGAGTCAGCTTGGAGCCGCCGATCCGACCCATCGAGTCGGAAGTCCAGCAAGTCACCATCCACCCA
ATTCCTCGGCGTCTCAAAAGAAGAAGTCGCTGAGAAGAAGGCCATCAAGTCTCGGGCGTCGAAGCTCT
ATGGCGATTTCAAGAAGGTGAAGCAGCCGATTTCTCCCGGGAATCGGCTTGCGAGTTTCTTAAACTCG
CTGTTTTCCTCCGCCGAAAAGGCAAAGAAGGCGAAGCTCGCACGCGTGGAGACCGATTTCCGCGCTTG
CGAGAACAAGCCGCCACCTCTGCCTTCCTCGTCTACTTGCTCCTCCGCCTCCTCTTATTCGAGGTCGT
GTCTCAGTAAAACACCGTCGAGTAGAGGCAAGTCCGGGCCTAACGGCGTCAGGCGTTCCGTCCGTTTT
TACATGCCAGAGGAGACGGCGGAGCTCACGACATCAGCCACCGTTAGGAGGTCCGTCAAGTTTTATGT
GCCTGGTGAGGCGCCCGAGCTAACGGAGAGCGACGCCTTTAGGGTGCCGTTTCATGAGAAGCTGAAGC
TCCATTTCATGGAAAAGAGCCAAAAGGTGGAGGCGGGGAAGGAGCTGTTCAACGAGTATCAGAAGAAG
AGTGAGATCATAAAAGAGGTTATAATGAGGGATTTTGTGGATTCCAGAGAGAGAGAATTAGGGTTAGA
AGAGGAAGAAGATGATGATACAGAGAGCTGTTCGAGCTCTGATCTCTTCGAGCTCGAGAATTTGGCGG
CCGTTGGAATCGATCGATATCAGAAGGAGCTTCCAGTTTATGAGACGACTGATTTAGGAACGAACCAG
GCCATTGCAAAAGGATTCCTTCTCTAA

>Atr_scaffold00047.124
ATGTTGACAAATGGGGCAGACCATGGCAAGCCTCGTCGAAGAAACGATTCAGGCGAGCTCGATGTGTT
CGAAGCGACGAGGTATTTCAGCGAAGAGAGATCAAATCTCTCTACAAACTTTGCTAAGAATGGTTCAA
TCTCACAGAAAGCCGCTTTGAATAGAGAAGGATCAACATGTGGAGGAGAGAGAAGGATCCTAGACATG
CCCATGAACAAGCAAAGCTCTTCTCCTCAAAAGAAAATAAAGGAGAAGAGATCCAAACAACCTAGCTC
ACCAGGAAACAAATTAGCCAGCTTCTTGAACTCTCTCTTCACACAAAATTCTTCGAAGAAGAAATCGA
TGTTCAGTAATCAATCCATTAAAGATGATGATGAGAGCCCATTTGGCAGAAGAAGGAGAAGAAGTAGC
ATAAGCCTTCTCCAAACCGCGAGCTCAAACGACTCGACATATAATTCATCTTCAAGCTCAGATTTCAG
AACCCCCCCTCCTTTCACAGCAACACCAACAAGCTTAGGTAGGGAATTAAATAGCTTTTATGATCTTC
ATAGCATGGTTTCTCTCTCAAGCTGCACAAGAAACCACACCTCTGCCTCACTTGTTCCAACAAATGGA
GCTCTAGAGGGCAAGGTGACTAACAGTATGGGGTGGTCTGATCACAAGTCCAAGTATGAAAGTGAATT
TTTAGAGAGAGAAAGTAATTGGGGTTATGTGTATTCTAGTGAGAAGGGGATTAGAAGGATCTCTGAAA
TTGATGATGGAGGAGAGAGTGACTCAAGTTCGGATTTGTTTGAGCTTCAAAGCAATGACTTTGGTGTT
TACTCAAAGGGATTGCCTGTTTATGAGACCACCCACTTGGAGAGCATTCAGAGGGGAGCACCCATTGC
AAATGGCTCATTCTGA

>Bdi_Bradi4g31530

ATGGAGAAGGGCCGCCGCAGCCACGGCCACGGCAAGCGCCCGCCCCCGCTCGCCCCGCCGCGTCGGGA
GCGCGCTGCCGCCGCGTCCTTCTCGGCCTCGCTCCTCGACACCATCTACCGCTCTCTCGACGAGGGCA
GCGACGCCGACGCCACGGCGGCGGATGTCGCCGACACCCCGCGCCGGTCGGAGGAGAATGCGCCGGCG
CCCGCGCAGTTCTGGTGGGCCAAGGAGGCCGGCGGCAAGCCGAACATGCGCCGGCTCGAAACGGGCCC
GGCTCGCCGGCGGCACTCGGGGTACGCCTCGTCCACCGCCTCCTCGTCGGACTCCGCTTCCAGCAGCT
ACAGCTTCTCCTGCTCCTCCGCTTCGACCACCGACACCGAGTCCACGGCGCGCCGCCGACGCAGCCCG
CCTCCTCCGCCACGGCGCCAGCCGGAGGAAGTCCGTGCCGACGCCGCGGAAGCCGAACCCCCGTCTCC
GCCCAACAACAAAGCAAAGACGAAGAAAGGCAGGCCGTGTTTCCCTGGAGCAAGGCTTCGTCCCAGAG
ATGCGTCCGGCCCGTCTTCTCCTGCTGGGGGCCCACTCCCGCCGCCGTCGCCGGGGTCGTTCGCGTGC
GTCCTCCGGACCCTCTTCACCTCAGGCCGCCTCCCGAGGAAGCAGCCCAAGACTCCGATCTCACGGGG
CCCGCAAACCCCGCAGAGGACATCGCCGGAGCCAGCTGAGACGCCGCGCGCATCGGCGACGTCGTCGG
AAAGGAGGTCCGTGAGGTTCTGCTCGGATGCCGAGGCGTCGTCGGTGGTGCGGCGCAGGGTGGAGGAG
CTGGTGCGGAGCCTCGGGGAGCTGGAGGAGAACGACGAGGGGAGCGACTCCAGCTCCGATCTTTTCGA
GCTGGAAAGCCTCGGCGGGGCCAACGGTGACGAGCTGCCCGTGTACGGGACGACGAGCCTCGTGGCCA
ACCGGGCTATCGCGCACCAGGCGGTTTTCTAG

>Aco002602.1
ATGGCAGCCGCACCTATCTCGCCGCACCCTGTGCCATCGAGGGCGATGAGAGACGCATTTTTTAGGAC
GAATTTGGATGTTCCTGCTGCAAATAGTTTGGCACCCTCGACGATCGCCCTTGCCGTCGCCATCCATG
TTCATACGCTCTATTGTGACCTCATCTCTGCAATCTGTGTCATATCCGAATCAGTGTCGGCCGATGTG
TGGTTTTTTTGCCTTGGTTTTGGTGATGGGATACGCGACTGGCGAGGGCGGCCTTGTCGCCGCTGCCC
CCCGGCAGCGCACCCGGCGATTACGCCTCCTTCTCCGCCTCCCTCCTCGACGCCATCTAACGCTACCC
TCGACACGCCCGAGGACTCACAACAGAACCCAACCCGACCCGGCGCCACCCCAGATGAAGCCCCCTGC
CCGATCTGGTGGGTCCCCGACGCTGCCGCTGCTGCGAACACGGCGCACCGAGAGCCCCGACCCCGGCC
GCCGTCCTGGGCGATACGGCATTCGCCCGCCCCACTCCTTCACCCTCCCCCTCCCCCTCCGATCGAAA
CCAAGAGCGCCGCGCCGAAGCCGCGCTCGATCCGGAGCCGGCTCCGAGAGCTGAGGAAGGGGCGGGGC
TCGTCGGCCTCGTCGCCCCTCGCCTCCCTCCTCAAAGTGCTATTCACCTCCGCGGGGCACGTAAACAA
GGCCAAAATCCCGGCGCCGGAGCCGGAGCCGGAGCCGGAGCCGGCGTGCTCGTCGGCGCGGTCGTGCC
TCTCGGCTCGGCGGGGATCGGCGGCGGTGACGCCGCCGAAGAGGTCGGTGAGGTTCCACCCGGTGAGC
GTGATCGTGGACGAGGACTCGCGGCCGTGCGGGCACCAGCGCCTCCGCCGCGCGCCGGAGGAGAGCAG
CGACTCGAGCTCGGATCTGTTCGAGCTCGAGAATTTAACGTCGTCCGCCGGTCTACGGGACGAGCCGC
CGGTCTACGGGACGACGACGCGCTTCGCCGTCTCGCTGCCCGCCGCGCCACCGTCCACCGCAATTCAA
ACAGCACAGTTTCAAATTTCTTAA

>Aco030183.1
ATGGATTCAAGAGTAGAGGAGACGTTAAAACAAAACTTGATTAGACTACACATCACGGATATTAATAC
GGGACACAAGGTACGACACGACTTAAACACGACGACTTGGGACCTTCTAAAAACGAATTTGTCCCCTC
ATCTACCCCCGCTCTCTCTTTCTCAAGTATTCCTCGGTCGTTTGCACCCCTCATCTCCTCCCGTCGCT
CTCGATTTCTATCTCTCTCTCTCTCTCTTAGCATCCGCGGGTGAGGATCGGCATACTTGCGGACATCG
CCGAGTGCACAACGGGGATCGAGCGGCGGTGGAGGCGAGGAGGGTGGAGAGGCTGCTCCAAGGGTTTG
AGGAGGAGAAAGAGGATCGAAGGGGGAGCGATGCGAGCTCGGATCTCTTCGAGTTGGAAACCCTAATC
GCCATTGGAGGAGTCGGAGGAGGAGGATTTGGGGACGAGCTCTCGGTGTACGAGACCACAACGACAAG
GCGCAGTACGAATGATGCCATCGCCCGCCATGGTCTCATTTTATAA

>Aco004841.1
ATGAGCAGCTTCGTCTCCCGGAAGCTCTCTCCGCGCCGGAACAGCTCCTTCGAGCTCGATGTCTTCGA
CGCCAAGCGCTACTTCTCCACCGCCATCGACGACGATGCCAGCACCTTCGTCGGCTATCCTGGACGCG
CGAGACACCAAAAAGCCGCCGCCGCCGAAAAGAAGAGCTTCGTCACGAGCAGTAGCGCTCCCGTGAAA
GGTAAAGGGAGTTGTGATTATAACAAGTGCACGACTAAGCAGCCCACCTCGCCCGGGCTTAGGCTGGC
TAGCTTTATCAACTCTCTAATCCAACAAGCGGCTTCAAAGAGAAAGCCGAAGCCCTCGGCCCCGAACA
CGCCAACGACCGCTTCTCAGAAAAGCAGAAGCAACGAAGCCGTGTACTCCTGTGTGAGCAGTGGCTTT
ACAGCACCTTGTTTACAGTTTAACACGCGGTCGTACGTTCACAAGGAGAAGAGCAGACTCCGTAGCGA
CACGAACCAATATCGTAAGGAGCCAGAAGGAGCAGCATATCGGCCACACGCCAAAAATGGCATATGGC
CGTGGAAGGAGGATAAGTATGTGCCGAGGGGTGATCACGGCCTTTATGGAAATGTGTATGATGGTGAT
CAAAGTGTGTGCGGCGGGGAGAGGAGATGGGTGTTGAGCAATATTCATAAATATCGCGATGATCATCA

TGGGTATCTGATGAAGAGAGATTGGAGTTGGGTTGGCGAGCCTACGCTGCGGCAAAGAAAGAGCTTTA
AGAGATCAACAGAAGAGGTAGAAGAATACTGTGGTGAGGATTGGGAGAGTGACTCGAGCTCGGATCTA
TTTGAGCTCCAGATTGATAACTCCGATCTGCATCGACACGGTGCCTCCGGTGGCTTACCGGTGCTCGG
GACGACGAATATGAAGGCTCTTAAGAGAGGAACCGCCATTGCCACTGTTGCATCCTAA

>Aco009471.1
ATGGAGGGGTGGGGGAAGCCGCAGTTGCAACGGCGTAACGGGCACCGTTACGGTAACGGTAACGGTAA
CGGTAGCGGTAGCGGCTACGGTTGCGACAACCCTTCGTTCTCGTCGACGCTACTCGACGCGATCTACC
GCTCCATCGACGAGAGCGATGGAAGAGGGGAGAAGCATCGCGGCGGCGGCGGCGGCGGCGGCGCAGCA
ACGCACCGGCCGGTTCCCGTCGCCGCTCAGAAGAAGAGCGGGAGCTACGGGAGCGGCGCGGAGGCGAG
CGACGGGGCGGGGACGCGGCTCAAGCCGGGGCCCTCGACGTCGAGCTCCTCCGAGTGCTCCAGCTACG
GCGGGTTCTCGTCCTCCGAGGCCGAGTCGGGGCCCGGCTCGGCCCGGCTCAGGCCGATCCGGACTGGG
GTCCGGCCCGAACAGGCCCCGGCGCCGCCAATCCGGTCCCCGCGCCGCTCGGCCAAGCCCCCGGCGTC
GCCCGGGGCGAGGATCGCGAGCTTCGTCGCCTCCCTCTTCGCCGCCGCGGCGCGGAGCCCGCGTAGGC
CGAAGGCGGCAGCGGCGGCGGCGGCGCCGCCTCCGCCGCAGCCGCGGTCGTGCCTCAGCAGGACGCCG
TCGACGAGGGATCGCCCTCGTCCCCGCGCCCCGCCGCCGCGTGGGGAGGAGGGAGCGGGGAAGAGATC
GGTGAGGTTCCACGCGGCGAGCGCGGCCGCGGGCGAGGATCGGCGTACCTGTGGACATCGCCGGGTGC
ACGACGGGGATCGTGCGGCGGCGGAGGCGAGGAGGGTGGAGGGGCTCCTCCGAGGGTTTGAGGAGGAG
GAAGAGGATCGAAGGGTTGCGGAGGAGGGGGACTTCATGGATTGGAGCTTCTTGGAGGCCTTGTCGAA
GGGGGAGCGACGCGAGCTCGGATCTCTTCGAGTTGGAAACCCTAACCGCCATTGGAGGAGGAGGAGGA
GGAGGAGGGTTTGGGGACGAGCTCCCTGTGTACGAGACCACGACCACGAGGCGCGGTACGAATCGCGC
CTTCGCCCGCCACGGTCTCATTTTATAATAATTTAA

>Osa_Os10g25810
ATGCGCGACATGGAGATGAGGTGGGCGGCGCCGGCGCCGGCGGCGAGGGGGAGGGGGAGGGCGAGGCG
GCGGGCGCCTGACCAGCCGTCGTTCTCGTCGACGCTGCTCGACGCCATCTGCGACTCCATGGACGAGG
GCGGCGAGGACGGCCGGACAAGAAACGCGGCGAGTGCGGCGGCCAAGAAGAGGCAGGAGGCGGCGAAC
AGCTACCACTACTACTACTGCTACAAGCCGTCGCTGGCCGCCAGCTACAGGGCGGCGCCGGCGCTGGG
TTCCACGGCGGATTGCCCCGGGAGGGGCTACTTCTCGTCGTCCGAGGTGGAGTACTCGCTTCGCCGGC
TCCGCCCCATCCGCACCTCCGCCGCCGGTGGCGCGGGAGACGGCGCGGCGGTCGCGCGGAAGCAGCGG
CATGAGCAGCCGGATGTGGAGAAGACGGCGAAGACGAAGCCGGGCTCCGCCTCGGCCCGCGCGTGCCG
CAGGCCGGCGTCGCCCGGCGCGCGGCTCGCCAGCTTGCTCAACTCCATCTTCTCCGGCAAGCGTCCAT
CCGCGCAGCGCCCGGCGTGTTCGCCGGACTACCCGGAGCCCGCGTGCTCGACGGCGCCACCGTCGTCG
TCGTCGTCGTACGCGCGCCGCCCCTGCCACGCCAAGACGCCGCGCACCCCCCCCACCACCACCACCAC
GGCGAGGGCGCGGCCGAGCCGGAGCAGGACCGTCCGGTTCCTGGACATCGACGGCAAGGTCGCGGTGG
CCGCGGCCGTCGCCGGCTGCCGCCGAATTCCGGTCATGGAGGTGGAGGCCGACACCGACGACGGAGGT
GAGGAGAGCAGCGACGCGAGCTCGGACCTGTTCGAGCTCGACAGCCTCGCGGCCATTGCTCCGGCGGG
TGGTCGCGACGGCTCCTACGGGGACGAGCTGCCGGTGTACGGGACCACCGGAGTTGGGATCCGCCGCG
ACATTGGCCGCCGCCGTCCGTACGGCCATGCTCCTTGTCGGAGCTGGAGTAGGGCTGTCTAG

>Osa_Os03g07920
ATGGAGAGGTGGGCGGCGCCCAAGGTGACGGCTGGTTCGGCGAGGCGGTACGTCGCCGACCAGCCGTC
TTTCTCATCCACGCTGCTCGACGCGATATACAAGTCGATGGACGAGCAGCCGGGTCACGGCGGCGGCG
CCACCGGGGTGGAGGCGGTGGCTGCGGCGGCCAAGAAGCAGCACGAGGCGGCCCTGCACTATGGGAAC
TACTACAAGCCGTCGCTCGCGGGGAGCTACCGGGCGCGCGCGCCGGGTCCGCACGCCACGACGTCGAG
CTCGTCGGAGTGTTCTAGCTACGGCGGGTTCTCGTCGTCCGAGGCGGAGTCGTCGCACCACCGACGCC
TCCGCCCCATCCGCACGACTGTCCCCGGTGGTGCGCCGGGGCCCGCGCCGGAGAAGAAGGCCAAGAAG
CCCGGGGCCTCCATACGCGCCAAGCTCAGGGACCTCCGCAAGCCGGCTTCCCCCGGCGCGCGCCTCGC
GGGCTTCCTCAACTCCATCTTCGCCGGCAAGCGCGCGCCGGCCACGCCGCCCTCGGCGACGGCCGGGG
CGGAGTCCGCGTGCTCGACGGCGTCGTCCTACTCCCGCTCCTGCCTGAGCAAGACGCCGTCGACGCGC
GGGCAGGCGAAGCGGACCGTGCGGTTCTTGGACAGCGACACGGAGTCCCTGGCGTCGTCGACGGTGGT
CGACCGCCGCAGGGTGCCCGTGGAGGCGGTGCAGCAGATGCTGCTCCAGCGGATGGAGATGGAGAGCG
ACGAGGACGACGACGAGAGCAGCGACGCGAGCTCCGACCTGTTCGAGCTCGAGAACTTCGCCGCCATT
GCTCCCGCCGGGGCCGCGTACCGGGACGAGCTGCCGGTGTACGAGACGACCAGAGTGGCGCTGAACCG
CGCCATTGGCCATGGGTATGGCCATGGACGGAGCGCCAGAGTTGTCTGA

>Osa_Os02g53660
ATGTCTCCGCACCACAGCGGCCGTCTCGCGCCGCCGCGCCGCAACCGGGAGAACCCGTCCTTCTCCGC
CGCGCTGCTCGACGCCATCTACCACTCCCTCGACGCCGATGGCAGCTTGCCGGCGTCCCCCGCCGACG
CCGAGGGGTCACCGGTGCCCGGACGCCGCCGCCGTCCTTCGCAGTGCAACAACCTGTCGCCGTCTGCT
TCTTCCGTGCGGTCGCCGCGGCTGCAGAAGACGCCGCGGCCGTGTCGCGTGCGGCCTGACCCACAGCC
TTCGCTTCTCTTGCCGCCGCCGCAGCCGCCGCCGCCGATGCCGGAGTCAACAGGAGACGTCGCGGAGA
AGAAGAGGGGCAGAAGGAAGAACAAGAATGGTGCCAAGTCGGCGCCATTCGCGTGCCTTCTGAATGCT
CTGCTTTGCAACAGGAGGTCAGCCAGGTCGGCCGAGCCGACGACGCCGCGGGCTCTGGCGGTGGCGCC
GGCGGCAGTGGCAGTCACCGCCGCTGAGCCGGCGTCCGCGAGGTCGATCCTGTCGTCGCGCGCTTCCC
GGCGACAGCCCGCGGCGACGGGTGGGATCCTGACTCCGGCGAGGCGGGCAGTGCGGTTCTCGCCGGTG
GCGGTCGTGGTGGACGACGGCGAGCACGGGTGCCGGGACGCCGGGGTGGCGAGGCTGCGCGGCGCGGA
GAGGGAGGTGGCCGCGGCGCAGGAGTCGGCGGCGGAGGCGGAGAGGAGGGTGGAAGAGCTGCTGCGCG
CGCTCGGCGTGGCGGAGGAGAGCGAGAGGGCCAAGGAGAGCAGCGAGTCCAGCTCCGACCTGTTCGAG
CTCGAGAGCTTGCCGGCGTTCGACGACGCCGAGCTGCCGCGGCCGCGAGCTGCCGCCGGACTCGTGCT
GGCACGTCCGCGACCTCGCGTTTGTTGA

>Osa_Os09g27160
ATGGGCCACGTCACTGTTCACTCCTGGTCCCATTCCATAAGTTTAAGGGTGTGTTTAGTTCAGCGGCG
GCAATGCCACGGCAAGAACGCGCCGCCAACGCTGGCGCCGCCGCGGAGGGCGCGCGGCGGCGCGGGCG
GGTCGTTCTCGGCGTCGCTGCTCGACGCGATCTACCGTTCTCTGGACGAGGGAGGGGGTGGCGATGGC
GCGGGTGCTGTCGTCGATGACGCGCGGCGGTCGGAGGCGGAGGAGATGAAGGCAGCGGCAGCGGCAGC
CGTGCCGCCGCAGTTCTGGTGGGCCAAGTCGAAGCAGGCGGCGGGCGCCGCGGGCAGGAGCCGTCGCG
AGTCGGTGGCTCGCCCGCGGCACTCGGGGTACGCGTCGTCCACCGCCTCGTCGTCGGACGCGTCGTCG
TCCAGCTACAGCAGCTTCACCTGCTCGTCCGCGTCGACCACCGACACGGAGTCGACCACGCACCGGAG
ACGCCACAGCCAGCCTCCGCCGCAGCAGCCGGAGGACGTCGACGCCGCCGCCGCCGCCGCCGCTGCCG
CGCCACCGAACAGCAAACCGAAGAAGAAGAAGAAGAAGAGCCGGCCGTGTTTCCCT
GGAGCAAGGCTGCGGCCAAGAGGCACAGTGCCGCCGCCACCGCCGTCGTCATCCGGCCCGTCGCCGGC
CACGTTCGCGTGCGTCGTCAAGGCGCTGTTCTCCTCGTCGCGCCTCCCAAGAAAGCCCAAGGCTCCAA
CGGCCGTCCCTCTGCCGCCGGCATCGCCGCCGGTTCCGCAGCCACCGTGCATGTCGGCGGCGGCGACG
ACGACGTCGAACACCAAGGCGTCGGAGCGGAGGTCCGTACGCTTCTGCCCCGGCGCCGAGACGTCGGT
GGTGCGGCGCAGGGTGGAGGAGCTGGTGCGGAGCCTCGCCGATGTGGAGGAGGACGAGGACGGCAGCG
ACGCGAGCTCCGATCTCTTCGAGCTGGAGAGCCTCCGCGGCGCCGACGGCGACGAGCTGCCGGTGTAC
GGCACGACGAGCCTCGCGACCAACCGCGCCATTATATTGCGCAGAGAGCAGCTTGCTAGTTCATAA

>Zmar_Zosma188g00060
ATGGAGAGAAACAACACTCACCGCCGGCTAACGCCATCTTTCTCGTCCATTCTTCTCCACGCAATCGA
CCGTTCCATTGACGAAGCCGATGGTATCGGAAACACCCGAGTGCAAACCCCATGCACATGCAATAGAA
GTAGAAGTAACAGTACCACTCCCGCTGCTGCTACTTATAATCAGCAGCAGCAGCAGTACGGTGGTACT
CGCAACCCAAAACTGCGTGTCAAGGAAAAAAGATTTTCCTCGTCAGAAGCGGAGTCGAGTGTCGGGGG
AAGTCTTGTCGACGTTTCACCAACAAACAAGCAGAAGAATAAACAGAAGAAGAAGAAGCAGGATTCGA
AATCGCTCGGGAATCGATTTCGGGCATCGAAGTTGTACGCTGAGATAAAGAAAGCAAAACCACCAGTG
TCTCCCGGAAGCCGTCTCGCGAGCTTCGTCAACCAACTCTTCACCTCCACTGGGAAGACAACGACAAC
GAATCAAAACCTGATCGATCGGTATCGCTCTTACTCCACTACTTCTTCTTCTTCGTATCTAAACAAAG
CTCTTTCGTCGAAATCGCTGAACAATTCGAAATTGACGGTGAGGGTGGATGAAGAAATCGTTATGGAC
GACGACGAGACGAGAAGCTATTGCTCGAGTTCAGATCTGTTCGAACTCGAGAATCTGACGGGTGTGGG
TGCGTTGGATAACTACACGAACGAACTCCCCGTGTACGAGACTACTCATCTCCAAACTAATCGGGTCA
TCGCTCGAAGGCTCATCGTTTAA

>Zmar_Zosma87g00320
ATGGATAAAAGTGGCACCTGTTACTGCTGTTACTGCCGTCGCCGAGAAGCTGCAGCCAACCCCTCCTT
CTCCTCCAGTTTACTTGACGCAATCTATCGATCTATTGACGAAGGAGATGACGGCAACAACGATCGGC
CGACAAGACCTAGATATTGTGGAATCCAAAAAGGAAACATTTCGTTAAACATTACTGCAGTCGAGAAC
AAAAAGAAGCAAGTCACCACTGTGGTGACGGAAAAAGCTAACGCTCGGCAACTCACCACTGTCAAGAC
CTACGATCGTCGCGGATGCAAGCCAAATCAAGACAAGGAAGTCAAGGAGAGACGTTTTATCAACTCTG
CTTCGAGCTCATCCGAGTCGAGTTGTGGTGGATTCTCCTCTTCCGAACCGGAAACCACTCGACTCAAA
CCATTTGCTCGTCCGGTTCTTTCGTCTTTCGAGCAGGTGAAACAAAAGATGGAGAAGCATCCGAATTC

ATTAGGGAATCGATTCCGCTCTTCGAAGATCTACGGAGAACTTAAGAAAGCGAAGTTACCTGTCTCTC
CAGGTGGCCGGCTGTCAAGTTTCATCAACCAGCTATTCAACTCTGCTTCTCATCTGAAGAAAGTAAAA
ACCTCGTTGCCCGTAGGAAACACCAAAGATGGACTAGGAGATGACTCTACATGCTCCTCGGCATCCTC
TTACTCAAGATCGTGTCTCCGAAAAAATCCGTCGACGAGATCGAAAACCGCAAACAGTGGGAAAAGAT
CTGTCAGATTCTACCCCGTCAGCGTGATTGACAGCGACGATCTCTGTCCATCTATCAACAAGTGCCTA
GACAAGGACTGCAATTGTGGATACAAAGTAACCGATGTTTCGCCAAGAATGCATCCACTGCCGCCATT
GCGGGTAAAAGATCCTACAGTGGAGGACATGAAGGCAAGGGATATGATCGAAAGAGCCTACGCTAATG
GGAAGAGATCGTTGTTTCGATTCGATGACGATGATGATGACTTCGATGATGCAGCTAGTTATTCAAGT
TCAGATTTGTTCGAACTCGATAATCTGGTAACGATCGGCGACGGATGTGAAGAAGCTCCCCGCGACAG
GGAGATGTACTCGAACGAACTTCCTGTGTACGAGACCACTCTCTTCCGCAATAATCGCGCTATCCCCA
AACAAGTTGTAGTGTAA

>Sbi_Sobic.004G316600
ATGCCTCCACACGACGACGACGGCGCCCGCGTCCCGCCGCCCCGGCCTAGCCGGGGCCACCAGCCGTC
CTTCTCCGCGGCGCTCCTCGACGCCATCTACCACTCCCTCGAGGCCGACGGCGAGGCGCGCTTGTCCA
CGGAGGCGCGCCGGACCCGGACGCCGGCGTCGTCGTCGTCCCCGGCGCGGCGGACGCCGCTTCCGAGC
CGACGTCGCCCGACGCCCGAGCAGTCGCCGTCCCGTTCCTCGGTCCGGTCACCGCGGCTGCAGAAGAC
GCCGCGGCCGTGCCGCGTCCGTCCAGACCCGCAGCCCATCCTCCTGCCGCCTCCGCTCCCTCCGCCAC
ACCCACAAGAGTCGTCGACCGGGGACCGCCGGGCTGCGGACGCGGAGAAGAAGAGGGGCCGCAGGAAG
AAGAGCAAGGGGACGGCGAAGGCGGCACCGTTCGCGTGCCTCCTGAACGCTCTCCTCTGCAACAGGAG
GCCGGCCAGGGCCAGGTCGGTCGACCACACGCCGCGGGCAACGGCCGCGGCGTCGGAGCCGGCGTCGG
CGAGGTCGATCCTCTCGTCGCGCGCCTCCCGGATGGAGTCCGCGGCGGCGGGAGGGATCCTGACCCCG
GCGAGGCGGGCGGTGCGGTTCTCGCCCGTAGCGACGGTGGTGGGCGACGACCACGGGCACTTGACGGC
GCCGACGGGGCTGCGGGACACGGGGGCGGAGATGGCGCGGGCCAACAAGGAGTCCGCGGCGGAGGCGG
AGAGGAAGGTGGAGGAGCTGCTGCGCGCGCTAGGCGTGGCGGACGAGCGGGACAGGGCCAAGGAGAGC
AGCGAGTCCAGCTCCGACCTGTTTGAGCTCGAGAGCCTCCCGGCGTTTGAAGACAGAGACACCGAGAT
GCGGCGTTCCAGAACTGCCGCCGGCGACGGCGCCGGGCTGCTGGCACGGCCGCGTCCCCGCGTAGCTG
TGTAA

>Sbi_Sobic.001G337500
ATGATGATGCAGAGCCGGCAGCGGGAGCACGGCAGCTCCGGCGAGCTCGACGTGTTCGGGGCGACGAG
TTACTTCGCCGGCCTGCCGCCGCCGGCGCCTGACGACGCCGACTGTCGTCGTCCCAGCAGTGCAACGA
CAGAGCCGTCTGCCGACCGGCTGTACTTCCAAGCTACTAAGGTGGTGGTGCATCCTGACACGAGGACA
AAGACGATGGAGGACAACAGTTTCCGCGGTCCACATCAGCAGCAGCAGCAGCCTGGGCTGCATGGTCA
CGCCGACCGCCACGACACCAATAAGCAGCTGCAGGTAGTGGCCGCCAAGCGGCAGCAGCCTCCTTACT
CAGGTAAGAGCAAGCTCGCCGCCCTCTTGAGCTTCATGGTGTCGCCGTCGCCGTCGCCGAGAGCTAGC
TTTCGCAAGGAGATCAACAAGCAGGAGGCACCATCGACGACGACGACGACTAGGCTGCTGCGGCAGGC
GGAGGCGGCACCAGCAGCAGGCGGCGAGAGAGAGACTGCTGCTGCTAATAATACTTACAAGGCGGCGG
CGAACTCCCCGTCGTCGTCCTCGCGCGAGCGCAGCAGCAGCATGCAGCTGCACGGTCTGTTCGGCGCG
CACGACGACGACGACGACGACGAGGAGCTCGACCTGGGCGTGGCGACGGGGGACAGGAGGCTGCAGGG
CATCACGGTCGTGAGGGGCATCGGCGGCGGCGAGGAGAGGTGGGTGGTGAGGTGCTGCGTGCCCGTGC
CCGGCGGCGGCGCTTGGGACGACGAGGAGCACCGTGAGAAGATGCTGCTCGATGCAGCAGAATCGGCG
TCAAGCGAGCAGAACGTCAAGGACGAGCTGCTGGTTGAGGTTGAGCAATTGCAAGGAGATGATGATGA
TGATGGTAATGTTGTTGATCCTGCTCCTAGCAGCTGGGACAGCGATTCGAGCTCTGATCTGTTCGATT
TGGATCTTGAGTACAGATGA

>Sbi_Sobic.001G485400
ATGGAGAGGTGGGGGGACAAGGACAAGCGGGCGGCGGGTGCGGCGCCGGGGAGGGCGAGGCGGTACGC
CGACCAGCCGTCCTTCTCGTCCACGCTGCTCGACGCCATATACAAGTCCATGGACGAGCCCGACGATG
GGGTGACATCATCCGGCGCCGCCACCGCGGCAGCGACCAAGAAGCAGAACCATGACCTGCACTACAGC
TACTACTACAAGGCGTCGCTGGCGGGGAGCTACCGCGGCAGCAGCAGGGCGGCGGCCCCTGGGCCGCA
CGCCGCCACCACGTCGAGCTCCTCCGAGTGCTCCAGCTACGGTGGGTTCTCGTCGTCCGAGGCGGAGT
CGTCGCAGCACCGGCGCCTGCGGCCCATACGCACGAGCGCCGCCGCCGGGGCGGCGGCCACCGCGCCC
GCGCCCGCGCTGGCGCCGGAGCAGAAGAAGAAGGCCGCCAAGAAGGCCGGCGCCAACATCCGCGCCAA
GCTGAGGGAGCTCCGCAAGCCGGCGTCTCCCGGCGCTTCCCCAGGCGCGCGGCTGGCGGGGTTCCTCA
ACGCCATCTTCAACGGCAGGCGCGCGCCGCAGACGCCGCCGTCGGCGTCGGCGTCGCGGGGCGCGGCG

GCGGAGTCCGCGTGCTCCACGGCGTCGTCCTACTCGCGCTCCTGCCTCAGCAAGACGCCGTCCACGCG
GGGCCAGCCGAAGCGGACCGTGCGGTTCCTGGACAGCGACGACGGCGGCGAGGCGGCGGCGCCCGCCC
CGGGCGTCGAGCGCCGGAGGGTGCAGGTCGGGGTGGCGGAGCTGGAGCAGATGCTGCTCCACCGGATG
GAGATGGACAGCGACGAGGAGGACGACGAGGAGGACGAGGAGAGCAGCGACGCCAGCTCCGACCTGTT
CGACCTCGAGAATTTCGCGGCCGTTGCCCCCGACGCCGGCGCCGCCGCCGCGGCGTACAGGGACGAGC
TGCCAGTGTACGAGACGACGAGGGTGGTGCTGGGCCACCGCGCCATTGGCCACGGGTACGCGCACGGG
AGGAGTACCAGAGTCGTGTGA

>Pda_XP_008797636.1
ATGGAGAGGTGGGCGAAGCCGCCGGTGCGGCGCGGCAACGATAACCCGTCCTTCTCCTCCACTCTCCT
CGACGCCATCTACCGCTCCATCGACGAGGCCGACGGCGGAGCCGCGAGGGAGCACTTCACCGGCGGAG
CACCCGACCGCCGAGACGCCTCCGTTCCCGCGGCAGCAAAGAAGCAGCAGAGCTTTTTCACCGACGAG
TGGCCGGCGGAAGGGAGGAGGACCGCGGTGGTGAGCGAAAAGGCGGTGACACGACTTCGCAAGCCCGA
AAACCGCCCCAGGTTCCCGGTGAACTCGACCTCCAGCTCCTCCGACTGCTCCAGTTACGGCGGGTTCT
CGTCCTCCGAGGCAGAGTCGGTGCCCCGCCCTTCCGGGCTCCGGCCGATCCGGACCGGCGGTCTTCTT
TACCGGTCTGAGAGGGCCCGGTCGAACCCCGCGCCTCCCCAGCCGCCAGCTGTAGGGTCGCCCTCGGC
TCACCACCACCAGGAGAAGAAGAAGTCGGGCTCGATCCGGAGCAAGTTCCGGGATCTAAGGAAGGCCA
AGGCGCCGGCATCCCCTGGAGCCCGCCTCGCCAGCTTCCTCAACTCCCTCTTCACCGCCAACGGCAAC
TCGAGGAAGTCCAAATCCGCCGCTGCCGCTTACGGCGGCAGCGGCGTGAGAGGAGGGGAGGAGTCGGC
GTGTTCGACGGCAACATCGTACTCGCGGTCGTGCCTGAGCAAGACGCCGTCGTCGAAGGGGCGGCCGG
CGGTGGCGGCGGCGGCCGGCCAGGGTGTGAAGAGGTCTGTGAGGTTCTACCCGGTGAGCGTGATCGTG
GACGAGGACTGCCGGCCGTGCGGGCAGAAGTGCCTGTACGAAGGGGATTCGGCGGCGGTGGCGCGGCG
GCCGCCTGAGGCAGCGACGATTGCGAAGAAGAGGGTGGAGGAGCTTCTCCGAGGGTTTGAGGAAGAGG
AGGAGGACGAGGAGGCGAGCGATTCGAGCTCAGATCTGTTCGAGTTGGAGAACCTGACGGCGATCGGA
GGGCGAGGATACAGGGATGAACTTCCGGTGTACGAGACCACTCATCTTGGCACTAATCGCGCCATTTC
CCACGGTTTCTTCTTGTAA

>Pda_XP_008787849.1
ATGGAGAGGGGGCCGAAGCCGGCACTGCGGTGCGGTAACGCTCACCCGTCGTTCTCCTCGACCCTCCT
CGACGTCATCTACCGGTCCATCGACGAGACCGACGGCGGAGCAGTAAGGGAGCACCGCAGCGGCGGAG
CGCCAGACCGCCTCTACGACCGTGGCCCGGCCGCCGCTAAGAAGCAGCAGAGCTCCGTAGCCGACCGG
TGGCCAGCGGAGAGGAGAAGCACCGCAGCGGTGACCCGACTTCGCAAGCCCGACAACCGCCGCGGTTT
CTTGGTGAACTCGACTTCCAGCTCCTCCGATTGCTCCAGTTACGGCGGGTTCTCGTCCTCCGATGCCG
AGTCGGTGTCCCGCCCGCCCGGGCTTCGGCCGATCCGGACCGGCAGTTTTCTTTACCGGTCCGAGGAG
GCCAGGTCGAGACCCCCGGCTCCGCCGCCGCCGGTGGCCGCGTCGCCACCGGCGCACCCCCACCAGGA
CAAGAAGAAGTCGGGCTCGATCCGGAGCAAGTTCCGGGATCTGAGGAAGTCCAAGTCTCCGGCGTCGC
CGGGGGCCCGCCTCGCCAGCTTCCTCAACTCCCTCTTCACCGCGGCTGGGAGCCCAAGGAAGTCCAAA
TCCGCCACCGCCGCCGCCGCCGCCGCCGCCGGGCCCGCCTCTGGCGGTGGCGGCGGAACGGGAGGGGA
GGAGTCGGCGTGTTCGACGGCGTCCTCGTACTCACGGTCGTGCCTGAGCAAGACGCCATCGTCGAAGG
GGCTGCCGGCGTCGGTGGCCGGGGAGGCGGTGAAAAGGTCGGTGAGGTTCTACCCGGTGAGCGTGATC
GTGGACGAGGACTGCCGACCGTGCGGCCAGAAGTGCCTCTACGACGGGGATCCGGCGGCGGGGGCGAC
CCGGCGGCCGCCGCCGCCGGCAGCGGTGGCGAAGAAGAGGGTGGCGGAGCTTCTCCGAGGATTTGAGG
ACGAGGAGGACGACGAAGAGGGGAGCGATTCGAGCTCGGATCTGTTTGAGTTGGAGAATCTGACGGCG
ATCGGTGGGCGAGGATACAGGGATGAGCTTCCGGTGTACGAGACCACTCACTTTGGCACCAATCGCGC
CCTTTCCCACGGTCTCATCCTGTAA

>Pda_XP_008784466.1
ATGCGGTCCGGTCCAGAGGTCCCACCGGAGAAGAAGAAGGGCGGCTCGATCCGGAGCCGGTTGCGCGA
CCTGACGAAGGGCCGGGCCCCGACGTCGCCGGGGGCGCGGCTGGCGAGCTTCCTCAACACGCTTTTCG
CCGCCGTCGGGAGCCCCAAGAAGCCCAAGATCGCGGCCCCGCCACCGCTGGAGTCCGCATGCTCGTCG
GCGTCGTCCTACTCGCGTTCGTGCCTCAGCAAGACGCCGTCGTCGAGGGGTCGCCGTAAGCCGCCGCC
GCCGCCGCCGGCGGCGGAGGGAGGCAAGAGGTTGGTGCGGTTCTGCCCTGTGAGCGTGGTCGTCGGCG
AGGACTGCCGGCCTTGTGGGCGCAAGTGCCTCCACGACGCCGATCTGGCGGCGGCGGCGGTGGCGGGG
CCGCTGCCCCCGCCGGCAGTGGCGAGGAGGGTGGAGGAATTGCTCCGGGGTTTGGAGAGGGAGGAGGA
GGAAGGGGCGATCAGCGATTCGAGCTCGGATCTGTTCGAGCTGGAGAATTTGACGGGGATCGGACGGT

ATCGGAACGAGCTACCATTGTACGAAACCACCGACATGGGAACCAATCACGCCATCGCCCGGGGTTTG
GCTGTGTAA

>Pda_XP_017697775.1
ATGGAGAGGTGGCCAAAGCAGTGCGGGAACGTTAAACCTTCGTTCGCCTCGGCCCTCCTCGACTTCAT
CGACCGGTCCATCGACGAGACCGACGGCGGAGCAGTGAGAGAGCACCGCACCGGCGGAGCGCCTGACC
GCCTCTACGACCGTGGCCCGGCCGCCGTTAAGAAGCAGTGGAGCTTCGTAGCCGACCGGTGGCCAGTG
GAGAGGAGAAGCAACGTACCGGTGGCCAGTGGAGAGGAGAAGCAACGTACCCGACTTCGCAAGCCCGA
TAACCGCCGTGGTTTCTTGGCGAACTCGACTTTCATCTCCTCTGACTGCTCCAGTTACGGTGGGTTCT
CGTCCTCCAATGCCGAGTCGGTGCCCCACCCGGCCGGGCTCCGGTCGATCCGCATCGGCAGTTTTCTT
TACCGGTCCGAGGAGGCCATCTCGAAACCCCCGGCTCCGCCGCGGCCGGTGGCCGCGTCGCCACCAGC
GCACCCCCACCAGGACAAGAAGAAGTCGGGCTTGATCCGGAGCAAGTTCCGGGATCTGAGGAAGTCCA
AGTCTCCGGCGTCGCTGGGGGCCCGCCTCGCCAGCTTCCTCAACTCCCCCTTCACCGCGGCCGCCGCC
GGGCCCGCCTCTGACCCGCCTCTGGCAGGGATGAGCTTCCGGCGTACGAGACCACTCACTTTGGCACC
AATCGCGCCGTCTCCCACGGTCTCATCGTGGAATTAA

>Pda_XP_008785744.1
ATGTCCACCGTTGGGCTGCCAAACACCGACAAGAGCCTCTGCTCGAGGCCGGCTCATCGTCGACAAGA
TTCTGGTGAGATTGATATCTTTGAGGCTGCGCTTTACTTTGCCGGTGGCGTTGACGGTGCTGGTCTCC
CCGGCAGAATTGGTCATCAAAGAGTCATGAGGGAAGACAGGGTGGGTTGGAGGGCAGAGAGGAAGAGT
TTGGACGCACCAATGAGCATCATACTTCCCCAAGAGTGCCAAAGGGTGGAGAATTATGACGCGAAAGA
GAAGAAGGGTAAGCAACCAAGCTCCCCAGGTAGTAAGTTGGCTAGCTTTCTAAAATCTATATTCCACC
AAACTGCTTCTAAGAAGAAATCCAAGTCTCTCGCCACCACTAAGTCATTGAAGGATGGAGAGGTGGAG
GATAGACATGGAGGCAGGAATAGGAGGAGGAGCATTGGCCATTCCCAGAGCATAAGAAGCAGCAGTGA
CTCAAAATCCATCTTCTCTTCTGAGAGCAGTGTATTCAGCACCCCTGCTCCTCACGCTAACATCCTAA
CAAAGTTGCAAAAGAAACAGAGCAGGAGCTCCAAGTCCAATGGTCAGCCAAAGATG
GCAACTTTCTACCCACAAGGAGAGGTTTTGGATGATAAAAGGGTAGAAGGGGAGAGTTTGATAGCTGA
GAGAGCTAAGTCCAGCGATGGACTTTCTAAGAAAAGCAAGGTCTTTGAGGGTGGGAAGCCTGATGTGG
GTTGGAATGAAGGATGGTTTTTGGAGAACAGATGGGTCTTGAATGGAGATGAAGGAAAGCTCTTCAAG
AAGCATGGTGAATTGGGGGAGGTGTTTAGAAGAAAGGAGAAGGAGAGTAGGGAGGAGGATGGAGGGGA
GAGTGACTCCAGCTCTGATCTGTTTGAGCTGAAGAACTATGACTTTGGGGAACTCTCAAGTGGATTGC
CTGTTTATGGGACCACAGATATGGAGATAATTAAGACGGGGGCTTCCATTCACAGAGCTGCATTTTAA

>Zma_GRMZM2G027519
ATGGAGCGGCGCGGCCATTGCCACGGCGGCAAGCCCCCGCACCCGCACCCGCCGCCGCGCCGGGTGCG
CACCCGGCAGCCGTCCAGCGGCTCCTTCTCGGCGTCGCTCCTGGACGCCATCTACCGCTCCCTCGACG
ACGGCGACGGCGCCGACGTCGTCGTTGACGCCGCGCGTGGGAGTGTAGAGGAGAAGGCGGCCGCGACC
GCGACGGCGCAGTTCTGGTGGTTGAACAAGGCGGCGGCGCCCAAGCCCTGCAGGCAATCGTCGTCGAC
CGCGGACAGGGACAGGCGCCGGCGGGAGGCGGGAGTTGCGCGCCCGCGCCACTCGGGGTCGGGGTACG
CGTCGTCGACCGCGTCGTCGTCCGACTCATCGGCTGCTAGCTACAGCAGCCTCTCCTGCTCGTCGGCG
TCGACCGTAGGTATCGAGTCTACGTGCCGCCGCCACGGCCTGCCCCCGCCGCGGGTGTCATTGTCAGA
GGAATCCGTCGCCACGGACGCGGAGGAGACGACCCCGCCGCCACCCAACAGCAAACCAAAGAAGAAAG
CCAGGCCATGTTTCCCCGTGGCAAGAATCCGACCAAGGGCCTCAGTGCCACCACCATCGTCCGGGCCA
CAGCCGCCGTCGTCGCCGGCGACGTTCGCGTGCGCACTCAAGGCTCTGTTCTCCTCGGCGCGCCTCCA
GAGGAAGCCCAAGGCTCCGGCACCAGCAAGAACGACCCCTCCGCCCAAAATCTCGCATCCTCCGCGCA
TGTCGACCACGAGCGCTGCGAAGGCTGCTGATGCGCCGCAGCCGTCGGAGCCGACGACGGTACGGCTC
CACCCTGAAGCCGAGGCGTCGGTGGTGCGACGAAGGGTGGAGGAGCTGGTGCGGGGCCTCGAGGAGCT
GGAGGAGGACGAGGAGAGGAGCGACGCCAGTTCCGACCTCTTCGAGCTGGAGAGCCTTCGTGGCGCCG
GCGCCGACGAGCTGCCCGTGTACGGCACCACCAGCCTCGTGGCCAACCGCGCTATCGCGCAGGGGCCA
GGCGGCCAGCTCGTTAATAAGTAA

>Zma_GRMZM5G843781
ATGGAGCGGCGCGGCCATTGCCACGGCGGCAAGCCCCCGCACCCGCACCCGCCGCCGCGCCGGGTGCG
CACCCGGCAGCCGTCCAGCGGCTCCTTCTCGGCGTCGCTCCTGGACGCCATCTACCGCTCCCTCGACG
ACGGCGACGGCGCCGACGTCGTCGTTGACGCCGCGCGTGGGAGTGTAGAGGAGAAGGCGGCCGCGACC

GCGACGGCGCAGTTCTGGTGGTTGAACAAGGCGGCGGCGCCCAAGCCCTGCAGGCAATCGTCGTCGAC
CGCGGACAGGGACAGGCGCCGGCGGGAGGCGGGAGTTGCGCGCCCGCGCCACTCGGGGTCGGGGTACG
CGTCGTCGACCGCGTCGTCGTCCGACTCATCGGCTGCTAGCTACAGCAGCCTCTCCTGCTCGTCGGCG
TCGACCGTAGGTATCGAGTCTACGTGCCGCCGCCACGGCCTGCCCCCGCCGCGGGTGTCATTGTCAGA
GGAATCCGTCGCCACGGACGCGGAGGAGACGACCCCGCCGCCACCCAACAGCAAACCAAAGAAGAAAG
CCAGGCCATGTTTCCCCGTGGCAAGAATCCGACCAAGGGCCTCAGTGCCACCACCATCGTCCGGGCCA
CAGCCGCCGTCGTCGCCGGCGACGTTCGCGTGCGCACTCAAGGCTCTGTTCTCCTCGGCGCGCCTCCA
GAGGAAGCCCAAGGCTCCGGCACCAGCAAGAACGACCCCTCCGCCCAAAATCTCGCATCCTCCGCGCA
TGTCGACCACGAGCGCTGCGAAGGCTGCTGATGCGCCGCAGCCGTCGGAGCCGACGACGGTACGGCTC
CACCCTGAAGCCGAGGCGTCGGTGGTGCGACGAAGGGTGGAGGAGCTGGTGCGGGGCCTCGAGGAGCT
GGAGGAGGACGAGGAGAGGAGCGACGCCAGTTCCGACCTCTTCGAGCTGGAGAGCCTTCGTGGCGCCG
GCGCCGACGAGCTGCCCGTGTACGGCACCACCAGCCTCGTGGCCAACCGCGCTATCGCGCAGGGGCCA
GGCGGCCAGCTCGTTAATAAGTAA

>Sbi_Sobic.002G218300
ATGGAGCGGCGCGGCCATTGCCACGGCGGCAAGCACCCGCACCCGCACCCGCACCCGCCCTCGCTCCC
GCCGCCGCGCCGGGCGCGCGGCGGGGAGCGCACTCGGCAGCCGTCCAGCGGCTCCTTCTCGGCGTCGC
TCCTGGACGCCATCTACCGCTCCCTCGACGACGGCGGTGGTACCGACGCCGTCGTCGTTGATGCCGCG
CGTGGGAGTGGTGCGGAGGAGAATAAGGCGGCCGCGACGGCGCAGTTCTGGTGGGCGAATAATAAGGA
GATGGCGGCCAACAAGCCCAGGCAGTCGTCTTCGTCGAGCGCGGACAGGGACAGGGACAGCAGGCGCC
GGCGGGCGGAGACGGGCGTCGCGCGCCCTCGCCACTCGGGGTACGCGTCGTCCACCACGTCGTCGTCC
GACTCGTCGGCCGCTAGCTACAGCAGCTTCTCCTGCTCGTCGGCGTCGACCACGGATACTGAGTCGTC
CACGTGCCGCCGCCACAGCCCGCCCCTCCCGCCGCGGGTGTCGTCGTCAGAGGAATCCGTCGCCACGG
ACGCCGAGGAAGGGGCAACCCCGCCGCCGCCGCCACCCAAGAGCAAACCGAAGAAGAAGTCCAGGCCG
TGTTTCCCCGTGGCCAGAATCCGACCAAAGGCCTCAGTGCCAGCATCATCATCCGGGCCGCAGCCGCC
GTCGCCGGCGACGTTCGCGTGCGCGCTCAAGGCTCTGTTCTCCTCGGCGCGCCTCCAAAGGAAGTCCA
AGACTCCGGCAGCCACTCCTCAGCCCAAAACCTCGCATTCGCAGCCGCAGCCGCAGCCGCCGCGCATG
TCGGCAACGACGAGCGCTGCGAAAGCGGCTGACGCGGCGGCGGAACCGTCGGAGCCGAGGACGGTGAG
GCTCCGCCCGGAAGCCGAGGCGTCGGTGGTGGTGCGGCGAAGGGTGGAGGAGCTGGTGCGGAGCCTCG
AGGAGCTGGAGGAGGACGAGGAGGGGGAGCGACGCCAGCTCCGACCTCTTCGAGCTGGAGAGCCTTCGC
GGCGCCGGCGCGGACGAGCTGCCCGTGTACGGCACCACCAGCCTCGTGGCCAACCGCGCTATCGCCCA
GGGGCCAGCTCGTTAA

>Aco003950
ATGGAGAGGTGGAAGAAGCCGCCGCCGCCTCGGGCGCGGCGAGGGTATAGCTGCGACCACCCCTCCTT
CTCCTCCACACTCCTCGACGCCATCTACCGCTCCATCGACGAGCCCGAGGGCGCCGGCGCGGCGGCGA
AGCCCCCGCCGCCGCCCGCCGCCGCGTGGAGGAGCGCCACCGTGCGGGAGCGGGACGCGGCGCCGCGC
CGCCCCCAGCAGCAACCGCAGCCGCCGCAGCAGCAGCGCCGCTGCGGCGCGTGGCCGACGTACTCGAC
CTCGAGCTCGTCCGAGTGCTCCAGCTACGGCGGGTTCTCGTCGTCCGAGGCCGAGTCGTCGGCGCCCG
GCTCGGCCCGGCTCAAGCCGATCCTCCCGGAGAAGAAGAAATCGAGCTCGATCCGGAGCCGGATCCGG
GAGTTCCGCAGATCATCGAAGGCCCCCGCTTCCCCGGGGGCGCGCCTCGCGAGCTTCCTCAACTCGAT
CTTCGCCGCCGCGGCCGGAAACCCTAGGAAGCCCTCCAAGCGCCCCGCCTCCGCCGCCGCCGCGAGCT
GCGGCGAATCGGCCTCGGCGTGCTCCACGGCGTCGTCCTACTCGCGCTCGTGCCTCACCAAGACTCCA
TCGACCCGCGGCGGGCTCCCCCCCGCGGCGCCGCCCCCGCCGCCGCCGAAGAGATCTGTGCGGTTCGG
CCCCGTGAGCGTGATCGTCGACGAGGAGCTCCGCCCCTACGAGAAGAAGCTCCCGTACGACGGCGATC
CTCTACCAATGGCGAGGAAGAGAGCGGAGGAGATGGCGCTAGGGTTTCATAGGGAGGAGGAGGAAGAG
GAGGAGGAAGGGGAGAGCGATTCGAGCTCGGATCTCTTCGAATTGGAGAGCTTAACTGTGACGGGTGA
CGAGCTCCCGGTGTACGAGACCACCAGCTTCCTCACCAATCGCGCCATCGCCCATGGTCTCATCGTGT
AA

Table S3. The promoter sequences used for analysis.

>OsaBGL1
TTATCTTGTTTCAATACGACAAGCTTTGACTCAACATTGGTAACTTTGTCTATATAGGAGTATTATTT
ATGTGCTATAACCTTTTGAATTCGAATTTAATAGCATCAACTTTTATGTCAAAATCGTATCCTGACGA
TACAAATTAAACATGTTTTATTGAAAAAATTTGTATTCTGACGATAGAGTTACCCCTCTTATCACTTG
AGAGTAAAAACCTTAAAATGATTAAACAGATCGAAGAAAAGTGAACGGCCAACACATTACAACTTTAA
AAAACGAATGATTAAACATGTGACTAAAATTCAATAGCGTCATTTATTAAAATACGAAGGTACAGTAC
CTCATAGAGTTATATCTCCGGAATAGTACCAACAATAATATCTCTGAAAGCTAGGGAAAGAAAACTGG
ATCAGCAAATACAAATGGTGAATTTCCATTCCTGCTTCTACTTGTGGTACTACCTCCATATTTTAATG
TATGACGCCATTGACTTTTTGTCCAACGTTTGACCATTCGTCTTATTCAAAAAATTTATGTACTCTCT
CCGTTTCACAATGTAAGTCATTTTAGCATTTTCTATATTCATATTGATGTTAATGAATCTAGAGATAT
ATATCTATCTAGAATCATTGATATTAATATGAATGTGTAAAATGTTAGAATGACTTACATTGTGAGAC
GGAGGAAGTAGCTATCATTTATTTCATTGTGACTTGATTCGTCATCAAATGTTTTTTAAGCATGATAT
AAGTATTTTTCATATTTGCACACAAATTTTGAATAAAACGAATGATCAGACGTTGGTCAAGAAGTTAA
TGGCGTCATACATTAAAATGCGGAGGGAGTATTATATATTCTGATACCTTTTCTATGGACAAATCTCG
CTTACCTTCTCAAAGATCAGAGAAAACGTCTCAATATAAACACTAGTCACCCGGCCAACTGCATTGGT
CCATCCTATATCCAACATTAGTTCGATACATATATATTGTATAATAAT

>OsaBG3
GGGTTCAGGTTTATAAGTTAGTTTTTTATACAAGCTTTTGCGTCGGCACTGGCCTGCACCATCATGAA
CCGTAGTTAGAAAAAGAGCACGATTCGAATATCTTACAAGGAAAGAAATTGTTCATTGTATCCAGCAG
AGAAACGATGCACCACCCCCGCGCCTTTCTTTGCTCCGGGAAAGAGCTCGCTTTGCCGCTCGACGGAA
AGCTTCTCTGTTTTATGAACTAACTAACGACACGTACGTTAATCTAGCATCCCTAGCATCCGGCTAAA
CGGGCTAAAGTAAATGGAAATCAGTGAACGGAGCACATGCATGCCATACGGAAGGGCCCTAATAGTCA
CAAAAATTTCCGATCGTCCAAATACCGTTTTCATACCGAGCGTCATTTTTTTTATATAAGAGGTACCG
TTTACAACCGTTACCAAGTATTGCAAATTCTAATTTCTTTCTGCATCATACTGATATCAACGCCGAGT
AATTTAAATGATAAATATGATCAATCACCGATTGATTGAGCGTCGGTTTCGAAGAGATGCTATCGATT
CTCGTTGTTTTCACTCACTACATCTTTTTTTTCGCGAACTCATCACTCACTACATCGATAGACTGTTT
TACTGTTAACGCTTCAATTTCCACTGCGGGAGAAAGAAAATGTACAAATTGTCGACGGGCAGTGTCTT
TGCATGACTCGAGTTTCGCTTTACCAGCACGCCGTCACCGCGCGCCGCGCAGAGCCCTCACCGTCCGT
TCCTCCCCAACAAACTCCACCGCTGGGGAAGAGACGAGCAACTAAAAAAAAAAAAAGGCGAAAACTCGA
GTGCGCGCAGTGTCATCCGCCCCCGTTTCCACCTGTGCGCGCGCGCGGCGTTTTTCAACCCCCACGGA
CACCTCCGCACGAACGCACCAACCAAACCAAAAAGCCCCTGCGAATCTGTGATCCTCTCTCCCTCGCC
GCGATCGCGTTCGTCCCACGGCCGCGCGCGCGTCGCGGGGGCGTCACT

>OsaBG4
ACGGCAAAGATACAGAAAGAAAAAAAAACAGAAAAAACAAAAAGAAAGAAAACCGTCCAAAAAAAAAA
GAAAGAAAAATCGTTCGGAAAAAATAAAAACCGCACCGCCCGCGCCACCTCCGCGACACGCGTCCAGT
TGCGCGGGGGAGGGGGCGCGCGACTGGTCGCCGATTAGCGTTCTCGGCTACCATGACAGGACAAACAG
ACTCCTGCAACTTTAGAACGTGTTAATTAGCTGACTTTCCCATATTCTTCTCCCAAATTTTCACACTC
ACCTTCACTGAACTGATAAACGATGTGTTTTCTATTTTTTAAAAAAAATCTAGAGAAAAGTTGCGTTT
AGAATAAATTATATTAATCCATTTCTCATGTTTTTAGCTGATACTTAATTAATCATATGCTTGAGCTA
TCTCGTTTTCTACATGGGGACAAGTTCGCAACTTCTCCTCCCAAACATAGCCTTAAGCCATTCTCAAT
AAATGAAATGCCATGTAAGCAAATATAGTGACATGGCAATATAATTATAAAGAAAGGGAGGGAAAAGT
TTCATCTAGATGAAACTGGGTGTACACAATTTACAGAACTATGAAATTGGTTAAAACTAGCATTGGGG
GCTAGAATATTTCATTTCATCCAACCATATCTAATCCCATGCATCGCTATTAGGGGAATGTTCTTTTC
TTCAACTCCAACCTACAACTTATTCATTGTCCGTTAACACGCTTTCAAAATGTTAAATGATGCGTGTT
TTTAAATCTTTTTATATAAAAAATTCTTTTAGAAGTTCAAATAAATTCATTGTTTAAGTTTGTCCTAA
TTACCTGTCACATTTTCCGTGCCATAGAAAAGCTCAACTCAAATGCCATGACTAATCTATGAAACCGC
GAAATGAAATTATTCACTGAAGACTCTCCATTTAGAAATGGCCTTAGACCGCGGGGCCCTACAAAATT
TGTCAGTGAAGGACATGGGCAAGCTGAGCTGGTCAGGGGGCCGCCGGA

>AtrBG1
AAGCCGCCCCTGTCATGGAGCAACGAGCAACGAGCAACGAGCAACTTGAAGCCACGCTGGCGCCTCTCT
ACCTGTCTGCTTCAGGCGCGCATGACCACTCTCCTCCTTAAAAGGAAACCCAAGCGTGCTCTCTCTCT

CTTTCATTACACCCCGCCCCCCCCCCAAATCCCTCTTTCTCTCTCTACCCCAAACCCCCCCCTTCCAT
CATAAACTCTCCTCACTCTCCAGCCCCCATAACTCTCTCTCTCTCTCTAACAAAAGCCTCTCTTTCTC
TCTCTAAAAAAAAAAAGCCTCCCTCTCTCTCCAGAAATAAAACTCTTCCTCCACTGCCAACTCTCTATA
CACTCCCCCGGCTTTCTCTCTATACACTCCCCCCATCCCATCCTCTCTGTCCTGTTACAACTGCCCCT
TCTCTCTCTCTCTCTCTCTCTCTCTCTAGCCCCCATTGATTGCTCTGAGCTCTGCGAGAAAGAAAAAA
GAGTAGAAAATCGAAATCACGCCCGCATTTCTTAAACCACACGAACGTACCTCGACAATGTCTGCCCT
TCTTCACGGGATCGGACTCATCTCCTCCGCCTACCAATCGCCACCTCTCCGCAGCTTTTGATCGTCCT
GTACCCGACAAAGAAGGAAACACACACATTACAATAACTCTCCCTTCTGGTGTCCATTTTCTCTCTCT
AGCTTCTGTCTCGTATTTTATCTCTCTTTGACATCTATATATAGCGGCTGGCCTGTACTGGTTTTCTC
TCTCTAACCCATTACCCCAAAACTCACAGAATCGAAGCATCTATCCTGTGTCTGTTTGGGCACAGGCT
CCTCTCACTAGACTCTTGCCTTTGTTGAAAAACTGCCATTTTTTTCTCAGTCTTCAGACGTTTAACAT
ACACAAAACCGGCTTTCTCGCACTACCCCAATTCGATCAAACCCAACCAGCTGTGAATCCTCTTCCTA
ACTGCTGTTTTCTCTCGCTAGGAAGGCCATTTTTGGGGCCTCTGCAAA

>AtrBGL1
AATAAAAATCTGTCTATTTCTAAAACGGGTAGGGGCATGTAATTGGCACCGAGGTTAAAAAAAAAAAA
AGCATGAGAGTGATAGAGAGAGTAGGTTTTTTTTTTTTTTTCTTTTTTTTTTTTTTTTTTTTTGGAGA
GAGAGTTGTGGTTGGTCTTTGTACATGTGGTTGGTCTTTTTTTATTGTTCATTTCCCAATCTTGCGTT
TAGCATGTAGCAGTAACTAAGAGAGAAAGGGAGAGAGAGAGAGAGAGAGAGAGGAGGGAGGGGAGGGA
GGGAGGTAGACGGACAATAGTAGGAGTACAAGTCGTAGTTAAGGTAAGTAGGGTAAGGTAGGTAGTCA
CTACTCCGTCCGTCGGGTTTACGTTTACGTTTCGTTTACGTTTACGTTTACGTTTACGTTAGTTACGT
TACGTTACGTTACGTACGTAGTAGTAGTAGAGAGAGAGAGAGAGAGAGAGAGAGAGAGAGAGAGAGAG
AGAGAGAGAGAGAGGGGGAGGGAGGGAGGGTAGAGGCCAATATAGGAGTCAATGCATTAGGTAATGGA
AGTAGGATGCATCTCCTGGGCATTCCCAAGAAGTGGAGCAAATAAAAACTGATGGTTAGATGCATCCC
CTGGACCAAAGCCTAGGACTGGCAGTAAAGAAAAAGTTAAACATGGTTTCCCTTTTCAGCATCAACAA
ATGAATCACCAGCAAATTAAGAATTTATGGGACTCTAACTCTTCAAAAAGGTGAGGCCCTCATCTACA
GGAACACTCAACAGTGCTTCAAGGATATAAAGTGACCTTCCACTGCTCTCAATGATTCCCAAACACAC
AAAGAACCTCTCTCTCTCTCTCAGAGGGGAACAGATAAATACCACCACACCTTTAATGTGACAGACCC
ATTCATGCAAGTATACACACACATTCCCTCTACCTCTGTCACCATTTCTCTCCCCAAATCTCTATCTC
ATTATCTTTATATCTCTCTTTTCTCACATACATAGACAGATAAAGATC

>AthBG1
TTTTTTAACTGTCTTGTATTATTATGTTTAGGTAAAAGTATGGTCCAAACTAGAAACAATATTTACGG
GGGAATTATTCGAAACTGGATATGATAAGGAGATGTCAAACGAAAAACATTGGCCGAAGTGTGACAAA
GATATATAGAATAGACACACAAGAAGACATAAGTGTTTTTCATAACGAAAAAAGAAAAAAAAAAAGAA
GAAGAAGACATAAGTGTTTATGAAATGAGGAACAATGAAGGAACAAGAAAGGAAGGAAGCATGTGAGA
CTGAAAAAGACGCAAAACAAGTACAATTCCTTAACCGGTCCTTGTCGACCCAACCAATGAGCAATGTG
GTGTAAGAGACATTTTGCTTATATTTAAACATATTAATCGTTTGATTAATTAAGACCCTACTTGTTCC
TAATCACATCCTTTTCCTAAACCTATATACGCTTGAGATCTGTCTAAACAATATGAGACAATCCGTAA
TGATATGGCTACATCAAGATTTCGGCAAATCGTTGAGAAACAATTGTTGATGTTATTTTTCTAAAAGA
GATGGTTTTGTTTCATTGATCCAATGCAATCATTCATCTCCAATTGTGTCAAATATACAAATTTTAAT
GATTACTTGATTATAAAGAATCAAGAAACAACTTCTGAGATACATTTCTATAGGAAAATGTTGAGATA
TAATCTCCATAAAATCATTAGTTTCTCACATTATGAGGAAAATGACATATAAATTGGGCATTGGACAA
CATATTATTAGGTACATGCACACATAAACAAATTGAAGTATCCTCTAGTATGGTCCTAACATCAATAT
GGTCAGCATACGTGAACAACACGCGCCGAGATCGTTGCTTATATATACGTCCTCTCTCCCTCTTCTTC
TATCTTCATTCGTCGTCTTCTTTCTGCAAGAAAAACAAAAAAACAGTTACGAGACATAATCTCTCTTT
TTCCTCCTTTGTCTCTTCTTCTCATTCTTCTTTGTTATCGATCAAAAC

>AthBG2
AAGATTTGATGACATTTAAATTAGCATTTAAAACTAAAATCGTTTTAAAATCAAATAATTCTAGATAT
GGTTAGTTAGGATTGATTTTCGAATTTTGCTAATTAGGAAATAATCATTCATTAATTCCGTAGAGTTT
TAGGGATTGATTTGTGCAATTTATTAGGGTAGTAGTTAACAATATCTATGTTTAGGCAAGTAATTAGT
GCAATATCTTAATTGTTAATAAAAAATTTGGTCATTGTTCTAAAGAAAATATCTTGTCTGATTGATTT
TGTAGGGACTAAACATACAATATCAAAATTGAAAAAAGATTTTTCGACACAAATCGTCTCCAAAAATG
TATATGTAGATAGATTTATAAGTGTCATCAACCGCTTGAGTTACTTATGAAATCCTATTTTGTTTGAA

TTTTTTTTTTTGATATATTTAAGATAAAAAAAGTAAAATAAGATTACATTTTTCAGTGGATTTAATAA
ACAAATGGTTAGAACTAAAACAAGAAATATGAGATCTAAAAAAGAAAGGAAGCTAAGTAAATCTCATA
GAGATAGAGATCTAATGAGGATGTCTACAAGGATATTACTGATAGAAAAAAACAAAAAAACAAAAACA
AAAGAGTGTAAACATAGTAGCACACACTCCCTAATGCATCTAATAATTACCCGCACGCATTAACATCT
CTCACATGAGTCTAATGAGAAAGTCTCTTAGAAACAAAACACCCAAAGCATGTCTCATCACTTGACCA
GCTTTTGTCTTCGTCCCACAGACATCTCCACTCTCTTTTGTCTTCTCTCCACAAACCCTAATCACTAT
TAACACACAAATCACAGCTTTTCAAAACTATATATTATATCCAAATATCAATATATCCTTTTTCTAAC
TCTCATCAATATGTCTTGGCAGCTTTTGATATTTTCTTAGCAGACAAACACACTTGAGCTGTTTTTTT
TTAATCTATAAATACTCTTTTTTTGTTGATAGCTTTGAGATCTCTCTC

>AthBG3
TGTATAATACTAATGCCAGTATTTGGTAATCAACATTACGTACGAATATAAATTTAAGAAAGGAAAAG
CTGTAATGGACCCAAGTATCAATATTTGATTTGCTGTTATGAATCAACGATTATGTTTATTTCGTGTT
ATTAAATTACACGTACTTTAGTTTTGTTCTTTGTTGTTTTTAAAAAGAATGACTTGTCAAGAGACATA
AACATTACCACTAAAAGAATTATACAATTTTGTATTACCAATCGTATTTATAATATTTCTGAAGATAA
GAAACAACCAAACTTTAACTATAACTAAGTAGTGATAGCTAATGGGAGTGACTAAGTTTTATAACAAG
TTAATTACTAGTTATTTATACTAGTACCAACTTATCTTAACTTGGATCTAAATTTTGTAATTTATCTG
TGTGAGTTGTATACGAAAATATATACGCATCGATAAATATGAATATTATAGAATAAGAAAAATATGGA
AAAAAAATTCACTTAATTTAACGTTTTCTTTGTCTACATCAATTTTTTTCTATATTCCTAAAAATTGC
ACTTATTACGTTTTATTTTTGTGCTTTGACTTTTCTGGAAACAAAAATGCAGAAAGTAAATTATTTTC
CCACAAAGCTACTACATATATATTGAGAAAAATAAAGAAATTAAACTTATATTTCTGACAAAATTAAA
AATAGAAATGTTGTTGCCAACAACTATATTATTCCAGTCTGGCACCCAATTCCACCTACGCAAAAACA
GTAAATAAAATAACACGTGGCAATAACACTATTTTGGCTGATAAAGACTCTTTCACTTTAATTCTTCT
TCTTCTTGTTCCTCATACACACACACTACTACTACTCTCATCTCTCTCATATCAAACAGAAGAAACAA
AGCGTGCCTCTTCTCTCTTTCTTCATCATTCTCATCAAGCTTTCTTCTTCACCAAATTCCCAACACTA
TCATTCCCTTCTTCCTCTATAAAGAATCATCACTTCTTCTTCTTCTCA

>AthBG4
TTCGGTATTTTGTTAACCAAACCATATATCCTTAATTCACTTTTATATTTGTGACTATTTATGTCTTC
ACTTAAAGTTTTTTTTTCTTTGATTAGTGTATCTCTCGAGTTTCGATCCTTTTCCTAGATTTAGAGTT
TCTCAAACTTTCTTAATGCCTCGTGGTTTTTTCTTGAAACCATGATCAAAAAGTATCGCACCGATATA
GTTTTCTAATAGGTTTGTTGTCCAAAAAGCTTCACAAATAAATTATCAATACTATTAATGTTGTGTAA
TTAGTATAAAAAATGTGTATGAAACCATTAGAATGCTTGTAGTTTTGCACTGTTTAATTAGTGAGTTT
GGAAGATAAATAACCAAACAAAAAAAGTTTTAATGGACCCAAGAAATCAATATTGGGTTTGCTGAAAT
AACTCACATATTTTTATTTTTCTAGTACTTAAAGAAAAACAAATAAGACTTATGATGAGGAAGGAACA
ACTTTAATCTTTTACTTTATAAGTAGGGAACACATATAAGTGAATCTTTGGGTCGTGTCATGTGAATA
AAGTCTAAGCTGTTCTGTAACCCAGCAGTGATATCCCATAATTCTTTTGGTTCAAGACAAAATTCTTA
ATTCTTAAACCTTACCATTTTCATACATATAGATCTTAGATCATTTTACTAAGCTTTGATTTTTGATC
TTTCCGATATTCGTCTTGCATACCGTAATCCTATAAACATTTTAAACAACTTTTCTCCCAATAATAAC
ATGAAAGAAACATCAGTTCTGTTTGATCATGTATATATGTATTTTTACCATGAGTATAAAAATATTTT
TAAAAGAGAGGGTAAAAAGATGATTGGTGGTTTAGTTTTGTGGGGGACGAGGCAGAGAGTTGTCGAGA
AAGACACGTGGCAATGATTACCACTTGTCTGCCTTGTTATAAACAACACACTCTTTCTGTCTTCCTCT
GCTCTCTCTCACATATCCTTCCTCCTCCATTTCCTCTTCTTCCCTCTA

>AthBGL1
AACCATGGTGGATGAAATACAAACAGTAATCACAGATAAATCTTTGAGGAAACATTGTCTTTTCTTAA
CAAGGTACACATTTCAGCTTGTAGTCTACTCGATTTGGAGAGAGGAAAACAGTAGAAGACATGAAGAA
CCTCCAACTCCGCCAGCACAACTAATTCGAAGTCTCGACAAACAGGTTTGGAATCCTCTATACGTGAA
CAAGGAAATAGACTATGTGACGGCTGTATATATGGAGATGTTTGTTTCAAGATAATCAACAAGGAGAC
TAGCGTTGACTAGAAAGTTTGCTTAAAATCTTTAATCGACCGATGTATTTGCTGTAACGACATCTTTT
TTTTGAATAAATTTTATATTTTATAAAAAACCTATTTAGTATACATACATATATACATATATATTTAT
ATTCATATATAGAAATTTAATGGAGTCATGGGTTGTGTGTGTGGTTTGATATACATAAGCATAAAGAT
TTTCAAAAATGAACGACAATATATTAAATCACAAAAACACACACTCGAAGTTGGAATCCAAGGCAACA
AGAGACGATAGGTCCACATTATCATAAATACAATCAATTAAATTTCCTACACCTTATCAGAAAGATCA
TAAAATCAAATGAGCCCACCTTCTCCTTCAATTAAGTTCTTCACCCACGCATTTAGTGGGTCACACAA
ACTCTTATTACTTGTGTCCCTCCAAAATTTCTAATTTTTTCCGCATTCAAAACATTTAATCCAAAAAA

TATAAACCAATCGTAATCCACTAATATTAACGTATGAGTATCGCATGATACGTATATTCACATACTAC
GACCTTATTTAATGATGAAGGAGTTCCATATCACATATTCACATCAATTGTTATATAAAACCTAAACA
CATTATATTCATTATATCATTCCCTAGCAACTTGTTCCTCATTCTCCGCAAATCAAGCATAGAAAGAG
ACTCTTGGAGATCATCCAAGTATCCGATTCTCAGACCCTAAAAATATA

>AthBGL2
AGTATCAACAAACATACAAGTTCCATCTTGTTCCTTCTTTTGCATACACTGGAAAGATGTCAGAGTAG
AAAAAAAGAAGAAAAAGTAAGTGATCCAACACCAGTTCATGTACACCATTAATTCTTTGACTGCTCTC
TTTGAACTTGGTCACAATTGATCAATCAATTATGGTCATTTTATGTCTGTTGTTCGCCTTGTTCGTGA
CGTAACTGTCGAAACAGTTGAAATCCACCTAGATAAAAAGAACCAATTACTCAAAAATTTAAGGAAAT
CATAATAATAATAAGAAAAAAAAATAATGTCATAAATTATTAAATAATATTAAGCATATGAATAATTTG
ATTATGATATTAGTTTACAAATTTAGTGAATATATTTGTTTCTGTTTCAAATAATCATATCTCTACTG
TAAAATAATTATGAAATAACAAAACTTGACAATATGATGTATAAACAACACATGTTTAGATATTATTG
CAACGCTTCTCACTTAAGTTCTCTTTTATACTATAGTAGAATGCTATATAGTCCGGTAACAATTTAAA
TAAAATATTCTTAACCAACTTTCCCAAGATGCAAAGAAAATTGATTTTAGAACCTCTACTTTTTTGGT
ATCTACTAATACTATATATCTAATCATTATATAAAATTCTCCCACCAAATTGATCAATGGGTCATCTT
ATCTTTATCGCCTTTTGCCTTTGTGACCATAAAAGGGTTTGAGAACTTGCATGACAGCCGAAAAAGGT
GTGAATATTCATTGATATTAACGTGGCATTCCTATGAACACATAGAAACGTATAATTAAACTTGCTAA
TATTTTCATTTCACTATTATTTTCAACATCAAGTGGGATATTAATTTCTGAACTGAAGATAACTAAGA
TATTAACCGGTTCAGTTAACCGATTCCTTGCATTCTATAAATCTGACGCACCATATTTCTTCTCATAC
CACAATTACTAATTAACCAACTCAAATCTTGGAACTCAAGAAACAACA

>OsaBG1
ACTGTTTATGTTTGTTTCTCTTCATTACCAGTAGTAGCAACTAGCAATGGAGATTAGTGTTTTCTTTT
CCCGTTTTTTATTTGGTCGATGGAGCACGAAGTGGCTCGAGATCGGCTCACTGCACCGTTCGCCACGT
CCATCCGTTTTCTGTGCCATAGGCAGGCCCATAGCAGCAGGTCGGTGTCTGTGGATCAGAGCGGTGAA
CTCCCGTCACCGTCACTGCTGCAAAACGACGCAATACGGTGTGTGTATGCAGAATGAAGCCCAAAATG
GTACGAAACGAAACGGAATACTCGCACTAGATTACTGCGCTATAATTAGCACTAGTACTACTACTCCC
AGAAGTTATTGCCGCTGCTGCAATGCATGCGTGCTCACGTTTTGGGTGGGTGAGGGACAGAGGCAGAG
GCGACAGTACTACATGCGTCTCGATCGCCAGAGTTAAATCTTGCGACGTCGTACTCTACAGGGGGAGC
AGCAGCAGCGCGCGGTAGTATAGTTGCGTTTGCGTACCCTTCGATCTTTGATGGGCGGAATAGAGGAA
CGTCTGAACCATCGTAATGTATGGTATAGGACGACACATTCCCATTCACCTCGGTCTGAAAAAAAAAA
GAAGCTGAAATTCACTCAGGTATATGGACTGATTAGCAGAGGTCGTGGCAGTGTCAATTGTGAAGAAA
TGATAAGTTCACCGGCGGTGAGGGGACGAGAAATAAGGAGAGACAGGGAGAGAGACGGGATGGCCCCA
TGACCCCCATCACCGTCACCCGCACGCCCAAACCGAATTAGCCGGGCGCCACACCCCCGTTTCCACCA
CCCAAGCTTCCCCTTCCACCGCTATATCTTCCGCCCGATCCCTTCCCCTCTCACCCTTCTCTCCTCCA
CCTTTCCCCAACACGTACCACCAACTCACCGCGTACCAAGCCACCGGCTCAGCAGCAGCAGCTGCCAT
TTCACAGAGGCATTGGTTGCTGCTCTCTCTTCTTGGCGTTTGTTTCCT

>OsaBG2
ATGATGGACGACAAGGTGGCGTCGGCGGCGGAGTGGGGGCGCGGCGAAACTGACGCGGCCGAGGCGGC
GTCGGAGCTGGAGCTCGAGATGATGCGCTCCCGGTTCTACAGCAGCGGCTTCTAGCGGAGCCCCTCCT
CAGGCTCCAGCTCCCGCGCCTCCTCCCTAAGCCGAAAGATGACGACGATGAGGCTGGCGTCCTCCGCT
TGGTTTCACCGCCCCGCCGGCTTCGGGTGCCGCCACTCTGCCCACGCGTCCGCTCATCGCTGCCCCAC
TCCGTCCCACCAGCTTCGCGTGGCGCTGGTGACTCAGCAACGAAGACCAAGGTCAAAGCGCCACGGTG
GACAAAACCAGCGCCACGTAGGATAAAACCATGTGAAATACTGCCTTGGGACCACTTGCGACCGGTTT
TAGTAAGTTAAGGGACCCTCGATATCTGGTTTTGCAGTTCGAGGACGTTTTTTTATCTCGGTGACAAG
TTGAGGGAACTTCGGTGTACTTTTTCCTAAACACAAAGAACAAAGCCCCAACCCAACATCACCCACGT
CCGAAACAGCCCAACCCGTTAGGCAGCAGCCCATGATCATCCCGCCGGCGCCCATGGCGGTAAAAACT
AAAAACGGCGAGGCAAGTAAGCAGCAACAGTGAGACGTGAGAGCGGCAGGGGACACAGGTACGGCCAT
CCTCGTGCCGGCGCCGACAAAGCAAAAAGCTAACAAATAAAGCGGCCGCCGAGTGAGTGAGAGTGAGA
CGGCGAGGTGGAGAGATGGAAGGAGCACGAGGCGTGGAACGGAAGAACGGACAGACAGCGATCGCGCG
CGCGACACCGAGATGGCGCGGGCCCCGCCCCCCGCCCCCCGGCAACAAGATCCCCCCTCCCACCCCCG
CACGCCCGCCCGCCACTCCACTCTCCACTCCACTCCACTCCACCATCGAATCGAATCACGCGCGCCAT
TGCCATTGCCATCGCCATCGCCATTGCGTTCTGCTCTGTTTCTGCGCC

**Table S4. Primers used in this study**	
qRT-PCR	
AthBG2 F	GCGGTACTTAAAAGAACTTC
AthBG2 R	CAAAAGCAAGCTTCTAGCAA
Promoter cloning	
pAthBG2 F	CACCTTAGAGGAAGCAAACTATTGAGT
pAthBG2 R	GAGAGAGATCTCAAAGCTATCAAC
CDS cloning	
GWAthBG2 F	CACCTTAGAGGAAGCAAACTATTGAGT
GWAthBG2 R	CTACAAAAGCAAGCTTCTAGC
GWY14 F	CACCATGGCGAACATAGAATCAGA
GWY14 R	GTAACGTCTTCTCGGAC

**Table S5. The similarity of selected BG and BGL proteins with already known structures (The first three hits showing maximum similarity is given against each query)**				
**Query**	**Hit**	**Coverage**	**Confi dence**	**Percentage identity**
AtrBG1	c2lpbB (central activation domain of gcn42 bound to co-activator domain 1 of med15)	45% (13-39)	14.0	33
	d2gdqa1 (TIM beta/alpha-barrel)	19% (32-43)	12.6	50
	d1vzva (Herpes virus serine proteinase, assemblin)	52% (13-43)	11.6	31
OsaBG1	d2gdqa1 (TIM beta/alpha-barrel)	47% (25-48)	39.5	33
	d1rvka1(TIM beta/alpha-barrel)	45% (26-48)	24.6	35
	d1jpma1 (TIM beta/alpha-barrel)	39% (26-45)	8.7	30
OsaBG2	d2d6fc2 (GAD domain-like)	20% (30-40)	19.2	64
	d1zq1c2 (GAD domain-like)	20% (30-40)	18.8	55
	d1tdza3 (Glucocorticoid receptor-like)	33% (24-40)	14.4	53
AthBG1	c2jowA (protein prig)	13% (26-33)	22.5	50
	c4gf1B (putative adp-ribosyltransferase certhrax)	13% (27-34)	18.7	75
	c2l3jA (double-stranded rna-specific editase 1)	23% (21-33)	17.6	54
AthBG2	d2gdqa1 (TIM beta/alpha-barrel)	20% (30-41)	11.8	50
	d1r7la (Bacillus phage protein)	29% (22-38)	10.4	44
	c2lpbB (central activation domain of gcn42 bound to co-activator domain 1 of med15)	27% (12-27)	8.7	25
AthBG3	c5tscA (lpg2147 from *Legionella pneumophila*)	35% (22-41)	41.9	53
	c5sujB (lpg2148 from *Legionella pneumophila*)	35% (23-41)	39.4	58
	c1hllA (t3-i2, a 32 residue peptide from the alpha-2 2a adrenergic receptor)	19% (22-32)	29.7	55
AthBG4	c2lpbB (central activation domain of gcn42 bound to co-activator domain 1 of med15)	46% (11-37)	12.3	37
	d2gdqa1 (TIM beta/alpha-barrel)	19% (30-41)	11.6	67
	c4h8oA (parallel 6-helix coiled coil cc-hex-n24)	26% (38-53)	9.7	38
AtrBGL1	c2cw1A (lambda cro fold2 protein)	28% (22-36)	12.9	47
	c1uw7A (nsp9 protein from sars-coronavirus)	16% (12-20)	9.0	33
	c1emsB (*C. elegans* nitfhit protein)	83% (6-47)	6.3	33
AthBGL1	c3m92B (ycin from *Shigella flexneri*)	63% (18-54)	14.6	32
	c2cw1A (lambda cro fold2 protein)	17% (40-50)	14.4	64
	c2kukA (leaf cyclotide 2)	10% (37-43)	10.7	71
AthBGL2	c2z5cA (protein ypl144w)	39% (18-38)	8	24
	d1xg0b (Phycoerythrin 545 alpha-subunit)	45% (2-25)	7.8	40
	c2eluA (5th c2h2 zinc finger of human2 zinc finger protein 406)	15% (2-10)	7.2	67
